# Enhanced Optical Properties of Azaborole Helicenes by Lateral and Helical Extension

**DOI:** 10.1002/chem.202202280

**Published:** 2022-09-02

**Authors:** Felix Full, Quentin Wölflick, Krzysztof Radacki, Holger Braunschweig, Agnieszka Nowak‐Król

**Affiliations:** ^1^ Institut für Anorganische Chemie and Institute for Sustainable Chemistry & Catalysis with Boron Universität Würzburg Am Hubland 97074 Würzburg Germany; ^2^ Institut für Organische Chemie and Center for Nanosystems Chemistry Universität Würzburg Am Hubland 97074 Würzburg Germany

**Keywords:** azaborole, circularly polarized luminescence, fluorescence, helicene, π-extension

## Abstract

The synthesis and characterization of laterally extended azabora[5]‐, ‐[6]‐ and ‐[7]helicenes, assembled from *N*‐heteroaromatic and dibenzo[*g*,*p*]chrysene building blocks is described. Formally, the π‐conjugated systems of the pristine azaborole helicenes were enlarged with a phenanthrene unit leading to compounds with large Stokes shifts, significantly enhanced luminescence quantum yields (Φ) and dissymmetry factors (*g*_lum_). The beneficial effect on optical properties was also observed for helical elongation. The combined contributions of lateral and helical extensions resulted in a compound showing green emission with Φ of 0.31 and |*g*_lum_| of 2.2×10^−3^, highest within the series of π‐extended azaborahelicenes and superior to emission intensity and chiroptical response of its non‐extended congener. This study shows that helical and lateral extensions of π‐conjugated systems are viable strategies to improve features of azaborole helicenes. In addition, single crystal X‐ray analysis of configurationally stable [6]‐ and ‐[7]helicenes was used to provide insight into their packing arrangements.

## Graphical abstract

**π‐Extended Azaborole Helicenes**. A unique set of π‐extended azaborole helicenes, assembled from *N*‐heteroaromatic and dibenzo[*g*,*p*]chrysene building blocks, shows excellent optical properties. The lateral and helical extensions of the helicene scaffold significantly enhanced emission quantum yields and dissymmetry factors.

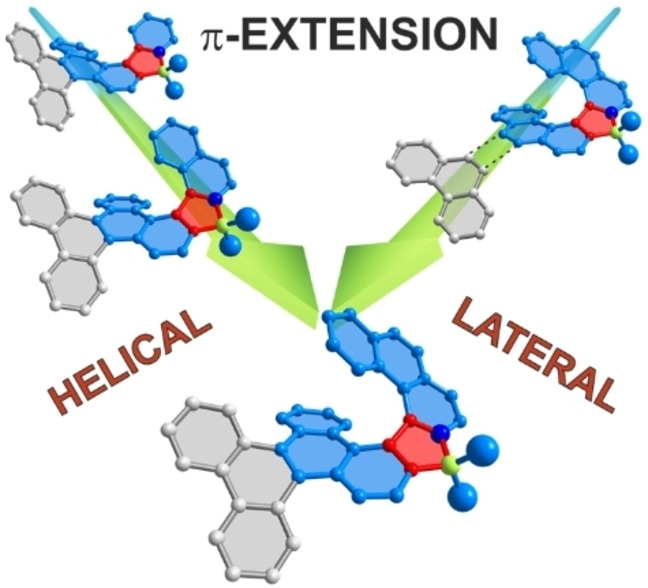

## Introduction

The rapidly evolving research of chiral compounds has culminated in an increased focus on helicenes, a group of polyaromatic hydrocarbons (PAHs) characterized by a screw‐shaped skeleton consisting of *ortho*‐fused benzene or heteroaromatic rings. Their complex three‐dimensional packing arrangement in the solid state, stereodynamic nature and mechanical properties of molecular springs, with the capability to modulate their pitch by external stimuli through the extension‐contraction motion, have been the focus of in‐depth theoretical and experimental studies.^[1]^ While helicenes constitute promising candidates as ligands or catalysts in asymmetric catalysis,^[2]^ the interest in helically chiral PAHs grew especially in the field of materials science.[^2a^, ^3^] Their inherent chirality in combination with the extended π‐system leads to appealing electronic, charge‐carrier and chiroptical properties, such as optical rotatory dispersion (ORD), circular dichroism (CD) and circularly polarized luminescence (CPL).^[4]^ These characteristics make helicenes applicable in various research areas, including chiral switches, and sensors,^[5]^ semiconductors,^[6]^ in addition to spin transport materials.^[7]^ Owing to their differential emission of left‐ and right‐handed CP light, chiral compounds show potential for applications as emitters in chiral optoelectronic devices. The utilization of chiral luminescent materials in CP‐organic light emitting diodes (OLEDs) allows for reduced energy losses as well as simplification of device architecture.[^3a^, ^8^] To obtain efficient CP‐OLEDs, the emitting materials should exhibit large luminescence dissymmetry factors (*g*_lum_) and high luminescence quantum yields, the preconditions being difficult to meet in a single molecule. Since chiral organic molecules commonly suffer from relatively low *g*_lum_, the development of new CPL materials and the elucidation of design principles to enhance the key parameters are of utmost importance.[^4b^, ^9^] In general, there are different strategies to alter the electronic and photophysical properties of π‐conjugated scaffolds. For example, increasing the polyaromatic system by further addition of benzene units, leads to high delocalization of electron density, resulting in remarkable optical and electronic features, as shown for planar compounds,^[10]^ although high distortion of the π‐system in a helical framework tends to reduce the effective conjugation length. On the other hand, it evades some luminescence quenching processes typical of planar, strongly aggregating PAHs.

Moreover, theoretical investigations revealed that the dissymmetry factors of carbo[*n*]helicenes could be influenced by the number of fused rings forming the helix.^[13]^ Pieters, Müllen and Narita recently reported a significant boost in CPL performance of a π‐extended carbohelicene from 7.7×10^−4^ up to 7.44×10^−3^ by elongation of the helix from *n* of 7 to 9.^[14]^ These compounds outperform pristine carbo[7]‐ and ‐[9]helicenes profiting from the laterally extended π‐system.

**Figure 1:**
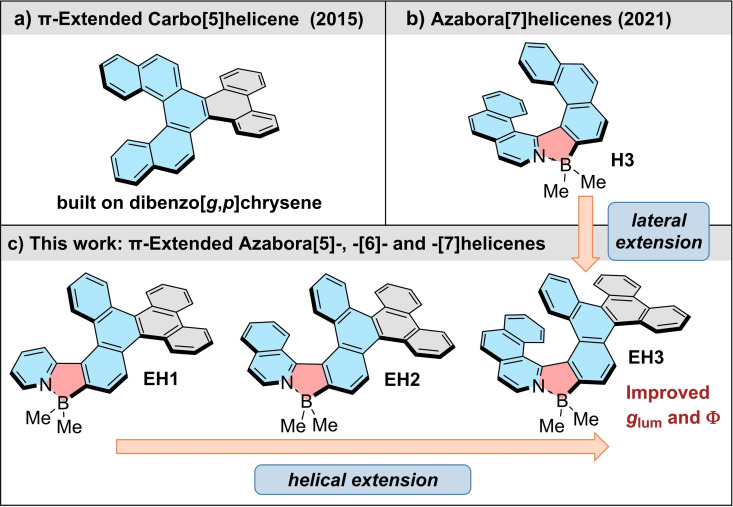
Structures of a) π‐extended carbo[5]helicene containing a dibenzo[*g*,*p*]chrysene unit,^[11]^ b) recently published azabora[7]helicene **H3**,^[12]^ and c) π‐extended azabora[5], ‐[6]‐ and ‐[7]helicenes **EH1**‐**EH3** reported in this work. For simplicity, only (*P*)‐stereoisomers are shown.

Another opportunity to fine‐tune PAHs is offered by doping π‐conjugated frameworks with heteroatoms,^[15]^ a highly appealing choice being the incorporation of boron. Its interactions through its vacant p_z_ orbital with the surrounding π‐donating system ensure enhanced electron delocalization within the conjugated scaffold.^[16]^ The additional embedding of nitrogen into the boron‐doped π‐system reinforces these effects providing materials with high electron affinity and mobility^[17]^ and leads to compounds with strong emission and large two‐photon absorption cross‐sections.^[18]^ Merging the structural motifs of distorted azabora‐π‐extended systems with helical chirality can provide materials with outstanding photophysical and chiroptical characteristics. Recently, we showed that such systems (e.g. **H3**, Figure 1b) can combine high fluoresecence quantum yields in both solution and solid state with large absorption dissymmetry factors.^[12]^ Nonetheless, only a handful of configurationally stable azaborole helicenes has been reported to date.^[19]^ Thus, their potential has yet to come to fruition. These compounds, as well as other boron‐containing helicenes, are typically less accessible due to the inherent strain and the requirement of highly congested intermediates that impede their synthesis. The successful actualizations include azabora[6]helicenes,^[20]^ and double helicenes^[21]^ incorporating either dihydro‐1,2‐ or ‐1,4‐azaborinine or 1,2‐oxaborinine six‐membered rings in the molecular scaffolds.

In this study, we report the synthesis and properties of π‐extended azabora[*n*]helicenes containing a five‐membered azaborole ring (Figure 1c). This unique set of three B,N‐helicenes with a gradually increasing helix length from five to seven angularly fused rings was prepared by our modular approach from *N*‐heterocycles and a dibenzo[*g,p*]chrysene (**DBzC**) building block. To access the latter coupling partner bearing one functional group at a sterically hindered position, we developed an effective synthetic route. Dibenzo[*g,p*]chrysenes exhibit appreciable emission, charge transport and constitute supramolecular building blocks to form, for example, liquid crystals but were rarely incorporated into the helicene structure.^[22]^ In 2015, Kamikawa^[11]^ described the synthesis of π‐extended carbo[5]helicene with the outer helicene rings fused to the two benzene rings of **DBzC** (Figure 1a). Our molecular design is based on the elongation of a helical system starting from a single ring of **DBzC** giving rise to the low‐symmetry molecules. The longest homologue can be formally considered as previously reported pristine azaborole helicene **H3** (colored in blue) fused with a phenanthrene unit (colored in grey). Our studies have revealed that such an enlargement of π‐conjugated system leads to a strong enhancement of the emission quantum yields and luminescence dissymmetry factors. In addition, we have analyzed the effect of both lateral and helical extension on electrochemical properties. These studies are described together with insights into their complex enantiomerization processes that involve conformational changes of the **DBzC** moiety. The longer homologues, azabora[6]‐ and azabora[7]helicenes represent the first examples of configurationally stable π‐extended azaborole helicenes. Their helical structures were unambiguously confirmed by the single crystal X‐ray analysis.

## Results and Discussion

**Scheme 1:**
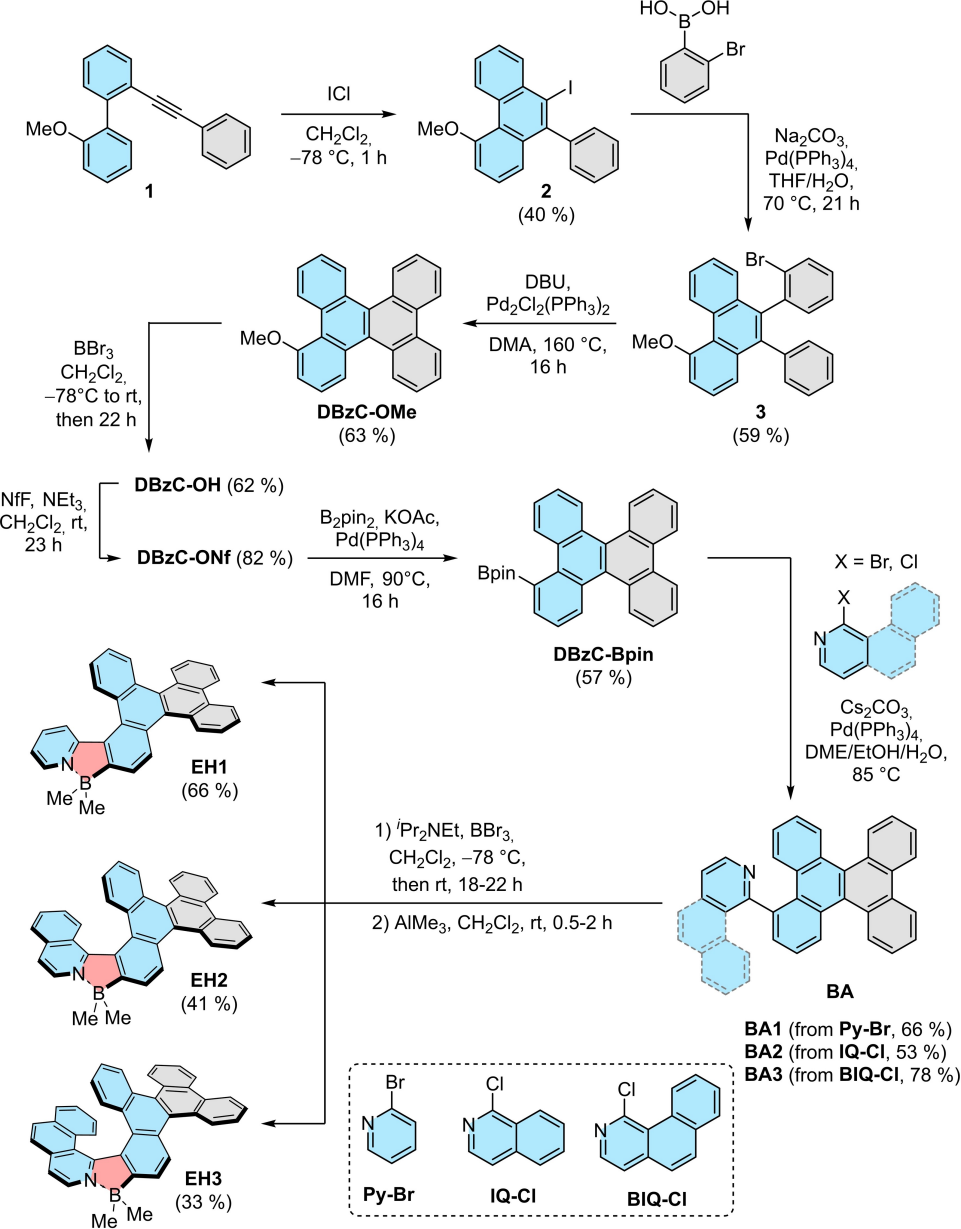
Synthesis of **EH1**‐**EH3**.

**Synthesis**: Target molecules **EH1‐EH3** were built on the basis of a dibenzo[*g,p*]chrysene scaffold and different nitrogen‐containing heteroarenes (Scheme 1). 2‐Bromopyridine (**Py‐Br**) and 1‐chloroisoquinoline (**IQ‐Cl**) were commercially available, while 1‐chlorobenzo[*h*]isoquinoline (**BIQ‐Cl**) was prepared in five steps according to a synthetic route established in our group.^[12]^ Synthesis of the unreported key intermediate, borylated dibenzo[*g,p*]chrysene **DBzC‐Bpin**, required functionalization of **DBzC** at position 1, a synthetically challenging site to access. Here, we developed an eight‐step synthesis starting from the Sonogashira cross‐coupling reaction of 1‐bromo‐2‐iodobenzene with phenylacetylene. The resulting 1‐bromo‐2‐(phenylethynyl)benzene was converted into biphenylacetylene (**1**) via Suzuki coupling with (2‐methoxyphenyl)boronic acid. The subsequent ICl‐induced intramolecular cyclization thereof afforded the corresponding iodophenanthrene **2**. The following cross‐coupling of **2** with 2‐bromophenylboronic acid and Pd‐catalyzed C−H arylation of **3** gave 1‐methoxydibenzo[*g,p*]chrysene (**DBzC‐OMe**). Noteworthy, the presence of bromide in **3** was indispensable, as the attempts to fuse 4‐methoxy‐9,10‐diphenylphenanthrene under various conditions, including photocyclization with I_2_ in the presence of propylene oxide and oxidative aromatic coupling with FeCl_3_/MeNO_2_ or 2,3‐dichloro‐5,6‐dicyanobenzoquinone, were unsuccessful otherwise. Next, ether cleavage with BBr_3_ provided hydroxydibenzo[*g,p*]chrysene **DBzC‐OH**, which was subsequently converted into perfluorobutane‐1‐sulfonate (nonaflate) **DBzC‐ONf**. The synthesis of the desired building block was accomplished by Miyaura borylation to obtain **DBzC‐Bpin** in overall 3.6 % yield. Having all the components in hand, we commenced to assemble the target helicenes according to our modular approach.^[12]^ To this end, we prepared biaryls **BA1**‐**BA3** in yields of 53–78 % by cross‐coupling of **Py‐Br**, **IQ‐Cl** and **BIQ‐Cl** with **DBzC‐Bpin**. The incorporation of boron to form an azaborole ring was executed by nitrogen‐directed intramolecular electrophilic borylation with BBr_3_ in the presence of *i‐*Pr_2_NEt.[^12^, ^23^] The bromide ligands were subsequently exchanged by treatment with the 2.0 m AlMe_3_ solution to receive target π‐extended helicenes **EH1**‐**EH3** in moderate to good yields (33‐66 % over two steps). As opposed to their brominated analogues, these helically chiral compounds exhibit excellent stability against air, moisture and light.

**Figure 2:**
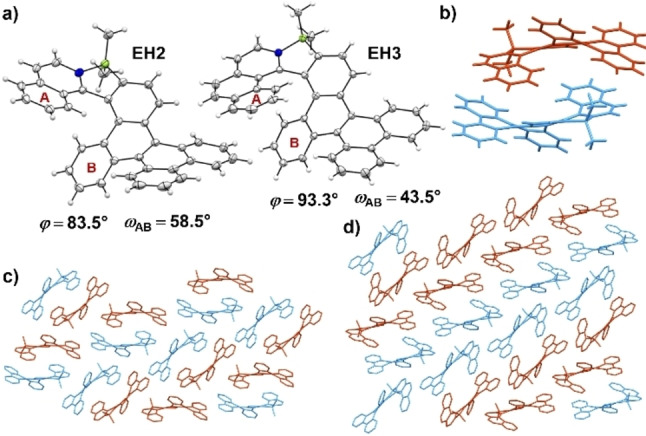
a) Molecular structures of **EH2** and **EH3** determined by X‐ray analysis at 100 K. Only (*P*)‐enantiomers are depicted. ORTEP drawings are shown with 50 % probability. b) An (*M*)‐ and (*P*)‐dimer of **EH2**. Packing arrangements of c) **EH2** and d) **EH3**. Hydrogen atoms are omitted for clarity. (*P*)‐ and (*M*)‐enantiomers are colored in blue and maroon, respectively.

**Solid state structural analysis**: To gain insight into the structures and packing arrangements of these new scaffolds, we grew single crystals of the racemic samples of **EH2** and **EH3** by slow diffusion of hexane into their CH_2_Cl_2_ solutions (Figure 2a,b and Supporting Information). Both compounds were highly soluble in CH_2_Cl_2_. On the other hand, **EH1** showed significantly lower solubility than its longer homologues, presumably due to the less distorted structure and stronger tendency to form aggregates. All the attempts to grow crystals of **EH1** led only to amorphous powders, unsuitable for single crystal X‐ray analysis. The racemates of **EH2** and **EH3** crystallized in the space group *P*2_1_ */n* and adopt the most stable helical conformations **C1** (cf. Figure 5). The B−N bond lengths were measured as 1.622(2) Å in both helicenes indicating strong Lewis pair interactions and stable azaborole rings. The sums of all dihedral angles of the inner helicene rims (*φ*) are 83.5° and 99.3° (comparable to the corresponding angles in the optimized geometries, i.e. 79.2° and 104.5°), while the dihedral angles (*ω*_AB_) between the mean planes of the terminal rings are 58.0° and 43.5° for **EH2** and **EH3**, respectively. The splay angle of **EH2** is similar to that of pristine carbo[6]helicene (58.5°),^[2a]^ whereas the value for **EH3** is significantly higher when compared to all‐carbon [7]helicene (32.4°).^[24]^ The smaller angle for **EH3** compared to its shorter homologue results from the more pronounced intramolecular π‐π interactions between the stronger overlapping terminal rings. This is in agreement with the trend observed for extended helicenes which attain more compact structures upon elongation of the helix.^[2a]^ Direct comparison of pristine azabora[7]helicene **H3** (splay angle of 28.1°) and its laterally extended congener unfolds a difference of more than 15°, which can be ascribed to the strong distortion of the helicene backbone of **EH3**, enlarged by the repulsion from the fused phenanthrene moiety. Apparently, the twisted helicene structures in combination with the distorted arrangement of the dibenzo[*g,p*]chrysene subunit reduces distinct π‐π interactions. As opposed to the pristine azabora[7]helicene, molecules of **EH3** do not form the characteristic offset face‐to‐face *P*‐*M* stacks. Its packing arrangement (Figure 2d) is governed to a large extent by multiple C−H⋅⋅⋅π interactions, predominantly between the face‐to‐edge aligned molecules of the same chirality, while very weak π‐π interactions can be observed between the homochiral parallel molecules forming a stack with a huge offset. The intramolecular π‐π interactions are also reduced in the crystal of **EH2** (Figure 2c). Similar to **EH3**, the packing arrangement of **EH2** is stabilized by C−H⋅⋅⋅π interactions between the neighboring molecules of the same or opposite chirality. However, it also shows substantial differences. When compared to **EH3**, the (*P*)‐ and (*M*)‐enantiomers of **EH2** assemble in a more distinctive stack (Figure 2b) in a slipped fashion with a significant offset. The two vicinal molecules of a stack are oriented antiparallel so that the **IQ** and outer dibenzochrysene rings of one molecule interact with the **DBzC** and **IQ** rings of the second enantiomer. It appears that despite the extension of the helicene skeletons by a phenanthrene subunit, the interactions between the molecules are quite weak. Such behavior may improve processability of these PAHs.

**Figure 3:**
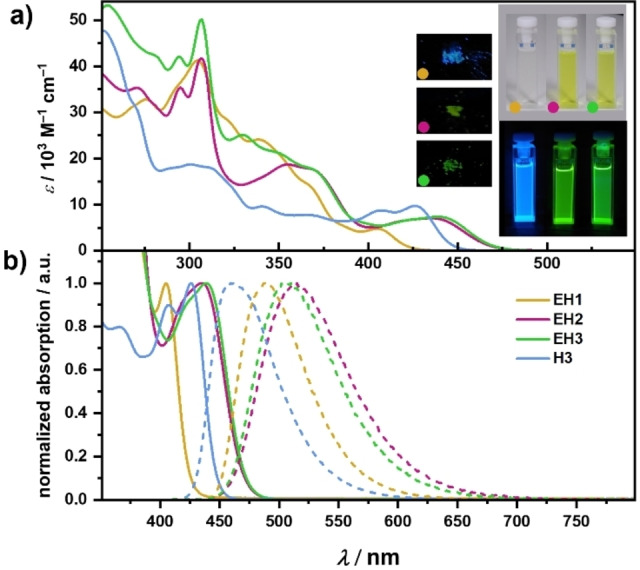
a) Absorption spectra of **EH1‐EH3** (*c*=4.7×10^−5^–8.7×10^−5^ m) and **H1** in CH_2_Cl_2_. Inset right: Photographs of the solutions of **EH1‐EH3** under visible light (top) and UV light (bottom). Inset left: Photographs of the powders of **EH1‐EH3**. b) Normalized absorption (solid lines) and emission (dashed lines) spectra in CH_2_Cl_2_ (**EH1**: *λ*_ex_=375 nm, **EH2** and **EH3**: *λ*_ex_=400 nm; *c*=6.2×10^−6^–1.1×10^−5^ m).

**Absorption and emission properties**: The lowest‐energy absorption bands of **EH1** and **EH2** are centered at 435 and 439 nm (Figure 3a), respectively. While both bands nearly overlap, the absorption maximum of **EH1** is blue‐shifted to 405 nm. This shift is accompanied by a change in the DCM solution colors from intense yellow for **EH2** and **EH3** to pale yellow for **EH1**. The S_0_→S_1_ transitions of **EH2** and **EH3** are associated with moderate molar absorption coefficients (*ϵ*) (7.06–7.37 10^3^×m ^−1^cm^−1^) (see Table 1). A somewhat lower *ϵ* value (4.79 10^3^×m ^−1^cm^−1^) was recorded for **EH1**. According to time‐dependent DFT (TD‐DFT) calculations at the CAM−B3LYP^[25]^‐D3(BJ)^[26]^/def2‐TZVP^[27]^ level (solvent CH_2_Cl_2_, PCM model) the lowest‐energy band corresponds predominantly to the HOMO→LUMO transition (74–79 %), where the HOMOs are largely distributed on the dibenzo[*g,p*]chrysene moiety, while the LUMOs are confined to azaborole helicene subunits (Figures S59–S62, Supporting Information) implying charge‐transfer character. A direct comparison of a phenanthrene‐fused **EH3** with structurally similar non‐extended **H3** ^[12]^ reveals lateral extension has a significant effect on the optical properties of azaborole helicenes. In contrast to pristine azaborahelicene **H3**, the bands of **EH1**‐**EH3** are nearly devoid of vibronic fine structures. The lowest‐energy band of **EH3** is bathochromically shifted by ca. 700 cm^−1^. The compounds show blue or green fluorescence with maxima at 448–515 nm (Figure 3b). More specifically, the lateral extension of the azabora[7]helicene shifted the emission maximum by 2180 cm^−1^, which entailed the change in emission color from blue for **H3** to green for **EH3**. Thus, the bathochromic shift is even stronger in the case of emission, which translates to large Stokes shifts in the range from 2370 to 3570 cm^−1^.

**Table 1:** Absorption and emission properties of **EH1‐EH3**.

Cpd	*λ*_abs_^[a]^ [nm]	*ϵ*_max_^[b]^ [10^3^ m ^−1^ cm^−1^]	*λ*_em_^[c]^ [nm]	Stokes Shift [cm^−1^]	Φ^[d]^	*|g*_abs_|^[e]^ [10^−3^]	*|g*_lum_|^[f]^ [10^−3^]	*|g*_abs,calc_|^[g,h]^ [10^−3^]	*|m*|^[g,i]^ [10^−20^ erg G^−1^]	*|μ*|^[g,j]^ [10^−20^ esu cm]	*θ*_*μ,m*_^[g,k]^ [deg]	*|g*_lum,calc_|^[g,l]^ [10^−3^]
**EH1**	405	4.79	448	2370	0.18	–	–	–	–	–	–	–
**EH2**	435	7.06	515	3570	0.28	2.1 (341)	1.6	1.34	0.59	423	104.5	1.39
						1.6 (437)						
**EH3**	439	7.37	510	3170	0.31	3.3 (373)	2.2	1.79	1.38	373	96.7	1.73
						3.2 (358)						
						2.2 (443)						

[a] Absorption maximum in CH_2_Cl_2_. [b] Molar absorption coefficient. [c] Fluorescence maximum in CH_2_Cl_2_ solution. [d] Relative fluorescence quantum yield determined by the optical dilution method. [e] Absorption dissymmetry factor at a given wavelength is parentheses. [f] Luminescence dissymmetry factor. [g] Calculated at the M06‐2X/def2‐SVP level in the gas phase using the geometry optimized in the S_1_ state. [h] Calculated absorption dissymmetry factor for the S_0_→S_1_ transition. [i] Transition magnetic dipole moment for the S_1_→S_0_ transition. [j] Transition electric dipole moment for the S_1_→S_0_ transition. [k] Angle between *μ* and *m* for the S_1_→S_0_ transition. [l] Calculated luminescence dissymmetry factor.

Notably, the Stokes shift of **EH3** is nearly two‐fold larger than that of the non‐extended counterpart, presumably due to the more extensive structural relaxation of the molecular framework upon photoexcitation associated with the dibenzo[*g,p*]chrysene moiety. Importantly, the beneficial effect of π‐extension manifests itself in emission intensity. Building the helical structure on a dibenzochrysene moiety enhanced the quantum yield by ca. 50 % in comparison to the parent helicene (cf. Table 1). Accordingly, **EH3** achieved Φ in CH_2_Cl_2_ as high as 0.31. Likewise, the one‐ring shorter homologue also exhibits appreciable emission with a Φ of 0.28. These values position **EH3** and **EH2** among the more efficient configurationally stable single helicene emitters containing five‐membered rings.^[28]^ On the other hand, Φ recorded for **EH1** (0.18) was significantly lower than that of **EH2**, although still comparable to the emission intensity of **H3**.^[12]^ To contrast these B−N helically chiral compounds with all‐carbon non‐extended derivatives, it should be pointed out that a gradual elongation of carbohelicene with benzene rings leads to a drop of Φ from 0.04 for [5]‐ and [6]helicenes to merely 0.02 for [7]helicene.^[29]^ Despite the quite weak interactions in the solid state, defined by the prevalent C−H⋅⋅⋅π interactions, the fluorescence intensity of **EH2** and **EH3** is substantially lower than in solution. Φ determined for the powder samples of **EH3** and **EH2** is reduced to 0.13 and 0.08, respectively. Surprisingly, emission of the smallest system **EH1** was essentially retained upon going from solution to the solid state (0.18 in the CH_2_Cl_2_ solution vs. 0.16 in the solid state), even though this compound shows stronger tendency to form stacks than its longer homologues, as deduced based on its poor solubility in chlorinated solvents. A distinctive feature of all three compounds are small differences between the positions of the emission maxima for the solution and solid samples (Figure S51, Supporting Information), in contrary to the spectral shift of nearly 2000 cm^−1^ for non‐extended azaborole helicene **H3**. This implies a different arrangement of molecules when compared to **H3** and hence, different decay pathways involved in the de‐excitation of the extended helicenes.

**Electrochemistry**: The electrochemical properties of **EH1**‐**EH3** were investigated by cyclic voltammetry (CV) and differential pulse voltammetry (DPV) in CH_2_Cl_2_ in the presence of Bu_4_NPF_6_ as a supporting electrolyte and calibrated against ferrocenium/ferrocene (Fc^+^/Fc). The voltammograms are shown in Figure S52, while the redox potentials are summarized in Table S7 in the Supporting Information. All compounds undergo two oxidation processes, the first one being irreversible and the second one reversible. This differs from the electrochemical data for our non‐extended azabora[7]helicene **H3**, for which only one irreversible oxidation process could be recorded.^[12]^ The oxidation potentials fall in a similar range, i.e. +0.72‐+0.76 and +0.97‐+0.98 V (DPV) for the first and the second oxidation events indicating a negligible effect of the helical extension on oxidation. The effect is more apparent for the lateral extension, as the *E* ^ox1^ value of **H3** is shifted anodically by ca. 0.2 V, when compared with **EH3**. On the contrary, the impact of the helix elongation is evident for the reduction processes. While **EH2** and **EH3** exhibit similar reduction potentials (−2.07 and −2.12 V, respectively), the potential of **EH1** is cathodically shifted by ca. 0.3 V, which corresponds to the increase of the LUMO energy level and gives rise to the larger band gap for **EH1** (3.17 eV) than for the longer homologues (ca. 2.80 eV). This is consistent with the difference in the positions of the lowest‐energy absorption bands of these compounds. On the other hand, addition of the phenanthrene unit did not affect the first reduction potential of the azabora[7]helicene, the *E* ^red1^ values of **H3** and **EH3** being nearly equal. As opposed to parent **H3**, though, two reduction processes were observed for the extended congener, where the second one at −2.34 V is irreversible. Thus, the effect of lateral extension is more pronounced for oxidation, which is strongly linked to the manipulation of the more electron‐rich moiety (phenanthrene vs. dibenzo[*g,p*]chrysene), while the effect of helical extension manifests itself through changes in the reduction processes, being a consequence of the enlargement of the more electron‐poor helical moiety (**Py**, **IQ**, **BIQ**).

**Figure 4:**
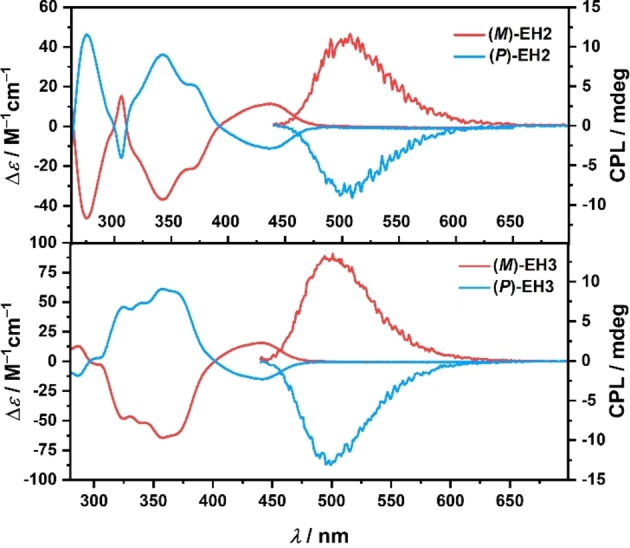
ECD (**EH2**: *c*=3.7×10^−5^ m; **EH3**: *c*=2.7×10^−5^ m) and CPL (*c*=9.0×10^−6^ m, *λ*_ex_=360 nm) spectra of **EH2** and **EH3** in CH_2_Cl_2_.

**Chiroptical properties**: Configurationally stable helicenes **EH2** and **EH3** were resolved via HPLC on a chiral stationary phase (for details see Supporting Information). While **EH2** showed partial, though rather minor, racemization through evaporation on a rotary evaporator with a water bath temperature of 40 °C, the isolated enantiomers of **EH3** were stable under these conditions. This indicates significantly lower racemization barrier of the hexahelicene and can be related to the smaller overlap of the outer rings, as observed in the solid‐state structures of **EH2** and **EH3**. Nonetheless, no indication of racemization of the **EH2** enantiomers was observed at room temperature, which permits the full characterization of their chiroptical properties. The corresponding electronic circular dichroism (ECD) and circularly polarized luminescence (CPL) spectra were recorded in CH_2_Cl_2_ (Figure 4). The absolute configuration was assigned by comparison of the experimental with the computed ECD spectra at the CAM−B3LYP‐D3(BJ)/def2‐TZVP level (solvent CH_2_Cl_2_, PCM model). Accordingly, the first and second fractions of **EH2** and **EH3** were assigned to the (*M*)‐ and (*P*)‐enantiomers, respectively (cf. Figures S63 and S64, Supporting Information). Comparison of the ECD spectra of **EH2**, **EH3** (Figure 4) and **H3** reveals that both helical and lateral extensions affect their profile and intensity. (*P*)‐**EH2** exhibits negative Cotton effects (CEs) in the ranges of 392–ca. 500 nm (Δϵ=−11 m ^−1^ cm^−1^ at 437 nm) and 300–311 nm (Δϵ=−16 m ^−1^ cm^−1^ at 306 nm), while positive CEs can be observed at 311–392 nm (Δϵ=+36 m ^−1^ cm^−1^ at 342 nm) and 264–300 nm (Δϵ=+46 m ^−1^ cm^−1^ at 276 nm). Likewise, the lowest‐energy CD band of (*P*)‐**EH3** between 402 and ca. 495 nm corresponds to the negative CE (Δϵ=−15 m ^−1^ cm^−1^ at 443 nm). The spectrum of the (*P*)‐enantiomer of **EH3** also shows negative CEs below 297 nm (Δϵ=−12 m ^−1^ cm^−1^ at 287 nm), and a broad band in the 298–401 nm range with a positive CE (Δϵ=+61 m ^−1^ cm^−1^ at 358 nm). The spectra of (*M*)‐ and (*P*)‐enantiomers of both compounds reveal a mirror image relationship. While the higher‐energy CD bands of the π‐extended helicenes are considerably weaker than that of pristine **H3**, the intensities of the lowest‐energy bands are somewhat stronger and correspond to the absorption dissymmetry factors (*g*_abs_) of 1.6×10^−3^ (at 437 nm) and 2.2×10^−3^ (at 443 nm) for **EH2** and **EH3**, respectively, being ca. two‐three times larger than that of **H3** (0.8×10^−3^ at 426 nm).^[12]^ The highest |*g*_abs_| values are observed at 341 nm (2.1×10^−3^) for **EH2** and at 373 nm (3.3×10^−3^) for **EH3**.

In addition to being appreciable emitters, these compounds are CPL active. The mirror‐image CPL spectra of their (*P*)‐ and (*M*)‐enantiomers in CH_2_Cl_2_ are shown in Figure 4. The (*M*)‐enantiomer of **EH2** reveals the emission maximum at a wavelength of 507 nm, while the maximum of (*M*)‐**EH3** is positioned at 501 nm. These positive bands correspond to the |*g*_lum_| anisotropy factors of 1.6×10^−3^ and 2.2×10^−3^ for **EH2** and **EH3**, respectively.

To unravel the effect of helical extension on the chiroptical properties of **EH2** and **EH3**, we performed DFT calculations on the excited state using several combinations of basis sets and functionals. The calculations at the M06‐2X^[30]^/def2‐SVP^[27]^ level satisfactorily reproduced the signs and trend in the *g*_lum_ values observed experimentally, although the increase upon going from **EH2** to **EH3** is less distinctive. The computed values of *g*_lum_, *m* and *μ* are summarized in Table 1.

The *g*_lum_ (or *g*_abs_) value can be calculated according to equation 1:

(1) $$g_{lum}=\frac{{4\left|\mu \right|\left|m\right|\cos\theta }_{\mu ,m}}{{\left|\mu \right|}^{2}+{\left|m\right|}^{2}}$$

and depends on the magnitudes of the transition electric (*μ*) and magnetic (*m*) dipole moments for the first excited state in addition to angle *θ*_μ,m_ between these two vectors. Thus, to maximize the *g*_lum_ value, the vectors should be oriented parallel or antiparallel to one another and *m*, typically small for organic molecules, should be increased relative to *μ*. Our computations indicate that the enhanced *g*_lum_ value of **EH3** is likely attributed to the almost 2.5‐fold larger *m* for this compound compared to the azabora[6]helicene. This increase overcompensates for a less favorable angle between *μ* and *m* (96.7° for **EH3** vs. 104.5° for **EH2**) and hence, a smaller absolute cos*θ*_μ,m_ value for **EH3** (Figure 6b). Interestingly, the calculated *g*_abs_ values for the S_0_→S_1_ transitions (Table 1 and S13) are comparable to the *g*_lum_ values, which is also observed for the experimentally determined dissymmetry factors.

**Figure 5:**
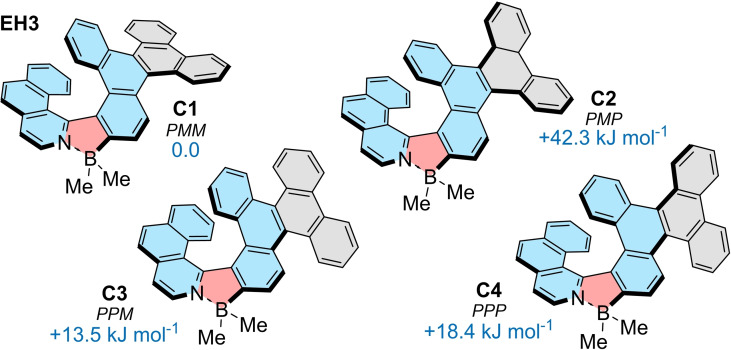
Conformations **C1**‐**C4** of **EH3** and their relative Gibbs free energies in kJ mol^−1^ (B3LYP‐D3(BJ)/def2‐SVP, solvent CH_2_Cl_2_, PCM model).

**Figure 6:**
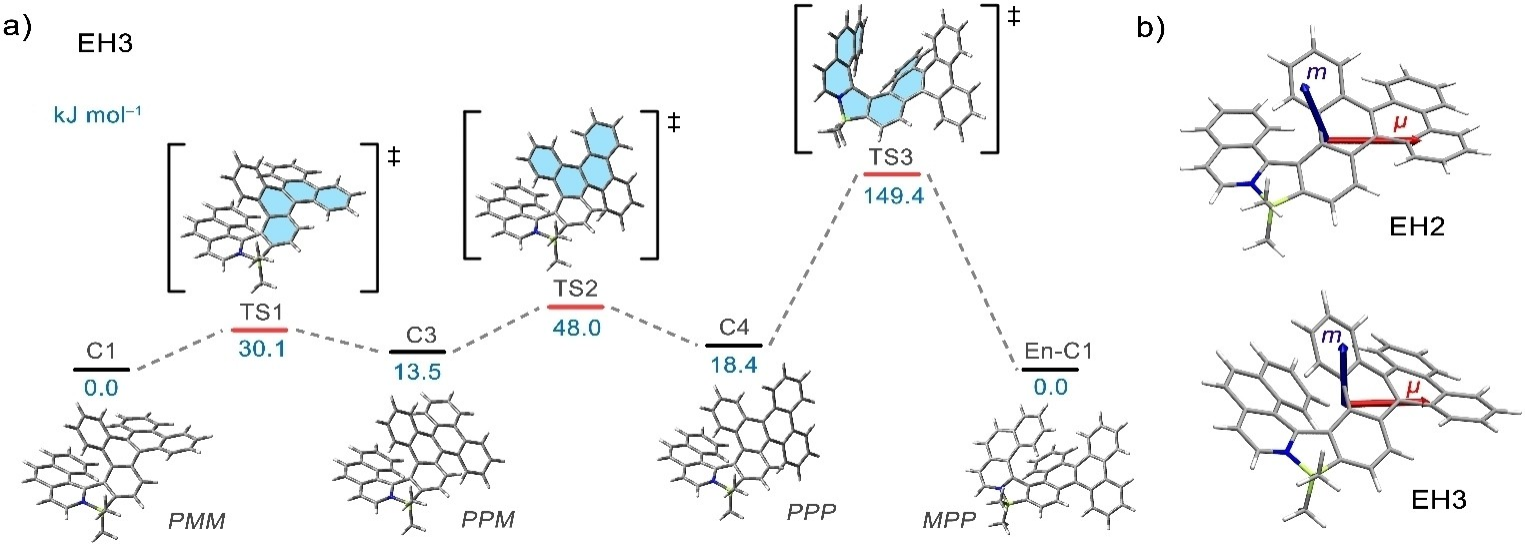
a) The most plausible interconversion pathway of **EH3** calculated at the B3LYP‐D3(BJ)/def2‐SVP (solvent CH_2_Cl_2_, PCM model) level. The relative Gibbs free energies of the stationary points are given in kJ mol^−1^. b) DFT‐optimized structures of **EH2** and **EH3** and the spatial arrangement of the electric (*μ*, red) and magnetic (*m*, blue) dipole moments in the S_1_ excited state at the TD‐M06‐2X/def2‐SVP level.

To our delight, the lateral extension of the azabora[7]helicene not only increased the quantum yield, but it also boosted the preferential emission of CPL. Accordingly, the |*g*_lum_| value of **EH3** is more than doubled when compared to its non‐extended congener **H3** (1.0×10^−3^). Notably, both |*g*_lum_| values are also significantly higher than those for azabora[6]‐, azabora[8]helicenes with one boron atom, as well as for azabora[10]helicene possessing two boron centers.^[19a]^ In addition, |*g*_lum_| of **EH3** surpasses the dissymmetry factors of an all‐carbon π‐extended [7]superhelicene^[31]^ or a carbo[7]helicene constructed on tribenzo[*fg*,*ij*,*rst*]pentaphene,^[14]^ azahelicene‐fused BODIPY,^[28b]^ and the recently reported B,N‐embedded double [7]helicenes.^[21c]^ These results validate the lateral extension as a suitable method to enhance the optical and chiroptical properties of azabora[*n*]helicenes.

***P***‐***M*** **interconversion**: In contrast to **EH1**, (*P*)‐ and (*M*)‐enantiomers of **EH2** and **EH3** are configurationally stable at room temperature. All three derivatives consist formally of one azabora[*n*]helicene and two carbo[4]helicene subunits of the dibenzo[*g,p*]chrysene moiety and, principally, can adopt four conformations in solution (Figure 5). DFT calculations (B3LYP‐D3(BJ)/def2‐SVP, CH_2_Cl_2_, PCM solvent model) revealed that the **C1** conformations with the azaborahelicene of one helicity and carbohelicene of the opposite helicity represent the most stable conformations for all three derivatives. **C3** and homochiral **C4** of **EH3** are ca. 14 and 18 kJ⋅mol^−1^ higher in energy, while **C2** are the most destabilized. The differences in energies are similar for conformations of **EH1** and **EH2** (Figure S58 and Table S9). Based on the Boltzmann distribution, the presence of **C2**‐**C4** is negligible. The second most abundant conformation, **C3**, would be adopted by less than 0.5 % of the overall population of molecules in solution at room temperature, while the energetically disfavored **C2** can be completely neglected. Nonetheless, these conformations have to be considered in the analysis of the *P*‐*M* inversion process, as they occur as stationary points alongside the transition states on the interconversion pathways from global minimum **C1** to its enantiomer **En‐C1**. The interconversions of **EH1**‐**EH3** were studied at B3LYP‐D3(BJ)/def2‐SVP (CH_2_Cl_2_, PCM solvent model) level of theory. Evaluation of various pathways indicates the most accessible scenario involves three transition states (Figure 6 and S65–S69). Accordingly, the most feasible inversion of the [7]helicene was found for the conversion of **C4** to **En‐C1** (149.4 kJ⋅mol^−1^). The two preceding steps involve the inversion of two carbohelicene subunits and are thus associated with a small energy penalty (30.1 and 48.0 kJ⋅mol^−1^ vs. global minimum from **C1** to **C3** and **C3** to **C4**, respectively). The inversion of **EH1** and **EH2** follows similar pathways (for details, see Supporting Information). The rate‐determining step is the inversion of the azaborahelicene in each case. While **EH2** exhibits sufficient resistance to racemization so as to resolve its enantiomers (Δ*G* ^≠^=112.4 kJ⋅mol^−1^), **EH1** interconverts rapidly at room temperature in accordance with its small calculated energy barrier (Δ*G* ^≠^=63.1 kJ⋅mol^−1^). The inversion barriers of the configurationally stable systems are consistent with the experimental results. The thermal racemization of (*M*)‐**EH2** (at 65 °C) and (*M*)‐**EH3** (at 170 °C) in 1,2‐dichlorobenzene were monitored by analytical HPLC following the decay of the enantiomeric excess to provide a Gibbs activation energy of 103.1 and 138.5 kJ⋅mol^−1^ for **EH2** and **EH3**, respectively (Figures S54 and S55 in the Supporting Information).

Thus, the helical elongation of an azaborole helicene gradually increases the configurational stability from **EH1** to **EH3** in accordance with the increased overlap of the terminal rings. The lateral π‐extension of the helicene backbone slightly lowers Δ*G* ^≠^ of **EH2** by about 11 kJ ⋅ mol^−1^ versus pristine azabora[6]helicene **H2** (Δ*G* ^≠^, 123.9 kJ ⋅ mol^−1^), while the barriers of the congeners with an odd number of rings are similar (Δ*G* ^≠^ of 152.3^[12]^ and 59.4 kcal ⋅ mol^−1^ for the non‐extended [7]‐, and [5]helicenes **H3** and **H1**, Figures S70 and S71, Supporting Information). On the other hand, fusion of the phenanthrene unit had a positive effect on the inversion barriers of the carbo[4]helicene subunits enhancing its Δ*G* ^≠^ by ca. two‐fold when compared with Δ*G* ^≠^ of the parent molecule (Figure S72, Supporting Information). This amplification is presumably attributed to the fusion mode of the two [4]helicene moieties in dibenzo[*g,p*]chrysene that enhance the repulsion from the neighboring helical systems at the corresponding transition states, as observed before for various double helicenes.^[1c]^

## Conclusion

In this work, we presented a set of azaborahelicenes with lateral π‐extension of the helicene backbones. These compounds differ in the degree of π‐extension along the helical axis. Their synthesis is based on the dibenzo[*g,p*]chrysene intermediates bearing a functional group at a sterically hindered position. The single crystal X‐ray analysis of aza[6]‐ and ‐[7]helicenes unambiguously confirmed the structures of the first configurationally stable π‐extended azaborole helicenes and provided insight into their packing arrangements. Despite the larger scaffolds, the intramolecular interactions, dominated by C−H⋅⋅⋅π interactions, are relatively weak. Fusion of the phenanthrene unit provided materials with bathochromically shifted absorption and emission spectra, large Stokes shifts and high emission quantum yields of up to 0.31 for the longest homologue, thus increasing Φ by ca. 50 % compared to the pristine azabora[7]helicene. Not only did we obtain a stronger emitter through lateral extension, but also enhanced its chiroptical properties to reach a |*g*_lum_| value over two times larger than that of the non‐extended congener. Likewise, helical extension contributed to the increase in Φ and |*g*_lum_| values from 1.6×10^−3^ for **EH2** to 2.2×10^−3^ for **EH3**. According to DFT calculations, this enhancement originates from the increase in magnitude of the magnetic transition dipole moment that overcompensates for the inferior *θ*_μ,m_ angle. The effect of lateral and helical extensions was also evident in the electrochemical properties of the helicenes. While fusion of phenanthrene elevates the HOMO levels, the elongation of the helix decreases the LUMO levels leading to lower band gaps for [6]‐ and [7]helicenes. Our computations revealed the complex interconversion pathways of these π‐extended systems due to the presence of **DBzC** moiety consisting of two carbo[4]helicenes. The rate‐determining step is the inversion of an azaborole helicene in each case. While azaborole[5]helicene interconverts rapidly at room temperature, the longer homologues are configurationally stable. In particular, the azabora[7]helicene exhibits high resistance to racemization showing potential for the application as a CPL emitter in optoelectronic devices. In conclusion, we demonstrated that both helical and lateral extensions are viable approaches to enhance the photophysical properties of azaborole helicenes. The molecules in this study may serve as model compounds for the rational optimization of luminophores through incorporation of functional groups at strategic positions of helical frameworks. These studies are ongoing in our laboratory.

## Experimental Section

**Materials**: All reagents were purchased from commercial sources and used as received without further purification, unless otherwise stated. Reagent grade solvents were distilled prior to use. Column chromatography was performed on silica (silica gel, 230–400 mesh)

**Methods**: ^1^H‐, ^11^B‐, ^13^C‐ and ^19^F NMR spectra were recorded on a Bruker Avance 400 or an Avance III HD 400 spectrometers and were calibrated to the residual solvent signals. *J* values are given in Hz. The following abbreviations were used to designate multiplicities: s=singlet, d=doublet, t=triplet, dd=doublet of doublets, ddd=doublet of doublet of doublets, dm=doublet of multiplets, dt=doublet of triplets, td=triplet of doublets, m=multiplet, br=broad.

High‐resolution mass spectra were obtained by electrospray ionization (ESI) or atmospheric‐pressure chemical ionization (APCI). ESI and APCI spectra were recorded on an ESI micrOTOF Focus spectrometer from Bruker Daltonics.

UV/Vis spectra were recorded on a Jasco V‐770 UV/Vis spectrometer. All spectroscopy measurements were conducted with spectroscopic grade solvents from ACROS Organics. Conventional quartz cells (light path 1 cm) were used.

Emission spectra were recorded using a FLS 980 fluorescence spectrometer from Edinburgh Instruments equipped with a double monochromator for emission and excitation. The spectra were corrected against photomultiplier and lamp intensity. The fluorescence quantum yields were determined by the optical dilution method (OD≤0.05)^[32]^ as the average value of six different excitation wavelengths with perylene (Φ_fl_=0.94 in cyclohexane)^[33]^ as a standard.

Absolute fluorescence quantum yields of powders were determined on a Hamamatsu Absolute PL Quantum Yield Measurement System CC9920‐02. The system is composed of a 150 W CW Xenon lamp as the excitation source, a monochromator (250–700 nm, full width at half‐maximum (FWHM) 10 nm), an integrating sphere, and a multichannel spectrometer capable of simultaneously measuring multiple wavelengths between 300 and 950 nm.

CD and CPL spectra were recorded on a customized Jasco CPL‐300/J‐1500 hybrid spectrometer.

Cyclic voltammetry (CV) and differential pulse voltammetry (DPV) measurements were performed on a standard, commercial electrochemical analyzer (EC epsilon; BAS Instruments, UK) in a three electrode single compartment cell under an argon atmosphere. The supporting electrolyte NBu_4_PF_6_ was synthesized according to the literature,^[34]^ recrystallized from ethanol/water, and dried in a high vacuum. The measurements were carried out in CH_2_Cl_2_/0.1 m NBu_4_PF_6_ under the exclusion of air and moisture at a concentration of *c* ∼2.5–2.8×10^−4^ m with the ferrocenium/ferrocene redox couple as an internal standard for the calibration of the potential. Working electrode: glassy carbon (Ø 1 mm); reference electrode: Ag/AgCl; auxiliary electrode: Pt wire. The internal resistance was compensated by 50 %.

**Synthesis of 9‐iodo‐4‐methoxy‐10‐phenylphenanthrene (2): 1** (908 mg, 3.19 mmol, 1.0 eq.) was dissolved in CH_2_Cl_2_ (40 mL). The resulting solution was cooled to −78 °C under nitrogen atmosphere. Afterwards, iodine monochloride (1.0 m in CH_2_Cl_2_; 6.39 mL, 6.39 mmol, 2.0 eq.) was added dropwise via syringe and it was stirred at −78 °C for 1 h. Then the mixture was warmed to rt, aq. sat. Na_2_S_2_O_3_ (50 mL) was added and the aqueous phase was extracted with CH_2_Cl_2_ (4×40 mL). The combined organic phases were dried over MgSO_4_, filtered and concentrated *in vacuo*. Subsequently, the crude product was purified by column chromatography (SiO_2_, Hex/EtOAc, 49 : 1) to yield **2** (531 mg, 1.29 mmol, 40 %) as a yellow solid. m.p.: 144–147 °C. ^1^H NMR (400 MHz, CD_2_Cl_2_) *δ*=9.77–9.72 (m, 1H, Ar−H), 8.56–8.49 (m, 1H, Ar−H), 7.74–7.64 (m, 2H, Ar−H), 7.59–7.49 (m, 3H, Ar−H), 7.36 (t, *J*=8.0 Hz, 1H, Ar−H), 7.30–7.24 (m, 2H, Ar−H), 7.22 (dd, *J*=8.0, 0.9 Hz, 1H, Ar−H), 7.03 (dd, *J*=8.2, 1.2 Hz, 1H, Ar−H), 4.15 (s, 3H, OCH_3_) ppm. ^13^C NMR {^1^H} (100 MHz, CD_2_Cl_2_) *δ*=158.8, 146.8, 145.7, 135.0, 134.6, 132.9, 131.0, 130.3, 129.0, 128.8, 128.1, 127.8, 127.6, 127.2, 122.0, 121.2, 109.6, 108.5, 56.3 ppm. APCI‐DIP‐HRMS: m/z calcd for C_21_H_16_IO [M+H]^+^ 411.0240, found 411.0265.

**Synthesis of 9‐(2‐bromophenyl)‐4‐methoxy‐10‐phenylphenanthrene (3): 2** (975 mg, 2.38 mmol, 1.0 eq.), (2‐bromophenyl)boronic acid (620 mg, 3.09 mmol, 1.3 eq.), Na_2_CO_3_ (1.01 g, 9.51 mmol, 4.0 eq.) and Pd(PPh_3_)_4_ (275 mg, 238 μmol, 0.1 eq.) were dissolved in a degassed mixture of THF/H_2_O (80 mL, 3 : 2). The resulting mixture was heated to 70 °C and stirred for 21 h under nitrogen atmosphere. Afterwards, sat. aq. NaHCO_3_ (100 mL) was added and it was extracted with CH_2_Cl_2_ (5×80 mL). The combined organic phases were dried over MgSO_4_, filtered and concentrated *in vacuo*. Subsequently, the crude product was purified by column chromatography (SiO_2_, Hex/CH_2_Cl_2_ 4 : 1) to yield **3** (616 mg, 1.40 mmol, 59 %) as a colorless solid. m.p.: 76–78 °C. ^1^H NMR (400 MHz, CD_2_Cl_2_) *δ*=9.85 (ddd, *J*=8.7, 1.2, 0.6 Hz, 1H, Ar−H), 7.70–7.63 (ddd, *J*=8.4, 6.8, 1.6 Hz, 1H, Ar−H), 7.55 (ddd, *J*=8.0, 1.2, 0.4 Hz, 1H, Ar−H), 7.51–7.46 (ddd, *J*=6.8, 5.6, 1.2 Hz, 1H, Ar−H), 7.44 (t, *J*=8.1 Hz, 1H, Ar−H), 7.40–7.36 (m, 1H, Ar−H), 7.34 (ddd, *J*=8.2, 1.5, 0.5 Hz, 1H, Ar−H), 7.32–7.09 (m, 9H, Ar−H), 4.19 (s, 3H, OCH_3_) ppm. ^13^C NMR {^1^H} (100 MHz, CD_2_Cl_2_) *δ*=159.2, 141.2, 140.3, 137.5, 137.2, 134.8, 133.3, 132.5, 131.6, 131.2, 130.3, 129.7, 129.1, 129.0, 128.2, 127.8, 127.3, 127.1, 126.84, 126.82, 126.6, 126.5, 125.3, 121.2, 121.1, 109.1, 56.3 ppm. ESI‐HRMS: m/z calcd for C_27_H_20_BrO [M+H]^+^ 439.0692, found 439.0689.

**Synthesis of 1‐methoxydibenzo[*g,p*]chrysene (DBzC‐OMe): 3** (616 mg, 1.40 mmol, 1.0 eq.) was dissolved in *N,N*‐dimethylacetamide (65 mL) and the resulting mixture was degassed via Ar‐bubbling (10 min). Afterwards, 1,8‐diazabicyclo[5.4.0]undec‐7‐en (419 μL, 427 mg, 2.80 mmol, 2.0 eq.) and PdCl_2_(PPh_3_)_2_ (98.4 mg, 140 μmol, 0.1 eq.) were added under nitrogen atmosphere. The resulting mixture was heated to 160 °C and stirred for 16 h. The mixture was cooled to rt and diluted aq. HCl (100 mL) was added. Afterwards, it was extracted with CH_2_Cl_2_ (5 x 100 mL). The combined organic phases were dried over MgSO_4_, filtered and concentrated *in vacuo*. Subsequently, the crude product was purified by column chromatography (SiO_2_, Hex/CH_2_Cl_2_ 4 : 1) to yield **DBzC‐OMe** (313 mg, 873 μmol, 63 %) as a colorless solid. ^1^H NMR (400 MHz, CDCl_3_) *δ*=9.49‐9.41 (m, 1H, Ar−H), 8.74–8.61 (m, 5H, Ar−H), 8.30 (dd, *J*=8.2, 0.8 Hz, 1H, Ar−H ), 7.71–7.58 (m, 6H, Ar−H), 7.55 (t, *J*=8.1 Hz, 1H, Ar−H), 7.19 (dd, *J*=8.0, 0.6 Hz, 1H, Ar−H), 4.14 (s, 3H, OCH_3_) ppm. ^13^C NMR {^1^H} (100 MHz, CDCl_3_) *δ*=157.9, 131.8, 131.1, 131.0, 130.2, 129.5, 129.4, 129.02, 129.01, 128.9, 128.4, 128.2, 127.6, 127.0, 126.71, 126.67, 126.53, 126.52, 126.4, 125.8, 123.7, 123.6, 121.5, 120.9, 108.8, 56.1 ppm. ESI‐HRMS: m/z calcd for C_27_H_18_O [M]^+^ 358.1352, found 358.1358. m.p.: 104–107 °C.

**Synthesis of dibenzo[*g,p*]chrysen‐1‐ol (DBzC‐OH): DBzC‐OMe** (50.0 mg, 140 μmol, 1.0 eq.) was dissolved in CH_2_Cl_2_ (4 mL) and the mixture was cooled to −78 °C. Afterwards, BBr_3_ (19.9 μmol, 52.4 mg, 209 μmol, 1.5 eq.) was added dropwise under nitrogen atmosphere and the mixture was warmed to rt and stirred for 22 h. Then water (5 mL) was added and it was extracted with CH_2_Cl_2_ (7×10 mL). The combined organic phases were dried over MgSO_4_, filtered and concentrated *in vacuo*. Subsequently, the crude product was purified by column chromatography (SiO_2_, Hex/CH_2_Cl_2_ 3 : 7) to yield **DBzC‐OH** (30.0 mg, 87.1 μmol, 62 %) as a colorless solid. ^1^H NMR (400 MHz, CDCl_3_) *δ*=9.41–9.30 (m, 1H, Ar−H), 8.72–8.61 (m, 5H, Ar−H), 8.28 (dd, *J*=8.4, 1.1 Hz, 1H, Ar−H), 7.71–7.57 (m, 6H, Ar−H), 7.46 (t, *J*=7.9 Hz, 1H, Ar−H), 7.06 (dd, *J*=7.8, 1.0 Hz, 1H, Ar−H), 5.62 (s, 1H, OH) ppm. ^13^C NMR {^1^H} (100 MHz, CDCl_3_) *δ*=153.6, 132.2, 131.1, 131.0, 130.2, 129.38, 129.35, 128.98, 128.97, 128.5, 128.4, 128.1, 127.7, 127.0, 126.8, 126.7, 126.63, 126.58, 126.1, 123.7, 121.8. 119.5, 113.8 ppm. ESI‐HRMS: m/z calcd for C_26_H_16_NaO [M+Na]^+^ 367.1093, found 367.1036.

**Synthesis of dibenzo[*g,p*]chrysen‐1‐yl 1,1,2,2,3,3,4,4,4‐nonafluorobutane‐1‐sulfonate (DBzC‐ONf): DBzC‐OH** (30.0 mg, 87.1 μmol, 1.0 eq.) was dissolved in CH_2_Cl_2_ (5 mL) and NEt_3_ (360 μL, 26.4 mg, 261 μmol, 3.0 eq.) and perfluorobutane‐1‐sulfonyl fluoride (130 μL, 65.8 mg, 218 μmol, 2.5 eq.) were added under nitrogen atmosphere. The resulting mixture was stirred at rt for 23 h. Then water (10 mL) was added and it was extracted with CH_2_Cl_2_ (5×15 mL). All the organic extracts were dried over MgSO_4_, the desiccant was filtered off and the solvent was removed *in vacuo*. Subsequently, the residue was purified by column chromatography (SiO_2_, Hex/EtOAc 24 : 1) to afford **DBzC‐ONf** (45.0 mg, 71.8 μmol, 82 %) as colorless solid. ^1^H NMR (400 MHz, CDCl_3_) *δ*=9.03–8.88 (m, 1H, Ar−H), 8.75–8.68 (m, 4H, Ar−H), 8.67–8.62 (m, 1H, Ar−H), 8.54 (dd, *J*=8.1, 1.3 Hz, 1H, Ar−H), 7.76–7.57 (m, 8H, Ar−H) ppm. ^13^C NMR {^1^H} (100 MHz, CDCl_3_) *δ*=147.4, 132.7, 131.4, 131.3, 130.8, 129.21, 129.15, 129.1, 128.7, 128.7, 128.62, 128.59, 128.26, 128.24, 127.4, 127.1, 127.0, 126.93, 126.85, 126.7, 126.5, 124.3, 123.9, 123.8, 120.4 ppm. The very weak signals corresponding to the ONf group are in the 119.0‐106.8 range. ^19^F NMR {^1^H} (376 MHz, CDCl_3_) *δ*=−80.44 – (−80.72) (m, 3F), −109.19 – (−109.37) (m, 2F), −120.64 – (−120.84) (m, 2F), −125.69 – (−125.90) (m, 2F) ppm. ESI‐HRMS: m/z calcd for C_30_H_15_F_9_NaO_3_S [M+Na]^+^ 649.0490, found 649.0495.

**Synthesis of 2‐(dibenzo[*g,p*]chrysen‐1‐yl)‐4,4,5,5‐tetramethyl‐1,3,2‐dioxaborolane (DBzC‐Bpin)**: Bis(pinacolato)diborane (116 mg, 455 μmol, 1.5 eq.), KOAc (179 mg, 1.82 mmol, 6.0 eq.) and Pd(PPh_3_)_4_ (35.1 mg, 30.3 μmol, 0.1 eq.) were added under nitrogen atmosphere to **DBzC‐ONf** (190 mg, 303 μmol, 1.0 eq.) dissolved in degassed *N,N*‐dimethylformamide (15 mL). The resulting mixture was stirred at 90 °C for 16 h. Afterwards, water (30 mL) was added and it was extracted with CH_2_Cl_2_ (5×30 mL). All the organic extracts were dried over MgSO_4_, the desiccant was filtered off and the solvent was removed *in vacuo*. Subsequently, the residue was purified by column chromatography (SiO_2_, Hex/EtOAc 23 : 2) to yield **DBzC‐Bpin** (79.0 mg, 174 μmol, 57 %) as a colorless solid. ^1^H NMR (400 MHz, CDCl_3_) *δ*=8.78–8.68 (m, 4H, Ar−H), 8.65 (ddd, *J*=8.6, 8.0, 1.4 Hz, 2H, Ar−H), 8.40 (dd, *J*=7.8, 1.0 Hz, 1H, Ar−H), 7.88 (dd, *J*=7.0, 1.3 Hz, 1H, Ar−H), 7.72–7.51 (m, 7H, Ar−H), 1.50 (s, 12H, CH_3_) ppm. ^13^C NMR {^1^H} (100 MHz, CDCl_3_) *δ*=135.0, 133.0, 131.4, 131.1, 131.0, 130.5, 130.2, 129.5, 129.2, 129.09, 129.07, 129.05, 128.5, 128.3, 128.0, 127.6, 127.0, 126.7, 126.7, 126.6, 126.5, 126.2, 125.4, 123.7, 123.6, 84.5, 24.9 ppm. ^11^B NMR {^1^H} (128 MHz, CDCl_3_) *δ*=33.2 ppm. ESI‐HRMS: m/z calcd for C_32_H_27_BNaO_2_ [M+Na]^+^ 477.1996, found 477.1979.

**Synthesis of 2‐(dibenzo[*g,p*]chrysen‐1‐yl)pyridine (BA1): DBzC‐Bpin** (40.0 mg, 88.0 μmol, 1.0 eq.), 2‐bromopyridine (18.1 mg, 114 μmol, 1.3 eq), Cs_2_CO_3_ (57.4 mg, 176 μmol, 2.0 eq.) and Pd(PPh_3_)_4_ (5.10 mg, 4.40 μmol, 0.05 eq.) were dissolved in a degassed mixture of DME/EtOH/H_2_O (4 mL, 2 : 1 : 1). The resulting reaction mixture was stirred at 85 °C under nitrogen atmosphere for 20 h. Afterwards, aq. sat. NaHCO_3_ solution (5 mL) was added and it was extracted with EtOAc (5×10 mL). All the organic extracts were dried over MgSO_4_, the desiccant was filtered off and the solvent was removed *in vacuo*. Subsequently, the residue was purified by column chromatography (SiO_2_, Hex/EtOAc 7 : 3) to afford **BA1** (26.0 mg, 64.1 μmol, 66 %) as a colorless solid. ^1^H NMR (400 MHz, CD_2_Cl_2_) *δ*=8.87–8.69 (m, 5H, Ar−H), 8.67 (dd, *J*=7.7, 1.2 Hz, 1H, Ar−H), 8.55 (d, *J*=8.2 Hz, 1H, Ar−H), 7.82–7.61 (m, 7H, Ar−H), 7.50–7.32 (m, 4H, Ar−H), 7.10 (m, 1H, Ar−H) ppm. ^13^C NMR {^1^H} (100 MHz, CD_2_Cl_2_) *δ*=162.1, 149.6, 138.8, 137.6, 131.6, 131.1, 131.0, 130.7, 130.0, 129.7, 129.5, 129.3, 129.23, 129.18, 129.16, 129.0, 128.7, 127.6, 127.20, 127.16, 127.03, 126.98, 126.9, 125.5, 124.9, 124.0, 123.9, 122.6 ppm. ESI‐HRMS: m/z calc. for C_31_H_20_N [M+H]^+^ 406.1590 found 406.1593.

**Synthesis of EH1: BA1** (25.0 mg, 61.7 μmol, 1.0 eq.) was dissolved in CH_2_Cl_2_ (3 mL) under nitrogen atmosphere. Then *N,N*‐diisopropylethylamine (11.5 μL, 8.80 mg, 67.8 μmol, 1.1 eq.) was added and the mixture was cooled to −78 °C. Then BBr_3_ (1.0 m in CH_2_Cl_2_; 185 μL, 185 μmol, 3.0 eq.) was added dropwise. The reaction mixture was warmed to rt and stirred at rt for 22 h. The solvent was removed *in vacuo* and the resulting orange solid was washed with dry hexane (3×4 mL) under nitrogen atmosphere. Subsequently, the residue was dissolved in CH_2_Cl_2_ (3 mL) and AlMe_3_ (2.0 m in toluene; 91.3 μL, 183 μmol, 3.0 eq.) was added dropwise under nitrogen atmosphere. The resulting mixture was stirred at rt for 30 min. Then it was cooled to 0 °C and water (4 mL) was added. Afterwards, it was extracted with CH_2_Cl_2_ (5×10 mL). The combined organic phases were then dried over MgSO_4_, the desiccant was filtered off and the solvent was removed *in vacuo*. Subsequently, the residue was purified by column chromatography (SiO_2_, Hex/ EtOAc 4 : 1) to afford **EH1** (18.0 mg, 40.4 μmol, 66 %) as a yellowish solid. ^1^H NMR (400 MHz, CD_2_Cl_2_) *δ*=8.79–8.67 (m, 5H, Ar−H), 8.64 (d, *J*=8.2 Hz, 1H, Ar−H), 8.56 (dd, *J*=8.1, 1 Hz, 1 H, Ar−H), 8.55 (ddd, *J*=5.6, 1.5, 0.8 Hz, 1H, Ar−H), 8.50 (d, *J*=8.4 Hz, 1H, Ar−H), 7.93 (d, *J*=8.0 Hz, 1H, Ar−H), 7.84 (ddd, *J*=8.5, 7.4, 1.6 Hz, 1H, Ar−H), 7.75–7.66 (m, 4H, Ar−H), 7.63 (ddd, *J*=8.4, 7.1, 1.3 Hz, 1H, Ar−H), 7.47 (ddd, *J*=8.2, 7.1, 1.2 Hz, 1H, Ar−H), 7.37 (ddd, *J*=6.8, 5.7, 1.1 Hz, 1H, Ar−H), 0.28 (s, 3H, CH_3_), 0.06 (s, 3H, CH_3_) ppm. ^13^C NMR {^1^H} (100 MHz, CD_2_Cl_2_) *δ*=158.4, 142.8, 138.7, 131.5, 131.2, 131.1, 130.8, 129.7, 129.6, 129.5, 129.4, 129.3, 129.2, 128.9, 128.8, 128.6, 128.4, 128.3, 128.0, 127.8, 127.7, 127.2, 126.9, 126.81, 126.77, 124.6, 124.0, 123.9, 122.2, 121.6 ppm. ^11^B NMR {^1^H} (128 MHz, CDCl_3_) *δ*=0.4 ppm. ESI‐HRMS: m/z calcd for C_33_H_24_BNNa [M+Na]^+^ 468.1894, found 468.1891.

**Synthesis of 1‐(dibenzo[*g,p*]chrysen‐1‐yl)isoquinoline (BA2)**: 1‐chloroisoquinoline (30.0 mg, 183 μmol, 1.0 eq.), **DBzC‐Bpin** (91.7 mg, 202 μmol, 1.1 eq.), Cs_2_CO_3_ (239 mg, 733 μmol, 4.0 eq.) and Pd(PPh_3_)_4_ (21.2 mg, 18. μmol, 0.1 eq.) were dissolved in a degassed mixture of DME/EtOH/H_2_O (4 mL, 2 : 1 : 1). The resulting mixture was stirred at 85 °C under nitrogen atmosphere for 20 h. Afterwards, aq. sat. NaHCO_3_ solution (10 mL) was added, and it was extracted with EtOAc (5×10 mL). All the organic extracts were dried over MgSO_4_, the desiccant was filtered off and the solvent was removed *in vacuo*. Subsequently, the residue was purified by column chromatography (SiO_2_, Hex/EtOAc 4 : 1, 1 % NEt_3_) to afford **BA2** (46.0 mg, 101 μmol, 53 %) as a colorless solid. ^1^H NMR (400 MHz, CD_2_Cl_2_) *δ*=8.84 (dd, *J*=8.2, 1.2 Hz, 1H, Ar−H), 8.78‐8.73 (m, 3H, Ar−H), 8.72 (dd, *J*=8.0, 1.4 Hz, 1H, Ar−H), 8.69 (d, *J*=5.7 Hz, 1H, Ar−H), 8.54 (dd, *J*=8.3, 1.3 Hz, 1H, Ar−H), 7.96 (d, *J*=8.3 Hz, 1H, Ar−H), 7.82 (dd, *J*=5.7, 0.7 Hz, 1H, Ar−H), 7.81–7.62 (m, 7H, Ar−H), 7.54 (br d, *J*=5.6 Hz, 1H, Ar−H), 7.34 (ddd, *J*=8.3, 7.0, 1.2 Hz, 2H, Ar−H), 7.21 (dd, *J*=8.3, 0.7 Hz, 1H, Ar−H), 6.86 (ddd, *J*=8.4, 7.1, 1.3 Hz, 1H, Ar−H) ppm. ^13^C NMR (100 MHz, CD_2_Cl_2_) *δ*=163.8, 143.2, 137.9, 137.1, 131.4, 131.0, 130.9, 130.8, 130.53, 130.46, 130.1, 129.5, 129.4, 129.30, 129.28, 129.1, 128.7, 128.6, 128.5, 127.9, 127.7, 127.6, 127.5, 127.4, 127.2, 127.12, 127.06, 127.0, 126.8, 126.7, 125.3, 124.04, 123.95, 120.5 ppm. ESI‐HRMS: m/z calcd for C_35_H_22_N [M+H]^+^ 456.1747, found 456.1770.

**Synthesis of EH2: BA2** (31.0 mg, 68.1 μmol, 1.0 eq.) was dissolved in CH_2_Cl_2_ (3 mL) under nitrogen atmosphere. Then *N,N*‐diisopropylethylamine (12.7 μL, 9.70 mg, 74.9 μmol, 1.1 eq.) was added and the mixture was cooled to −78 °C. Then BBr_3_ (1.0 m in CH_2_Cl_2_; 204 μL, 204 μmol, 3.0 eq.) was added dropwise. The reaction mixture was warmed to rt and stirred at rt for 18 h. The solvent was removed *in vacuo* and the resulting orange solid was washed with dry hexane (3 x 4 mL) under nitrogen atmosphere. Subsequently, the residue was dissolved in CH_2_Cl_2_ (3 mL) and AlMe_3_ (2.0 m in toluene; 103 μL, 206 μmol, 3.0 eq.) was added dropwise under nitrogen atmosphere. The resulting mixture was stirred at rt for 2 h. Then it was cooled to 0 °C and water (4 mL) was added. Afterwards, it was extracted with CH_2_Cl_2_ (5×10 mL). The combined organic phases were dried over MgSO_4_, the desiccant was filtered off and the solvent was removed *in vacuo*. Subsequently, the residue was purified by column chromatography (SiO_2_, Hex/EtOAc 4 : 1) to afford **EH2** (14.0 mg, 28.3 μmol, 41 %) as a yellow solid. ^1^H NMR (400 MHz, CD_2_Cl_2_) *δ*=8.97–8.91 (m, 1H, Ar−H), 8.84 (d, *J*=7.9 Hz, 1H, Ar−H), 8.83–8.74 (m, 3H, Ar−H), 8.65 (d, *J*=7.8 Hz, 1H, Ar−H), 8.42 (d, *J*=6.3 Hz, 1H, Ar−H), 8.05 (d, *J*=7.9 Hz, 1H, Ar−H), 7.93 (d, *J*=8.2 Hz, 1H, Ar−H), 7.88 (dd, *J*=8.6, 0.8 Hz, 1H, Ar−H), 7.82 (d, *J*=6.2 Hz, 1H, Ar−H), 7.80–7.63 (m, 6H, Ar−H), 7.43 (ddd, *J*=8.4, 7.1, 1.3 Hz, 1H, Ar−H), 7.16 (ddd, *J*=8.4, 6.9, 1.2 Hz, 1H, Ar−H), 6.94 (ddd, *J*=8.2, 7.1, 1.2, 1H, Ar−H), 0.35 (s, 3H, CH_3_), 0.08 (s, 3H, CH_3_) ppm. ^13^C NMR {^1^H} (100 MHz, CD_2_Cl_2_) *δ*=159.4, 138.0, 134.4, 132.0, 131.8, 131.6, 131.2, 130.7, 130.3, 130.1, 129.7, 129.52, 129.48, 129.4, 129.3, 129.2, 128.7, 128.0, 127.4, 127.2, 127.1, 127.04, 126.98, 126.80, 126.78, 126.76, 125.0, 124.9, 124.00, 123.9, 120.9 ppm. ^11^B NMR {^1^H} (128 MHz, CDCl_3_) *δ*=0.7 ppm. APCI‐DIP‐HRMS: m/z calcd for C_37_H_27_BN [M+H]^+^ 496.2231, found 496.2257.

**Synthesis of 1‐(dibenzo[*g,p*]chrysene‐1‐yl)benzo[*h*]isoquinoline (BA3): DBzC‐Bpin** (40.0 mg, 88.0 μmol, 1.0 eq.), **BIQ‐Cl** (24.5 mg, 114 μmol, 1.3 eq.), Cs_2_CO_3_ (57.4 mg, 176 μmol, 2.0 eq.) and Pd(PPh_3_)_4_ (5.10 mg, 4.40 μmol, 0.05 eq.) were dissolved in a degassed mixture of DME/EtOH/H_2_O (4 mL, 2 : 1 : 1). The resulting mixture was stirred at 85 °C under nitrogen atmosphere for 20 h. Afterwards, aq. sat. NaHCO_3_ solution (5 mL) was added and it was extracted with EtOAc (5×10 mL). All the organic extracts were dried over MgSO_4_, the desiccant was filtered off and the solvent was removed *in vacuo*. Subsequently, the residue was purified by column chromatography (SiO_2_, Hex/EtOAc 7 : 3, 1 % NEt_3_) to afford **BA3** (35.0 mg, 69.2 μmol, 78 %) as a colorless solid. ^1^H NMR (400 MHz, CD_2_Cl_2_) *δ*=8.89‐8.80 (m, 2H, Ar−H), 8.80‐8.74 (m, 2H, Ar−H), 8.71 (d, *J*=5.4 Hz, 1H, Ar−H), 8.61 (d, *J*=8.1 Hz, 1H, Ar−H), 8.50 (d, *J*=8.1 Hz, 1H, Ar−H), 8.04 (d, *J*=8.8 Hz, 2H, Ar−H), 7.92 (dd, *J*=8.0, 1.1 Hz, 1H, Ar−H), 7.87 (d, *J*=8.8 Hz, 1H, Ar−H), 7.85 (d, *J*=5.4 Hz, 1H, Ar−H), 7.80–7.62 (m, 5H, Ar−H), 7.53 (br d, *J*=7.8 Hz, 1H, Ar−H), 7.47 (ddd, *J*=7.9, 7.0, 0.9 Hz, 1H, Ar−H), 7.40 (br d, *J* =5.8 Hz, 1H, Ar−H), 7.34 (ddd, *J*=8.3, 7.1, 1.2 Hz, 1H, Ar−H), 7.14–7.03 (m, 1H, Ar−H), 6.87 (ddd, *J*=8.3, 7.1, 1.3 Hz, 1H, Ar−H) ppm. ^13^C NMR {^1^H} (100 MHz, CD_2_Cl_2_) *δ*=161.4, 144.5, 142.5, 139.0, 133.9, 132.5, 131.8, 131.40, 131.39, 130.9, 130.2, 129.8, 129.7, 129.5, 129.4, 129.31, 129.30, 129.23, 129.18, 129.1, 128.7, 128.6, 128.3, 127.9, 127.5, 127.3, 127.19, 127.17, 127.10, 127.08, 127.02, 126.98, 126.8, 126.0, 125.4, 124.9, 124.0, 123.9, 121.1 ppm. ESI‐HRMS: m/z calcd for C_39_H_24_N [M+H]^+^ 506.1903, found 506.1928.

**Synthesis of EH3: BA3** (28.0 mg, 55.4 μmol, 1.0 eq.) was dissolved in CH_2_Cl_2_ (3 mL) under nitrogen atmosphere. Then *N,N*‐diisopropylethylamine (10.4 μL, 7.90 mg, 60.9 μmol, 1.1 eq.) was added and the mixture was cooled to −78 °C. Then BBr_3_ (1.0 m in CH_2_Cl_2_; 166 μL, 166 μmol, 3.0 eq.) was added dropwise. The reaction mixture was warmed to rt and stirred at rt for 22 h. The solvent was removed *in vacuo* and the resulting orange solid was washed with dry hexane (3×4 mL) under nitrogen atmosphere. Subsequently, the residue was dissolved in CH_2_Cl_2_ (3 mL) and AlMe_3_ (2.0 m in toluene; 83.3 μL, 167 μmol, 3.0 eq.) was added dropwise under nitrogen atmosphere. The resulting mixture was stirred at rt for 30 min. Then it was cooled to 0 °C and water (4 mL) was added. Afterwards, it was extracted with CH_2_Cl_2_ (5×10 mL). The combined organic phases were dried over MgSO_4_, the desiccant was filtered off and the solvent was removed *in vacuo*. Subsequently, the residue was purified by column chromatography (SiO_2_, Hex/ EtOAc 4 : 1) to afford **EH3** (10.0 mg, 18.3 μmol, 33 %) as a yellow solid. ^1^H NMR (400 MHz, CD_2_Cl_2_) *δ*=8.88 (d, *J*=7.9 Hz, 1H, Ar−H), 8.82‐8.72 (m, 3H, Ar−H), 8.62‐8.57 (m, 1H, Ar−H), 8.55 (d, *J*=5.8 Hz, 1H, Ar−H), 8.17 (d, *J*=7.8 Hz, 1H, Ar−H), 8.10 (d, *J*=7.9 Hz, 1H, Ar−H), 7.98 (d, *J*=8.7 Hz, 1H, Ar−H), 7.84 (d, *J*=5.9 Hz, 1H, Ar−H), 7.81–7.74 (m, 3H, Ar−H), 7.74–7.70 (m, 2H, Ar−H), 7.67 (d, *J*=7.8 Hz, 1H, Ar−H), 7.52 (d, *J*=8.4 Hz, 1H, Ar−H), 7.37 (ddd, *J*=8.0, 7.1, 1.1 Hz, 1H, Ar−H), 7.26 (dd, *J*=8.2, 0.8 Hz, 1H, Ar−H), 6.99 (ddd, *J*=8.3, 7.1, 1.3 Hz, 1H, Ar−H), 6.59 (ddd, *J*=8.3, 7.0, 1.3 Hz, 1H, Ar−H), 6.38 (ddd, *J*=8.2, 7.1, 1.1 Hz, 1H, Ar−H), 0.37 (s, 3H, CH_3_), 0.20 (s, 3H, CH_3_) ppm. ^13^C NMR {^1^H} (100 MHz, CD_2_Cl_2_) *δ*=156.8, 139.1, 135.9, 134.0, 133.9, 132.4, 131.5, 131.1, 130.5, 130.2, 129.98, 129.95, 129.7, 129.6, 129.5, 129.0, 128.4, 128.3, 128.1, 127.7, 127.5, 127.4, 127.34, 127.30, 127.0, 126.8, 126.7, 126.1, 125.8, 124.4, 124.1, 124.04, 123.97, 123.9, 120.3 ppm. ^11^B NMR {^1^H} (128 MHz, CDCl_3_) *δ*=1.4 ppm. APCI‐DIP‐HRMS: m/z calcd for C_41_H_29_BN [M+H]^+^ 546.2388, found 546.2398.

### X‐ray crystallography

Deposition Numbers 2175284 (for **EH2**), and 2175285 (for **EH3**) contain the supplementary crystallographic data for this paper. These data are provided free of charge by the joint Cambridge Crystallographic Data Centre and Fachinformationszentrum Karlsruhe Access Structures service.

Further experimental data, original spectra and computational details are provided in the Supporting Information.

## Conflict of interest

The authors declare no conflict of interest.

1

## Supporting information

As a service to our authors and readers, this journal provides supporting information supplied by the authors. Such materials are peer reviewed and may be re‐organized for online delivery, but are not copy‐edited or typeset. Technical support issues arising from supporting information (other than missing files) should be addressed to the authors.

Supporting Information

## Data Availability

The data that support the findings of this study are available in the supplementary material of this article.
