# Supplementary material for: Enhanced Optical Properties of Azaborole Helicenes by Lateral and Helical Extension
Source: Chemistry. 2022 Sep 2;28(62):e202202280. doi: 10.1002/chem.202202280 (PMC9826013; doi:10.1002/chem.202202280)
Supplement: Supplementary file 1 — Supporting Information [file CHEM-28-0-s001.pdf]

Chemistry–A European Journal

Supporting Information

Enhanced Optical Properties of Azaborole Helicenes by Lateral and Helical Extension

Felix Full, Quentin Wölflick, Krzysztof Radacki, Holger Braunschweig, and Agnieszka Nowak-Król*

Table of Contents

1. Materials and Methods S1
2. Synthesis and characterization of π-extended helicenes S2
3. NMR and MS spectra S4
4. Single crystal X-ray analysis S27
5. Optical and electrochemical properties S32
6. Resolution of enantiomers S33
7. Interconversion Barrier S34
8. Chiroptical properties S36
9. Computations S37
10. References S128

1. Materials and Methods

Synthesis: All reagents were purchased from commercial sources and used as received without further purification, unless otherwise stated. Reagent grade solvents were distilled prior to use. Column chromatography was performed on silica (silica gel, 230–400 mesh).

NMR Spectroscopy: ^{1}H-, ^{11}B-, ^{13}C- and ^{19}F-NMR spectra were recorded on a Bruker Avance 400 or an Avance III HD 400 spectrometers and were calibrated to the residual solvent signals. *J* values are given in Hz. The following abbreviations were used to designate multiplicities: s = singlet, d = doublet, t = triplet, dd = doublet of doublets, ddd = doublet of doublet of doublets, dm = doublet of multiplets, dt = doublet of triplets, td = triplet of doublets, m = multiplet, br s = broad singlet.

Mass Spectrometry: High-resolution mass spectra were obtained by electrospray ionization (ESI) or atmospheric- pressure chemical ionization (APCI). ESI and APCI spectra were recorded on an ESI micrOTOF Focus spectrometer from Bruker Daltonics.

UV/Vis Spectroscopy in Solution: UV/Vis spectra were recorded on a Jasco V-770 UV/Vis spectrometer. All spectroscopy measurements were conducted with spectroscopic grade solvents from ACROS Organics. Conventional quartz cells (light path 1 cm) were used.

Fluorescence Spectroscopy in Solution: Emission spectra were recorded using a FLS 980 fluorescence spectrometer from Edinburgh Instruments equipped with a double monochromator for emission and excitation. The spectra were corrected against photomultiplier and lamp intensity. The fluorescence quantum yields were determined by the optical dilution method (OD ≤ 0.05)[S1] as the average value of six different excitation wavelengths with perylene (Φ_{fl} = 0.94 in cyclohexane)[S2] as a standard.

Fluorescence Spectroscopy in the Solid State: Absolute fluorescence quantum yields of powders were determined on a HamamatsuAbsolute PL Quantum Yield Measurement System CC9920-02. The system is composed of a 150 W CW Xenon lamp as the excitationsource, a monochromator (250−700 nm, full width at half-maximum (FWHM) 10 nm), an integrating sphere, and a multichannel spectrometer capable of simultaneously measuring multiple wavelengths between 300 and 950 nm.

Circular Dichroism (CD) and Circularly Polarized Luminescence (CPL) Spectroscopy: CD and CPL spectra were recorded on a customized Jasco CPL-300/J-1500 hybrid spectrometer.

Electrochemistry: Cyclic voltammetry (CV) and differential pulse voltammetry (DPV) measurements were performed on a standard, commercial electrochemical analyzer (EC epsilon; BAS Instruments, UK) in a three electrode single compartment cell under an argon atmosphere. The supporting electrolyte NBu_4PF_6 was synthesized according to the literature,[S3] recrystallized from ethanol/water, and dried in a high vacuum. The measurements were carried out in CH_2Cl_2/0.1 M NBu_4PF_6 under the exclusion of air and moisture at a concentration of *c* ~ 2.5-2.8 × 10^{-4} M with the ferrocenium/ferrocene redox couple as an internal standard for the calibration of the potential. Working electrode: glassy carbon (Ø 1 mm); reference electrode: Ag/AgCl; auxiliary electrode: Pt wire. The internal resistance was compensated by 50%.

2. Synthesis and characterization of π-extended helicenes

The synthesis of **BIQ-Cl** and **DBzC-Bpin** are shown in Scheme S1.

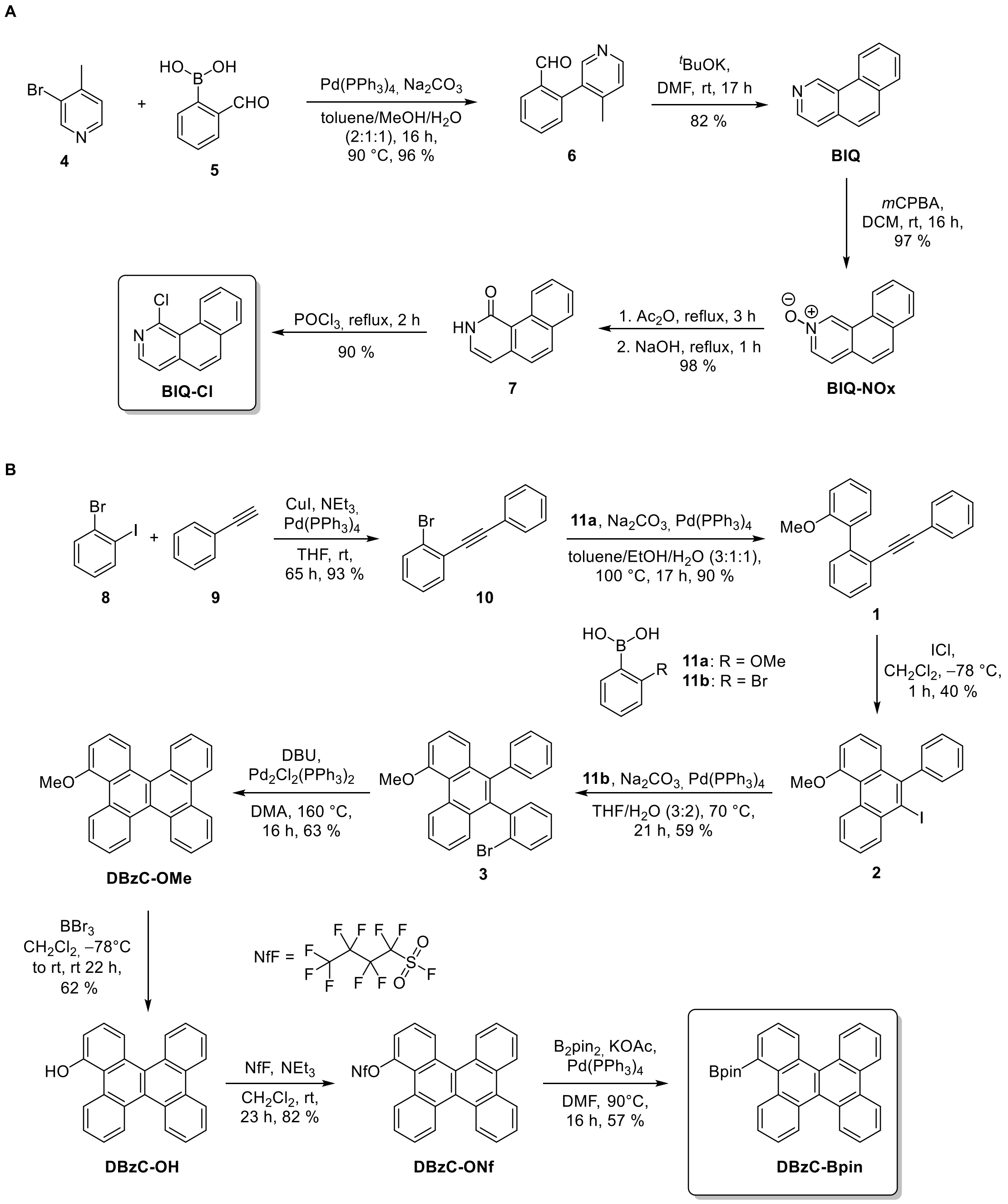

Scheme S1. Synthesis of building blocks **BIQ-Cl** (**A**) and **DBzC-Bpin** (**B**).

The synthesis of building block **BIQ-Cl** (Scheme S1) was performed according to the reported procedures.[S4] The modified procedures are listed below.

Synthesis of benzo[*h*]isoquinoline 2-oxide (BIQ-NOx).

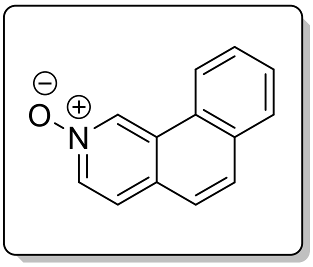

3-Chloroperbenzoic acid (5.97 g, 34.6 mmol, 2.0 eq.) was given to a solution of **BIQ** (3.10 g, 17.3 mmol, 1.0 eq.) in CH_2Cl_2 (40 mL) and the mixture was stirred at rt for 16 h. Subsequently, MeOH (10 mL) was added to the mixture to dissolve the precipitated solid. Afterwards, saturated aqueous $NaHCO_3$ solution (40 mL) was added and the aqueous phase was extracted with CH_2Cl_2 (4 × 30 mL). All the organic extracts were dried over Na_2SO_4, the desiccant was filtered off and the solvent was removed *in vacuo*. Subsequently, the residue was purified by column chromatography (SiO_2, EtOAc/MeOH 9:1) yielding **BIQ-NOx** (3.29 g, 16.9 mmol, 97 %) as a beige solid. **^{1}H-NMR (400 MHz, $CDCl_3$) *δ* =** 9.51 (s, 1H, Ar-H), 8.46-8.40 (m, 1H, Ar-H), 8.31 (dd, *J* = 6.9, 1.7 Hz, 1H, Ar-H), 7.94-7.91 (m, 1H, Ar-H), 7.85 (d, *J* = 8.8, 1H, Ar-H), 7.76-7.69 (m, 3H, Ar-H), 7.64 (d, *J* = 8.8 Hz, 1H, Ar-H) ppm. Analytical data are in accordance with the literature.[S4]

Synthesis of 1-chlorobenzo[*h*]isoquinoline (BIQ-Cl).

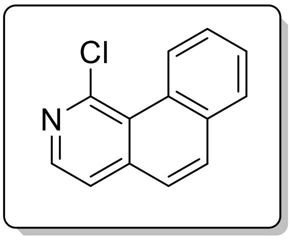

7 (2.00 g, 10.2 mmol, 1.0 eq.) was dissolved in $POCl_3$ (40.0 mL, 64.4 g, 420 mmol, 41.0 eq.) and stirred under reflux for 2 h under nitrogen atmosphere. It was cooled to 0°C and H_2O (200 mL) was added. Subsequently, it was stirred for 1 h at rt and then 2 M NaOH was added until pH reached 7. Afterwards, it was extracted with EtOAC (5 × 200 mL). All the organic extracts were dried over $MgSO_4$, the desiccant was filtered off and the solvent was removed *in vacuo*. The crude product was then purified by column chromatography (SiO_2, Hex/EtOAc, 7:3, 1 % NEt_3) yielding **BIQ-Cl** (1.97 g, 9.22 mmol, 90 %) as a yellow solid. **^{1}H-NMR (400 MHz, $CDCl_3$) *δ* =** 9.93-9.879 (dm, *J* = 8.4Hz, 1H, Ar-H), 8.45 (d, *J* = 5.1 Hz, 1H, Ar-H), 7.99-7.95 (m, 2H, Ar-H), 7.79-7.68 (m, 4H, Ar-H) ppm. Analytical data are in accordance with the literature.[S4]

Synthesis of 1-bromo-2-(phenylethynyl)benzene (10).

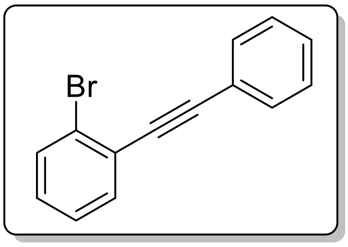

CuI (113 mg, 594 µmol, 0.06 eq.) and $Pd(PPh_3)_4$ (343 mg, 297 µmol, 0.03 eq.) were added to a Schlenk flask and it was evacuated and purged with nitrogen three times. Afterwards, NEt_3 (4.18 mL, 3.03 g, 30.0 mmol, 3.0 eq.) and THF (10 mL) were added to the flask and it was stirred at rt for 5 min. Subsequently, 1-bromo-2-iodobenzene (1.27 mL, 2.80 g, 9.90 mmol, 1.0 eq.), phenylacetylene (1.15 ml, 1.07 g, 10.5 mmol, 1.1 eq.) and THF (5 mL) were added successively to the mixture and it was stirred at rt for 65 h. The mixture was filtered through a short pad of celite, the remaining solid was washed with Et_2O (100 mL) and the solvent was removed *in vacuo*. Subsequently, the crude product was purified by column filtration (SiO_2, Hex) to yield **10** (2.36 g, 9.18 mmol, 93 %) as a colorless oil. **^{1}H-NMR (400 MHz, $CDCl_3$) *δ*** = 7.64-7.51 (m, 4H, Ar-H), 7.40-7.33 (m, 3H, Ar-H), 7.3 (ddd, *J* = 8.2, 7.5, 1.2 Hz, 1H, Ar-H), 7.21-7.15 (ddd, *J* = 8.4, 7.6,1.6 Hz, 1H, Ar-H) ppm. Analytical data are in accordance with the literature.[S5]

Synthesis of 2-methoxy-2'-(phenylethynyl)-1,1'-biphenyl (1).

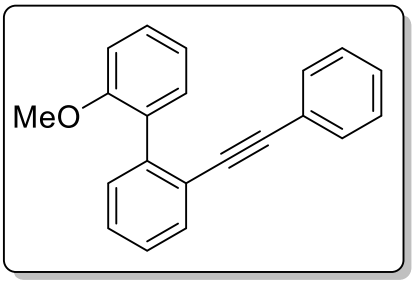

10 (2.00 g, 7.78 mmol, 1.0 eq.) was dissolved in a mixture of toluene/EtOH/H_2O (50 mL, 3:1:1). Na_2CO_3 (3.30 g, 31.1 mmol, 4.0 eq.) was added and the mixture was degassed (argon bubbling). Afterwards, 2-(methoxyphenyl)boronic acid (**11a**; 1.54 g, 10.1 mmol, 1.3 eq.) and $Pd(PPh_3)_4$ (629 mg, 544 µmol, 0.07 eq.) were added under nitrogen atmosphere and the reaction mixture was heated to 100 °C and stirred for 17 h. The reaction mixture was cooled to rt, H_2O (50 mL) was added and it was extracted with EtOAc (4 x 100 mL). The combined organic phases were dried over $MgSO_4$, filtered and concentrated *in vacuo*. Subsequently, the crude product was purified by column chromatography (SiO_2, Hex/EtOAc 95:5) to yield **1** (2.00 g, 7.03 mmol, 90 %) as an orange solid. **^{1}H-NMR (400 MHz, $CDCl_3$) *δ*** = 7.64-7.60 (m, 1H, Ar-H), 7.41-7.30 (m, 5H, Ar-H), 7.26-7.18 (m, 5H, Ar-H), 7.05 (ddd, *J* = 8.2, 7.4, 1.1 Hz, 1H, Ar-H), 7.03-6.99 (m, 1H, Ar-H), 3.78 (s, 3H, OCH_3) ppm. Analytical data are in accordance with the literature.[S6]

3. NMR and MS spectra

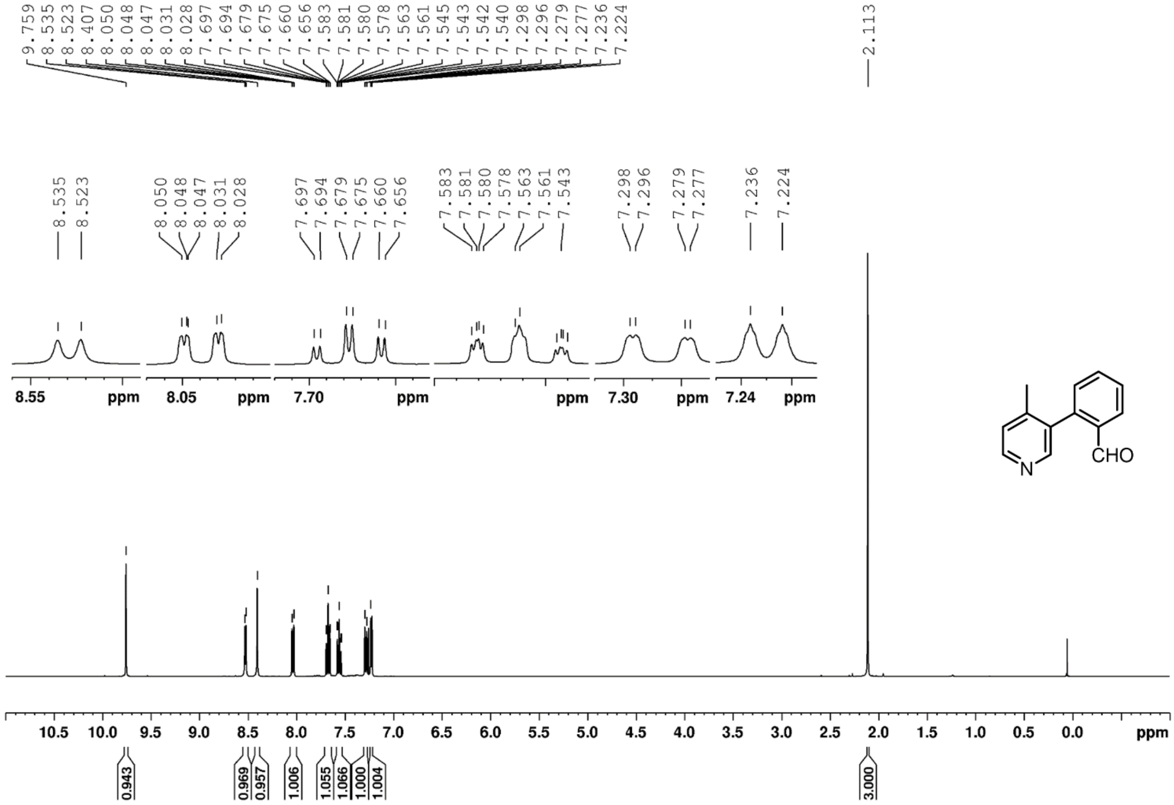

Figure S1. ^{1}H-NMR of compound **6** (400 MHz, $CDCl_3$, 25 °C).

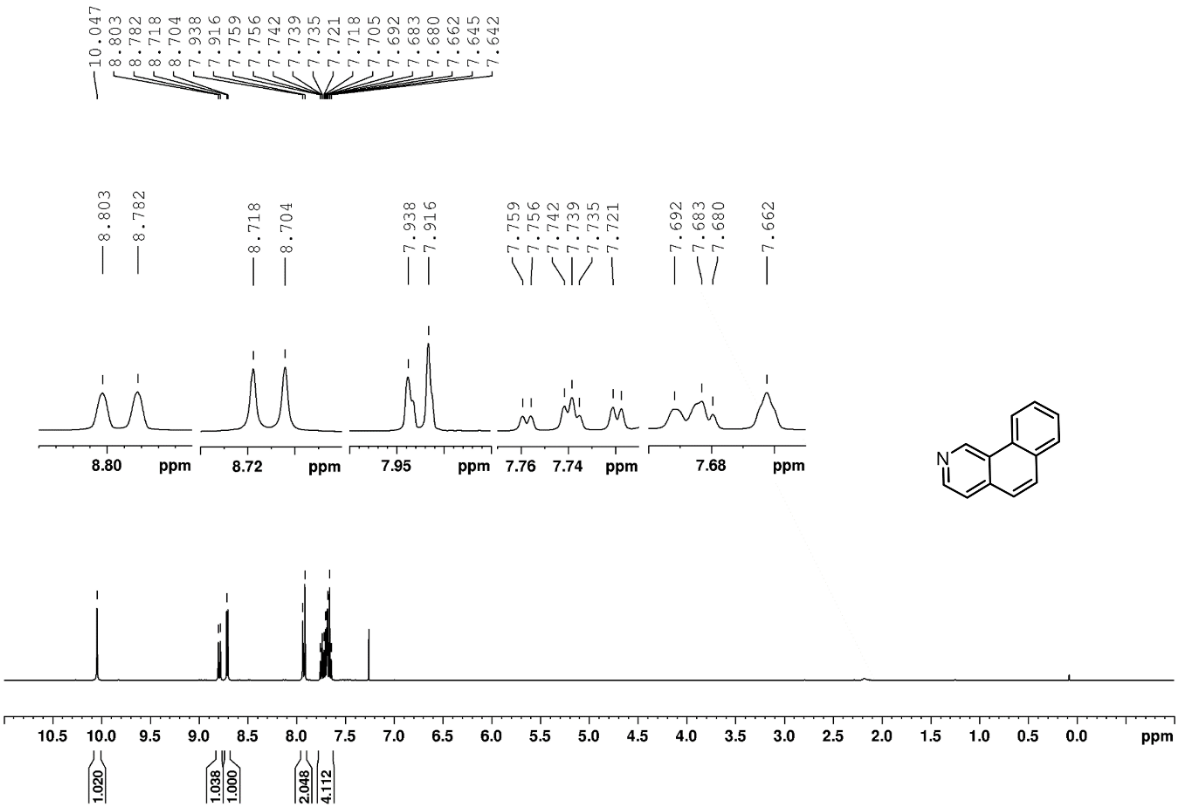

Figure S2. ^{1}H-NMR of compound **BIQ** (400 MHz, $CDCl_3$, 25 °C).

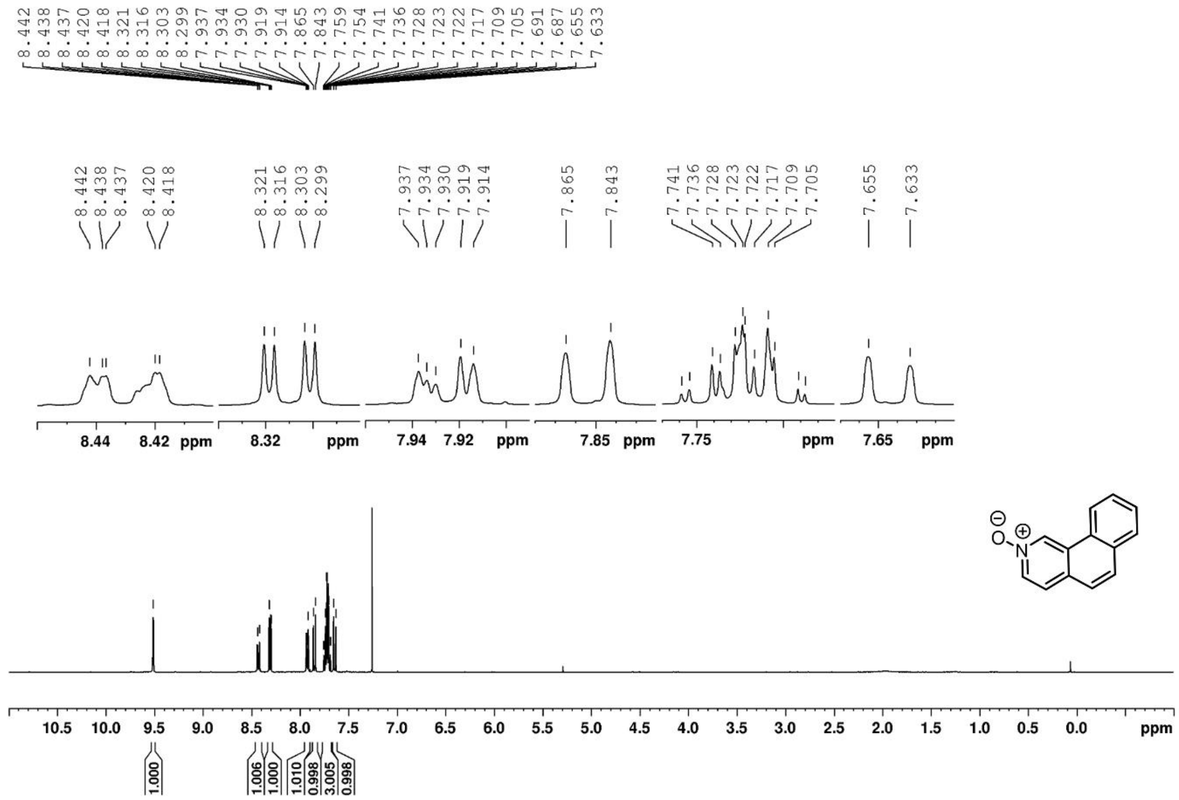

Figure S3. ^{1}H-NMR of compound **BIQ-NOx** (400 MHz, $CDCl_3$, 25 °C).

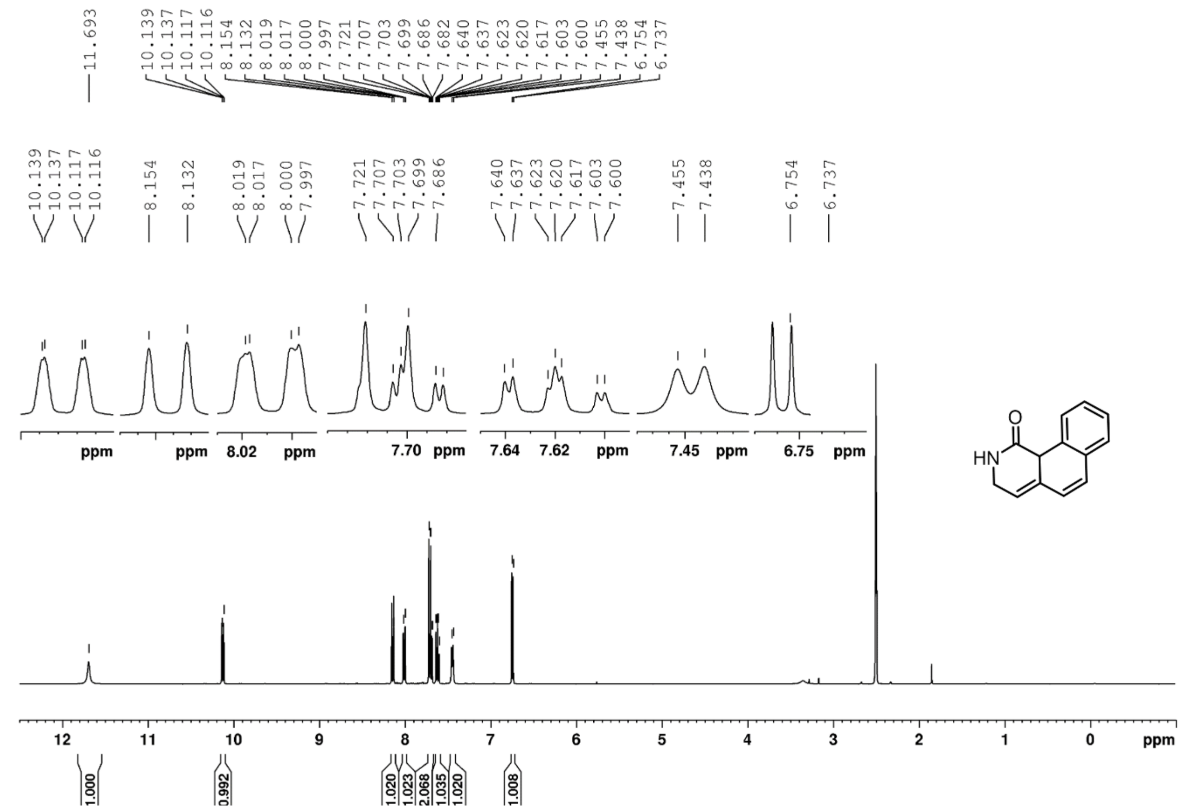

Figure S4. ^{1}H-NMR of compound **7** (400 MHz, DMSO-d_6, 25 °C).

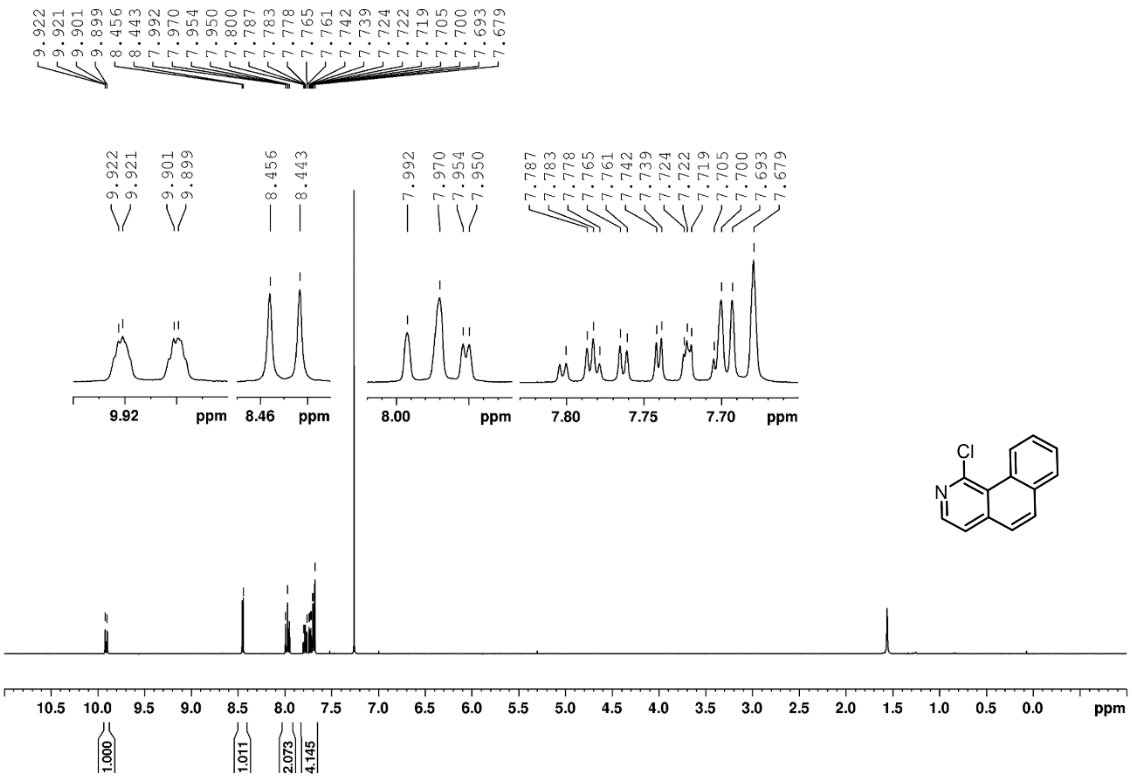

Figure S5. ^{1}H-NMR of compound **BIQ-Cl** (400 MHz, $CDCl_3$, 25 °C)

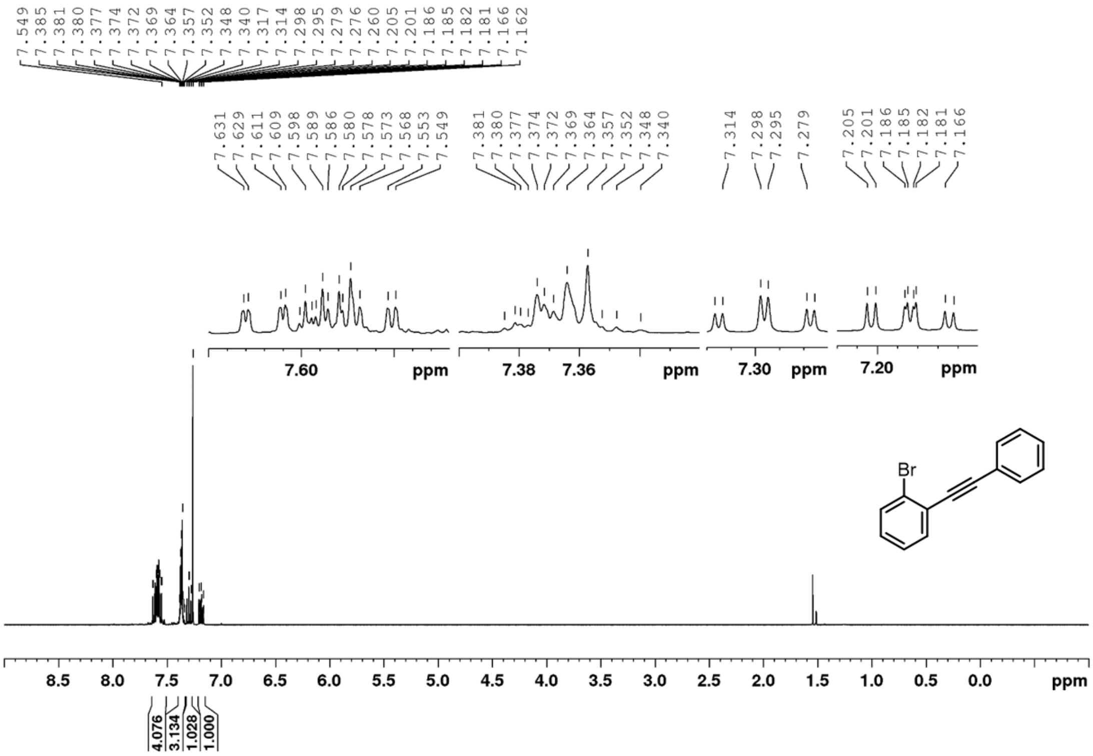

Figure S6. ^{1}H-NMR of compound **10** (400 MHz, $CDCl_3$, 25 °C)

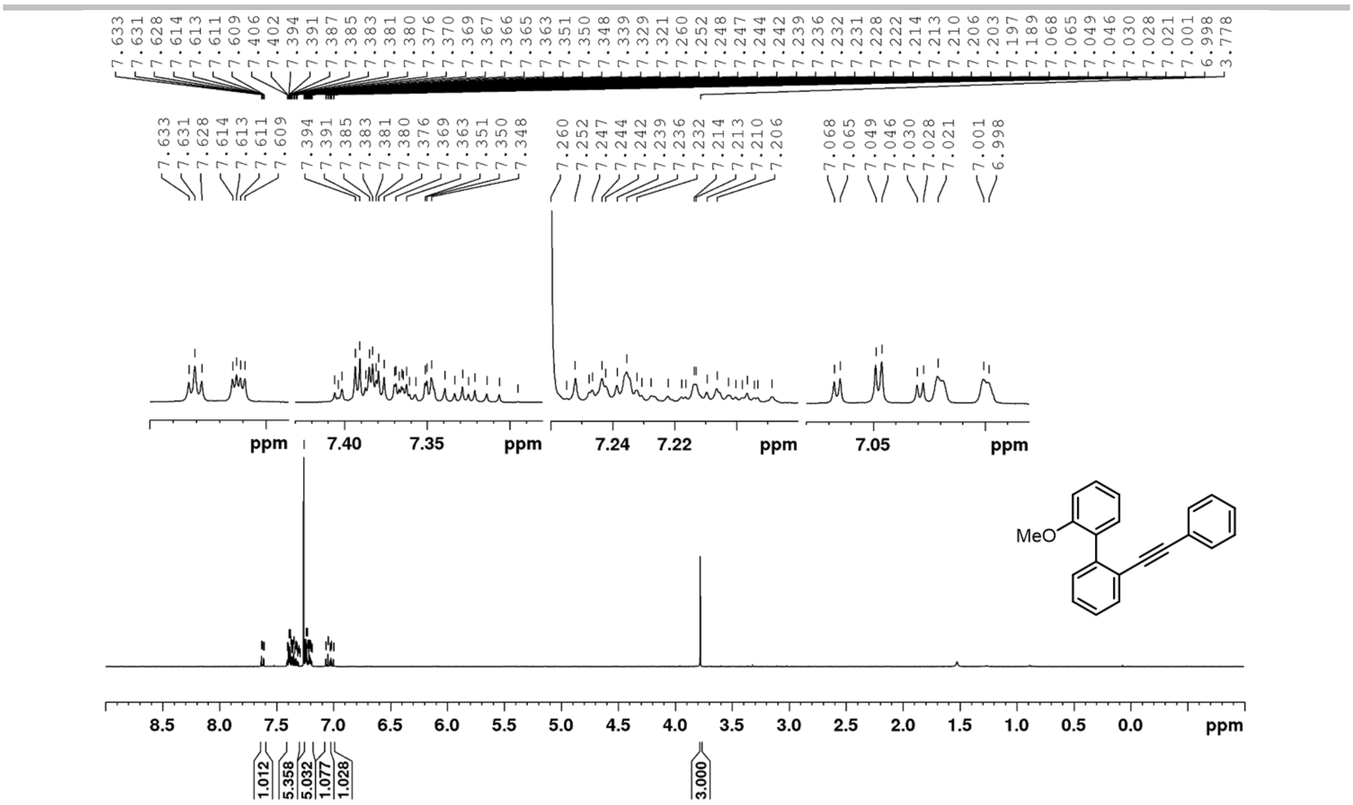

Figure S7. ^{1}H-NMR of compound **1** (400 MHz, $CDCl_3$, 25 °C).

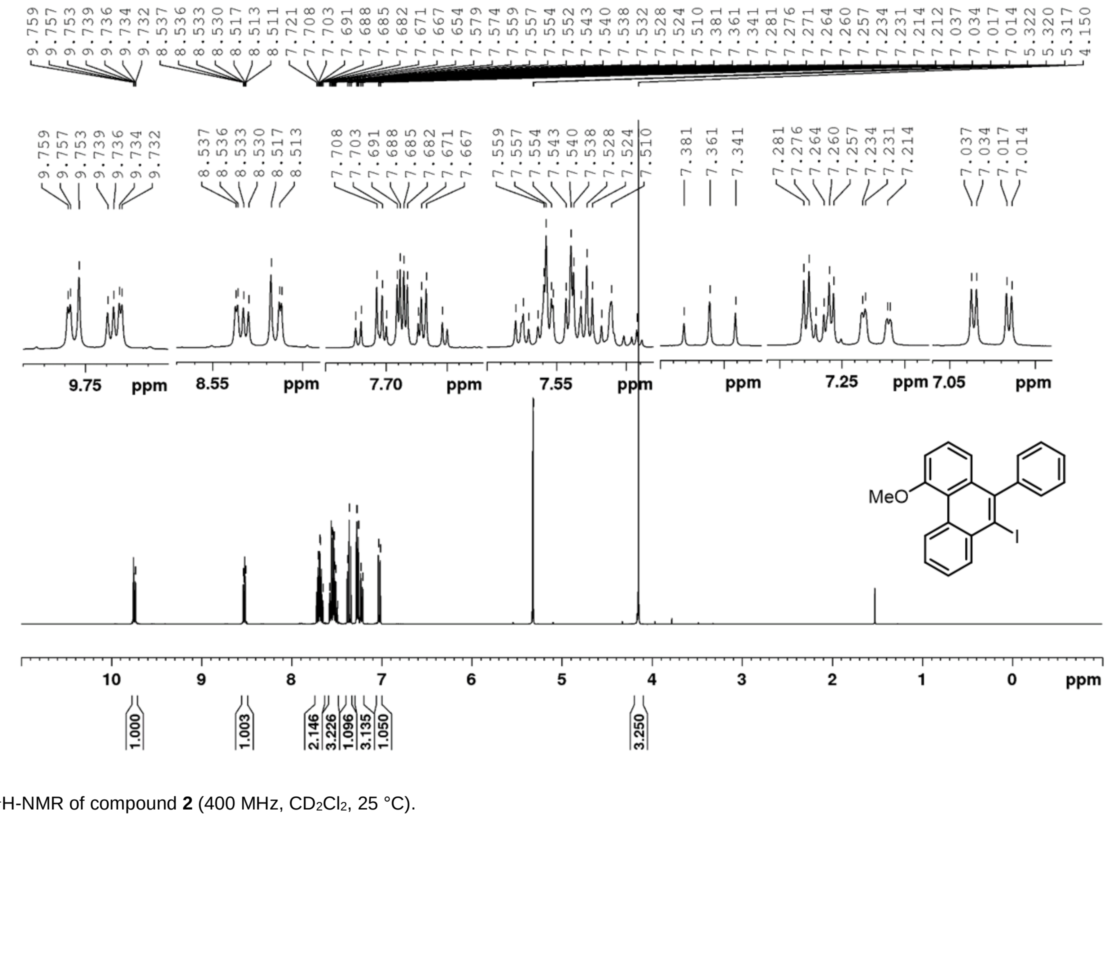

Figure S8. ^{1}H-NMR of compound **2** (400 MHz, CD_2Cl_2, 25 °C).

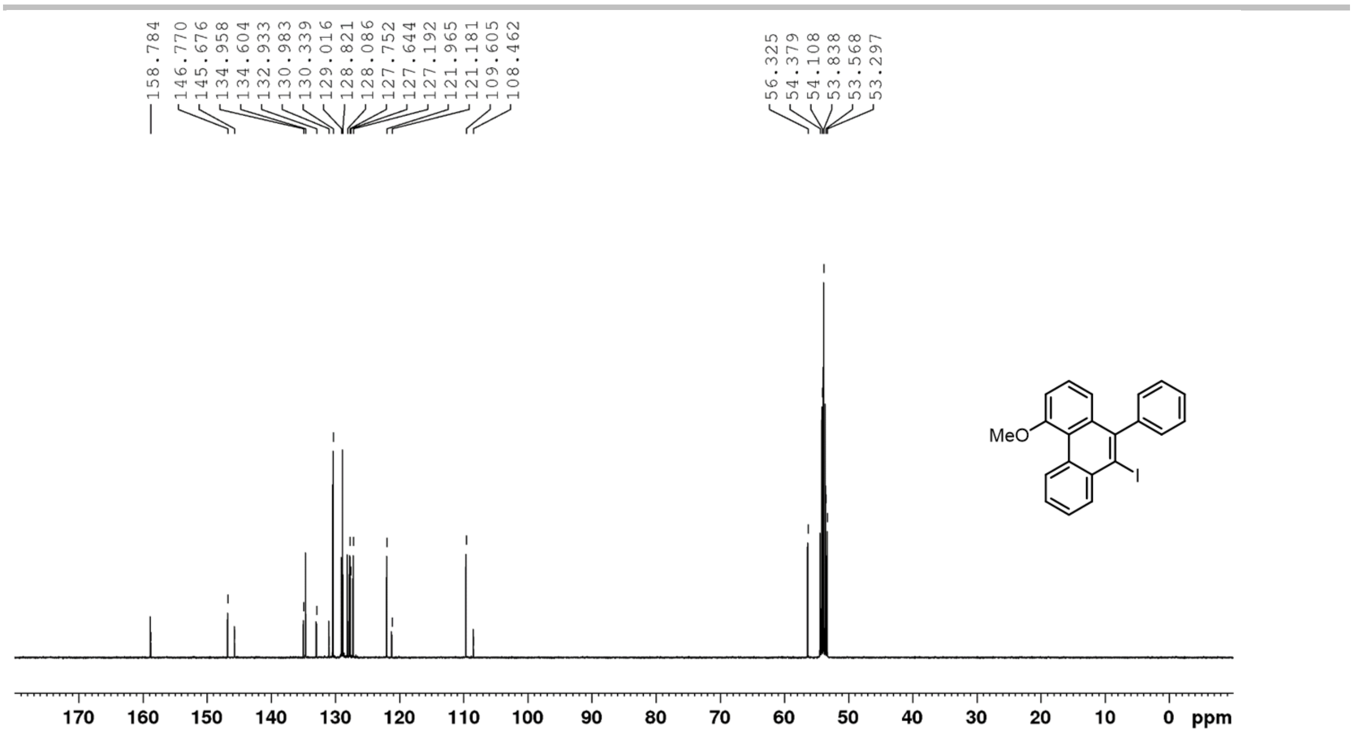

Figure S9. ^{13}C-NMR of compound **2** (100 MHz, CD_2Cl_2, 25°C).

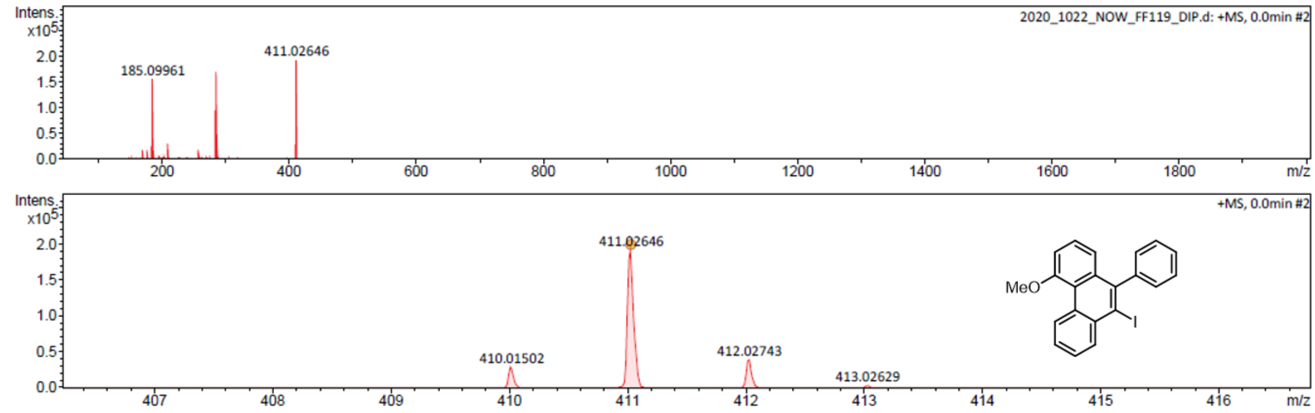

Meas. m/z	#	Ion Formula	m/z	err [ppm]	mSigma	# mSigma	Score	rdb	e⁻ Conf	N-Rule
411.02646	1	C21H16IO	411.02404	-5.89	17.9	1	100.00	13.5	even	ok

Figure S10. HRMS-(APCI-DIP) spectrum of compound **2**.

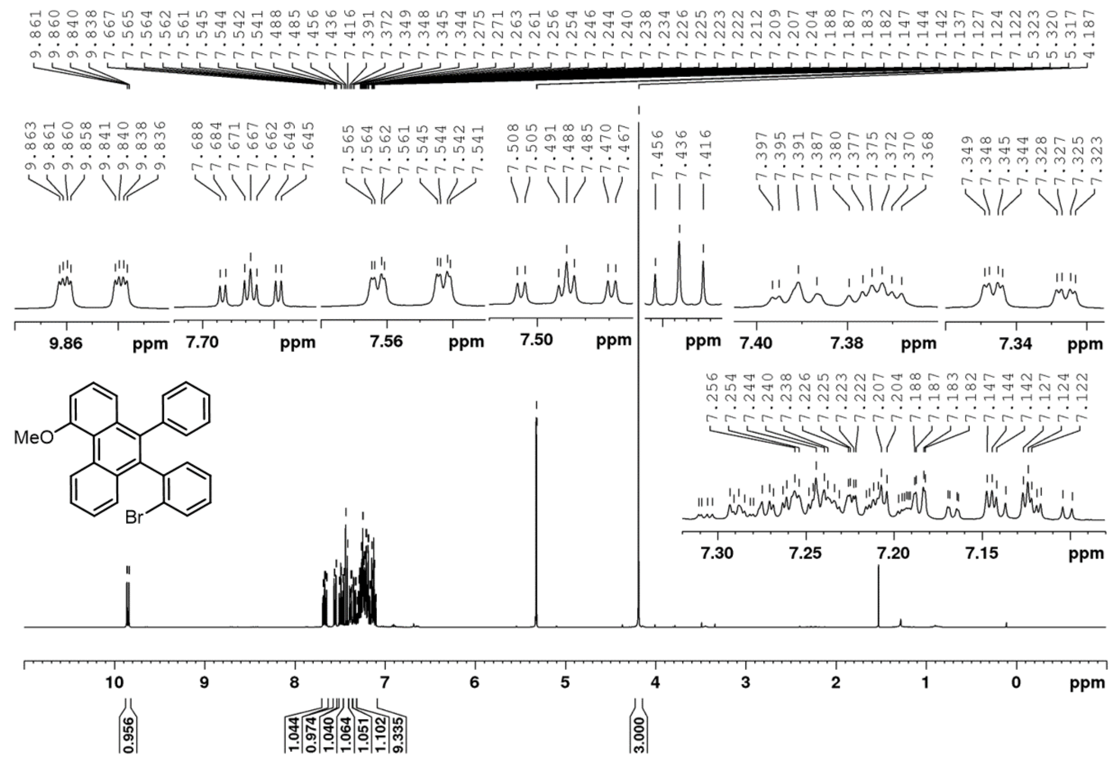

Figure S11. ^{1}H-NMR of compound **3** (400 MHz, CD_2Cl_2, 25 °C).

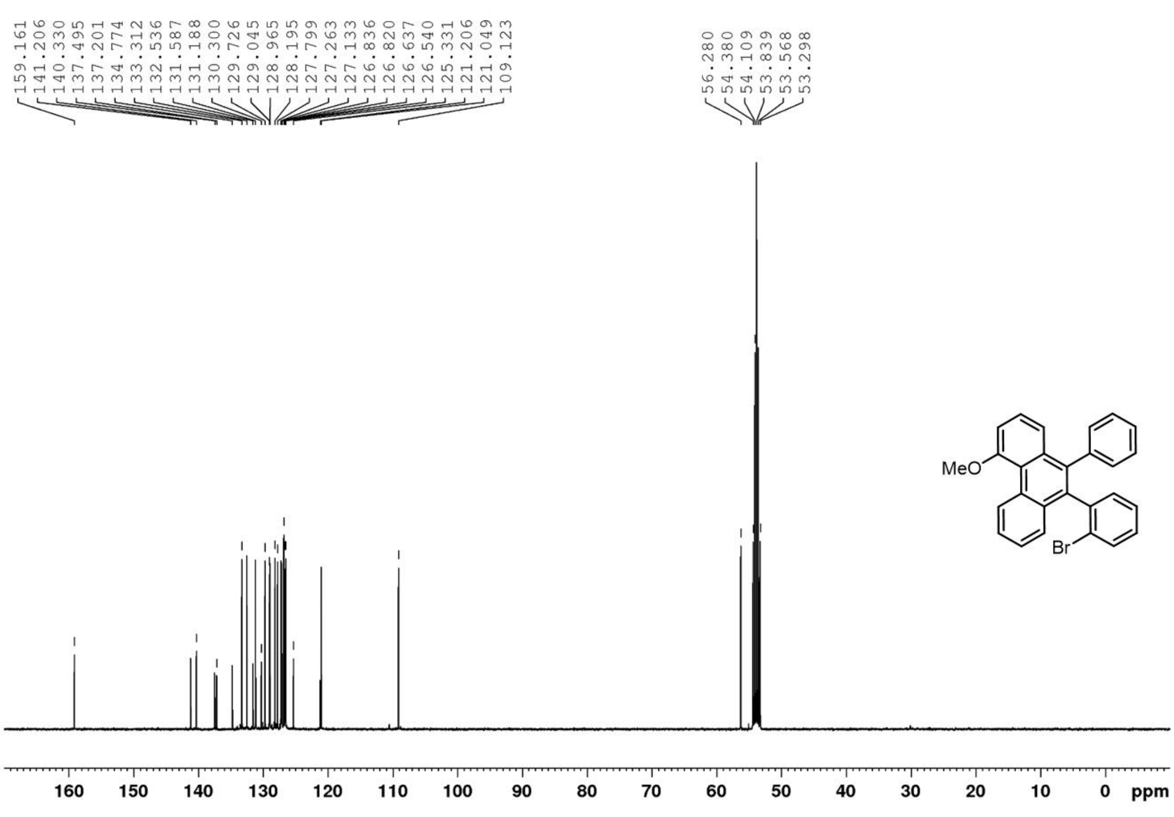

Figure S12. ^{13}C-NMR of compound **3** (100 MHz, CD_2Cl_2, 25 °C).

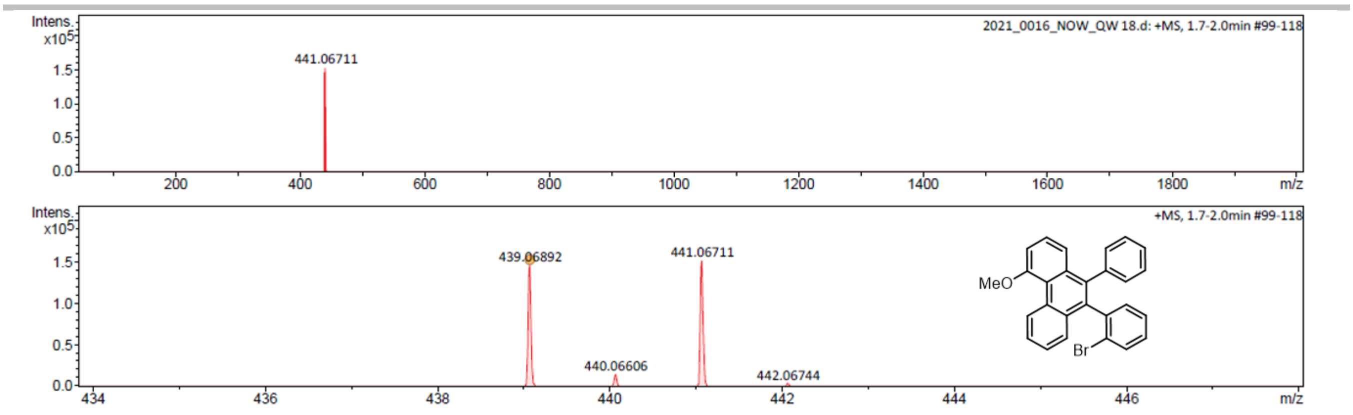

Meas. m/z	#	Ion Formula	m/z	err [ppm]	mSigma	# mSigma	Score	rdb	e¯ Conf	N-Rule
439.06892	1	C27H20BrO	439.06975	1.90	149.0	1	100.00	17.5	even	ok

Figure S13. HRMS (ESI) spectrum of compound **3**.

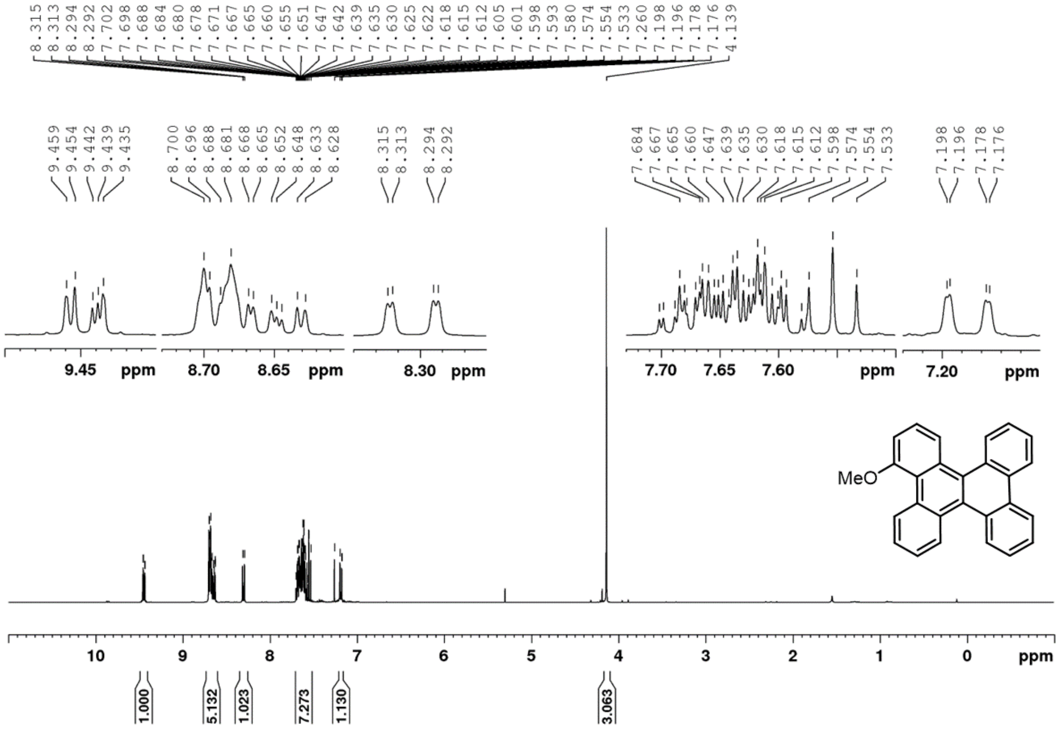

Figure S14. ^{1}H-NMR of compound **DBzC-OMe** (400 MHz, $CDCl_3$, 25 °C).

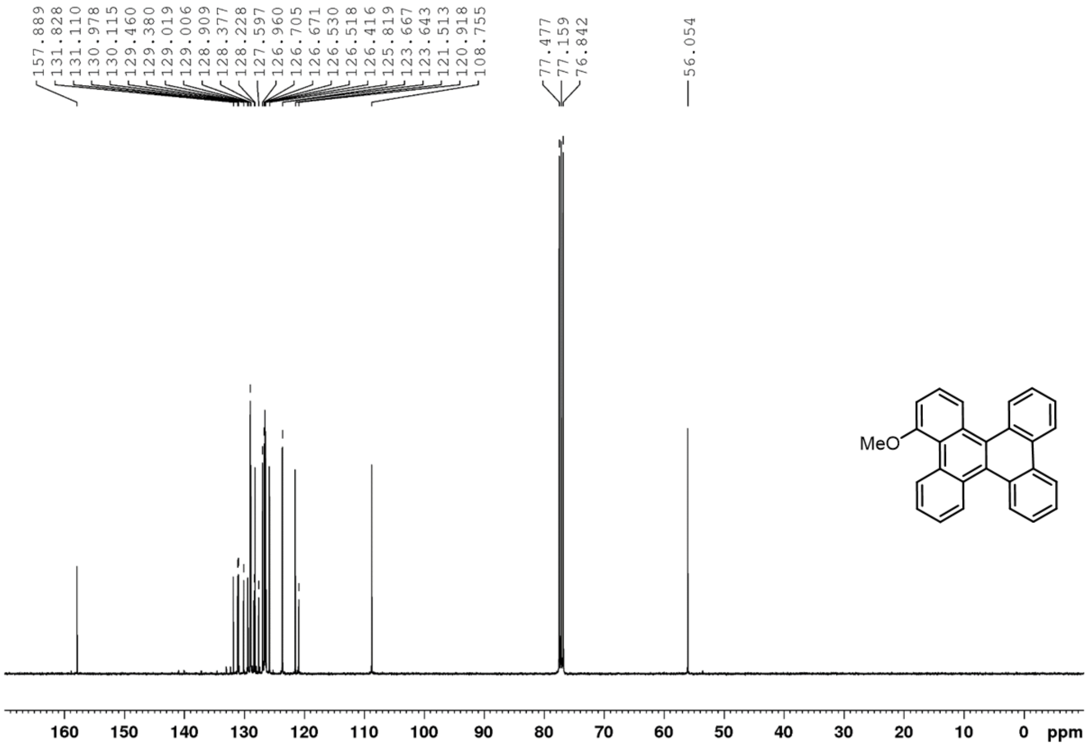

Figure S15. ^{13}C-NMR of compound **DBzC-OMe** (100 MHz, $CDCl_3$, 25 °C).

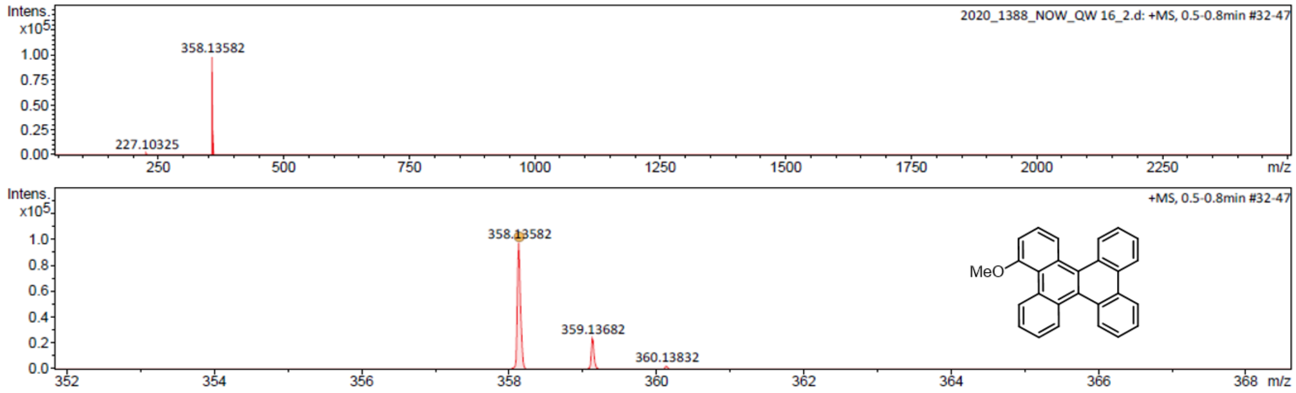

Meas. m/z	#	Ion Formula	m/z	err [ppm]	mSigma	# mSigma	Score	rdb	e¯ Conf	N-Rule
358.13582	1	C27H18O	358.13522	-1.70	29.3	1	100.00	19.0	odd	ok

Figure S16. HRMS (ESI) spectrum of compound **DBzC-OMe.**

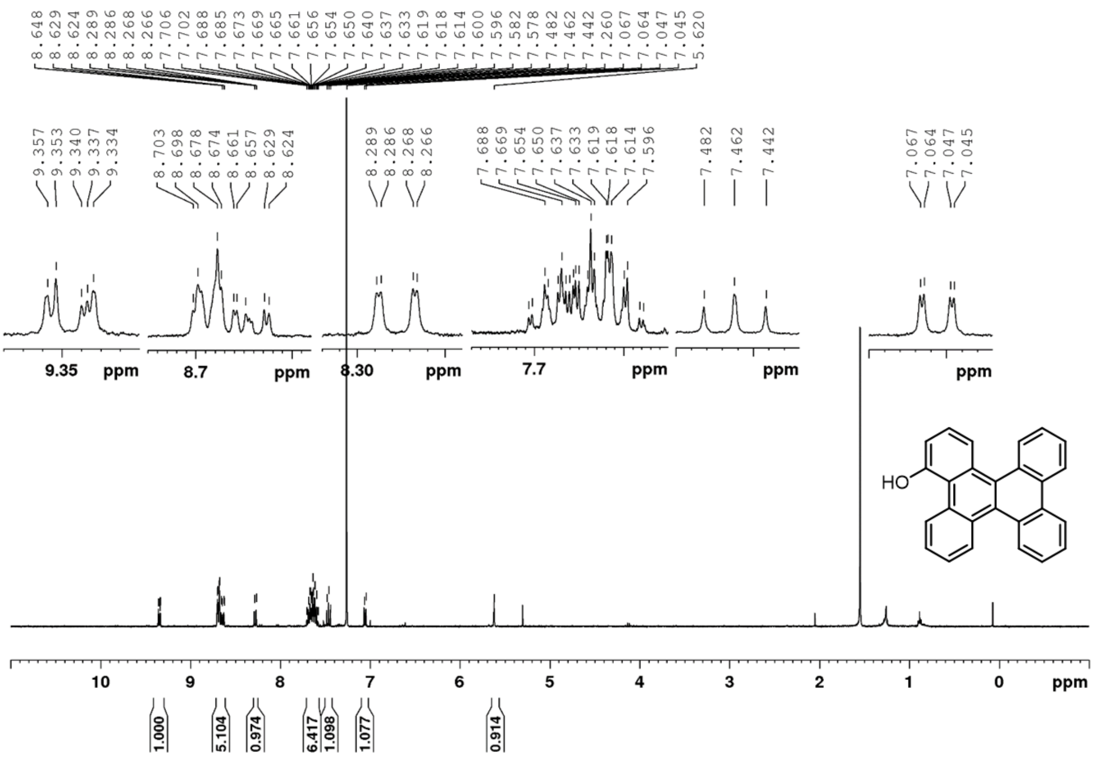

Figure S17. ^{1}H-NMR of compound **DBzC-OH** (400 MHz, $CDCl_3$, 25 °C).

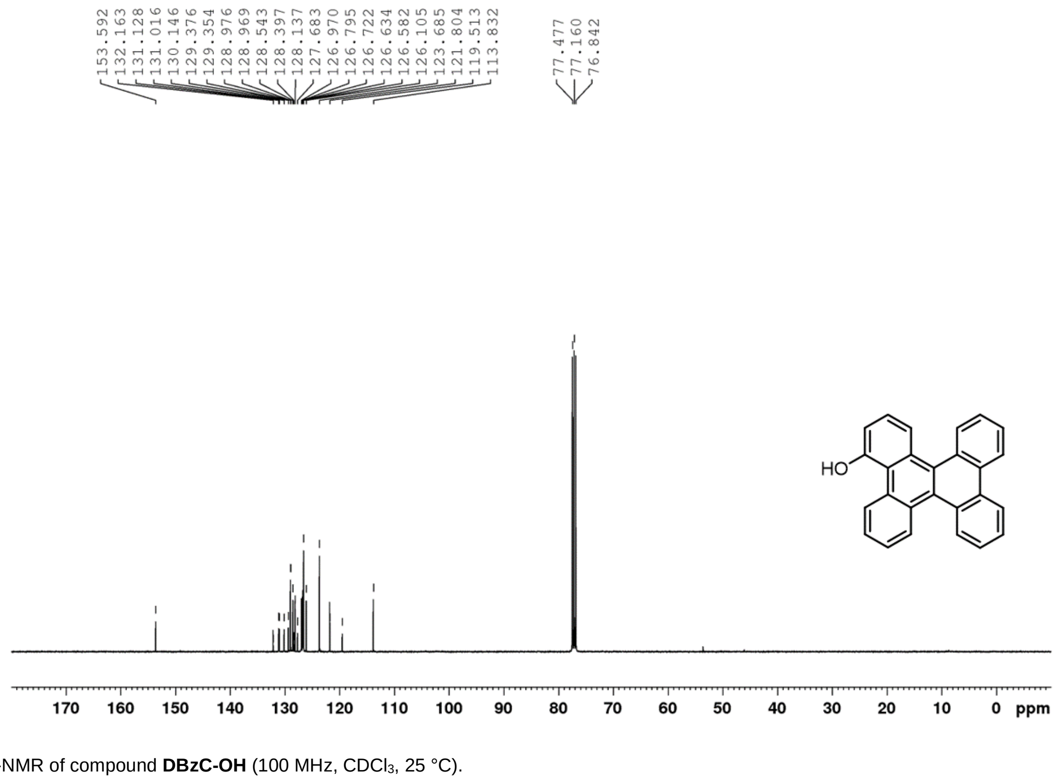

Figure S18. ^{13}C-NMR of compound **DBzC-OH** (100 MHz, $CDCl_3$, 25 °C).

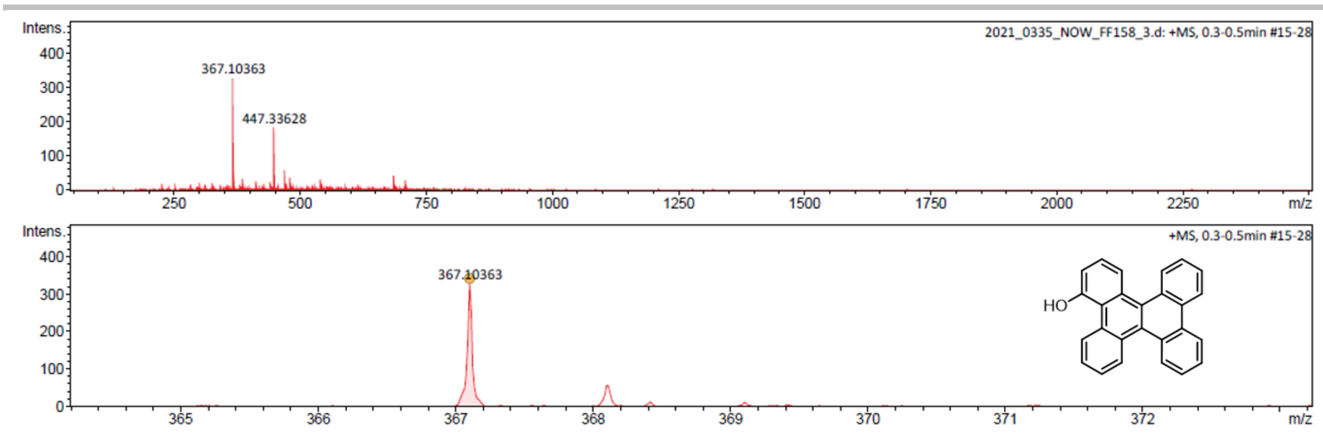

Meas. m/z	#	Ion Formula	m/z	err [ppm]	mSigma	# mSigma	Score	rdb	e⁻ Conf	N-Rule
367.10363	1	C26H16NaO	367.10988	17.02	n.a.	1	100.00	18.5	even	ok

Figure S19. HRMS (ESI) spectrum of compound **DBzC-OH**.

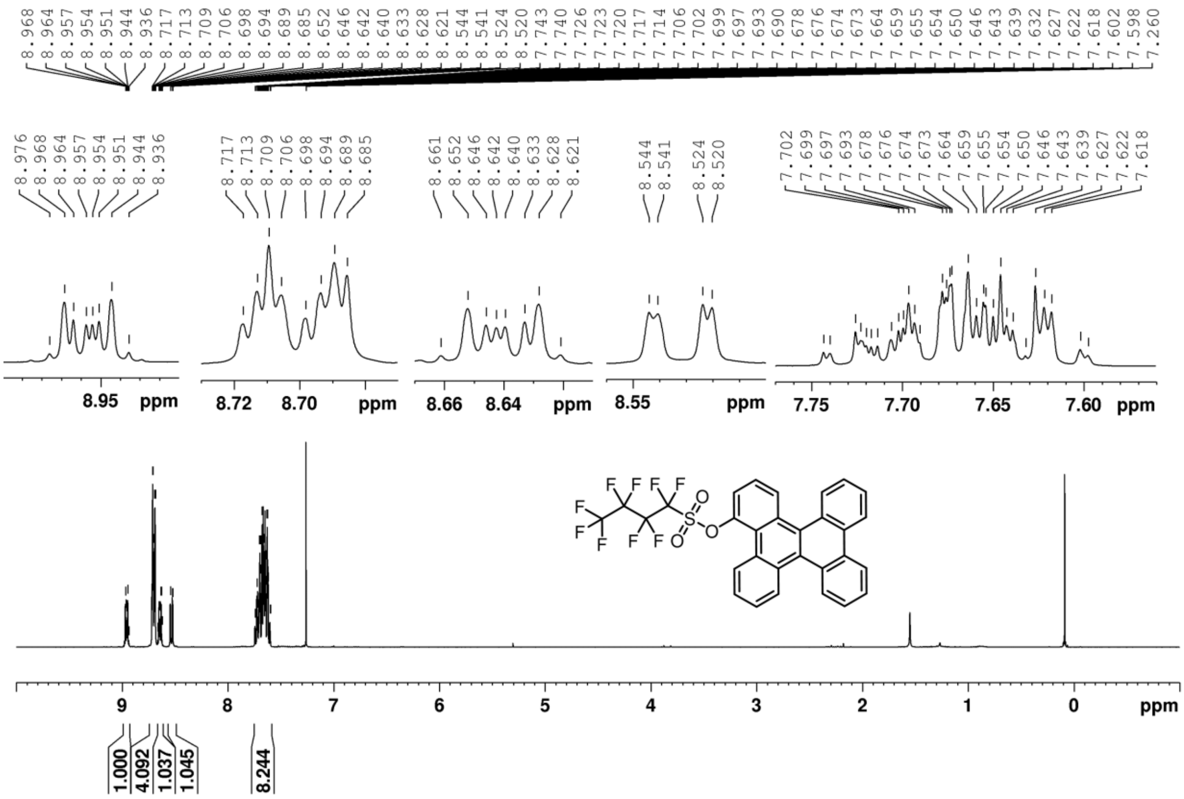

Figure S20. ^{1}H-NMR of compound **DBzC-ONf** (400 MHz, $CDCl_3$, 25 °C).

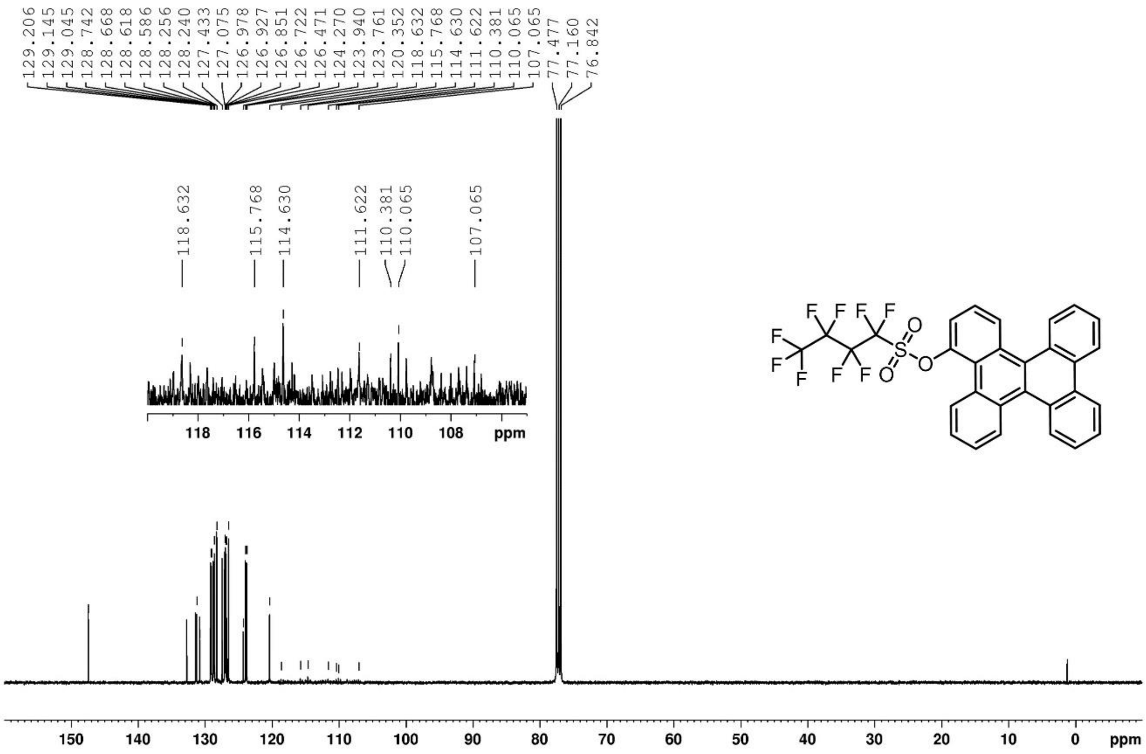

Figure S21. ^{13}C-NMR of compound **DBzC-ONf** (100 MHz, $CDCl_3$, 25 °C).

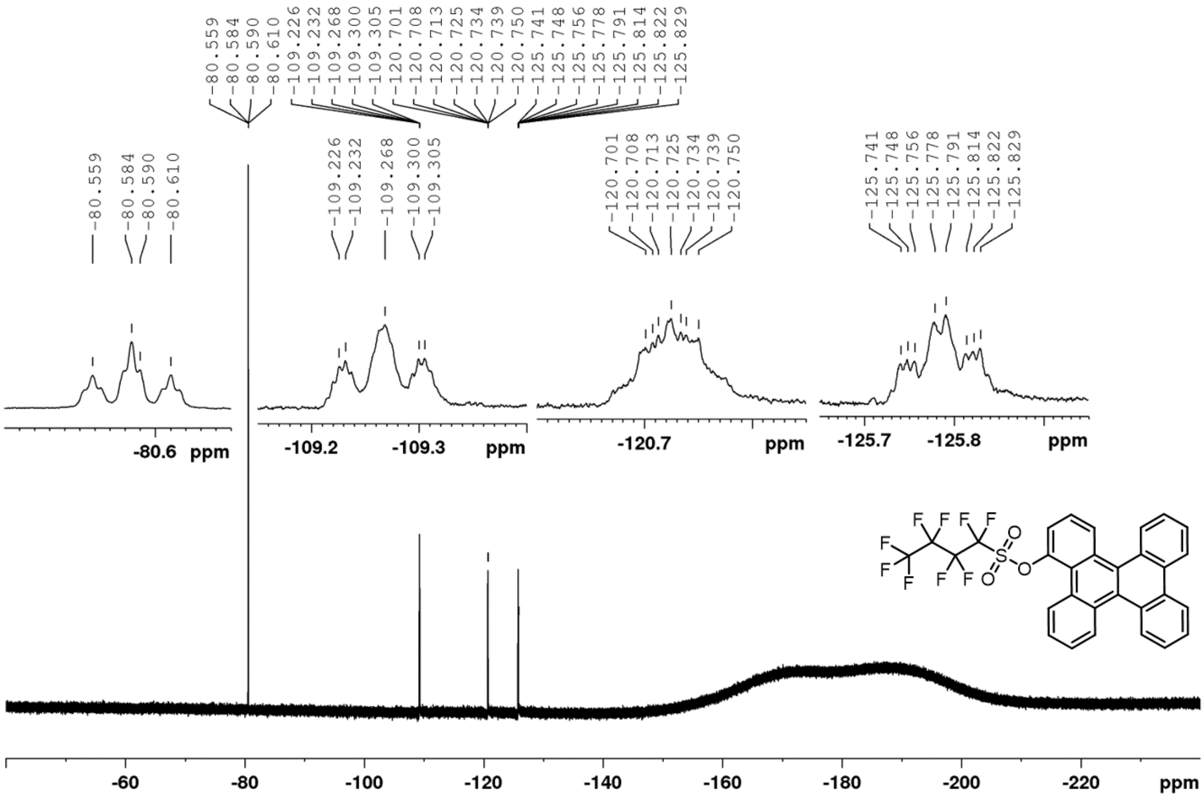

Figure S22. ^{19}F-NMR of compound **DBzC-ONf** (376 MHz, $CDCl_3$, 25 °C).

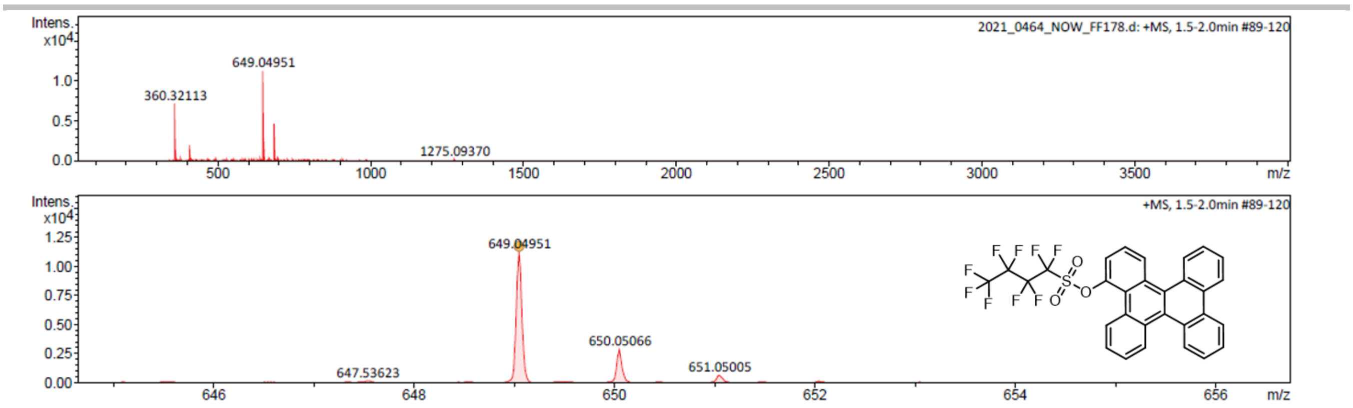

Meas. m/z	#	Ion Formula	m/z	err [ppm]	mSigma	# mSigma	Score	rdb	e⁻ Conf	N-Rule
649.04951	1	C30H15F9NaO3S	649.04904	-0.73	48.8	1	100.00	18.5	even	ok

Figure S23. HRMS (ESI) spectrum of compound **DBzC-ONf**.

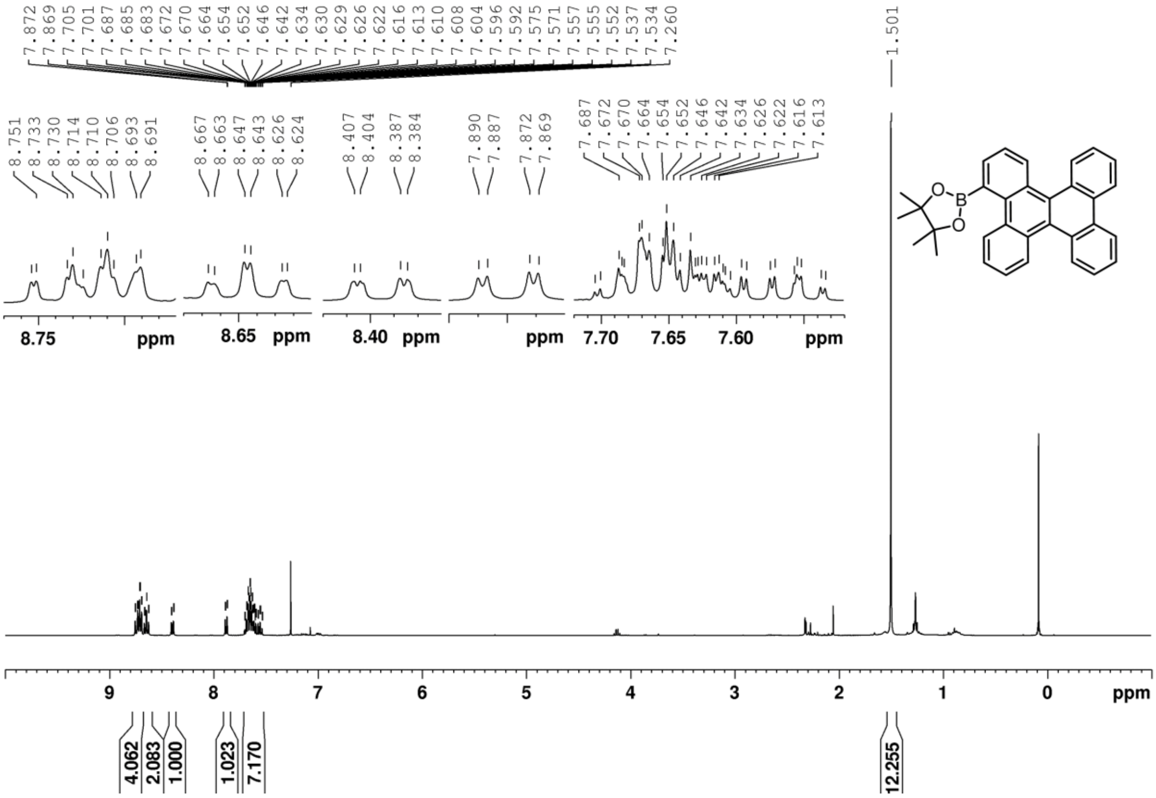

Figure S24. ^{1}H-NMR of compound **DBzC-Bpin** (400 MHz, $CDCl_3$, 25 °C).

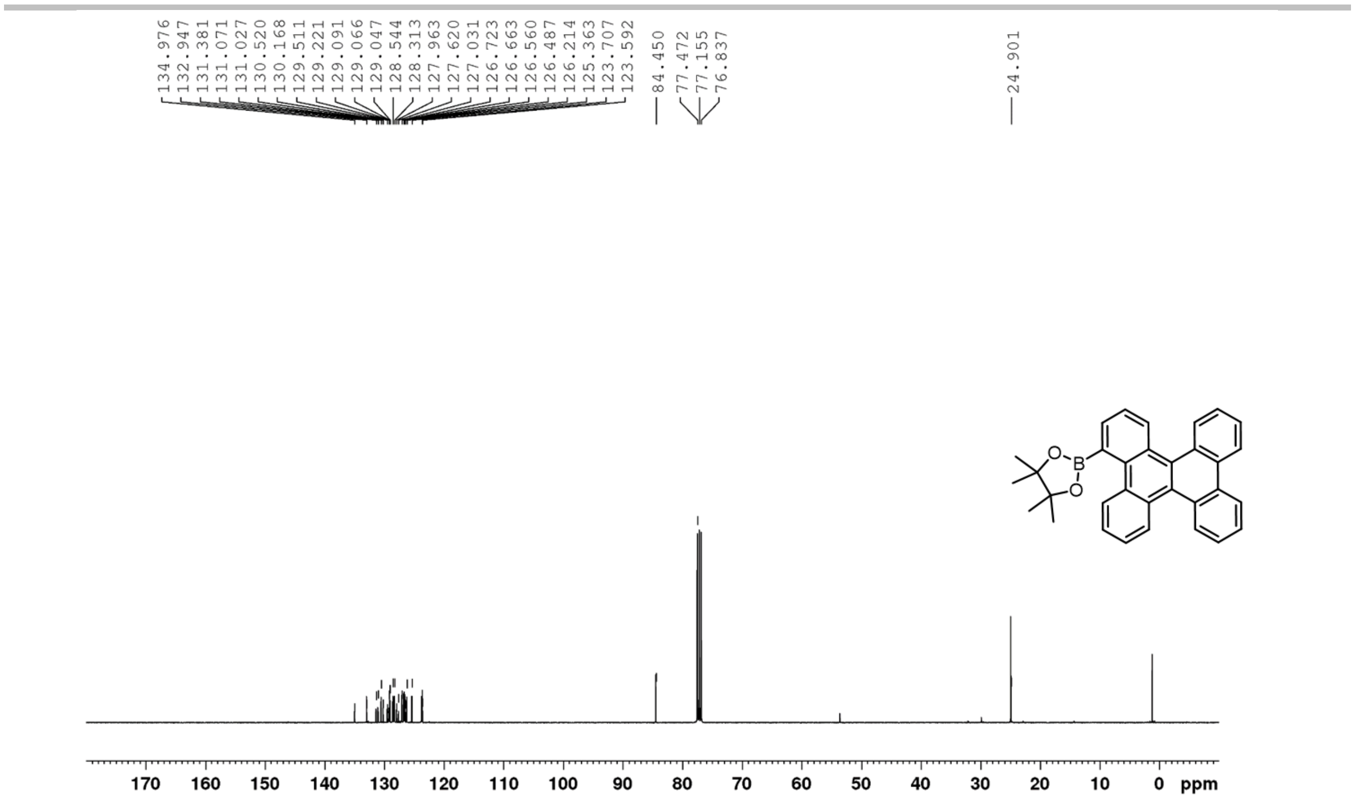

Figure S25. ^{13}C-NMR of compound **DBzC-Bpin** (100 MHz, $CDCl_3$, 25 °C).

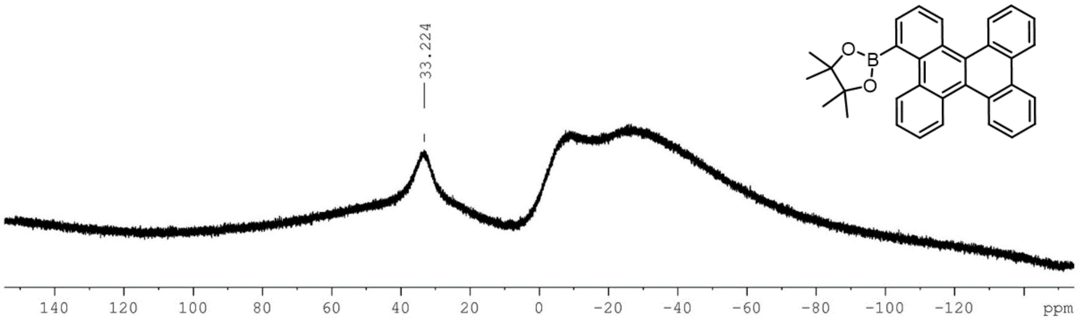

Figure S26. ^{11}B-NMR of compound **DBzC-Bpin** (128 MHz, $CDCl_3$, 25 °C).

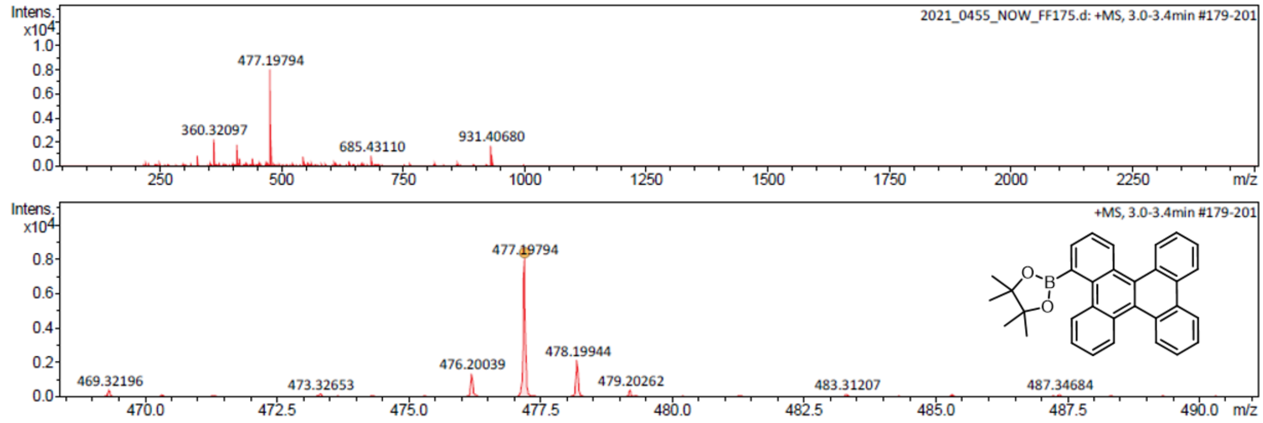

Meas. m/z	#	Ion Formula	m/z	err [ppm]	mSigma	# mSigma	Score	rdb	e⁻ Conf	N-Rule
477.19794	1	C32H27BNaO2	477.19963	4.72	46.2	1	100.00	19.5	even	ok

Figure S27. HRMS (ESI) spectrum of compound **DBzC-Bpin.**

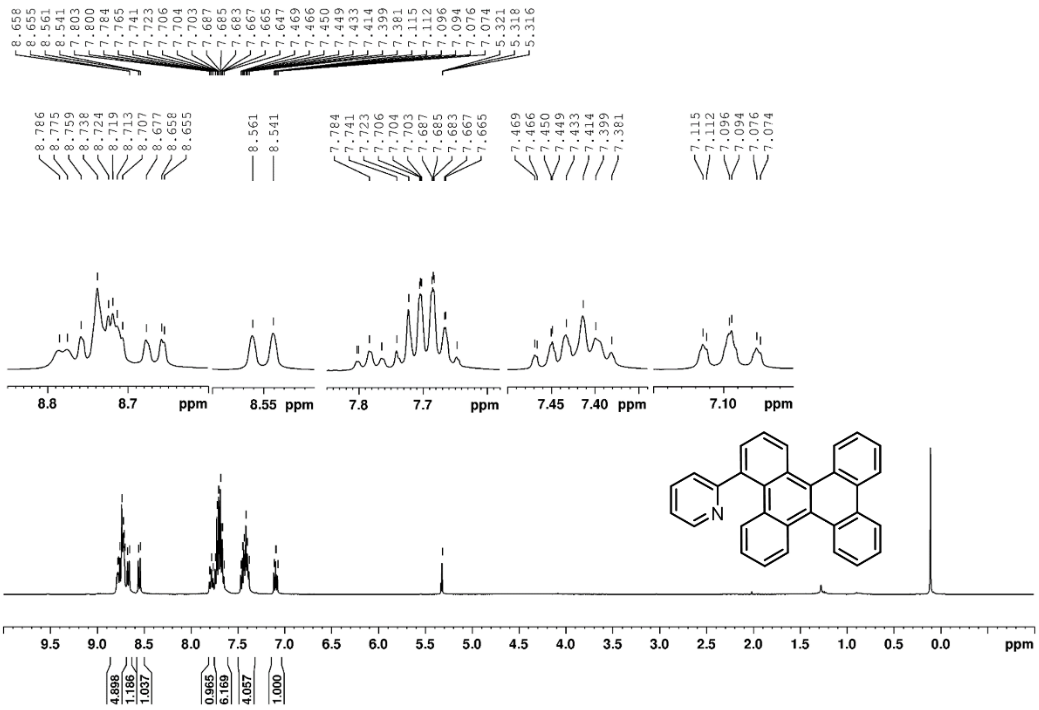

Figure S28. ^{1}H-NMR of compound **BA1** (400 MHz, CD_2Cl_2, 25 °C).

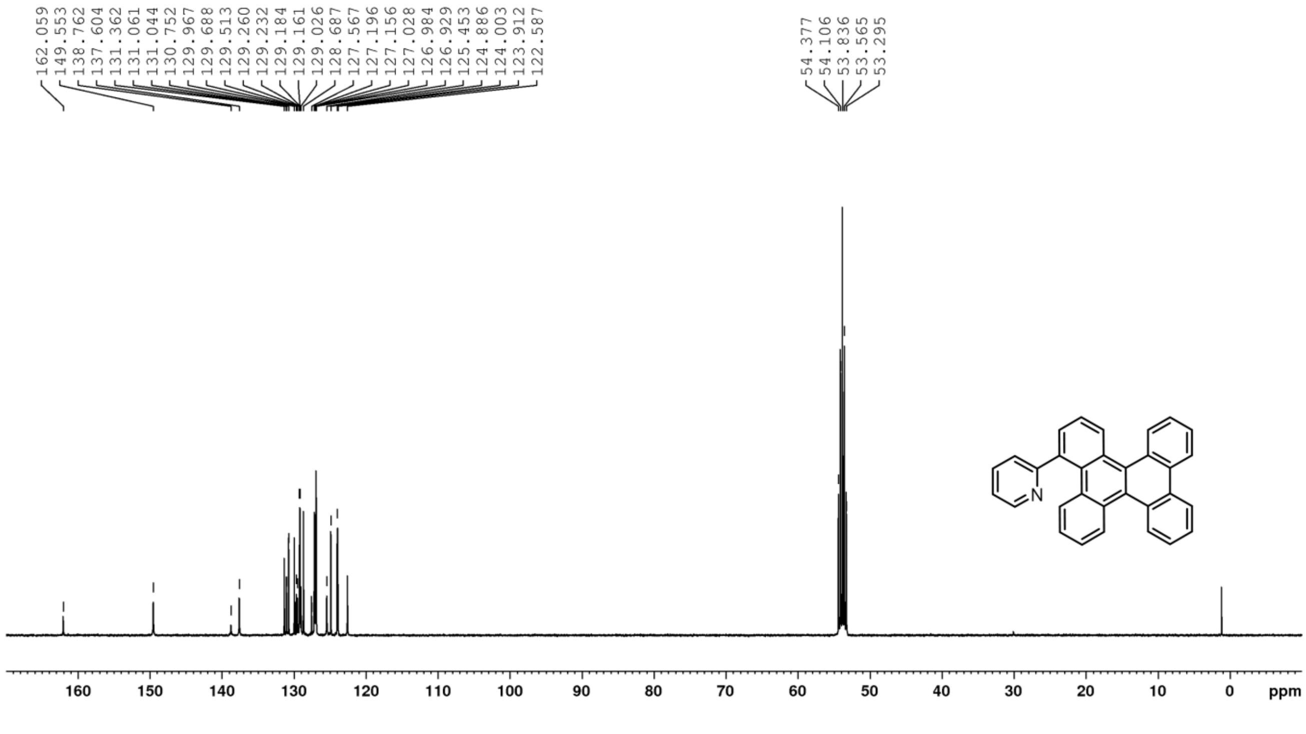

Figure S29. ^{13}C-NMR of compound **BA1** (400 MHz, CD_2Cl_2, 25 °C).

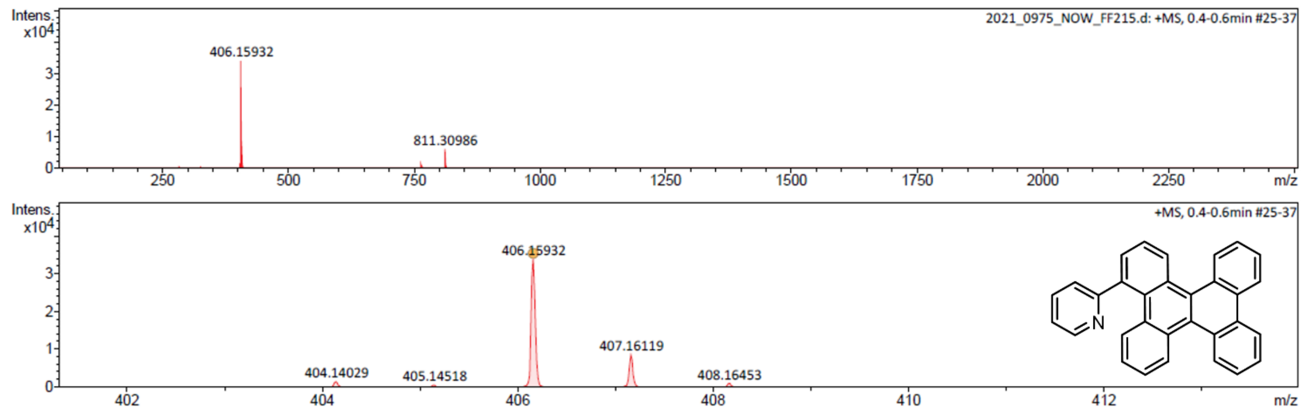

Meas. m/z	#	Ion Formula	m/z	err [ppm]	mSigma	# mSigma	Score	rdb	e⁻ Conf	N-Rule
406.15932	1	C31H20N	406.15903	-0.72	53.7	1	100.00	22.5	even	ok

Figure S30. HRMS (ESI) spectrum of compound **BA1**.

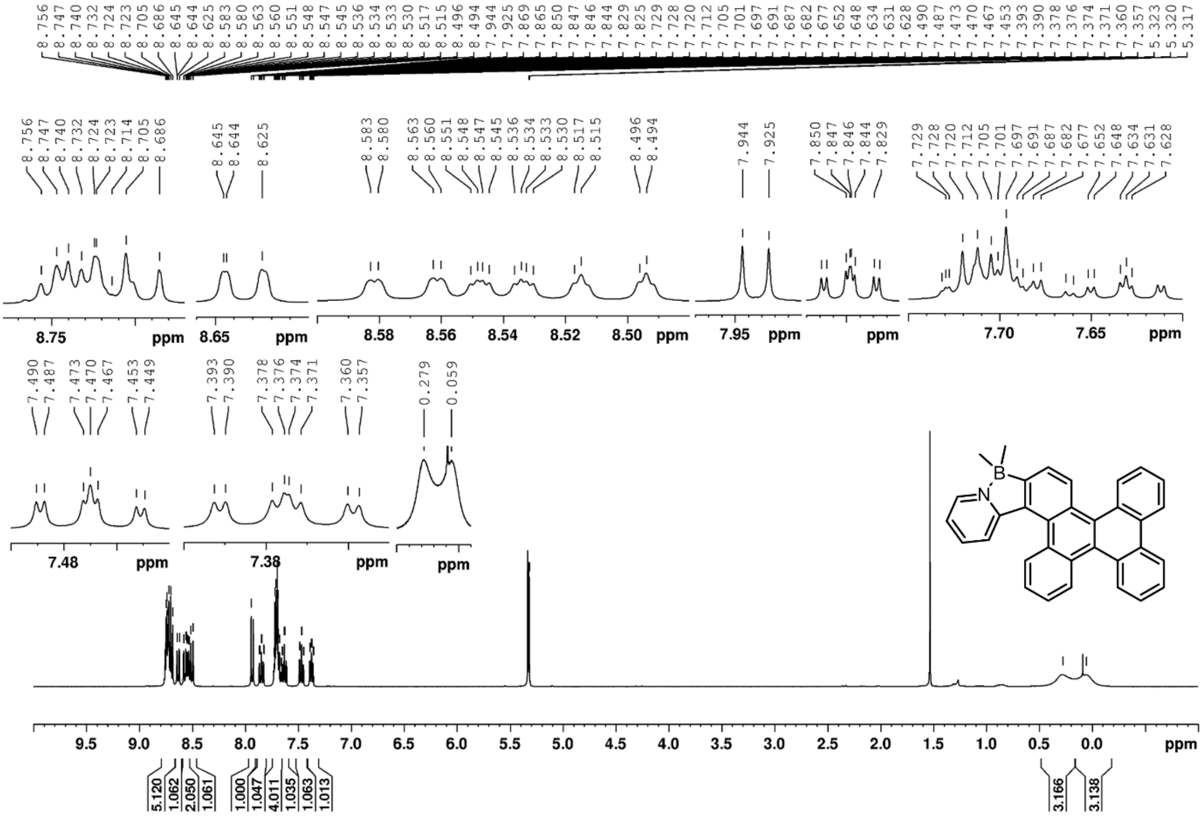

Figure S31. ^{1}H-NMR of compound **EH1** (400 MHz, CD_2Cl_2, 25 °C).

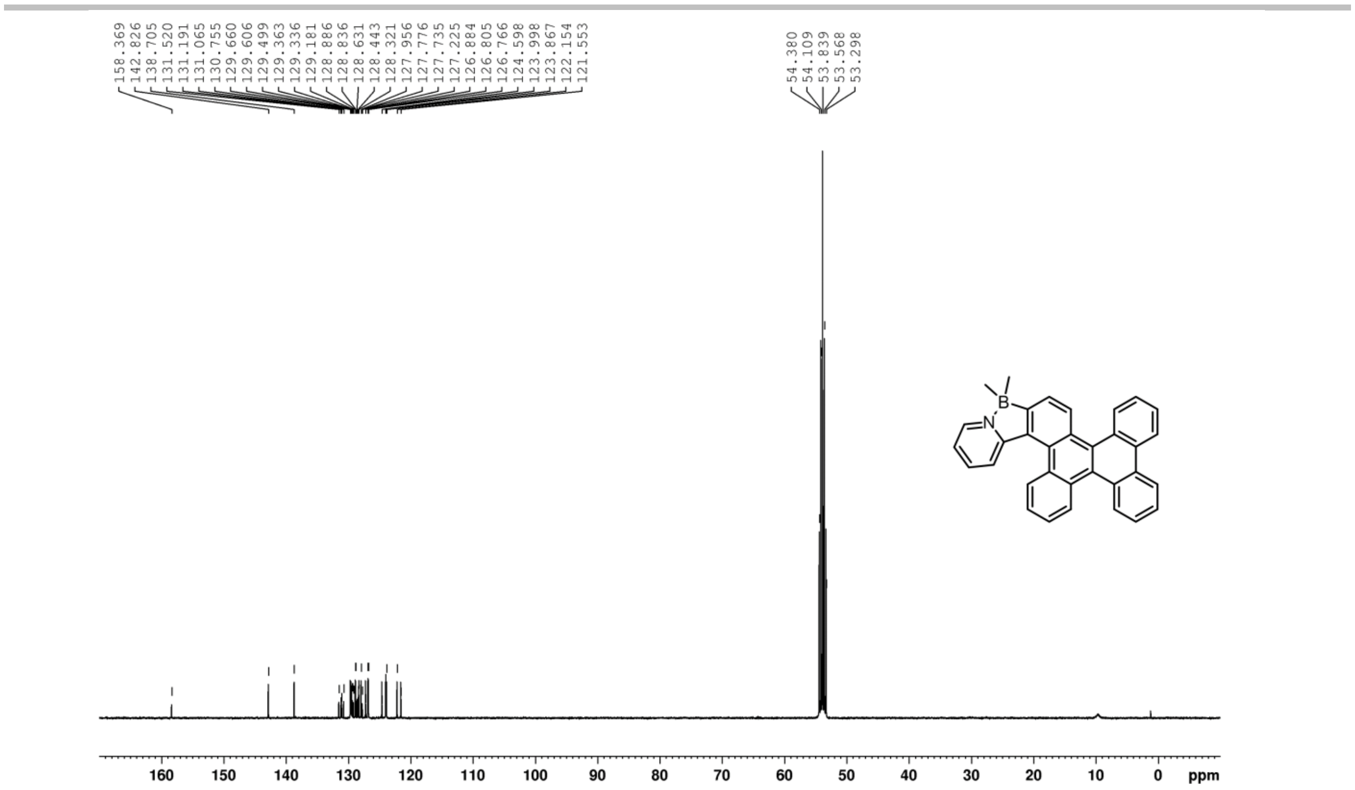

Figure S32. ^{13}C-NMR of compound **EH1** (100 MHz, CD_2Cl_2, 25 °C).

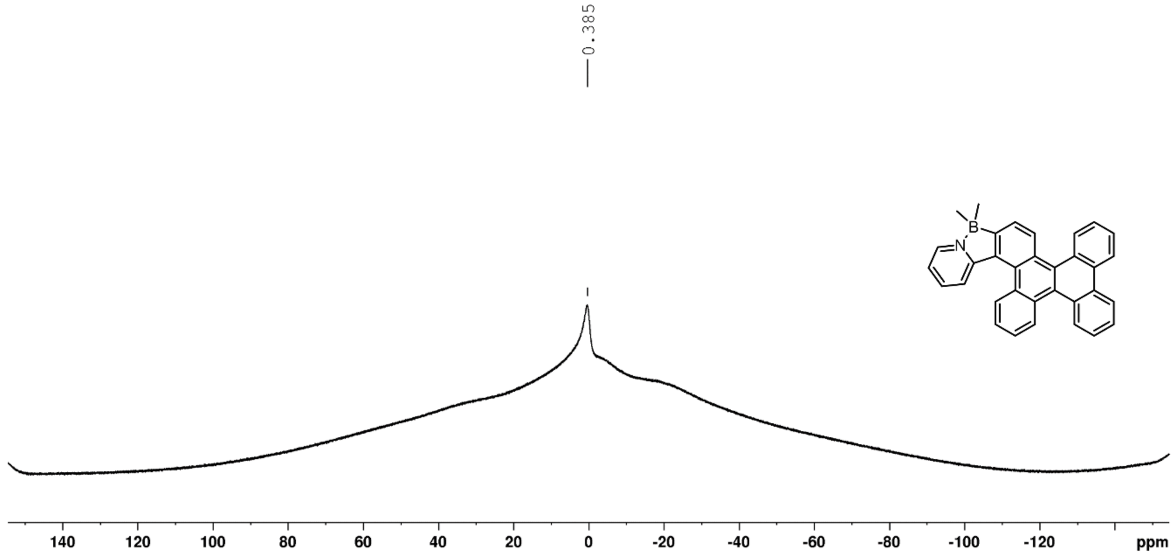

Figure S33. ^{11}B-NMR of compound **EH1** (128 MHz, CD_2Cl_2, 25 °C).

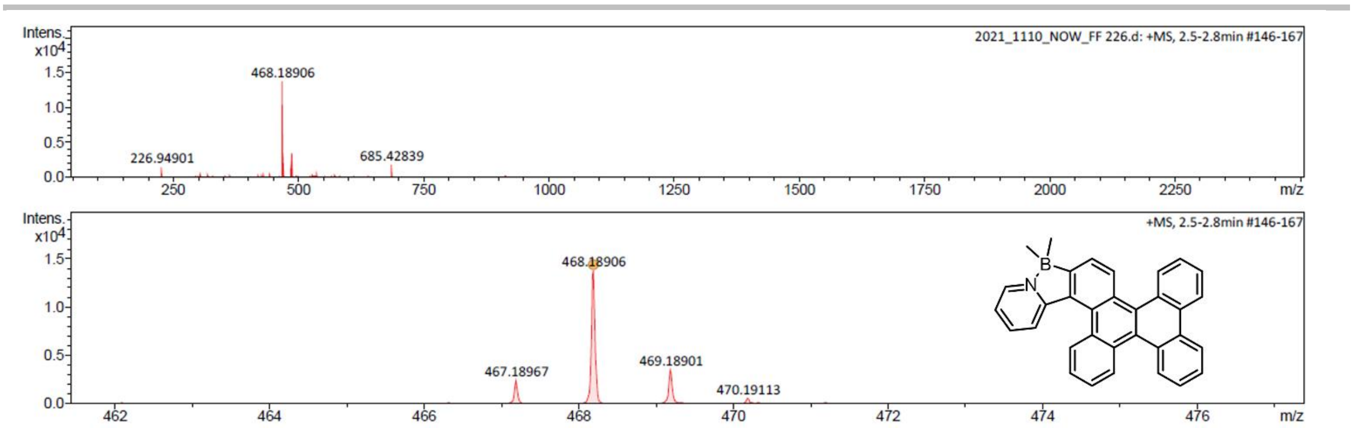

Meas. m/z	#	Ion Formula	m/z	err [ppm]	mSigma	# mSigma	Score	rdb	e⁻ Conf	N-Rule
468.18906	1	C33H24BNNa	468.18940	1.96	54.4	1	100.00	22.5	even	ok

Figure S34. HRMS (ESI) spectrum of compound **EH1**.

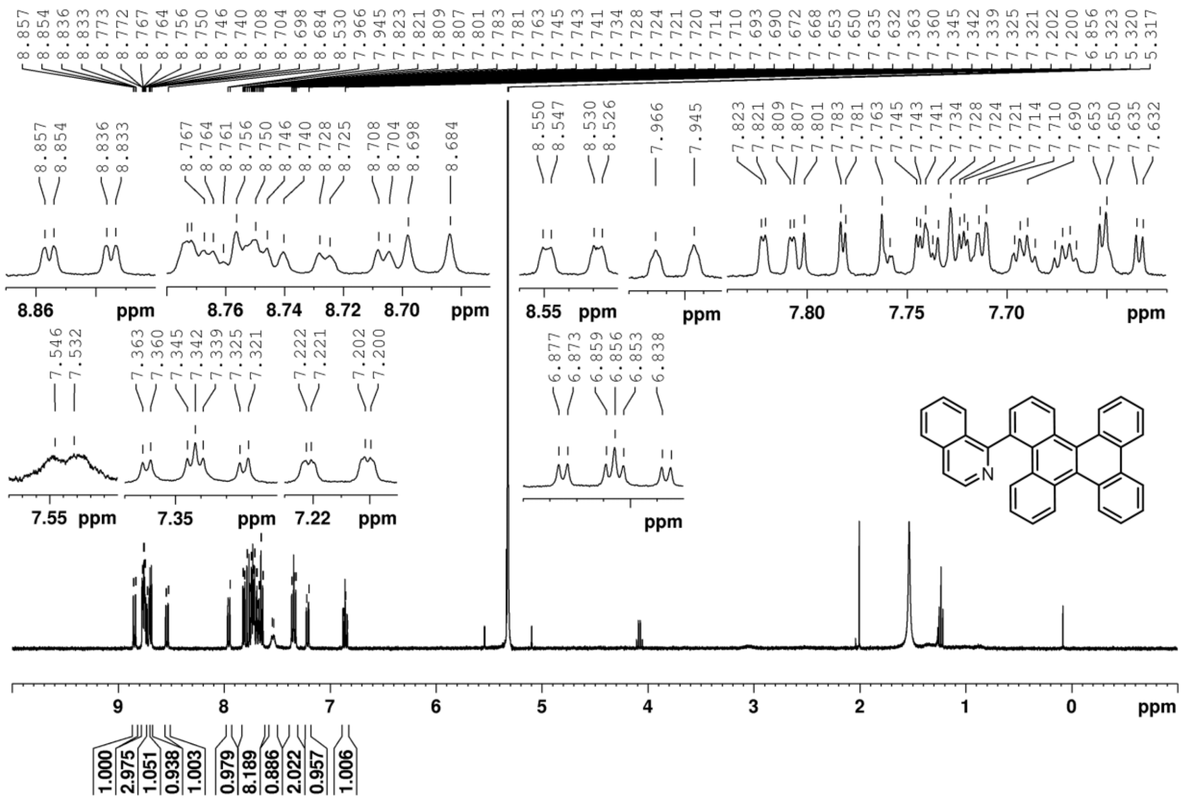

Figure S35. ^{1}H-NMR of compound **BA2** (400 MHz, CD_2Cl_2, 25 °C).

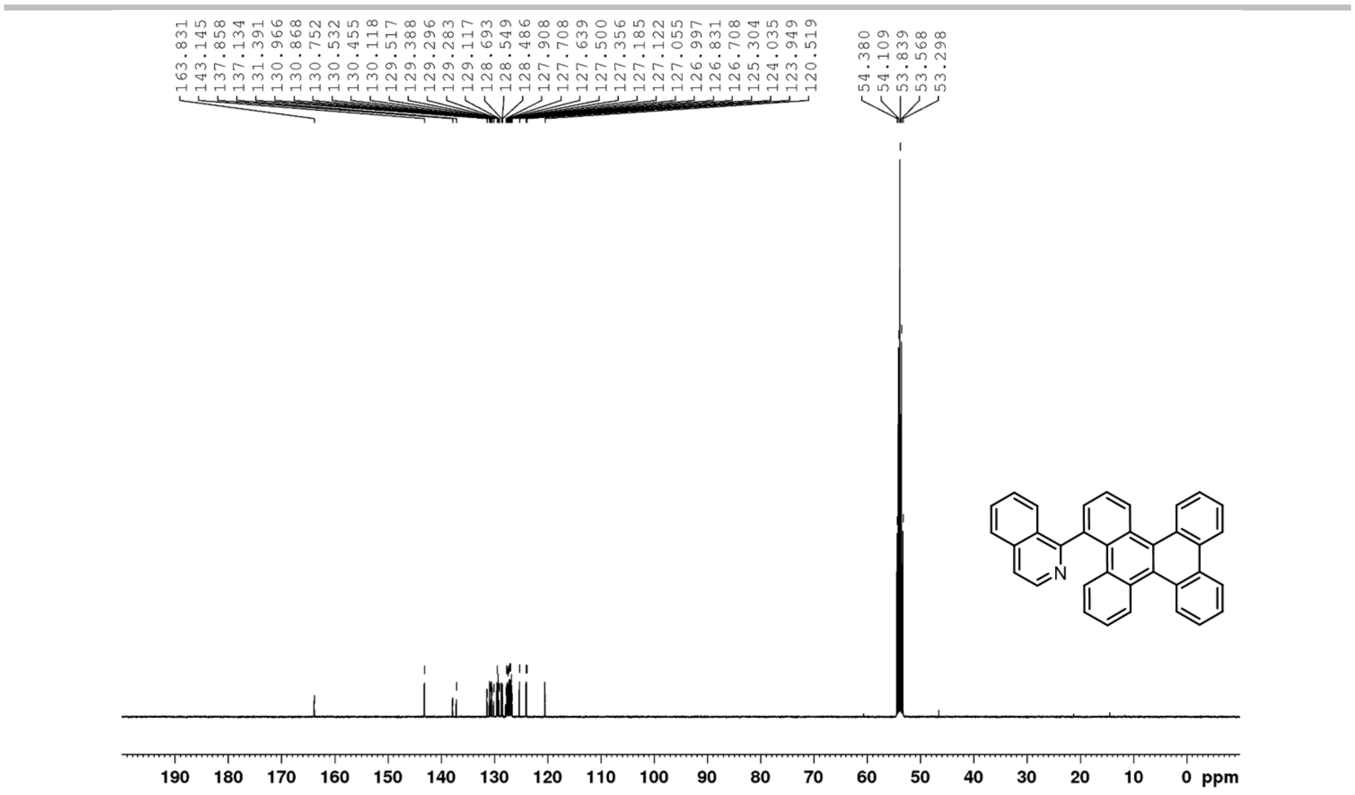

Figure S36. ^{13}C-NMR of compound **BA2** (100 MHz, CD_2Cl_2, 25 °C).

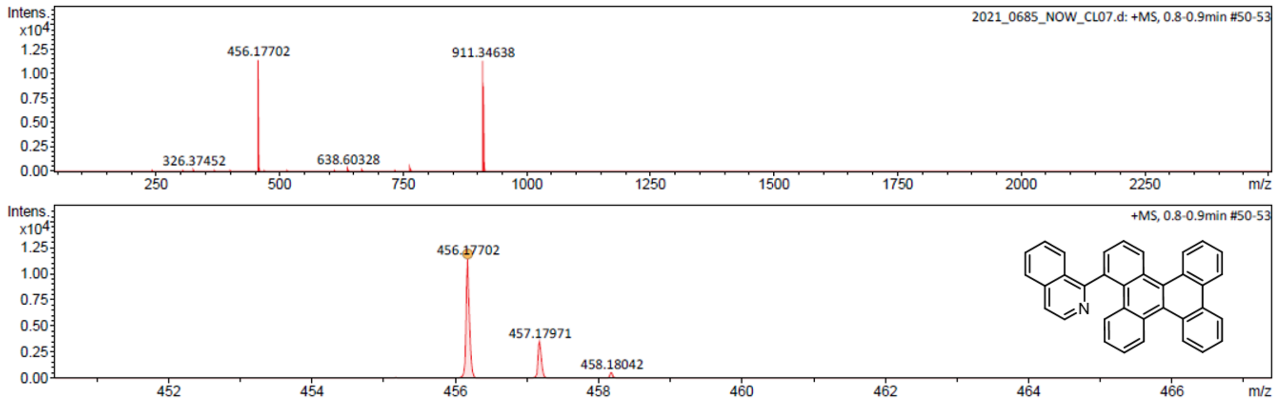

Meas. m/z	#	Ion Formula	m/z	err [ppm]	mSigma	# mSigma	Score	rdb	e⁻ Conf	N-Rule
456.17702	1	C35H22N	456.17468	-5.13	40.9	1	100.00	25.5	even	ok

Figure S37. HRMS (ESI) spectrum of compound **BA2.**

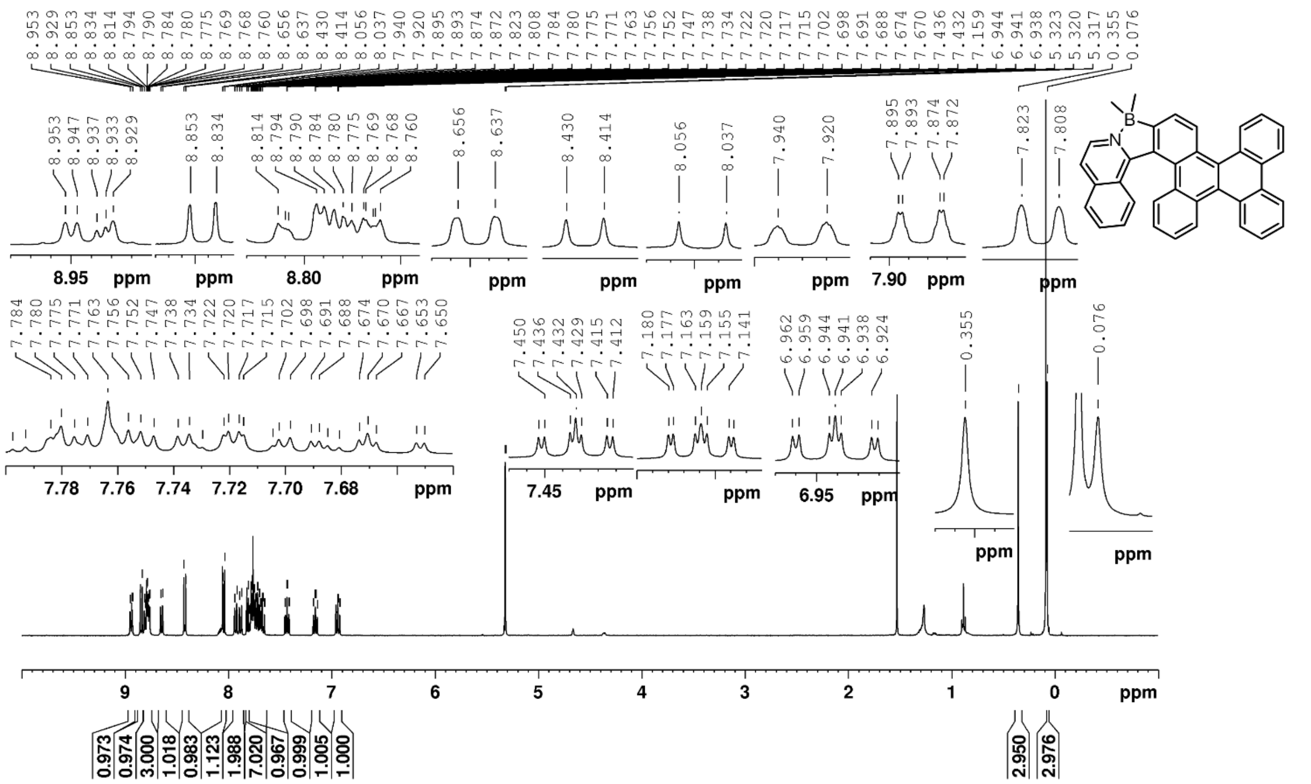

Figure S38. ^{1}H-NMR of compound **EH2** (400 MHz, CD_2Cl_2, 25 °C).

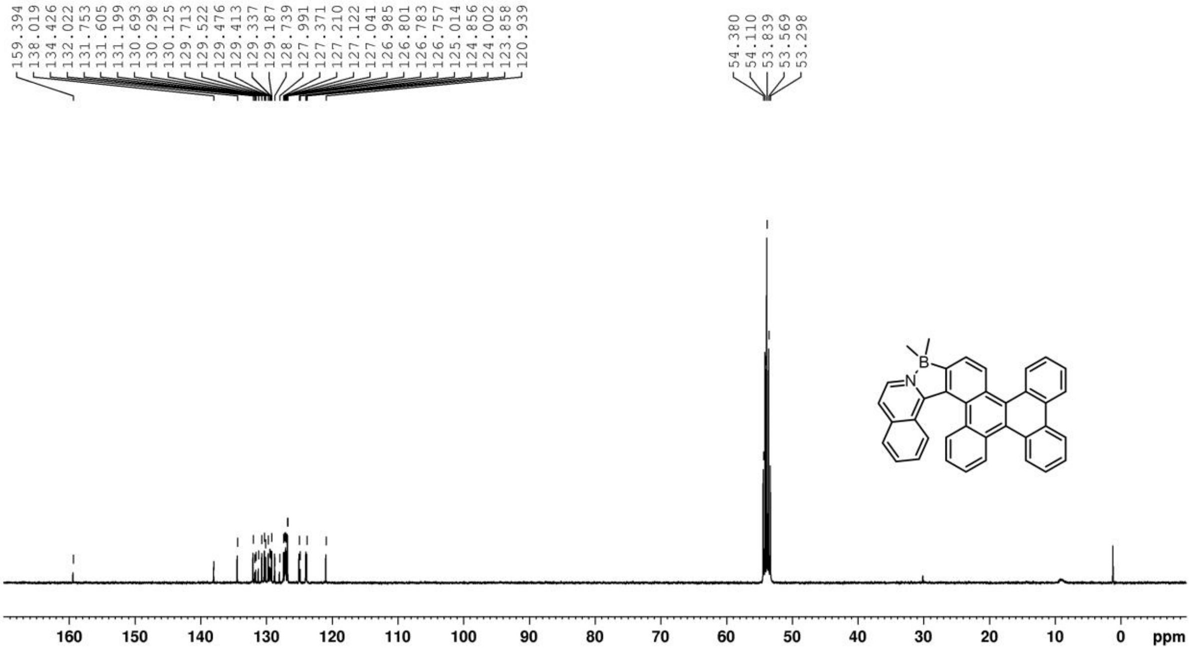

Figure S39. ^{13}C-NMR of compound **EH2** (100 MHz, CD_2Cl_2, 25 °C).

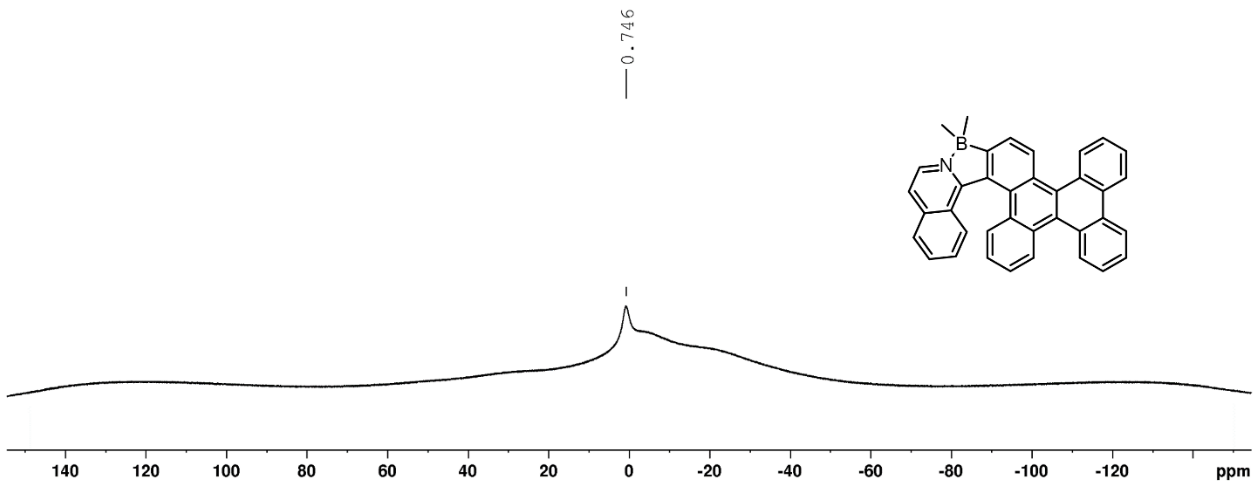

Figure S40. ^{11}B-NMR of compound **EH2** (128 MHz, CD_2Cl_2, 25 °C).

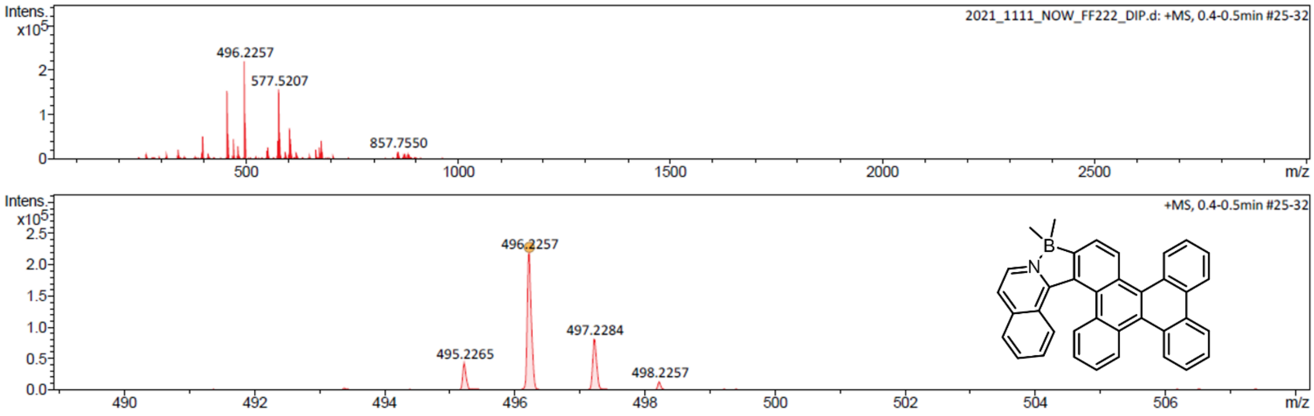

Meas. m/z	#	Ion Formula	m/z	err [ppm]	mSigma	# mSigma	Score	rdb	e⁻ Conf	N-Rule
496.2257	1	C37H27BN	496.2231	-3.9	14.5	1	100.00	25.5	even	ok

Figure S41. HRMS (APCI-DIP) spectrum of compound **EH2.**

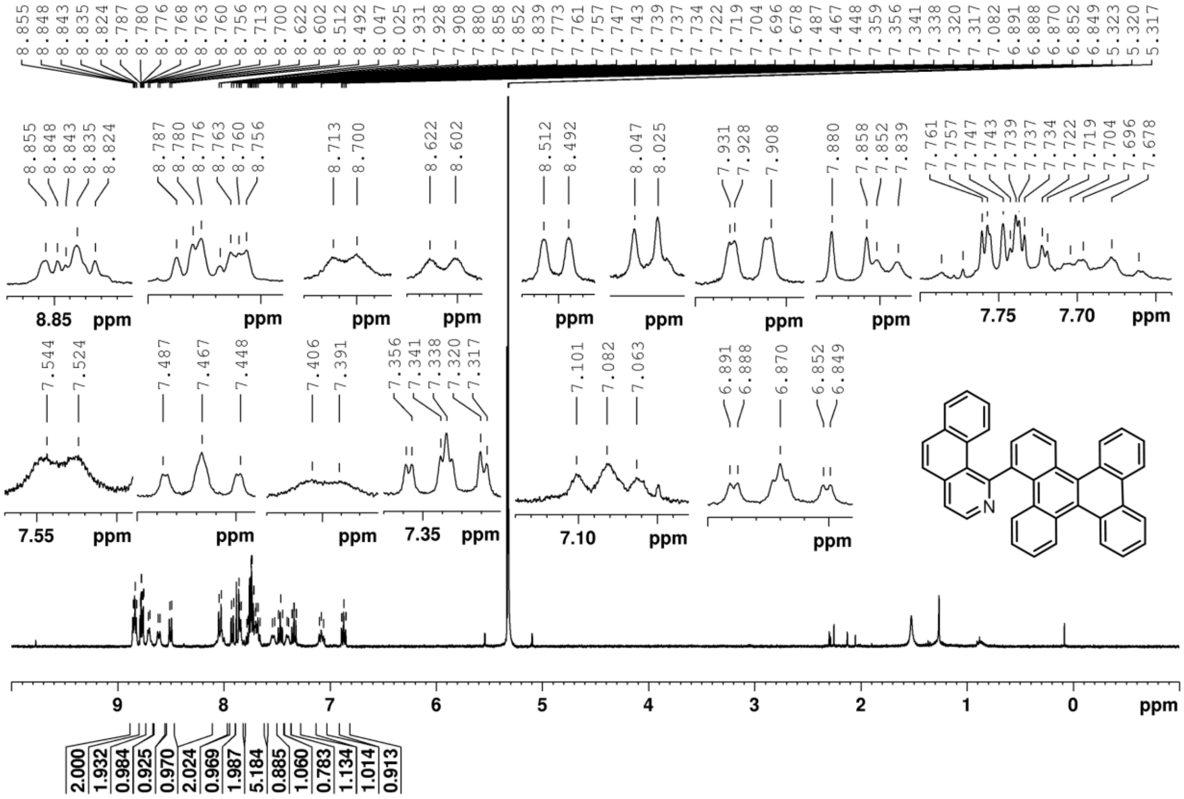

Figure S42. ^{1}H-NMR of compound **BA3** (400 MHz, CD_2Cl_2, 25 °C).

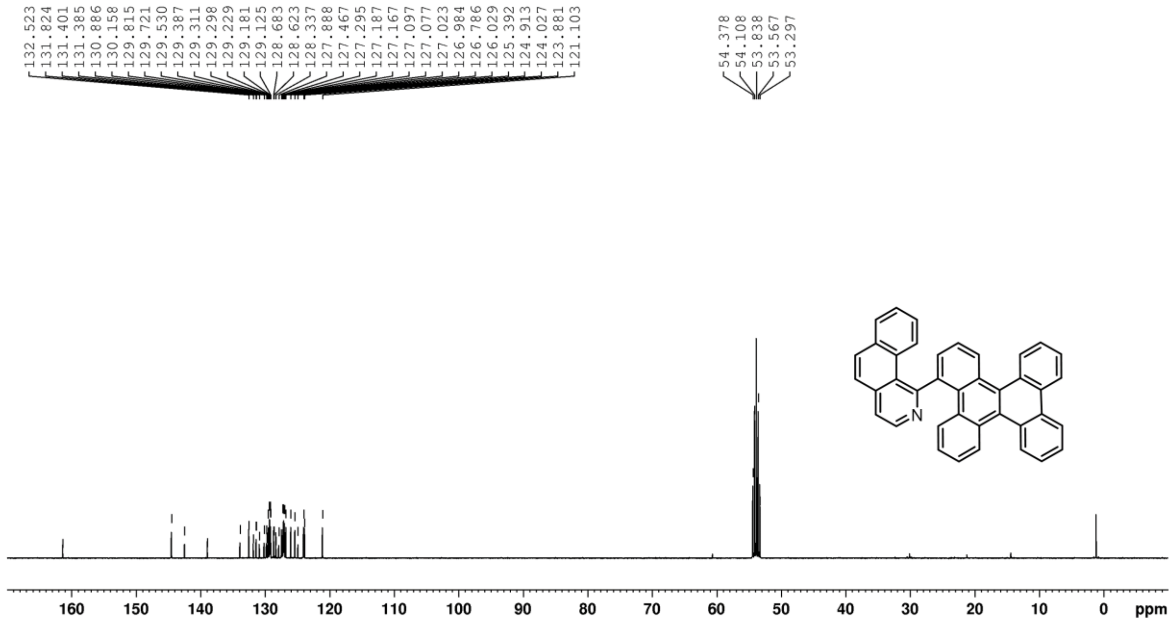

Figure S43. ^{13}C-NMR of compound **BA3** (100 MHz, CD_2Cl_2, 25 °C).

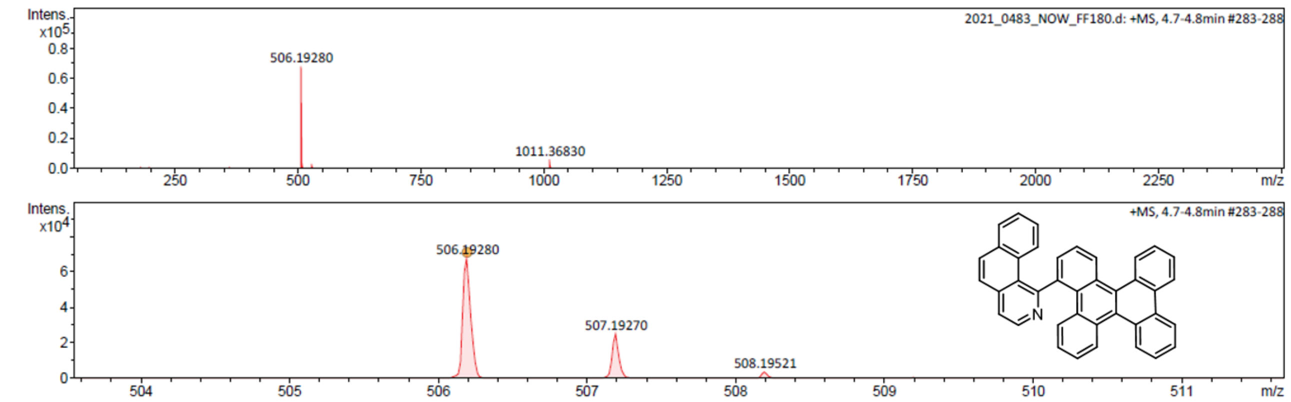

Meas. m/z	#	Ion Formula	m/z	err [ppm]	mSigma	# mSigma	Score	rdb	e⁻ Conf	N-Rule
506.19280	1	C39H24N	506.19033	-4.88	35.9	1	100.00	28.5	even	ok

Figure S44. HRMS (ESI) spectrum of compound **BA3**.

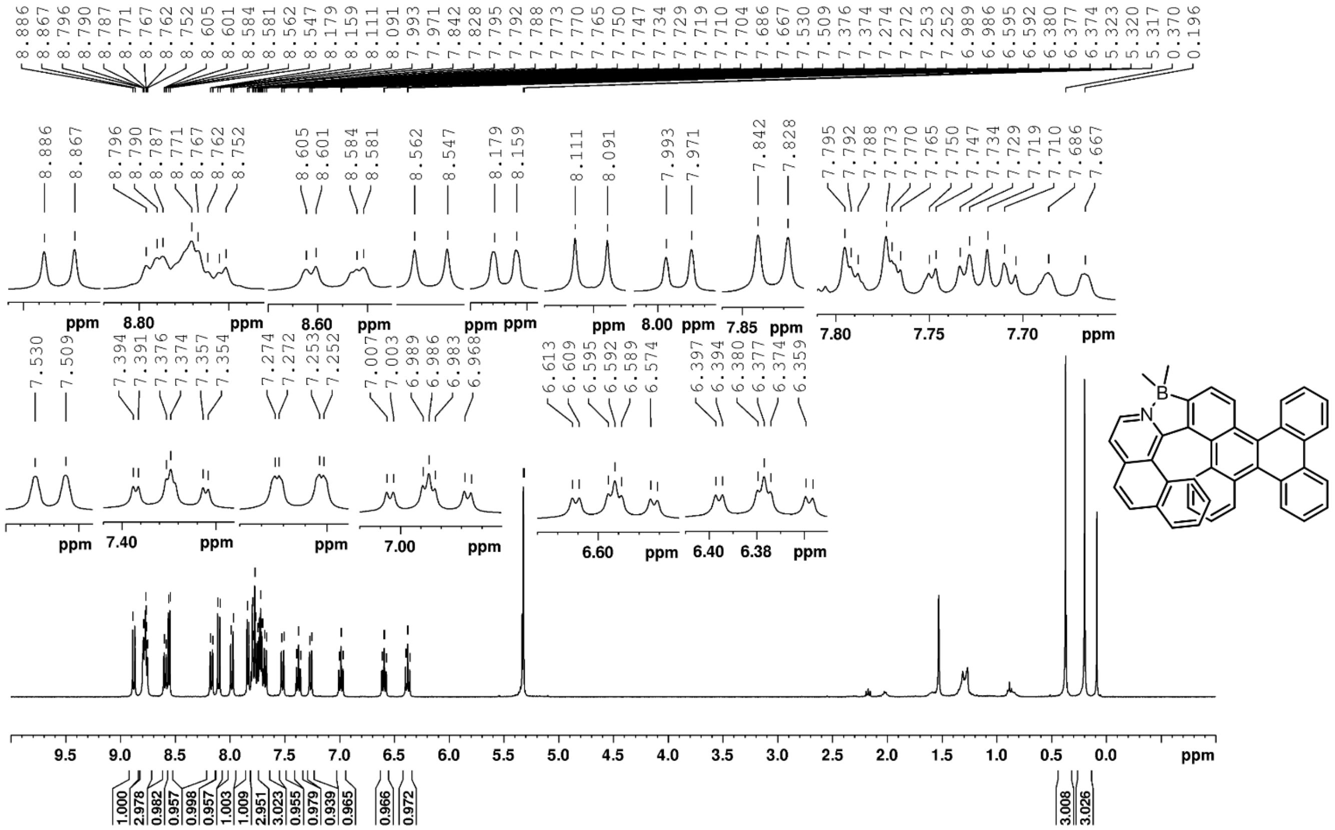

Figure S45. ^{1}H-NMR of compound **EH3** (400 MHz, CD_2Cl_2, 25 °C).

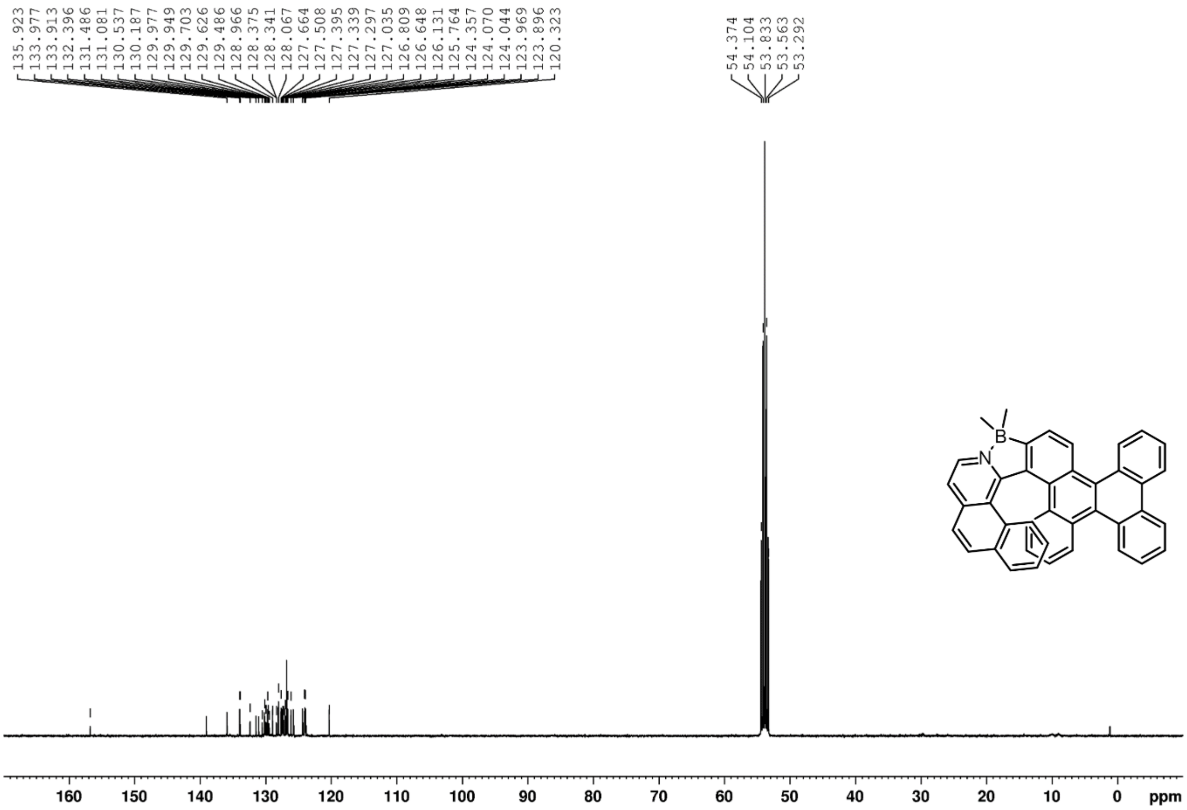

Figure S46. ^{13}C-NMR of compound **EH3** (100 MHz, CD_2Cl_2, 25 °C).

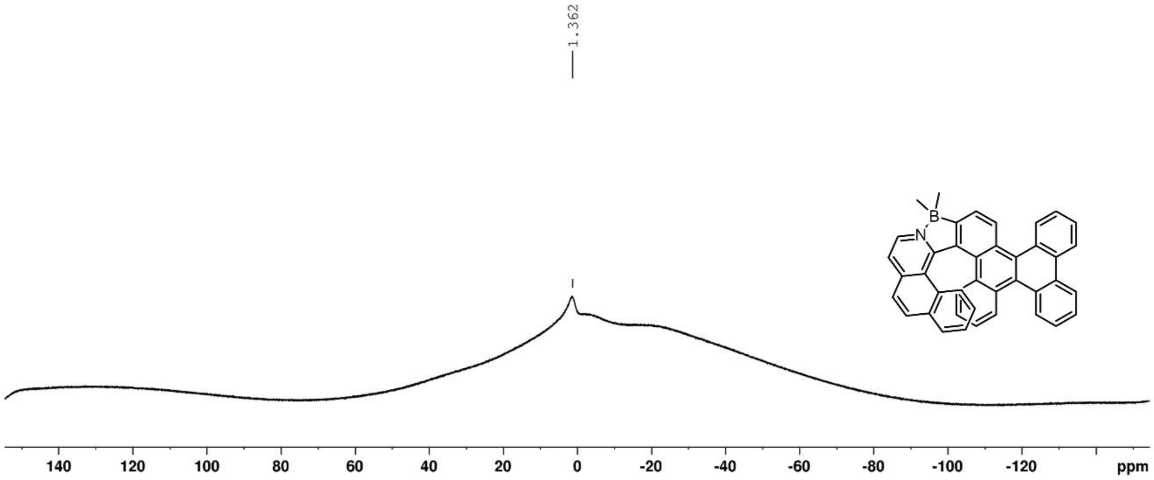

Figure S47. ^{11}B-NMR of compound **EH3** (128 MHz, CD_2Cl_2, 25 °C).

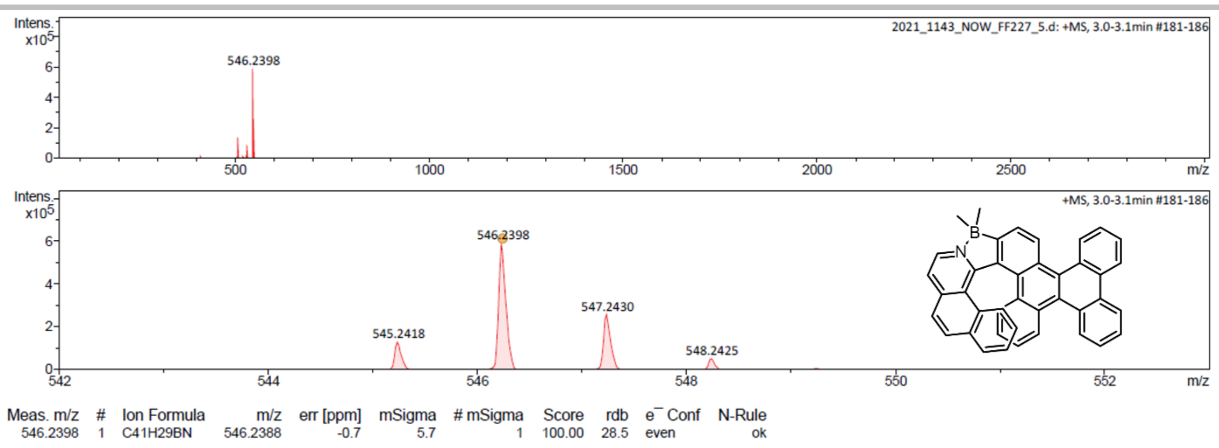

Figure S48. HRMS (APCI-DIP) spectrum of compound **EH3**.

4. Single crystal X-ray analysis

Single crystal X-ray data for **EH2** and **EH3** were collected at 100 K on a Rigaku XtaLAB Synergy-R diffractometer with a HPA area detector and multi-layer mirror monochromated $Cu_{K\alpha}$ radiation. The structure was solved using intrinsic phasing method,[S7] refined with the SHELXL program[S8] and expanded using Fourier techniques. All non-hydrogen atoms were refined anisotropically. Hydrogen atoms were included in structure factors calculations. All hydrogen atoms were assigned to idealised geometric positions.

Crystallographic data have been deposited with the Cambridge Crystallographic Data Center as supplementary publication no. CCDC-2175284 (**EH2**) and 2175285 (**EH3**). These data can be obtained free of charge from The Cambridge Crystallographic Data Centre *via* www.ccdc.cam.ac.uk/data_request/cif.

Crystal data for **EH2**: $C_{37}H_{26}BN$, $M_r = 495.40$, yellow block, $0.462\times0.282\times0.252$ mm^3, space group $P2_1/n$, $a = 10.63720(10)$ Å, $b = 16.11970(10)$ Å, $c = 14.90600(10)$ Å, $\alpha = 90°$, $\beta = 92.6900(10)°$, $\gamma = 90°$, $V = 2553.09(3)$ Å^3, $Z = 4$, $\rho_{calcd} = 1.289$ g·cm^{-3}, $\mu = 0.557$ mm^{-1}, $F(000) = 1040$, $T = 100.00(10)$ K, $R_1 = 0.0397$, $wR_2 = 0.0972$, 5079 independent reflections [$2\theta \leq 147.414°$] and 354 parameters.

Crystal data for **EH3**: $C_{41}H_{28}BN$, $M_r = 545.45$, orange block, $0.430\times0.397\times0.209$ mm^3, space group $P2_1/n$, $a = 9.14170(10)$ Å, $b = 12.94930(10)$ Å, $c = 23.7698(2)$ Å, $\alpha = 90°$, $\beta = 97.3480(10)°$, $\gamma = 90°$, $V = 2790.73(4)$ Å^3, $Z = 4$, $\rho_{calcd} = 1.298$ g·cm^{-3}, $\mu = 0.562$ mm^{-1}, $F(000) = 1144$, $T = 99.98(10)$ K, $R_1 = 0.0423$, $wR_2 = 0.1055$, 5581 independent reflections [$2\theta \leq 147.45°$] and 390 parameters.

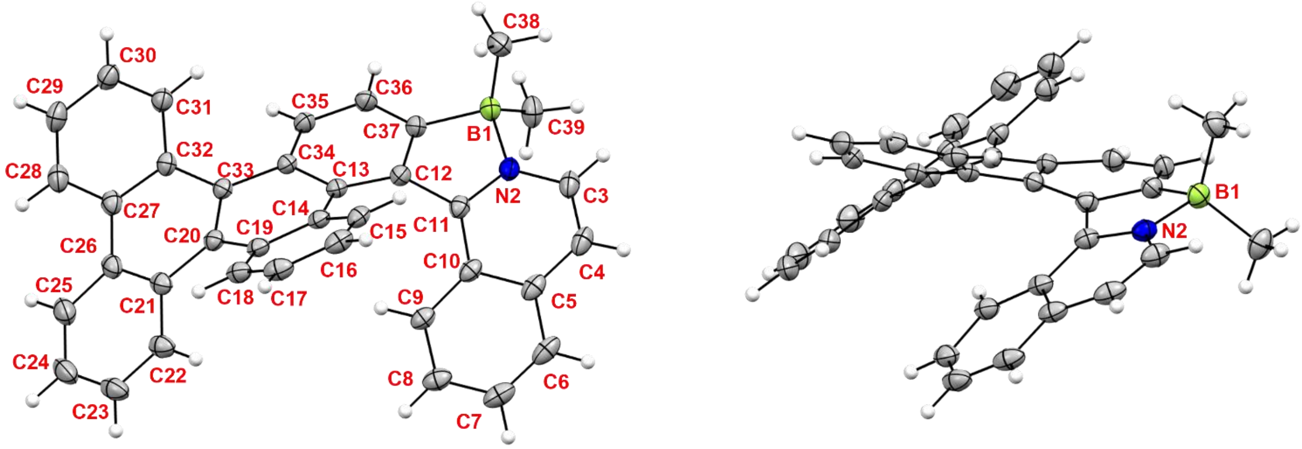

Figure S49. Molecular structure of **EH2** in the solid state. Thermal displacement parameters are displayed at the 50% probability.

Table S1. Selected angles of **EH2** in the solid state.

Angles	
∢ (C38-B1-N2)	108.33(9)°
∢ (C39-B1-N2)	108.33(9)°
∢ (C37-B1-38)	110.6(1)°
∢ (C38-B1-C39)	114.3(1)°
∢ (C37-B1-N2)	95.97(8)°
∢ (C37-B1-C39)	117.3(1)°

Table S2. Selected bond lengths of **EH2** in the solid state.

Bond Lengths / Å					
B1-N2	1.622(2)	C12-C13	1.416(1)	C23-C24	1.392(2)
B1-C37	1.610(2)	C12-C37	1.402(1)	C24-C25	1.373(2)
B1-C38	1.624(2)	C13-C14	1.453(1)	C25-C26	1.408(2)
B1-C39	1.613(2)	C13-C34	1.420(1)	C26-C27	1.456(1)
N2-C3	1.363(1)	C14-C15	1.410(2)	C27-C28	1.406(2)
N2-C11	1.343(1)	C14-C19	1.410(1)	C27-C32	1.419(1)
C3-C4	1.360(2)	C15-C16	1.377(2)	C28-C29	1.375(2)
C4-C5	1.414(2)	C16-C17	1.397(2)	C29-C30	1.392(2)
C5-C6	1.420(2)	C17-C18	1.376(2)	C30-C31	1.380(2)
C5-C10	1.423(2)	C18-C19	1.415(1)	C31-C32	1.414(1)
C6-C7	1.364(2)	C19-C20	1.456(1)	C32-C33	1.461(1)
C7-C8	1.411(2)	C20-C21	1.453(1)	C33-C34	1.466(1)
C8-C9	1.372(2)	C20-C33	1.395(1)	C34-C35	1.420(1)
C9-C10	1.419(2)	C21-C22	1.419(1)	C35-C36	1.382(1)
C10-C11	1.430(1)	C21-C26	1.411(2)	C36-C37	1.403(2)
C11-C12	1.474(1)	C22-C23	1.376(2)		

Table S3. Crystallographic data for single crystal X-ray analysis of **EH2.**

Empirical formula	$C_{37}H_{26}BN$
Formula weight (g·mol^{-1})	495.40
Temperature (K)	100.00(10)
Radiation, λ (Å)	$Cu_{K\alpha}$, 1.54184
Crystal system	monoclinic
Space group	$P2_1/n$
Unit cell dimensions	
a (Å)	10.63720(10)
b (Å)	16.11970(10)
c (Å)	14.90600(10)
α (°)	90
β (°)	92.6900(10)
γ (°)	90
Volume (Å^3)	2553.09(3)
Z	4
Calculated density (Mg·m^{-3})	1.289
Absorption coefficient (mm^{-1})	0.557
F(000)	1040
Theta range for collection	4.042 to 73.707°
Reflections collected	23701
Independent reflections	5079
Minimum/maximum transmission	0.351/1.000
Refinement method	Full-matrix least-squares on F^2
Data / parameters / restraints	5079 / 354 / 0
Goodness-of-fit on F^2	1.046
Final R indices [I>2σ(I)]	R_1 = 0.0378, wR_2 = 0.0957
R indices (all data)	R_1 = 0.0397, wR_2 = 0.0972
Maximum/minimum residual electron density (e·Å^{-3})	0.267 / –0.198

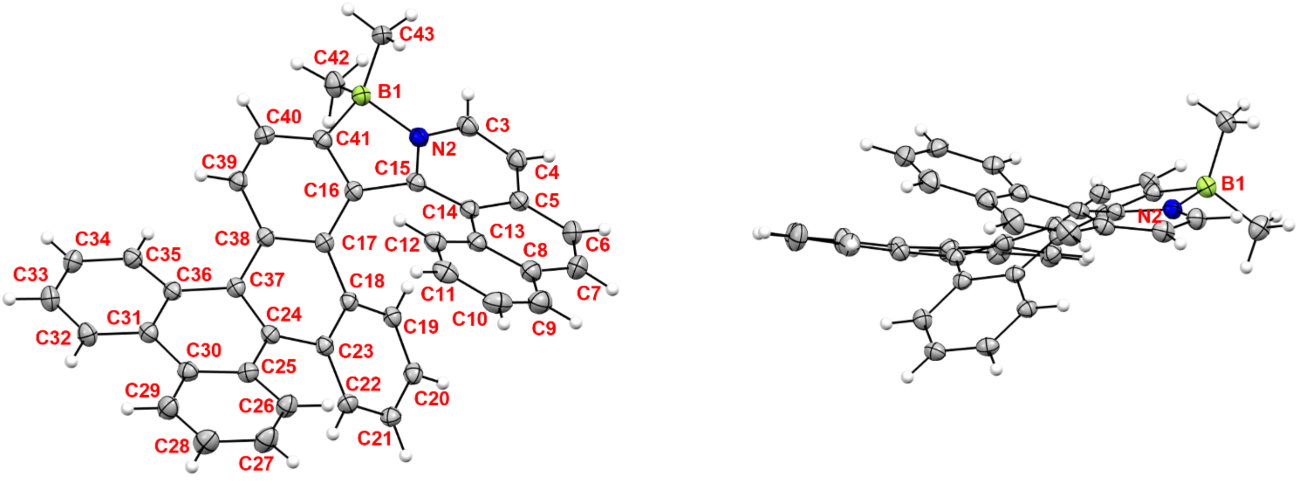

Figure S50. Molecular structure of **EH3** in the solid state. Thermal displacement parameters are displayed at the 50%

Table S4. Selected angles of **EH3** in the solid state.

Angles	
∢ (C41-B1-N2)	95.49(9)°
∢ (C42-B1-N2)	111.6(1)°
∢ (C43-B1-N2)	105.6(1)°
∢ (C43-B1-C42)	112.9(1)°
∢ (C43-B1-C41)	115.7(1)°
∢ (C42-B1-C41)	113.9(1)°

Table S5. Selected bond lengths of **EH3** in the solid state.

Bond Lengths / Å							
B1-N2	1.622(2)	C8-C13	1.419(2)	C19-C20	1.377(2)	C31-C36	1.420(2)
B1-C41	1.603(2)	C9-C10	1.373(2)	C20-C21	1.391(2)	C32-C33	1.372(2)
B1-C42	1.624(2)	C10-C11	1.390(2)	C21-C22	1.372(2)	C33-C34	1.393(2)
B1-C43	1.629(2)	C12-C13	1.411(2)	C23-C24	1.456(2)	C34-C35	1.381(2)
N2-C3	1.350(2)	C13-C14	1.457(2)	C24-C25	1.457(2)	C35-C36	1.412(2)
N2-C15	1.356(2)	C14-C15	1.426(2)	C24-C37	1.391(2)	C36-C37	1.462(2)
C3-C4	1.364(2)	C15-C16	1.485(2)	C25-C26	1.411(2)	C37-C38	1.463(2)
C4-C5	1.409(2)	C16-C17	1.423(2)	C26-C27	1.376(2)	C38-C39	1.419(2)
C5-C6	1.434(2)	C16-C41	1.403(2)	C27-C28	1.394(2)	C39-C40	1.377(2)
C5-C14	1.419(2)	C17-C18	1.455(2)	C28-C29	1.372(2)	C40-C41	1.402(2)
C6-C7	1.345(2)	C17-C38	1.422(2)	C29-C30	1.409(2)		
C7-C8	1.434(2)	C18-C19	1.407(2)	C30-C31	1.456(2)		
C8-C9	1.419(2)	C18-C23	1.417(2)	C31-C32	1.407(2)		

Table S6. Crystallographic data for single crystal X-ray analysis of **EH3.**

Empirical formula	$C_{41}H_{28}BN$
Formula weight (g·mol^{-1})	545.45
Temperature (K)	99.98(10)
Radiation, λ (Å)	$Cu_{K\alpha}$, 1.54184
Crystal system	monoclinic
Space group	$P2_1/n$
Unit cell dimensions	
a (Å)	9.14170(10)
b (Å)	12.94930(10)
c (Å)	23.7698(2)
α (°)	90
β (°)	97.3480(10)
γ (°)	90
Volume (Å^3)	2790.73(4)
Z	4
Calculated density (Mg·m^{-3})	1.298
Absorption coefficient (mm^{-1})	0.562
F(000)	1144
Theta range for collection	3.750 to 73.725°
Reflections collected	24511
Independent reflections	5581
Minimum/maximum transmission	0.405/1.000
Refinement method	Full-matrix least-squares on F^2
Data / parameters / restraints	5581 / 390 / 0
Goodness-of-fit on F^2	1.045
Final R indices [I>2σ(I)]	R_1 = 0.0407, wR_2 = 0.1043
R indices (all data)	R_1 = 0.0423, wR_2 = 0.1055
Maximum/minimum residual electron density (e·Å^{-3})	0.357 / –0.231

5. Optical and electrochemical properties

Absorption in the solid state

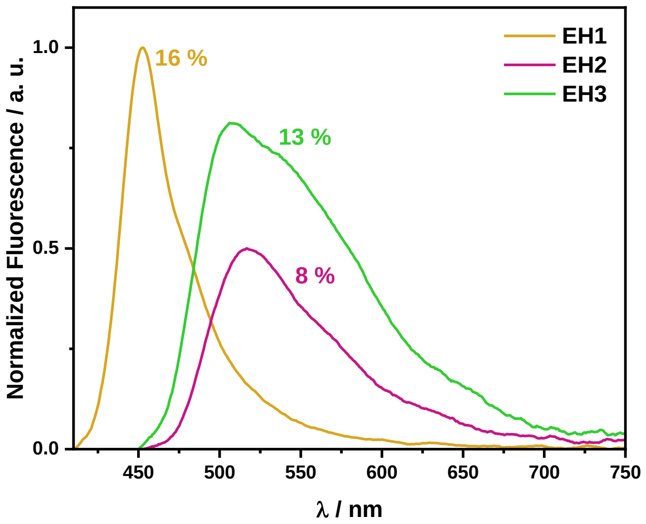

Figure S51. Emission spectra of **EH1** (yellow line, λ_{em} = 452 nm), **EH2** (magenta line, λ_{em} = 522 nm) and **EH3** (green line, λ_{em} = 507 nm) of amorphous powders upon excitation at λ_{ex} of 380 nm for **EH1** and 400 nm for **EH2** and **EH3**. The spectra are scaled to their absolute quantum yields determined by an integrating sphere setup.

Cyclic voltammetry and differential pulse voltammetry

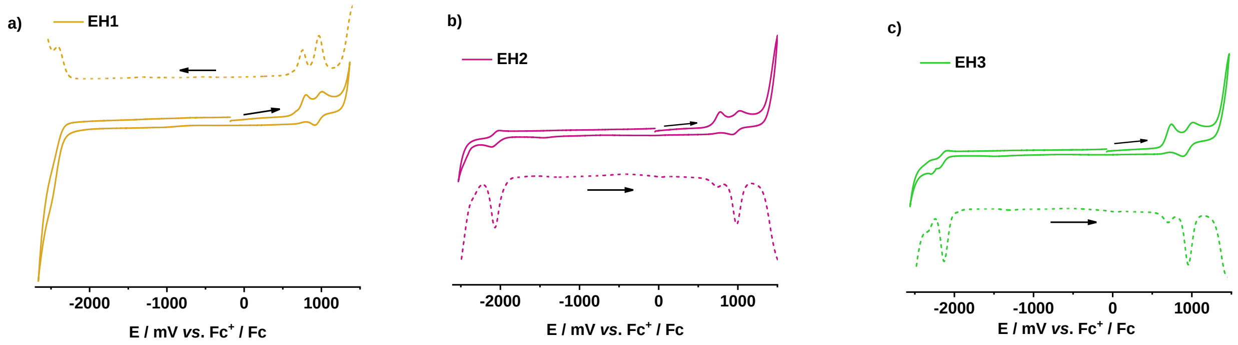

Figure S52. Cyclic (solid lines) and differential pulse (dashed lines) voltammograms of a) **EH1**, b) **EH2**, and c) **EH3** in CH_2Cl_2 with Bu_4NPF_6 as a supporting electrolyte and calibrated versus the ferrocenium/ferrocene (Fc^+/Fc) redox couple as an internal standard.

The HOMO / LUMO energy levels of **EH1-3** were calculated from CV measurements with the energy level of Fc^+/Fc set to -4.8 eV *vs.* vacuum:

$$E_{\mathrm{HOMO}} = -\mathrm{e} \cdot E^{\mathrm{ox}} - 4.8\ \mathrm{eV} \quad \text{(Eq. 1)}$$

$$E_{\mathrm{LUMO}} = -\mathrm{e} \cdot E^{\mathrm{red}} - 4.8\ \mathrm{eV} \quad \text{(Eq. 2)}$$

HOMO-LUMO band gaps were then calculated according to eq. 3 and are summarized in the table below.

$$E_{\mathrm{g}} = E^{\mathrm{ox}} - E^{\mathrm{red}} \quad \text{(Eq. 3)}$$

Table S7. Electrochemical properties of extended helicenes.[a]

Compound	$E_{1/2}^{red1}$ / V	$E_{1/2}^{red2}$ / V	$E_{1/2}^{ox1}$ / V	$E_{1/2}^{ox2}$ / V	E_{HOMO}[c] / eV	E_{LUMO}[c] / eV	E_g[c] / eV
**EH1**	(–2.41)	–	+0.80[b]<br>(+0.76)	+0.96<br>(+0.98)	–5.56	–2.39	3.17
**EH2**	–2.06<br>(–2.07)	–	+0.78[b]<br>(+0.74)	+0.98<br>(+0.98)	–5.54	–2.73	2.81
**EH3**	–2.15<br>(–2.12)	–2.30[b]<br>(–2.32)	+0.74[b]<br>(+0.72)	+0.95<br>(+0.97)	–5.52	–2.68	2.84

[a] Redox potentials were measured in dry CH_2Cl_2 (*c* ~ 10–4 – 10^{-5} M) at a scan rate of 100 mV s^{-1} and with 50% of iR compensation; supporting electrolyte Bu_4NPF_6 (*c* = 0.1 M). Measurements were calibrated vs. the ferrocenium/ferrocene (Fc+/Fc) redox couple as an internal standard. The values in parentheses correspond do DPV measurements. [b] Peak potential. [c] HOMO / LUMO levels and a band gap were calculated according to eq. 1-3.

6. Resolution of enantiomers

Analytical HPLC

Chiral resolution of racemates of **EH2** and **EH3** into their corresponding enantiomers was performed by HPLC using columns with a chiral stationary phase from Dr. Maisch GmbH (ReproSil Chiral-MIF). Samples of helicenes **(*rac*)-EH2** and **(*rac*)-EH3** were dissolved in *n*-hexane/CH_2Cl_2 (9:1), injected on a column with a chiral stationary phase (ReproSil Chiral-MIF, 250 ×4.6 mm) and eluted with *n*-hexane/CH_2Cl_2 (87:13) at room temperature with a flow rate of 1 mL min^{-1}.

Table S8. Parameters for chiral resolution of enantiomers by analytical HPLC

Compound	Column[a]	Eluent: ^{n}Hex/CH_2Cl_2	First fraction	Second fraction
(rac)-**EH2**	ReproSil Chiral-MIF	87/13	*M*	*P*
(rac)-**EH3**	ReproSil Chiral-MIF	87/13	*M*	*P*

[a] Dr-Maisch GmbH, ReproSil Chiral-MIF (250 × 4.6 mm). Sample injection: 10 µL of each racemate (rac)-**EH2** and (rac)-**EH3** in ^{n}hexane/CH_2Cl_2 (9/1, ~0.5 mg / 1 mL). Separation conditions: eluent, *n*-hexane/CH_2Cl_2, flow rate 1 mL/min, 20 ºC.

Semipreparative HPLC

Samples of helicenes **(*rac*)-EH2** and **(*rac*)-EH3** were dissolved in *n*-hexane/CH_2Cl_2 (9:1), injected on a column (0.5 mg mL^{-1}) with a chiral stationary phase (ReproSil Chiral-MIF, 250 ×10 mm) and eluted with *n*-hexane/CH_2Cl_2 (9:1) at room temperature with a flow rate of 20 mL min^{-1}.

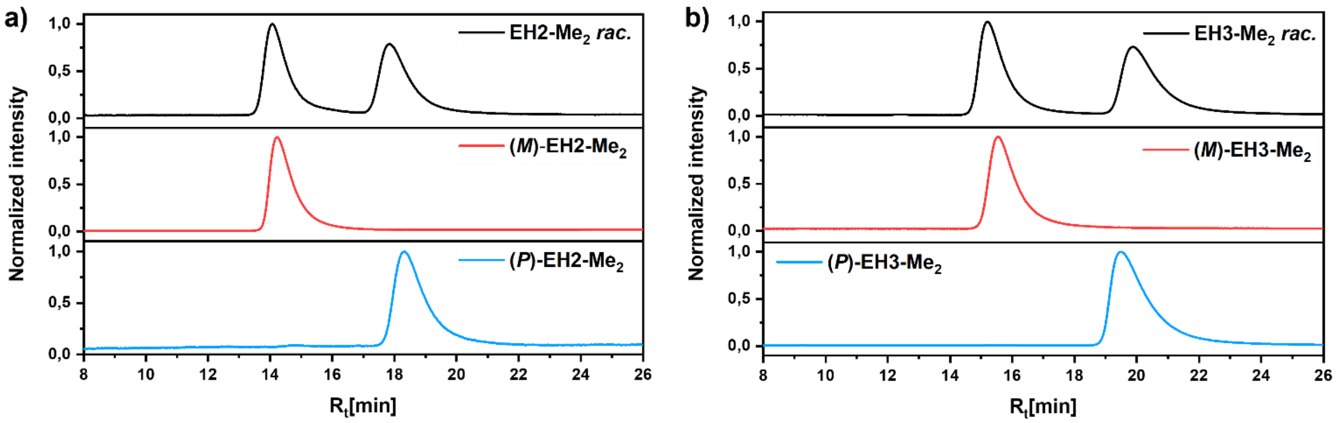

Figure S53. Chromatograms of a) **EH2** and b) **EH1**: racemates (top) and (*M*)- (middle), and (*P*)-enantiomers (bottom).

7. Interconversion Barrier

The baseline separation of the *M*- and the *P*-enantiomers of **EH2** and **EH3** allowed the determination of the activation parameters for racemization by time-dependent HPLC measurements. To this end, enantiopure samples of (*M*)-**EH2** (1.00 mg) and (*M*)-**EH3** (1.00 mg) were each dissolved in 1,2-dichlorobenzene (2.0 mL). Afterwards, the solutions were heated at 65 °C (**EH2**) and 170 °C (**EH3**). Aliquots (50 µL) were periodically removed from the solutions and analyzed by HPLC on a chiral stationary phase (ReproSil Chiral-MIF) at room temperature to determine the enantiomeric ratio. Our studies showed that the compounds were configurationally stable at room temperature. Thus, the racemization process during the resolution by HPLC can be neglected.

P-*M* isomerization is a reversible unimolecular reaction and follows first-order kinetics:

$$v = \frac{d(A)}{A_0} = kdt, \qquad \text{(Eq. 4)}$$

where, *A* denotes the concentration of the enantiomer which is present in excess and A_0 is the initial concentration thereof. *k* denotes the reaction rate constant. Integration of the first-order rate law yields:

$$\ln\frac{A}{A_0} = -kt, \qquad \text{(Eq. 5)}$$

Thus, the rate constant *k* at a given temperature can be determined from the exponential decay of the A/A_0 value over time:

$$\frac{A}{A_0} = e^{-kt}, \qquad \text{(Eq. 6)}$$

Half -life $t_{1/2}$ can be calculated from the equation:

$$t_{1/2} = \frac{\ln(2)}{k}, \qquad \text{(Eq. 7)}$$

The free activation enthalpy of racemization is determined from Eyring equation:

$$\Delta G^{\neq} = -RTln\frac{hk}{\sigma k_B T}, \qquad \text{(Eq. 8)}$$

Where *R, T, h, k,* k_B are gas constant, absolute temperature, Planck's constant, rate constant and Boltzmann constant, respectively. Parameter σ was assumed as 0.5, as the probability of conversion to *P*- or *M*- enantiomer is equal.

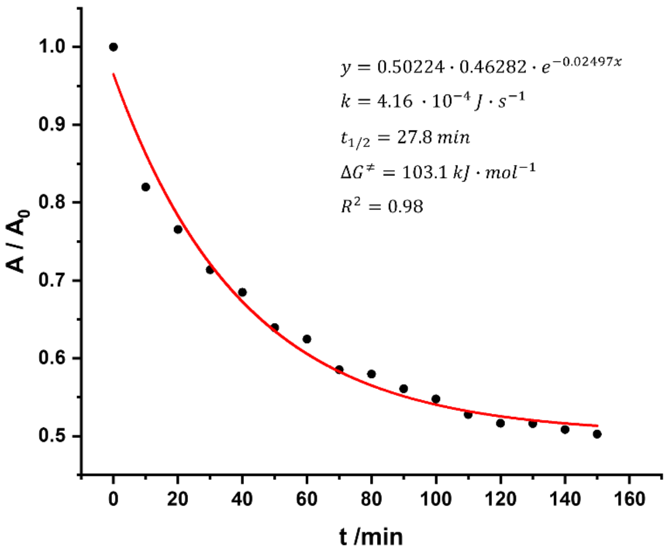

Figure S54. Exponential decay of the A/A_0 value for **(*M*)-EH2** at 65 °C in 1,2-dichlorobenzene over time.

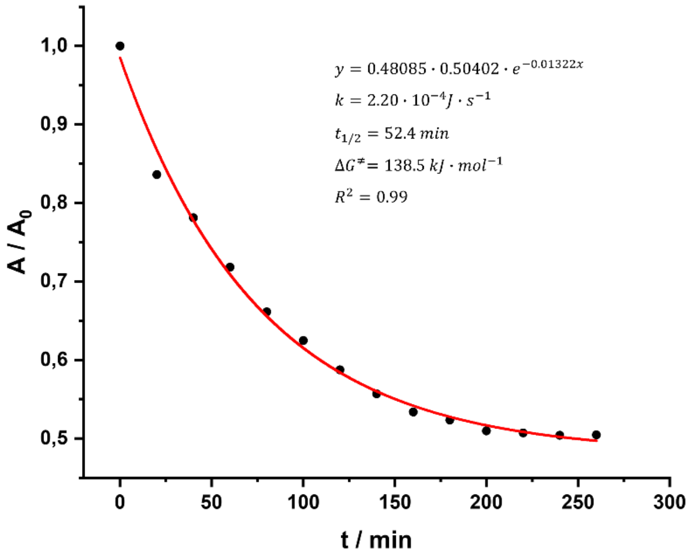

Figure S55. Exponential decay of the *A*/A_0 value for (*M*)-**EH3** at 170 °C in 1,2-dichlorobenzene over time.

8. Chiroptical properties

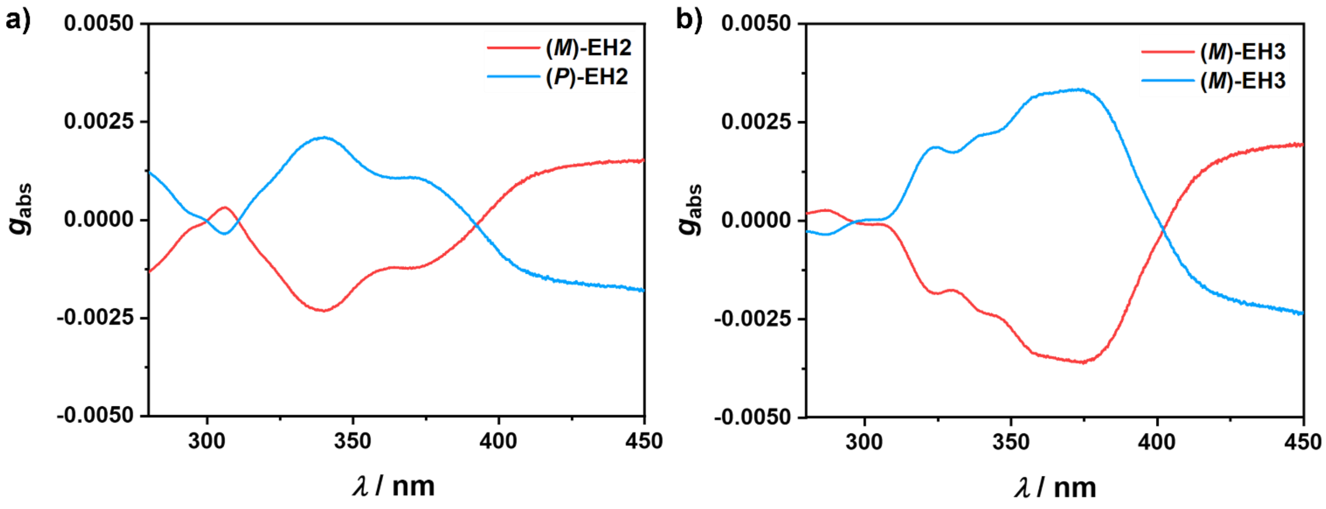

Figure S56. Absorption dissymmetry factors (g_{abs}) of a) **EH2** and b) **EH3**.

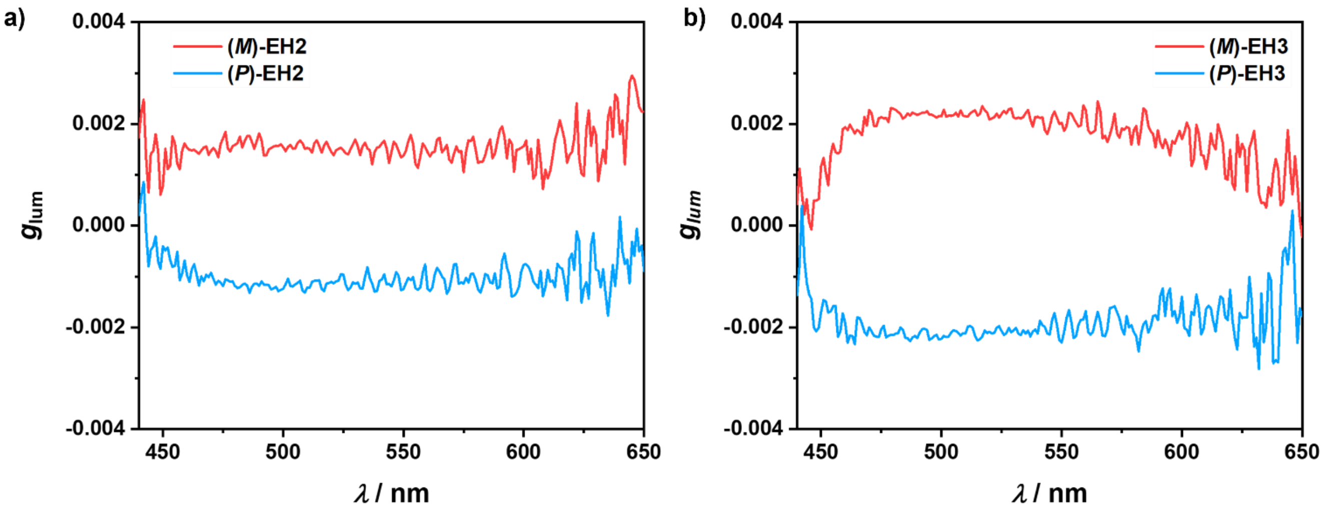

Figure S57. Luminescence dissymmetry factors (g_{lum}) of a) **EH2** and b) **EH3**.

9. Computations

DFT calculations were performed using the Gaussian 16 program package[S9] with B3LYP[S10] as a functional and def2-TZVP[S11] as a basis set with Grimme's D3(BJ) dispersion correction.[S12] CH_2Cl_2 was used as a solvent for calculations (PCM model). The structures were geometry optimized followed by frequency calculations on the optimized structures, which confirmed the presence of minima (no imaginary frequencies).

Time-dependent (TD)-DFT calculations were carried out on the optimized structures employing a hybrid exchange-correlation functional using the Coulomb-attenuating method (CAM-B3LYP)[S13] with Grimme's D3(BJ) dispersion correction and def2-TZVP as a basis set. CH_2Cl_2 was used as a solvent for calculations (PCM model). The most stable conformations of **EH1**-**EH3** were considered for calculations. The excited state energies as well as the corresponding oscillator strengths (*f*) are listed in Tables S10-S12. The UV/Vis and CD spectra were simulated using the GaussView 5[S14] visualization software package. The half-widths of 1600 cm^{-1} were assumed for a proper simulation of the absorption and CD spectra.

The g_{abs} and g_{lum} values were calculated following the approach published by Kubo *et al*.[S15] The S_0 and S_1 geometries were optimized using hybrid meta-GGA M06-2X functional[S16] and def2-SVP[S11] as a basis set. The presence of the minima was confirmed by frequency calculations on the optimized structures. The electric and magnetic transition dipole moments were visualized using VMD software.[S17]

The *P*-*M* interconversion processes were studied using B3LYP[S10] as a functional and def2-SVP[S11] as a basis set with Grimme's D3(BJ) dispersion correction.[S12] CH_2Cl_2 was used as a solvent for calculations (PCM model). The character of the obtained structures has been verified by vibrational frequency analysis (no imaginary frequencies for minima and one imaginary frequency for transition states).

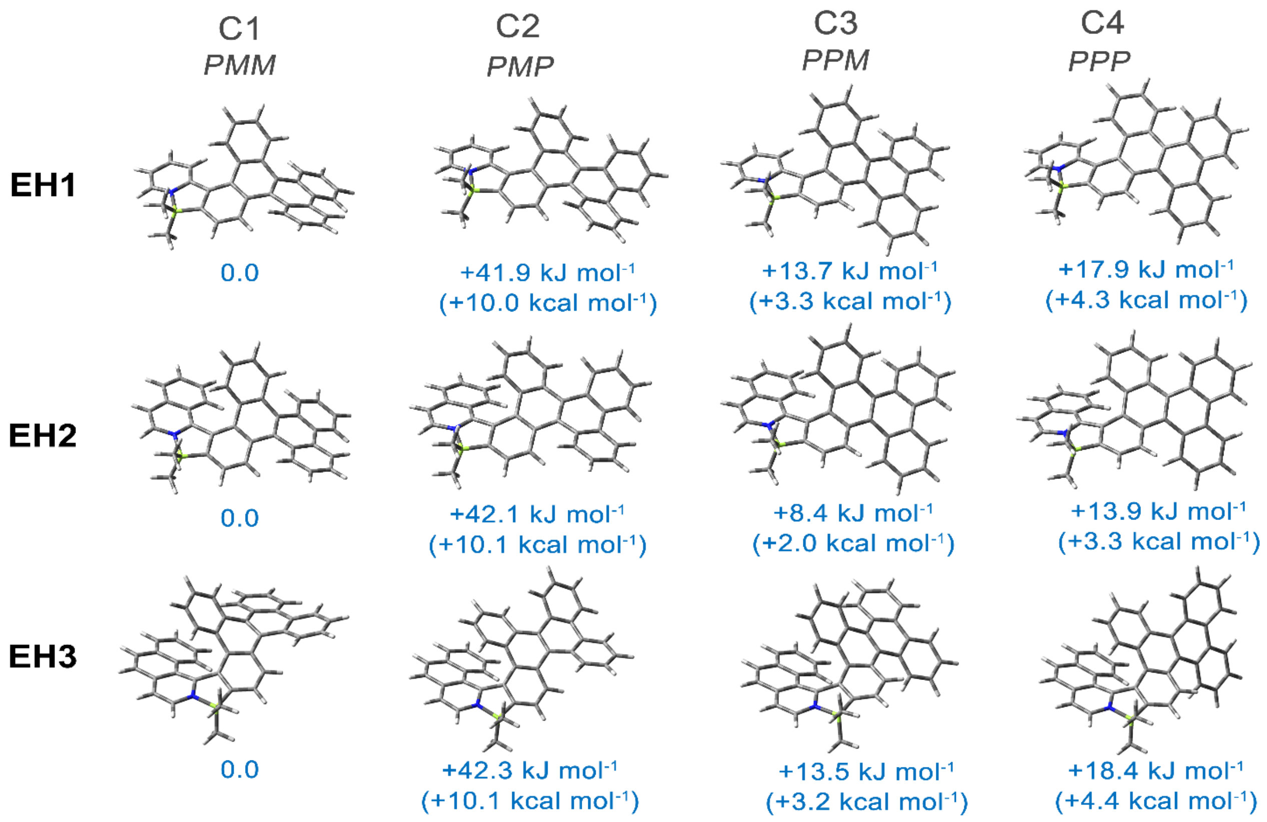

Figure S58. Structures of **C1**-**C4** conformations of **EH1**-**EH3** optimized at the B3LYP-D3(BJ)/def2-SVP (solvent CH_2Cl_2, PCM model) level of theory and their relative Gibbs free energies.

Table S9. Computed relative Gibbs free energies of **C1**-**C4** conformations of **EH1**-**EH3**.[a]

Method	Compound	C1	C2	C3	C4
B3LYP-D3(BJ)/def2SVP (CH_2Cl_2, PCM model)	**EH1**	0.0	+41.9	+13.7	+17.9
	**EH2**	0.0	+42.1	+8.4	+13.9
	**EH3**	0.0	+42.3	+13.5	+18.4
B3LYP-D3(BJ)/def2TZVP (CH_2Cl_2, PCM model)	**EH1**	0.0	+42.8	+13.8	+18.3
	**EH2**	0.0	+43.4	+8.3	+14.3
	**EH3**	0.0	+42.9	+13.3	+17.5

[a] In kJ mol^{-1}.

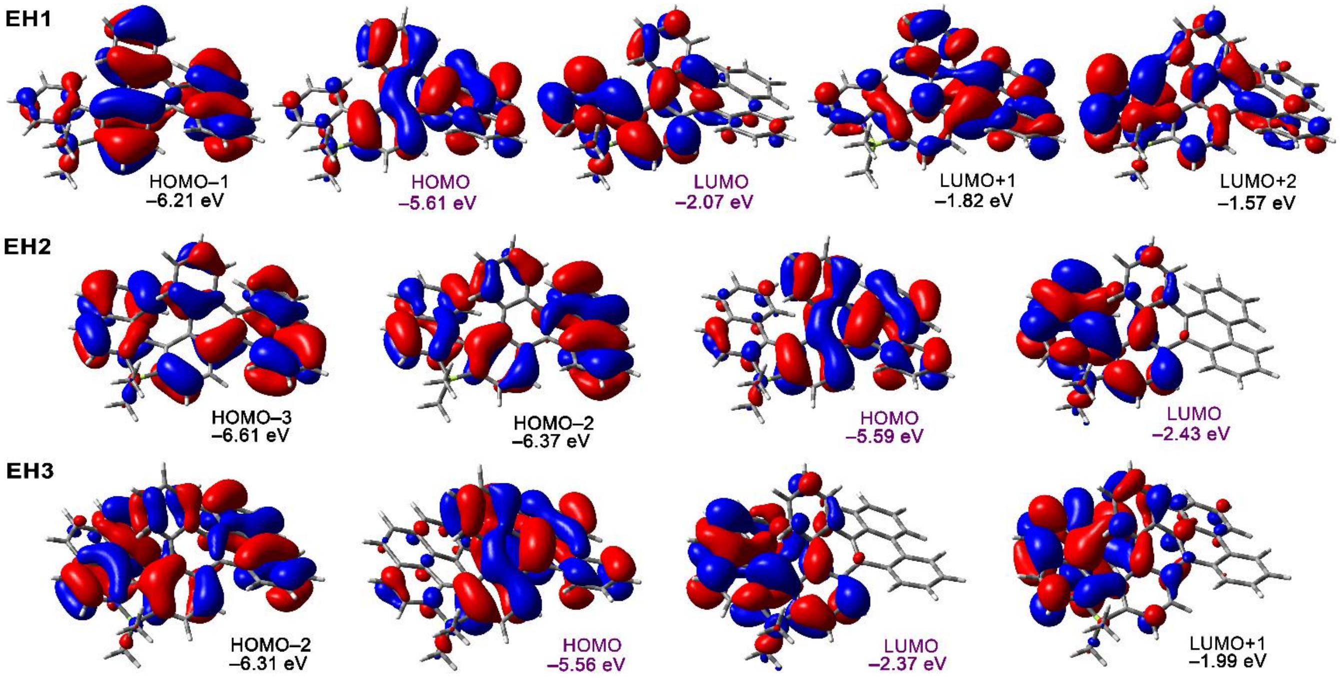

Figure S59. Contour plots of selected Kohn–Sham orbitals (isovalue=0.02 a.u.) of **EH1**-**EH3** calculated at the B3LYP-D3(BJ)/def2-TZVP (solvent CH_2Cl_2, PCM model) level of theory.

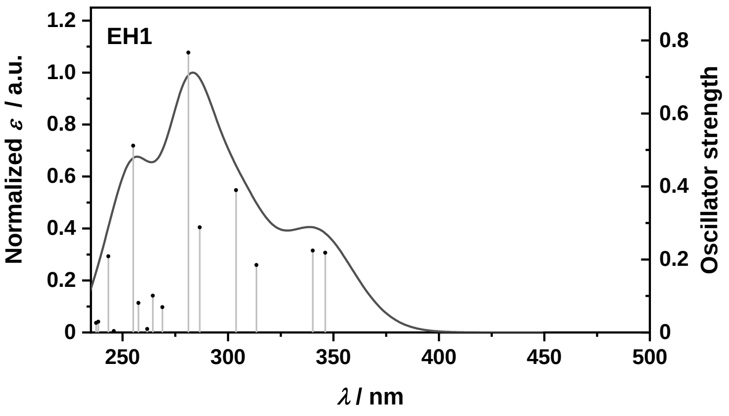

Figure S60. The simulated UV/Vis absorption spectra of **EH1** at the CAM-B3LYP-D3(BJ)/def2-TZVP (solvent CH_2Cl_2) level of theory.

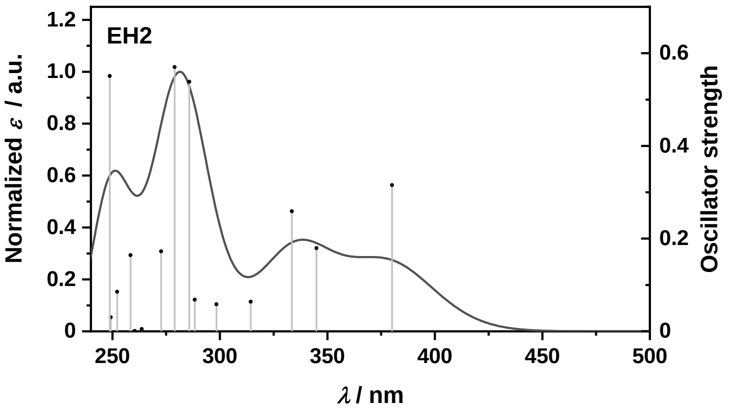

Figure S61. The simulated UV/Vis absorption spectra of **EH2** at the CAM-B3LYP-D3(BJ)/def2-TZVP (solvent CH_2Cl_2) level of theory.

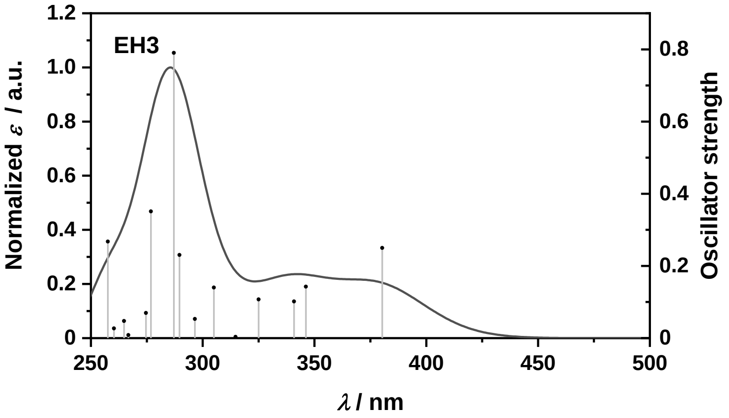

Figure S62. The simulated UV/Vis absorption spectra of **EH3** at the CAM-B3LYP-D3(BJ)/def2-TZVP (solvent CH_2Cl_2) level of theory.

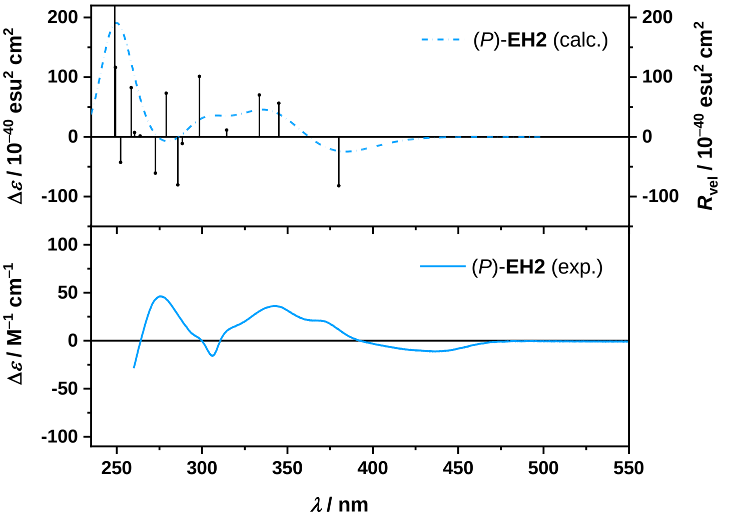

Figure S63. The simulated (dash-dotted line; CAM-B3LYP-D3(BJ)/def2-TZVP, CH_2Cl_2, PCM model) and experimental (solid line; CH_2Cl_2; $c = 3.7 \times 10^{-5}$ M) ECD spectra of (*P*)-**EH2**.

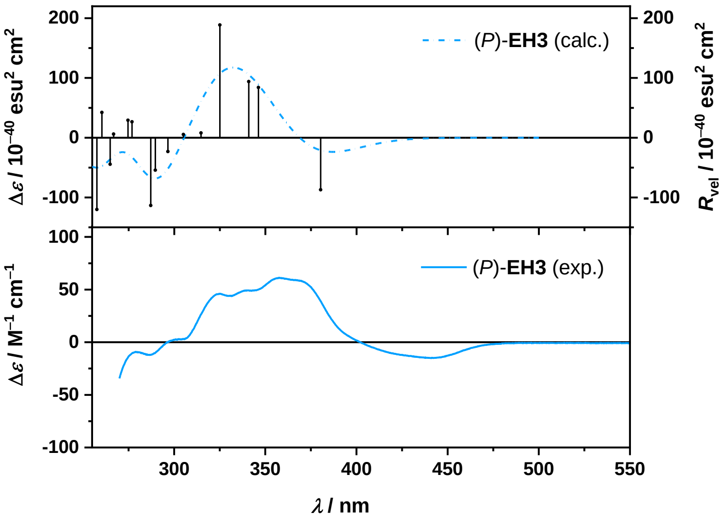

Figure S64. The simulated (dash-dotted line; CAM-B3LYP-D3(BJ)/def2-TZVP, CH_2Cl_2, PCM model) and experimental (solid line; CH_2Cl_2; $c = 2.7 \times 10^{-5}$ M) ECD spectra of (*P*)-**EH3**.

Table S10. TD-DFT-calculated UV/Vis absorption data for (*P*)-**EH1** at the CAM-B3LYP-D3(BJ)/def2-TZVP (solvent CH_2Cl_2) level.

	Calcd. *λ* [nm]	Oscillator strength (*f*)	Composition	Coefficient	Contribution
1	346.11	0.2185	HOMO-1 -> LUMO+1	-0.19932	8%
			HOMO -> LUMO	0.61424	75%
			HOMO -> LUMO+2	-0.15854	5%
2	340.2	0.2244	HOMO-1 -> LUMO	0.14352	4%
			HOMO -> LUMO+1	0.6544	86%
3	313.51	0.1847	HOMO-3 -> LUMO	0.14195	4%
			HOMO-2 -> LUMO	-0.16464	5%
			HOMO-2 -> LUMO+1	-0.18835	7%
			HOMO-1 -> LUMO	0.35533	25%
			HOMO-1 -> LUMO+1	-0.20283	8%
			HOMO -> LUMO	-0.13939	4%
			HOMO -> LUMO+1	-0.12374	3%
			HOMO -> LUMO+2	-0.29333	17%
			HOMO -> LUMO+3	0.29765	18%
4	303.82	0.3899	HOMO-2 -> LUMO	0.35344	25%
			HOMO-2 -> LUMO+1	-0.10472	2%
			HOMO-1 -> LUMO	-0.34379	24%
			HOMO-1 -> LUMO+1	-0.16259	5%
			HOMO -> LUMO+3	0.38828	30%
5	286.57	0.2881	HOMO-3 -> LUMO	-0.16807	6%
			HOMO-3 -> LUMO+2	-0.12355	3%
			HOMO-2 -> LUMO+1	-0.19745	8%

			HOMO-1 -> LUMO	0.31046	19%
			HOMO-1 -> LUMO+1	0.10229	2%
			HOMO-1 -> LUMO+2	-0.14484	4%
			HOMO -> LUMO	0.10529	2%
			HOMO -> LUMO+2	0.39421	31%
			HOMO -> LUMO+3	0.13741	4%
			HOMO -> LUMO+4	-0.18077	7%
6	281.21	0.7667	HOMO-3 -> LUMO	0.10871	2%
			HOMO-2 -> LUMO	-0.14723	4%
			HOMO-1 -> LUMO	-0.1058	2%
			HOMO-1 -> LUMO+1	0.54297	59%
			HOMO -> LUMO	0.15385	5%
			HOMO -> LUMO+2	-0.16032	5%
			HOMO -> LUMO+3	0.25349	13%
7	268.9	0.0693	HOMO-3 -> LUMO+2	-0.10813	2%
			HOMO-2 -> LUMO+1	0.12937	3%
			HOMO-2 -> LUMO+2	0.16175	5%
			HOMO-1 -> LUMO+2	-0.12071	3%
			HOMO -> LUMO+2	0.15147	5%
			HOMO -> LUMO+3	0.1342	4%
			HOMO -> LUMO+4	0.54942	60%
8	264.39	0.1007	HOMO-3 -> LUMO	-0.30477	19%
			HOMO-3 -> LUMO+1	-0.19398	8%
			HOMO-2 -> LUMO+1	0.38278	29%
			HOMO-2 -> LUMO+2	-0.11126	2%
			HOMO-1 -> LUMO	0.12843	3%
			HOMO-1 -> LUMO+2	0.16168	5%
			HOMO-1 -> LUMO+3	-0.11257	3%
			HOMO -> LUMO	-0.11761	3%
			HOMO -> LUMO+2	-0.1008	2%
			HOMO -> LUMO+3	0.14528	4%
9	261.69	0.0094	HOMO-3 -> LUMO+1	-0.11529	3%
			HOMO-2 -> LUMO	0.33268	22%
			HOMO-2 -> LUMO+1	-0.31188	19%
			HOMO-1 -> LUMO+3	-0.11607	3%
			HOMO -> LUMO+2	-0.18673	7%
			HOMO -> LUMO+3	-0.2822	16%
			HOMO -> LUMO+4	0.27432	15%
10	257.53	0.0809	HOMO-4 -> LUMO+1	-0.10069	2%

			HOMO-3 -> LUMO	-0.11644	3%
			HOMO-2 -> LUMO+2	0.36519	27%
			HOMO-2 -> LUMO+3	-0.1187	3%
			HOMO-1 -> LUMO+2	-0.19436	8%
			HOMO-1 -> LUMO+3	-0.2589	13%
			HOMO -> LUMO	-0.10775	2%
			HOMO -> LUMO+2	-0.15905	5%
			HOMO -> LUMO+4	-0.11251	3%
			HOMO -> LUMO+5	-0.27878	16%
11	255.07	0.5116	HOMO-3 -> LUMO	-0.1705	6%
			HOMO-3 -> LUMO+1	0.17053	6%
			HOMO-2 -> LUMO	0.12987	3%
			HOMO-2 -> LUMO+3	0.29375	17%
			HOMO-1 -> LUMO+1	0.12371	3%
			HOMO-1 -> LUMO+2	-0.28359	16%
			HOMO-1 -> LUMO+3	0.30328	18%
			HOMO -> LUMO+1	-0.1128	3%
			HOMO -> LUMO+2	-0.23371	11%
			HOMO -> LUMO+4	-0.11687	3%
12	245.87	0.0039	HOMO-6 -> LUMO+3	0.10554	2%
			HOMO-4 -> LUMO+3	-0.14088	4%
			HOMO-3 -> LUMO	0.11999	3%
			HOMO-3 -> LUMO+3	0.1489	4%
			HOMO-2 -> LUMO+1	0.12951	3%
			HOMO-2 -> LUMO+2	-0.10947	2%
			HOMO-2 -> LUMO+4	0.25769	13%
			HOMO-1 -> LUMO+4	0.40068	32%
13	243.25	0.2086	HOMO-3 -> LUMO	-0.29589	18%
			HOMO-3 -> LUMO+1	0.46629	43%
			HOMO-3 -> LUMO+2	0.11207	3%
			HOMO-2 -> LUMO+1	-0.14104	4%
			HOMO-2 -> LUMO+3	-0.15111	5%
			HOMO-1 -> LUMO+2	0.16364	5%
			HOMO-1 -> LUMO+3	-0.12774	3%
			HOMO -> LUMO+6	0.11639	3%
14	238.52	0.0295	HOMO-7 -> LUMO	-0.36547	27%
			HOMO-6 -> LUMO	0.37904	29%
			HOMO-1 -> LUMO+2	0.21743	9%
			HOMO-1 -> LUMO+3	0.13842	4%

			HOMO -> LUMO+5	-0.19434	8%
15	237.5	0.0263	HOMO-8 -> LUMO	-0.14748	4%
			HOMO-7 -> LUMO	-0.21388	9%
			HOMO-6 -> LUMO	0.24455	12%
			HOMO-4 -> LUMO	0.12173	3%
			HOMO-3 -> LUMO+2	0.13834	4%
			HOMO-1 -> LUMO+2	-0.21498	9%
			HOMO-1 -> LUMO+3	-0.1984	8%
			HOMO-1 -> LUMO+4	-0.11903	3%
			HOMO-1 -> LUMO+5	-0.10021	2%
			HOMO -> LUMO+5	0.32363	21%
			HOMO -> LUMO+6	0.12732	3%

Table S11. TD-DFT-calculated UV/Vis absorption data for (*P*)-**EH2** at the CAM-B3LYP-D3(BJ)/def2-TZVP (solvent CH_2Cl_2) level.

	Calcd. *λ* [nm]	Oscillator strength (*f*)	Composition	Coefficient	Contribution
1	380.05	0.3154	HOMO-3 -> LUMO	-0.10528	2%
			HOMO-2 -> LUMO	0.17387	6%
			HOMO -> LUMO	0.63009	79%
2	344.92	0.1796	HOMO-1 -> LUMO	-0.18781	7%
			HOMO -> LUMO+1	0.64476	83%
3	333.47	0.2591	HOMO-2 -> LUMO	0.33845	23%
			HOMO-1 -> LUMO	0.48499	47%
			HOMO-1 -> LUMO+1	-0.19979	8%
			HOMO -> LUMO	-0.1128	3%
			HOMO -> LUMO+1	0.15545	5%
			HOMO -> LUMO+2	0.17663	6%
4	314.35	0.0639	HOMO-3 -> LUMO	-0.11774	3%
			HOMO-2 -> LUMO+1	0.18681	7%
			HOMO-1 -> LUMO	-0.21081	9%
			HOMO-1 -> LUMO+1	-0.3166	20%
			HOMO -> LUMO	0.12881	3%
			HOMO -> LUMO+2	0.35873	26%
			HOMO -> LUMO+3	-0.3176	20%
5	298.39	0.0584	HOMO-6 -> LUMO	0.13981	4%
			HOMO-3 -> LUMO	0.37737	28%
			HOMO-3 -> LUMO+1	0.11397	3%
			HOMO-2 -> LUMO	-0.28155	16%

			HOMO-2 -> LUMO+1	0.1247	3%
			HOMO-1 -> LUMO	0.23552	11%
			HOMO -> LUMO+3	-0.22403	10%
			HOMO -> LUMO+4	-0.17647	6%
6	288.33	0.0683	HOMO-4 -> LUMO	0.15968	5%
			HOMO-3 -> LUMO	-0.23876	11%
			HOMO-3 -> LUMO+1	0.16095	5%
			HOMO-2 -> LUMO	0.22725	10%
			HOMO-2 -> LUMO+1	0.15346	5%
			HOMO-1 -> LUMO+1	0.32316	21%
			HOMO -> LUMO	-0.17516	6%
			HOMO -> LUMO+3	-0.26336	14%
			HOMO -> LUMO+4	-0.21556	9%
7	285.75	0.5383	HOMO-5 -> LUMO	0.12202	3%
			HOMO-4 -> LUMO	0.38829	30%
			HOMO-3 -> LUMO	-0.1316	3%
			HOMO-3 -> LUMO+1	-0.136	4%
			HOMO-2 -> LUMO+3	-0.14053	4%
			HOMO-1 -> LUMO+1	-0.32998	22%
			HOMO -> LUMO+2	-0.29709	18%
8	278.97	0.57	HOMO-6 -> LUMO	0.13627	4%
			HOMO-3 -> LUMO+1	-0.10486	2%
			HOMO-2 -> LUMO+1	-0.22819	10%
			HOMO-2 -> LUMO+2	-0.13564	4%
			HOMO-2 -> LUMO+3	-0.12181	3%
			HOMO-1 -> LUMO+1	0.22372	10%
			HOMO -> LUMO+2	0.17294	6%
			HOMO -> LUMO+3	-0.23443	11%
			HOMO -> LUMO+4	0.36507	27%
			HOMO -> LUMO+5	-0.17687	6%
9	272.64	0.1725	HOMO-8 -> LUMO	0.14549	4%
			HOMO-4 -> LUMO	0.4063	33%
			HOMO-2 -> LUMO	-0.22119	10%
			HOMO-2 -> LUMO+1	-0.13052	3%
			HOMO-2 -> LUMO+2	0.17296	6%
			HOMO-2 -> LUMO+3	0.10507	2%
			HOMO-1 -> LUMO	0.15642	5%
			HOMO-1 -> LUMO+2	0.14687	4%
			HOMO -> LUMO+2	0.20309	8%

10	263.65	0.005	HOMO-2 -> LUMO	-0.10067	2%
			HOMO-2 -> LUMO+1	0.37488	28%
			HOMO-2 -> LUMO+2	0.14296	4%
			HOMO -> LUMO+2	-0.10784	2%
			HOMO -> LUMO+4	0.34803	24%
			HOMO -> LUMO+5	-0.29295	17%
			HOMO -> LUMO+6	-0.145	4%
11	260.39	0.0008	HOMO-6 -> LUMO	0.14731	4%
			HOMO-3 -> LUMO+1	0.15857	5%
			HOMO-2 -> LUMO+1	0.28485	16%
			HOMO-2 -> LUMO+2	-0.11552	3%
			HOMO-1 -> LUMO+1	0.12519	3%
			HOMO-1 -> LUMO+3	-0.13893	4%
			HOMO -> LUMO+2	0.24193	12%
			HOMO -> LUMO+3	0.29383	17%
			HOMO -> LUMO+4	0.12046	3%
			HOMO -> LUMO+5	0.24275	12%
			HOMO -> LUMO+6	0.17878	6%
12	258.46	0.1643	HOMO-3 -> LUMO+1	-0.25348	13%
			HOMO-3 -> LUMO+4	-0.11221	3%
			HOMO-2 -> LUMO+2	0.13702	4%
			HOMO-2 -> LUMO+3	-0.22938	11%
			HOMO-1 -> LUMO+1	0.11796	3%
			HOMO-1 -> LUMO+2	-0.22138	10%
			HOMO-1 -> LUMO+3	0.22098	10%
			HOMO-1 -> LUMO+4	0.1218	3%
			HOMO -> LUMO+2	0.12172	3%
			HOMO -> LUMO+5	0.29992	18%
			HOMO -> LUMO+6	-0.10142	2%
13	252.24	0.0854	HOMO-9 -> LUMO	0.1859	7%
			HOMO-8 -> LUMO	-0.10448	2%
			HOMO-7 -> LUMO	0.48563	47%
			HOMO-6 -> LUMO	-0.20305	8%
			HOMO-3 -> LUMO	0.18785	7%
			HOMO-2 -> LUMO	0.13982	4%
			HOMO-2 -> LUMO+2	-0.10122	2%
			HOMO-1 -> LUMO	-0.13455	4%
14	249.22	0.0303	HOMO-7 -> LUMO	-0.13849	4%
			HOMO-6 -> LUMO	0.1813	7%

			HOMO-3 -> LUMO	0.2263	10%
			HOMO-2 -> LUMO	0.19117	7%
			HOMO-2 -> LUMO+5	0.13833	4%
			HOMO-1 -> LUMO+2	0.26622	14%
			HOMO-1 -> LUMO+4	0.34764	24%
15	248.75	0.5507	HOMO-8 -> LUMO	-0.11313	3%
			HOMO-7 -> LUMO	0.13193	3%
			HOMO-4 -> LUMO+1	0.13835	4%
			HOMO-3 -> LUMO+1	0.2668	14%
			HOMO-2 -> LUMO+1	-0.12295	3%
			HOMO-2 -> LUMO+3	-0.1282	3%
			HOMO-2 -> LUMO+4	-0.19358	7%
			HOMO-1 -> LUMO+2	-0.23493	11%
			HOMO-1 -> LUMO+3	0.21453	9%
			HOMO -> LUMO+3	0.21325	9%
			HOMO -> LUMO+4	-0.12196	3%
			HOMO -> LUMO+5	-0.22407	10%

Table S12. TD-DFT-calculated UV/Vis absorption data for (*P*)-**EH3** at the CAM-B3LYP-D3(BJ)/def2-TZVP (solvent CH_2Cl_2) level.

	Calcd. λ [nm]	Oscillator strength (*f*)	Composition	Coefficient	Contribution
1	380.31	0.2499	HOMO-2 -> LUMO	0.20077	8%
			HOMO -> LUMO	0.60694	74%
			HOMO -> LUMO+1	-0.17224	6%
2	346.16	0.1425	HOMO-1 -> LUMO	-0.22365	10%
			HOMO -> LUMO+1	0.35106	25%
			HOMO -> LUMO+2	0.51654	53%
3	340.88	0.1017	HOMO-3 -> LUMO	-0.17708	6%
			HOMO-2 -> LUMO	0.37791	29%
			HOMO-1 -> LUMO	-0.32285	21%
			HOMO-1 -> LUMO+1	-0.10341	2%
			HOMO-1 -> LUMO+2	-0.15802	5%
			HOMO -> LUMO	-0.10333	2%
			HOMO -> LUMO+1	0.15787	5%
			HOMO -> LUMO+2	-0.30860	19%
			HOMO -> LUMO+3	-0.10076	2%
4	325.01	0.1073	HOMO-4 -> LUMO+1	-0.10778	2%
			HOMO-3 -> LUMO	-0.19222	7%
			HOMO-2 -> LUMO+1	0.18635	7%

			HOMO-2 -> LUMO+2	-0.10058	2%
			HOMO-1 -> LUMO	0.40049	32%
			HOMO-1 -> LUMO+1	-0.23559	11%
			HOMO -> LUMO	0.13522	4%
			HOMO -> LUMO+1	0.33485	22%
5	314.68	0.0034	HOMO-4 -> LUMO	0.10859	2%
			HOMO-3 -> LUMO	-0.19367	8%
			HOMO-3 -> LUMO+2	0.13134	3%
			HOMO-2 -> LUMO	0.11445	3%
			HOMO-2 -> LUMO+1	0.16134	5%
			HOMO-2 -> LUMO+2	0.11239	3%
			HOMO-1 -> LUMO+2	0.27215	15%
			HOMO -> LUMO	-0.15730	5%
			HOMO -> LUMO+3	0.46264	43%
6	305	0.1401	HOMO-4 -> LUMO	-0.24253	12%
			HOMO-3 -> LUMO	0.40370	33%
			HOMO-3 -> LUMO+1	0.16279	5%
			HOMO-2 -> LUMO+2	0.13311	4%
			HOMO-1 -> LUMO	-0.10536	2%
			HOMO-1 -> LUMO+2	0.12042	3%
			HOMO -> LUMO+1	0.24453	12%
			HOMO -> LUMO+2	-0.17479	6%
			HOMO -> LUMO+3	0.14119	4%
			HOMO -> LUMO+4	-0.18536	7%
7	296.53	0.0532	HOMO-4 -> LUMO	0.33768	23%
			HOMO-3 -> LUMO+1	-0.25015	13%
			HOMO-3 -> LUMO+2	0.10664	2%
			HOMO-2 -> LUMO	-0.29666	18%
			HOMO-2 -> LUMO+1	0.16844	6%
			HOMO-1 -> LUMO	-0.12268	3%
			HOMO-1 -> LUMO+2	-0.12068	3%
			HOMO -> LUMO	0.17956	6%
			HOMO -> LUMO+1	0.19637	8%
			HOMO -> LUMO+2	-0.18972	7%
8	289.64	0.2304	HOMO-5 -> LUMO	0.11651	3%
			HOMO-4 -> LUMO+1	0.20871	9%
			HOMO-3 -> LUMO	-0.11471	3%
			HOMO-2 -> LUMO	0.16048	5%
			HOMO-2 -> LUMO+1	-0.25237	13%

			HOMO-2 -> LUMO+2	0.18108	7%
			HOMO-1 -> LUMO	0.25281	13%
			HOMO-1 -> LUMO+1	0.17464	6%
			HOMO-1 -> LUMO+2	-0.21062	9%
			HOMO -> LUMO+4	-0.25051	13%
9	287.08	0.7902	HOMO-5 -> LUMO	0.10502	2%
			HOMO-4 -> LUMO+2	-0.15067	5%
			HOMO-2 -> LUMO+1	-0.10516	2%
			HOMO-1 -> LUMO+1	0.36597	27%
			HOMO-1 -> LUMO+2	0.38589	30%
			HOMO -> LUMO+1	0.17100	6%
			HOMO -> LUMO+2	-0.13883	4%
			HOMO -> LUMO+3	-0.25449	13%
10	276.83	0.3512	HOMO-4 -> LUMO+2	-0.11055	2%
			HOMO-3 -> LUMO+1	-0.10178	2%
			HOMO-2 -> LUMO+1	-0.18349	7%
			HOMO-2 -> LUMO+2	-0.20128	8%
			HOMO-1 -> LUMO+1	0.10752	2%
			HOMO-1 -> LUMO+2	-0.13976	4%
			HOMO -> LUMO+1	0.19320	7%
			HOMO -> LUMO+3	0.31043	19%
			HOMO -> LUMO+4	0.33565	23%
			HOMO -> LUMO+5	0.23743	11%
11	274.62	0.0698	HOMO-5 -> LUMO	-0.11309	3%
			HOMO-4 -> LUMO	0.17813	6%
			HOMO-4 -> LUMO+1	-0.21028	9%
			HOMO-3 -> LUMO	-0.16439	5%
			HOMO-3 -> LUMO+1	0.36779	27%
			HOMO-2 -> LUMO	-0.22462	10%
			HOMO-2 -> LUMO+1	-0.28165	16%
			HOMO-1 -> LUMO+1	0.11304	3%
			HOMO -> LUMO+4	-0.10426	2%
12	266.75	0.0086	HOMO-5 -> LUMO	-0.28368	16%
			HOMO-5 -> LUMO+1	-0.10988	2%
			HOMO-4 -> LUMO	-0.26134	14%
			HOMO-4 -> LUMO+1	-0.17157	6%
			HOMO-3 -> LUMO+1	-0.17288	6%
			HOMO-2 -> LUMO+1	0.18541	7%
			HOMO-1 -> LUMO+1	0.36194	26%

			HOMO-1 -> LUMO+2	-0.18409	7%
13	264.83	0.0473	HOMO-4 -> LUMO+2	0.11337	3%
			HOMO-3 -> LUMO+1	0.13854	4%
			HOMO-2 -> LUMO+2	0.46086	42%
			HOMO-1 -> LUMO+2	-0.10740	2%
			HOMO -> LUMO+4	0.33888	23%
			HOMO -> LUMO+5	0.17773	6%
			HOMO -> LUMO+6	0.10169	2%
14	260.32	0.0268	HOMO-9 -> LUMO	0.14261	4%
			HOMO-6 -> LUMO+2	-0.12171	3%
			HOMO-5 -> LUMO	-0.24407	12%
			HOMO-5 -> LUMO+1	0.15266	5%
			HOMO-4 -> LUMO+1	0.29133	17%
			HOMO-4 -> LUMO+3	-0.10659	2%
			HOMO-3 -> LUMO	-0.18193	7%
			HOMO-3 -> LUMO+3	-0.15566	5%
			HOMO-2 -> LUMO+3	-0.18216	7%
			HOMO-1 -> LUMO+2	0.11077	2%
			HOMO-1 -> LUMO+3	-0.13208	3%
			HOMO-1 -> LUMO+4	0.14892	4%
			HOMO -> LUMO+5	0.15485	5%
			HOMO -> LUMO+6	-0.12618	3%
15	257.59	0.2677	HOMO-9 -> LUMO	-0.13712	4%
			HOMO-5 -> LUMO	0.22251	10%
			HOMO-5 -> LUMO+1	-0.18937	7%
			HOMO-4 -> LUMO+2	0.25486	13%
			HOMO-3 -> LUMO	0.15177	5%
			HOMO-3 -> LUMO+2	0.19203	7%
			HOMO-3 -> LUMO+4	0.12050	3%
			HOMO-2 -> LUMO+3	-0.15555	5%
			HOMO-1 -> LUMO+3	-0.24449	12%
			HOMO -> LUMO+3	-0.10526	2%
			HOMO -> LUMO+5	0.21906	10%

Table S13. Calculated absorption dissymmetry factors (g_{abs}) for the $S_0 \rightarrow S_1$ transition.[a]

Compound	$\|m\|$[b] / 10^{-20} erg G^{-1}	$\|\mu\|$[c] / 10^{-20} esu cm	$\theta_{\mu,m}$[d] / deg	$\|g_{abs,calc}\|$ / 10^{-3}
**EH2**	0.77	403.3	100.1	1.34
**EH3**	1.21	358.4	97.6	1.79

[a] Calculated at the M06-2X/def2-SVP level in the gas phase using the geometry optimized in the S_0 state. [b] Transition magnetic dipole moment for the $S_0 \rightarrow S_1$ transition. [c] Transition electric dipole moment for the $S_0 \rightarrow S_1$ transition. [d] Angle between μ and m for the $S_0 \rightarrow S_1$ transition.

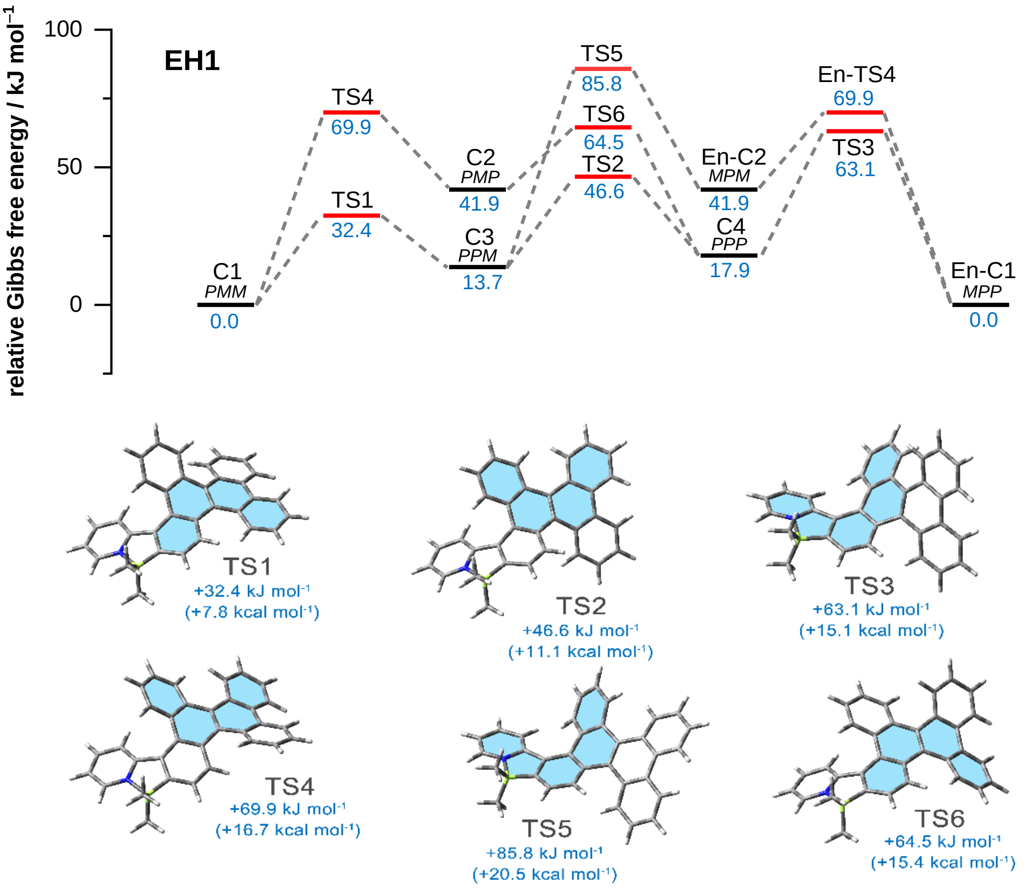

Figure S65. Interconversion pathway of **EH1** calculated at the B3LYP-D3(BJ)/def2-SVP (solvent CH_2Cl_2, PCM model) level (top). The relative Gibbs free energies of the stationary points are given in kJ mol−1. Only one enantiomeric pathway was shown in each case for simplicity. The structures of the transition states (bottom) alongside with their relative Gibbs free energies.

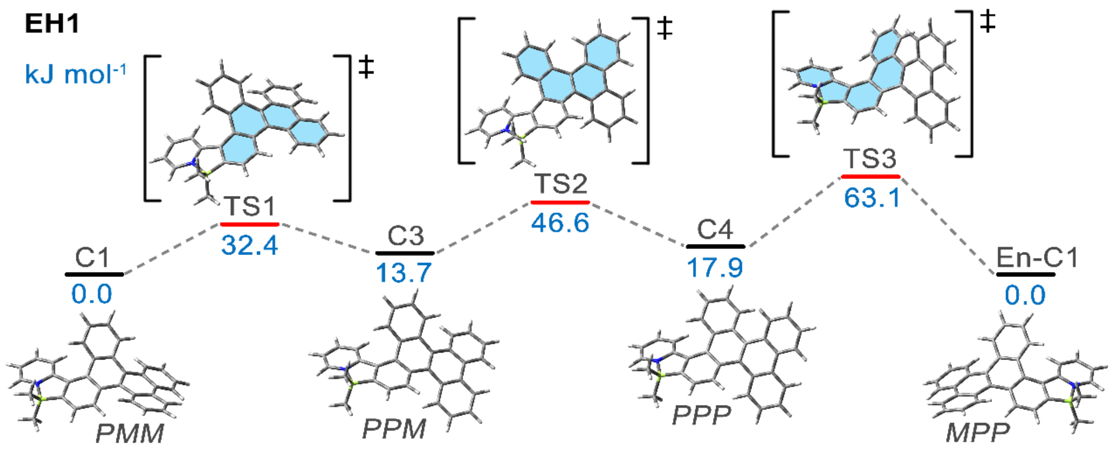

Figure S66. The most plausible interconversion pathway of **EH1**. The relative Gibbs free energies of the stationary points are given in kJ mol−1.

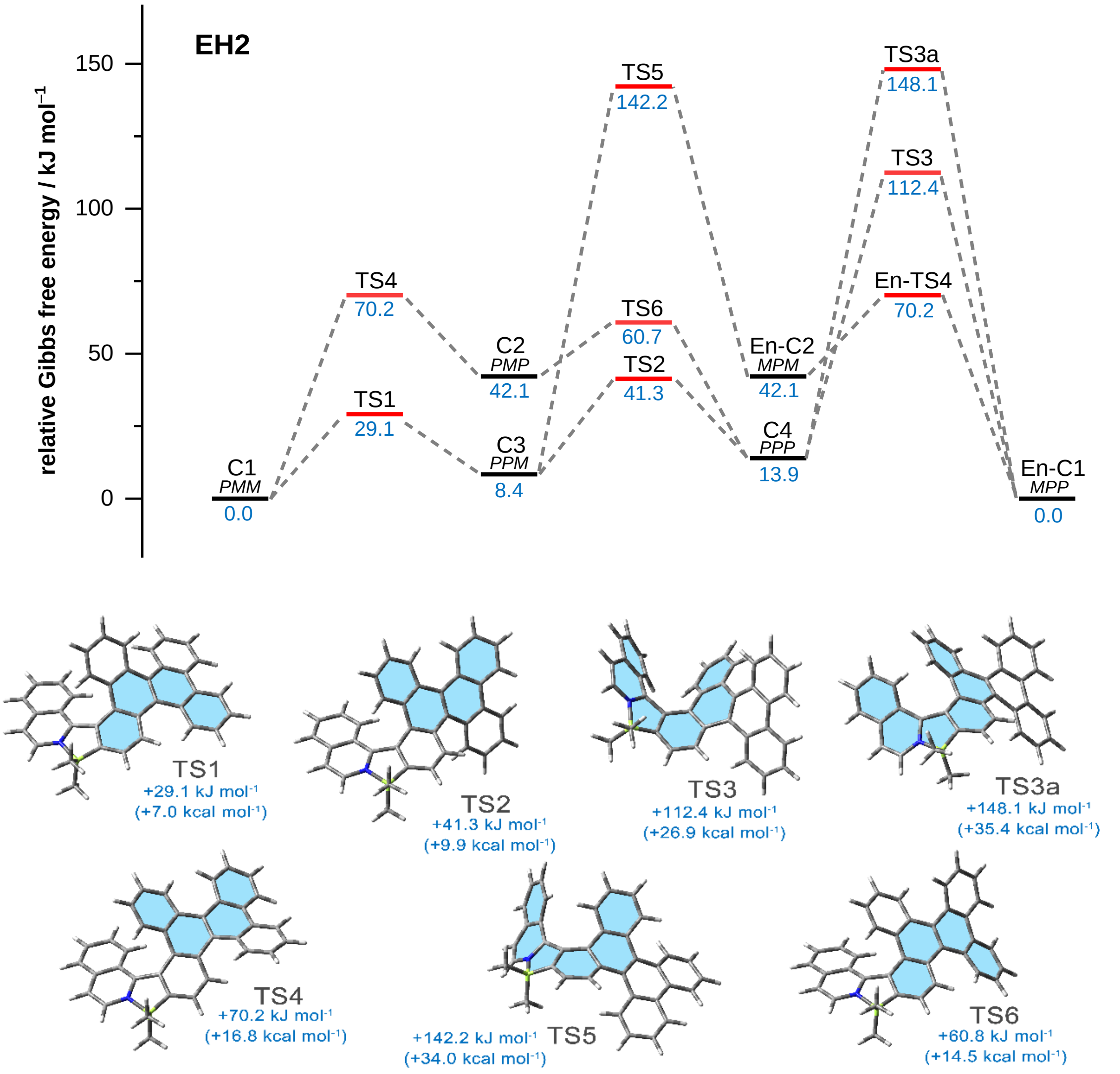

Figure S67. Interconversion pathway of **EH2** calculated at the B3LYP-D3(BJ)/def2-SVP (solvent CH_2Cl_2, PCM model) level (top). The relative Gibbs free energies of the stationary points are given in kJ mol^{-1}. Only one enantiomeric pathway was shown in each case for simplicity. The structures of the transition states (bottom) alongside with their relative Gibbs free energies.

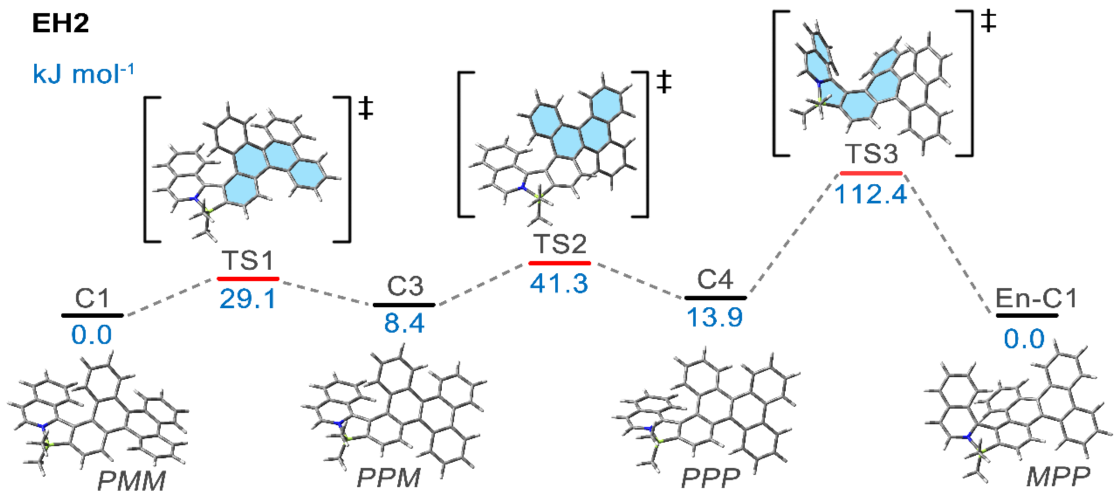

Figure S68. The most plausible interconversion pathway of **EH2**. The relative Gibbs free energies of the stationary points are given in kJ mol^{-1}.

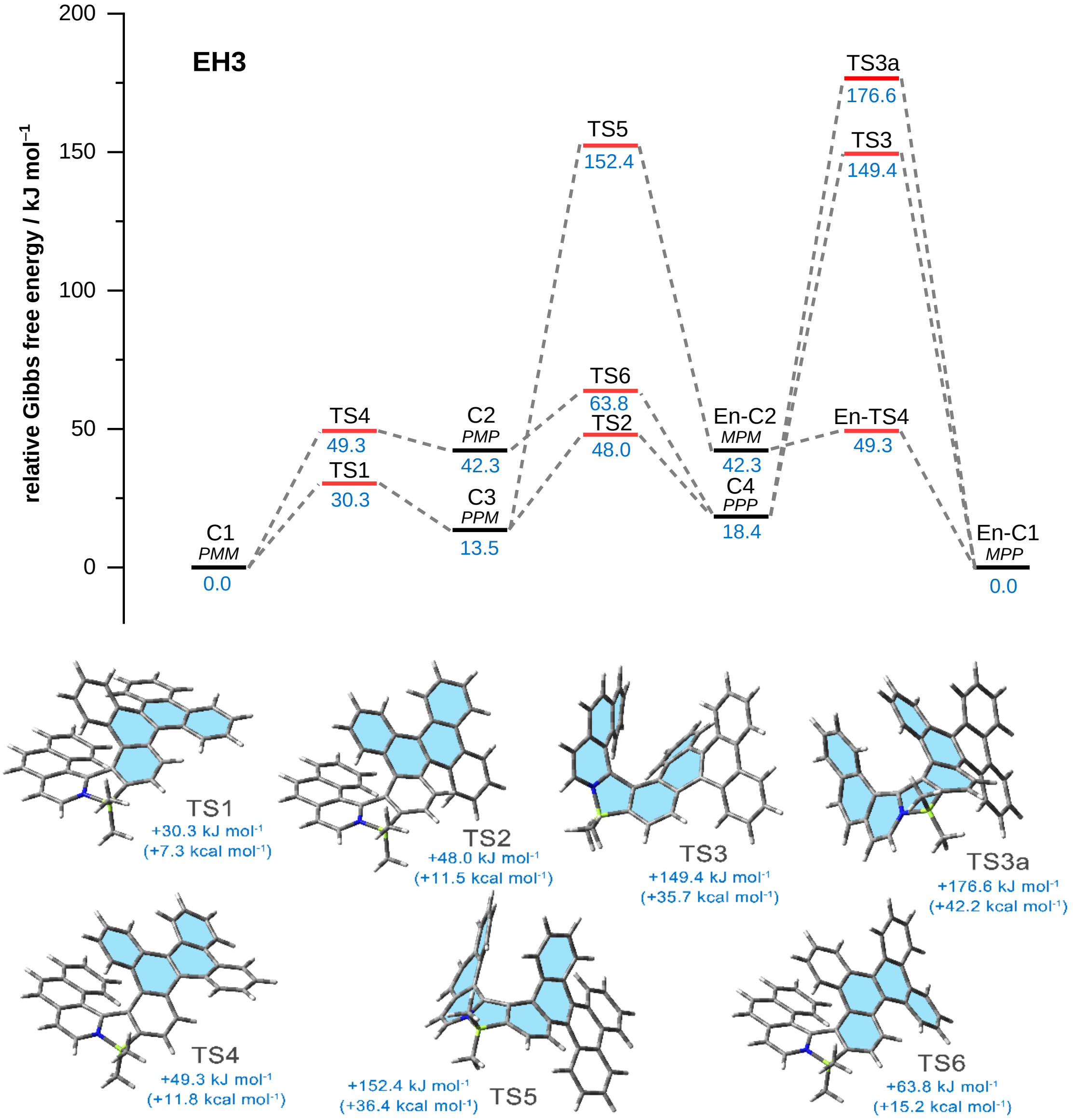

Figure S69. Interconversion pathway of **EH3** calculated at the B3LYP-D3(BJ)/def2-SVP (solvent CH_2Cl_2, PCM model) level (top). The relative Gibbs free energies of the stationary points are given in kJ mol^{-1}. Only one enantiomeric pathway was shown in each case for simplicity. The structures of the transition states (bottom) alongside with their relative Gibbs free energies.

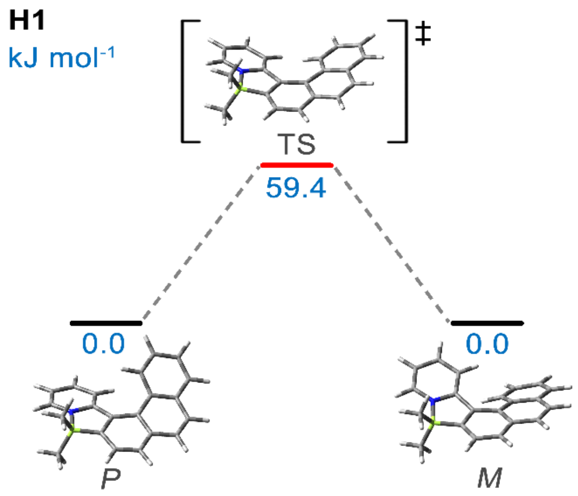

Figure S70. Interconversion pathway of **H1** calculated at the B3LYP-D3(BJ)/def2-SVP (solvent CH_2Cl_2, PCM model) level (top). The relative Gibbs free energies of the stationary points are given in kJ mol^{-1}.

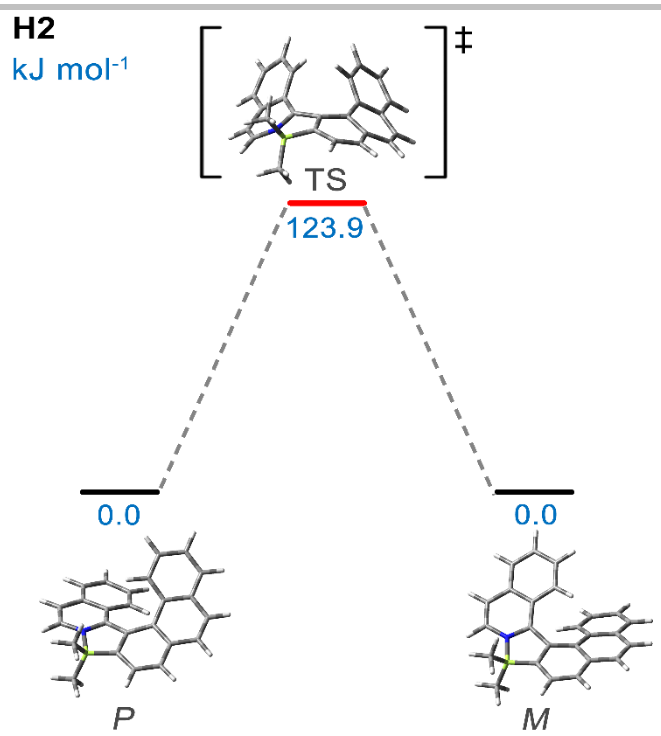

Figure S71. Interconversion pathway of **H2** calculated at the B3LYP-D3(BJ)/def2-SVP (solvent CH_2Cl_2, PCM model) level (top). The relative Gibbs free energies of the stationary points are given in kJ mol^{-1}.

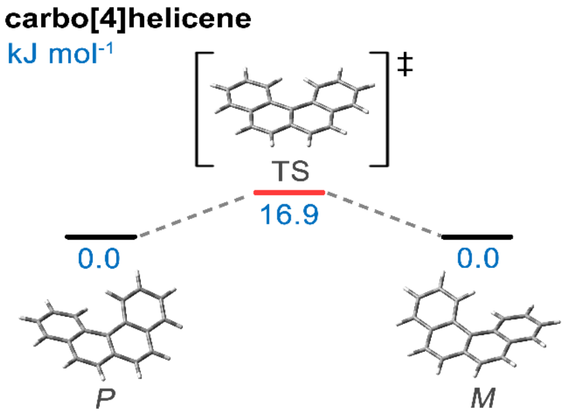

Figure S72. Interconversion pathway of carbo[4]helicene calculated at the B3LYP-D3(BJ)/def2-SVP (solvent CH_2Cl_2, PCM model) level (top). The relative Gibbs free energies of the stationary points are given in kJ mol^{-1}.

Cartesian coordinates (B3LYP-D3(BJ)/def2-TZVP, CH_2Cl_2, PCM model)

EH1

PMM (**C1**)

E = -1352.305789 Hartree

Symbol	X	Y	Z
C	1.596628	2.679003	-0.215243
C	0.269655	2.323417	-0.104431
C	-0.123759	1.006454	0.220820
C	0.883900	0.015393	0.365472
C	-1.831272	-0.741932	0.105048
C	-0.873536	-1.694021	0.639839
C	0.464506	-1.281856	0.859208
C	-1.283138	-2.938668	1.161964
C	-0.421509	-3.732524	1.888335

C	0.881343	-3.296696	2.147944
C	1.304315	-2.083720	1.649725
C	2.220586	0.375455	0.060569
C	3.376348	-0.486954	-0.224115
N	4.491557	0.270150	-0.400050
B	4.177129	1.854135	-0.355747
C	2.597616	1.709775	-0.146950
C	3.456285	-1.861840	-0.455368
C	4.674115	-2.431231	-0.779664
C	5.813943	-1.635589	-0.874656
C	5.673882	-0.275951	-0.693040
C	4.612002	2.507789	-1.776070
C	4.932411	2.522320	0.917842
C	-1.525127	0.609189	0.231725
C	-4.431255	-2.713865	-1.763546
C	-3.273257	-2.391861	-1.090138
C	-3.093885	-1.124503	-0.497187
C	-4.122560	-0.160828	-0.644061
C	-5.294677	-0.515098	-1.334362
C	-5.457977	-1.771643	-1.876834
C	-2.631385	1.547233	0.347931
C	-3.930058	1.158986	-0.070448
C	-2.490602	2.782490	1.015182
C	-3.559891	3.632108	1.197120
C	-4.824392	3.274675	0.721227
C	-5.001539	2.050507	0.113941
H	1.853163	3.709372	-0.432442
H	-0.485477	3.065040	-0.309071
H	-2.311772	-3.245976	1.056419
H	-0.773460	-4.670138	2.298384
H	1.543989	-3.890843	2.763306
H	2.291437	-1.723095	1.898358
H	6.780014	-2.054425	-1.113465
H	6.500513	0.411398	-0.801537
H	4.294071	3.554317	-1.814815
H	5.697432	2.501567	-1.923431
H	4.158817	2.000765	-2.633939
H	4.659431	3.578136	1.007651
H	4.675362	2.038941	1.865612
H	6.022051	2.482150	0.809671
H	-4.532951	-3.688212	-2.223354
H	-2.467715	-3.109063	-1.060928
H	-1.537092	3.048036	1.442990
H	-3.420688	4.564010	1.729319
H	-5.668333	3.937386	0.860798
H	-5.995645	1.760115	-0.192903
H	-6.366560	-2.016505	-2.411098

H	-6.077520	0.216842	-1.469425
H	2.571791	-2.472069	-0.390953
H	4.736313	-3.495560	-0.962734

PMP (**C2**)
E = -1352.289031 Hartree

Symbol	X	Y	Z
C	-1.633378	-2.633872	-0.132821
C	-0.309658	-2.263138	-0.181922
C	0.108673	-0.943640	0.107108
C	-0.884960	0.052208	0.291999
C	1.898527	0.763248	0.061426
C	0.870111	1.787185	0.197019
C	-0.443805	1.398149	0.575886
C	1.079553	3.176462	0.031482
C	0.184479	4.108546	0.510517
C	-0.961484	3.687779	1.194206
C	-1.279811	2.348959	1.188364
C	-2.248071	-0.324578	0.114617
C	-3.435314	0.500074	-0.172029
N	-4.555563	-0.271281	-0.154886
B	-4.219350	-1.841015	0.001949
C	-2.629513	-1.669964	0.025356
C	-3.552242	1.827256	-0.593262
C	-4.799828	2.350593	-0.878460
C	-5.937782	1.557523	-0.747762
C	-5.766133	0.234107	-0.402899
C	-4.797929	-2.624538	-1.295964
C	-4.816322	-2.395997	1.407400
C	1.517091	-0.576874	0.144818
C	5.188083	2.650515	-0.170113
C	3.889001	2.343376	0.174729
C	3.319402	1.080702	-0.096657
C	4.210260	0.059581	-0.524407
C	5.507164	0.410711	-0.940931
C	5.985591	1.694756	-0.803135
C	2.547525	-1.605210	0.210652
C	3.831634	-1.326771	-0.312751
C	2.339685	-2.861377	0.816404
C	3.289896	-3.857315	0.760266
C	4.493312	-3.629887	0.086099
C	4.764280	-2.376304	-0.416561
H	-1.903401	-3.669519	-0.303124
H	0.423291	-3.000569	-0.462485
H	1.953386	3.527301	-0.487842
H	0.389104	5.163196	0.380949
H	-1.616691	4.405109	1.670146
H	-2.202300	2.014556	1.639233

H	-6.926075	1.943685	-0.947330
H	-6.590625	-0.462091	-0.347944
H	-4.463437	-3.666529	-1.278861
H	-5.892830	-2.645260	-1.318737
H	-4.455609	-2.191333	-2.241294
H	-5.911655	-2.367487	1.418950
H	-4.522535	-3.438966	1.559567
H	-4.460786	-1.828782	2.273299
H	5.584577	3.631559	0.056557
H	3.317287	3.084688	0.703306
H	1.426321	-3.040169	1.361468
H	3.104765	-4.807620	1.243422
H	5.233758	-4.415160	0.008495
H	5.737307	-2.191107	-0.845760
H	6.163570	-0.348187	-1.339808
H	6.989642	1.939187	-1.123853
H	-4.886154	3.377169	-1.208526
H	-2.673438	2.436794	-0.707415

PPM (**C3**)
E = -1352.300298 Hartree

Symbol	X	Y	Z
C	1.761607	2.377289	1.082099
C	0.413683	2.106128	0.984155
C	-0.065686	0.872200	0.486438
C	0.880027	-0.162570	0.280092
C	-1.911886	-0.718105	0.154019
C	-0.983024	-1.777758	0.518879
C	0.412092	-1.522161	0.475378
C	-1.415115	-3.018722	1.029513
C	-0.525412	-3.999319	1.409821
C	0.848670	-3.761134	1.323826
C	1.299140	-2.535078	0.889996
C	2.239438	0.225159	0.136003
C	3.336137	-0.492695	-0.513719
N	4.493904	0.207399	-0.390617
B	4.279944	1.620214	0.367825
C	2.696276	1.478027	0.560233
C	3.321680	-1.650178	-1.294537
C	4.495361	-2.093673	-1.874626
C	5.680359	-1.384978	-1.680059
C	5.632964	-0.223604	-0.937914
C	4.726246	2.817999	-0.632373
C	5.102258	1.609622	1.768188
C	-1.472410	0.599970	0.211473
C	-5.029185	-2.562089	-0.904841
C	-3.703771	-2.275939	-0.666159
C	-3.289468	-1.007254	-0.207954

C	-4.258381	0.024144	-0.142353
C	-5.613324	-0.305819	-0.339418
C	-6.000902	-1.579346	-0.691334
C	-2.418345	1.677641	-0.053095
C	-3.810833	1.402347	-0.042844
C	-2.017386	2.994151	-0.368074
C	-2.925145	4.022970	-0.485698
C	-4.287145	3.773025	-0.295536
C	-4.716340	2.478750	-0.105927
H	2.086947	3.314192	1.518477
H	-0.295602	2.837191	1.338801
H	-2.468846	-3.192175	1.175260
H	-0.894011	-4.934022	1.811608
H	1.556442	-4.511020	1.651962
H	2.355482	-2.325409	0.938996
H	6.613137	-1.712659	-2.113934
H	6.502208	0.398236	-0.778899
H	4.482155	3.785581	-0.183105
H	5.804792	2.820227	-0.824050
H	4.218363	2.774296	-1.601128
H	4.893824	2.522141	2.334976
H	4.838284	0.762530	2.408966
H	6.184887	1.576521	1.603162
H	-5.313477	-3.543023	-1.262520
H	-2.963145	-3.035358	-0.859648
H	-0.976834	3.202331	-0.551805
H	-2.580605	5.019039	-0.730781
H	-5.005857	4.580177	-0.348864
H	-5.777376	2.291003	-0.042300
H	-6.370879	0.458554	-0.255958
H	-7.048175	-1.802273	-0.847613
H	4.490421	-2.988259	-2.482707
H	2.397724	-2.184694	-1.445617

PPP (**C4**)
E = -1352.298778 Hartree

Symbol	X	Y	Z
C	-1.616258	-2.288631	1.253241
C	-0.305261	-2.043730	0.917986
C	0.094173	-0.818236	0.332209
C	-0.867194	0.209486	0.181070
C	1.960593	0.744088	0.034187
C	1.003246	1.826890	-0.086331
C	-0.389957	1.570899	0.049785
C	1.396870	3.143520	-0.418157
C	0.505457	4.191565	-0.418650
C	-0.823006	3.967083	-0.040926
C	-1.253316	2.678915	0.174956

C	-2.237334	-0.190263	0.250310
C	-3.427026	0.422365	-0.352312
N	-4.547058	-0.244705	0.033529
B	-4.207673	-1.548364	0.922761
C	-2.615522	-1.398761	0.846055
C	-3.542346	1.417241	-1.326898
C	-4.791230	1.761131	-1.809240
C	-5.928343	1.115856	-1.325873
C	-5.758102	0.097045	-0.412731
C	-4.793915	-1.355318	2.425209
C	-4.797537	-2.857533	0.166310
C	1.489562	-0.563597	0.025753
C	5.224584	2.297609	1.136549
C	3.887323	2.138818	0.844167
C	3.387802	0.955362	0.258353
C	4.297384	-0.113906	0.057900
C	5.658929	0.076631	0.352702
C	6.126935	1.266523	0.865297
C	2.397684	-1.633153	-0.365725
C	3.792806	-1.391565	-0.410236
C	1.919406	-2.856567	-0.882700
C	2.775213	-3.821705	-1.366212
C	4.154202	-3.594935	-1.366627
C	4.645721	-2.391921	-0.908711
H	-1.875915	-3.201582	1.775753
H	0.452611	-2.771743	1.165658
H	2.419799	3.331114	-0.700961
H	0.841059	5.185641	-0.683309
H	-1.513294	4.793871	0.062563
H	-2.277727	2.515235	0.465616
H	-6.917468	1.372393	-1.674453
H	-6.585011	-0.488098	-0.036774
H	-4.491921	-2.193509	3.060465
H	-5.889268	-1.328525	2.430905
H	-4.436976	-0.439013	2.905577
H	-4.468204	-3.763493	0.684515
H	-4.458542	-2.937904	-0.871525
H	-5.892726	-2.877549	0.159149
H	5.565832	3.213640	1.600932
H	3.204446	2.922656	1.126400
H	0.856738	-3.026274	-0.948761
H	2.373509	-4.742289	-1.768978
H	4.831604	-4.345205	-1.752869
H	5.708734	-2.209017	-0.963697
H	7.178245	1.383681	1.093071
H	6.353479	-0.738197	0.211419
H	-2.659298	1.903475	-1.706822

H	-4.881199	2.528213	-2.566638

EH2
PMM (**C1**)
E = -1506.015593 Hartree

Symbol	X	Y	Z
C	0.768350	3.184314	-0.458652
C	-0.491968	2.629919	-0.376060
C	-0.693284	1.311640	0.090064
C	0.450115	0.508632	0.350496
C	-2.112887	-0.675954	0.254483
C	-1.028568	-1.378845	0.916871
C	0.231486	-0.745097	1.040521
C	-1.241361	-2.582103	1.621256
C	-0.260114	-3.129545	2.420044
C	0.969579	-2.480022	2.565857
C	1.200297	-1.301703	1.891891
C	1.724114	1.032048	0.013553
C	3.023561	0.362150	-0.132716
N	4.008912	1.274125	-0.118096
B	3.454210	2.783903	-0.257914
C	1.902296	2.399201	-0.250958
C	3.347117	-0.999247	-0.422013
C	4.722812	-1.375083	-0.409419
C	5.705203	-0.373123	-0.217961
C	5.317095	0.927933	-0.137579
C	2.387733	-1.963229	-0.805073
C	2.762269	-3.252863	-1.080760
C	4.111195	-3.644264	-0.974002
C	5.073732	-2.721573	-0.655168
C	4.009532	3.387749	-1.659688
C	3.880286	3.667136	1.036557
C	-2.017424	0.710086	0.167158
C	-4.342399	-3.287066	-1.278921
C	-3.262853	-2.692347	-0.663315
C	-3.290131	-1.335928	-0.276344
C	-4.448570	-0.575057	-0.572123
C	-5.536384	-1.205667	-1.200373
C	-5.495560	-2.540918	-1.540064
C	-3.257722	1.472206	0.147586
C	-4.472075	0.829936	-0.207084
C	-3.325241	2.797422	0.627457
C	-4.516429	3.487919	0.681645
C	-5.699205	2.873991	0.261207
C	-5.671618	1.561678	-0.157998
H	0.873200	4.224971	-0.742663
H	-1.340636	3.218975	-0.683270
H	-2.212947	-3.050133	1.591795
H	-0.461995	-4.040717	2.967905

H	1.726836	-2.885622	3.223606
H	2.134095	-0.779520	2.038269
H	6.753867	-0.633547	-0.203368
H	6.019017	1.746954	-0.089905
H	4.388741	-4.671046	-1.172913
H	6.118137	-3.002755	-0.616822
H	3.549899	4.363329	-1.846556
H	5.093405	3.544126	-1.646424
H	3.777344	2.750258	-2.518605
H	3.436888	4.665659	0.976816
H	3.551293	3.222144	1.980775
H	4.965860	3.803512	1.094735
H	-4.285264	-4.324911	-1.580141
H	-2.359896	-3.265507	-0.521752
H	-2.435925	3.266681	1.016157
H	-4.534497	4.496760	1.072759
H	-6.637894	3.410841	0.300384
H	-6.601835	1.078228	-0.418056
H	-6.343369	-3.000632	-2.030771
H	-6.416735	-0.631240	-1.449057
H	2.016470	-3.974871	-1.384727
H	1.354635	-1.676291	-0.904180

PMP (**C2**)
E = -1505.998917 Hartree

Symbol	X	Y	Z
C	-0.808348	3.153001	0.270555
C	0.438908	2.574338	0.356404
C	0.673326	1.241393	-0.058222
C	-0.451423	0.435573	-0.367221
C	2.176402	-0.716546	-0.245093
C	1.010731	-1.540282	-0.543698
C	-0.221020	-0.903086	-0.850198
C	0.999364	-2.954404	-0.587117
C	-0.028991	-3.655088	-1.180323
C	-1.097017	-2.969518	-1.767729
C	-1.203510	-1.614075	-1.562902
C	-1.746099	0.990913	-0.165710
C	-3.060037	0.350070	-0.012389
N	-4.033223	1.253646	-0.211419
B	-3.470273	2.768431	-0.189179
C	-1.928351	2.373619	-0.022837
C	-3.415814	-0.945719	0.475529
C	-4.792010	-1.316305	0.442098
C	-5.750165	-0.358277	0.026767
C	-5.344086	0.914843	-0.224768
C	-2.492611	-1.826852	1.081513
C	-2.899423	-3.044736	1.561342

C	-4.242561	-3.449112	1.437655
C	-5.172289	-2.599134	0.895615
C	-4.133585	3.519091	1.088825
C	-3.767496	3.515654	-1.599551
C	2.009062	0.665396	-0.126537
C	5.126892	-3.108442	-0.271704
C	3.906114	-2.549245	-0.584672
C	3.524437	-1.273406	-0.117388
C	4.540088	-0.486996	0.489525
C	5.748651	-1.100159	0.866316
C	6.031420	-2.404564	0.526284
C	3.189911	1.513265	-0.012630
C	4.387301	0.957720	0.496541
C	3.213965	2.860487	-0.427325
C	4.302086	3.671634	-0.189125
C	5.415357	3.158523	0.482565
C	5.462221	1.817083	0.792641
H	-0.924168	4.205396	0.502095
H	1.254914	3.162072	0.740785
H	1.802979	-3.511209	-0.139542
H	0.007682	-4.736245	-1.207042
H	-1.862174	-3.503129	-2.315259
H	-2.070194	-1.082305	-1.926479
H	-6.798502	-0.617310	-0.013410
H	-6.033188	1.719127	-0.434993
H	-4.544277	-4.423831	1.798050
H	-6.215083	-2.883446	0.839802
H	-3.670494	4.502658	1.216304
H	-5.208299	3.687736	0.963139
H	-3.991789	2.970405	2.025286
H	-4.842357	3.645790	-1.767717
H	-3.321838	4.514953	-1.602296
H	-3.360491	2.976280	-2.460438
H	5.379734	-4.089768	-0.651285
H	3.251465	-3.095234	-1.240035
H	2.373779	3.260729	-0.972015
H	4.293557	4.698972	-0.528661
H	6.261609	3.795043	0.705305
H	6.369664	1.416580	1.218705
H	6.495741	-0.527889	1.395657
H	6.971676	-2.852155	0.820060
H	-2.181855	-3.701690	2.033769
H	-1.462557	-1.529133	1.187827

PPM (**C3**)
E = -1506.012322 Hartree

Symbol	X	Y	Z
C	0.974906	3.063945	0.838364

C	-0.316641	2.604845	0.686481
C	-0.593745	1.246548	0.413640
C	0.489315	0.332952	0.446312
C	-2.196435	-0.617339	0.398825
C	-1.156054	-1.430291	1.012520
C	0.193410	-1.002639	0.930799
C	-1.442548	-2.578093	1.779196
C	-0.446654	-3.325937	2.368267
C	0.888378	-2.935418	2.232723
C	1.192079	-1.782169	1.546473
C	1.796363	0.871755	0.286148
C	3.043252	0.225461	-0.128346
N	4.085279	1.034879	0.122353
B	3.627797	2.509572	0.585292
C	2.054824	2.222986	0.560952
C	3.263182	-0.993500	-0.840474
C	4.610821	-1.403174	-1.063850
C	5.663445	-0.550828	-0.650711
C	5.364494	0.662606	-0.113571
C	2.219564	-1.773143	-1.387048
C	2.493570	-2.934941	-2.061854
C	3.822434	-3.376394	-2.215927
C	4.860570	-2.620958	-1.735368
C	4.146339	3.570727	-0.530164
C	4.182641	2.825078	2.078843
C	-1.935112	0.728090	0.166951
C	-4.985512	-3.063396	-0.291798
C	-3.724214	-2.551419	-0.085673
C	-3.502324	-1.167140	0.076055
C	-4.596667	-0.290574	-0.129236
C	-5.883983	-0.839865	-0.286758
C	-6.084955	-2.200786	-0.344391
C	-2.993234	1.581749	-0.356692
C	-4.336465	1.123843	-0.332487
C	-2.743801	2.844894	-0.935957
C	-3.766268	3.687917	-1.309873
C	-5.094590	3.294883	-1.122034
C	-5.366190	2.024355	-0.666628
H	1.145293	4.090957	1.138600
H	-1.139033	3.283786	0.849110
H	-2.469775	-2.856238	1.950745
H	-0.704552	-4.192922	2.962258
H	1.676426	-3.503199	2.709647
H	2.213963	-1.438637	1.534798
H	6.692589	-0.840618	-0.807646
H	6.119572	1.387863	0.150266
H	4.021939	-4.303523	-2.736950
H	5.886197	-2.933527	-1.882615
H	3.750907	4.565505	-0.302839

H	5.237993	3.657661	-0.546030
H	3.819894	3.315730	-1.543319
H	3.799450	3.788459	2.428534
H	3.880564	2.070355	2.811516
H	5.276060	2.890185	2.100562
H	-5.120852	-4.129506	-0.418774
H	-2.883327	-3.226152	-0.073770
H	-1.728074	3.153101	-1.119409
H	-3.537356	4.648187	-1.753088
H	-5.905818	3.963095	-1.379551
H	-6.396488	1.709019	-0.601642
H	-6.735940	-0.187986	-0.406387
H	-7.083929	-2.595000	-0.476980
H	1.683625	-3.514690	-2.483593
H	1.199554	-1.441692	-1.288641

PPP (**C4**)
E = -1506.010324 Hartree

Symbol	X	Y	Z
C	-0.808521	2.944958	-1.029564
C	0.425372	2.482523	-0.631214
C	0.629033	1.124156	-0.289465
C	-0.454712	0.222539	-0.405286
C	2.272560	-0.694693	-0.287963
C	1.187935	-1.642587	-0.467848
C	-0.153511	-1.183040	-0.582118
C	1.398805	-3.040063	-0.445721
C	0.386515	-3.933394	-0.713196
C	-0.884897	-3.457716	-1.050127
C	-1.143298	-2.110026	-0.967300
C	-1.758419	0.803790	-0.468391
C	-3.073213	0.257606	-0.118947
N	-4.045019	1.033822	-0.626585
B	-3.476708	2.431434	-1.195087
C	-1.934273	2.127162	-0.896570
C	-3.428711	-0.798195	0.776346
C	-4.807129	-1.143805	0.889205
C	-5.765930	-0.374239	0.185864
C	-5.356591	0.723527	-0.505328
C	-2.494886	-1.454496	1.608795
C	-2.896756	-2.451628	2.459734
C	-4.247404	-2.848781	2.508440
C	-5.185287	-2.202394	1.745256
C	-3.786371	2.570573	-2.783106
C	-4.123383	3.628231	-0.307206
C	1.959233	0.626191	0.009837
C	5.407373	-2.364957	-1.578820
C	4.079414	-2.103146	-1.320177
C	3.678212	-1.032677	-0.491708

C	4.688700	-0.167759	0.000573
C	6.037257	-0.463470	-0.266163
C	6.400793	-1.553671	-1.025642
C	2.954323	1.453653	0.676057
C	4.303478	1.025797	0.730716
C	2.584910	2.587078	1.432019
C	3.508536	3.299356	2.164871
C	4.846194	2.894672	2.184062
C	5.226673	1.767959	1.488044
H	-0.908897	3.957866	-1.400736
H	1.279030	3.142793	-0.666528
H	2.373009	-3.422058	-0.188436
H	0.584195	-4.996833	-0.682476
H	-1.668517	-4.142653	-1.345598
H	-2.131550	-1.754701	-1.207876
H	-6.816159	-0.620114	0.250691
H	-6.044983	1.404507	-0.983098
H	-4.546360	-3.650551	3.170748
H	-6.231194	-2.473546	1.807535
H	-3.326752	3.480076	-3.181527
H	-4.861635	2.644536	-2.979862
H	-3.399494	1.729029	-3.365946
H	-3.659340	4.580465	-0.582339
H	-3.969433	3.491214	0.767812
H	-5.199812	3.738188	-0.476169
H	5.673690	-3.184919	-2.232989
H	3.333228	-2.702424	-1.814566
H	1.547864	2.878734	1.479936
H	3.189167	4.155080	2.745190
H	5.574942	3.443744	2.765790
H	6.252923	1.437608	1.552770
H	7.445554	-1.752820	-1.224835
H	6.809415	0.198008	0.097945
H	-2.171780	-2.936155	3.099680
H	-1.460568	-1.153187	1.588657

EH3
PMM (**C1**)
E = -1659.730665 Hartree

Symbol	X	Y	Z
C	0.471919	3.497565	-0.624094
C	-0.756906	2.895274	-0.447095
C	-0.877594	1.612180	0.133748
C	0.312910	0.898523	0.431924
C	-2.160957	-0.446444	0.453404
C	-1.031421	-1.031193	1.150481
C	0.188345	-0.316596	1.205622
C	-1.156666	-2.200734	1.926555
C	-0.122535	-2.647055	2.721009
C	1.070207	-1.920540	2.794328

C	1.210033	-0.765937	2.057106
C	1.543982	1.442856	-0.006492
C	2.867119	0.811925	-0.118300
N	3.811429	1.778553	-0.152665
B	3.183685	3.236993	-0.470418
C	1.650759	2.786096	-0.394038
C	3.267175	-0.547332	-0.251568
C	4.627165	-0.836330	0.037797
C	5.549974	0.221909	0.163001
C	5.120833	1.504207	-0.028280
C	2.411652	-1.629668	-0.691677
C	2.863169	-2.966794	-0.524631
C	4.181432	-3.212346	-0.033384
C	5.047941	-2.193579	0.180499
C	1.194440	-1.428582	-1.368966
C	0.415706	-2.489715	-1.771363
C	0.819393	-3.806654	-1.514014
C	2.034799	-4.037335	-0.912651
C	3.705770	3.678128	-1.943902
C	3.577975	4.294276	0.695683
C	-2.159359	0.933717	0.268513
C	-4.189721	-3.300906	-0.910858
C	-3.158851	-2.593035	-0.332895
C	-3.286232	-1.220277	-0.033003
C	-4.493237	-0.563570	-0.379024
C	-5.530324	-1.309174	-0.965904
C	-5.392067	-2.657066	-1.219602
C	-3.447767	1.605942	0.194247
C	-4.614573	0.858369	-0.111182
C	-3.604921	2.956282	0.571346
C	-4.840272	3.566773	0.576457
C	-5.978032	2.843861	0.207850
C	-5.860970	1.508766	-0.112870
H	0.515366	4.513969	-0.997961
H	-1.642718	3.415787	-0.772932
H	-2.098596	-2.726482	1.947887
H	-0.253403	-3.538353	3.320746
H	1.868679	-2.246573	3.447614
H	2.112223	-0.180812	2.152675
H	6.593525	0.014109	0.350418
H	5.790150	2.351057	-0.060598
H	4.497153	-4.239280	0.101973
H	6.073434	-2.385868	0.467598
H	0.866671	-0.425992	-1.590197
H	-0.517246	-2.300976	-2.285512
H	0.191017	-4.635247	-1.812800
H	2.385802	-5.049026	-0.752437
H	3.213452	4.607720	-2.246487
H	4.784404	3.867840	-1.961976

H	3.488131	2.930172	-2.712840
H	3.084020	5.254396	0.517760
H	3.279955	3.957067	1.693382
H	4.655368	4.492072	0.721108
H	-4.056949	-4.348848	-1.146269
H	-2.217064	-3.087997	-0.154731
H	-2.749268	3.512277	0.919620
H	-4.927548	4.599067	0.889241
H	-6.950998	3.317386	0.209999
H	-6.755644	0.945497	-0.334538
H	-6.202238	-3.206578	-1.680700
H	-6.447859	-0.815947	-1.251958

PMP (**C2**)
E = -1659.713823 Hartree

Symbol	X	Y	Z
C	-0.405901	3.419550	0.545661
C	0.794947	2.746192	0.547051
C	0.928813	1.462922	-0.033912
C	-0.251024	0.781614	-0.427388
C	2.288861	-0.563128	-0.431198
C	1.063963	-1.273753	-0.772201
C	-0.120165	-0.528356	-1.014747
C	0.950607	-2.676565	-0.901897
C	-0.139729	-3.264243	-1.503524
C	-1.161066	-2.467154	-2.031369
C	-1.157973	-1.120569	-1.755871
C	-1.502617	1.403566	-0.152843
C	-2.873899	0.859819	-0.120525
N	-3.748351	1.878261	-0.294173
B	-3.069857	3.316038	-0.006650
C	-1.577095	2.769769	0.153483
C	-3.393862	-0.439594	0.150567
C	-4.740156	-0.675345	-0.238136
C	-5.558450	0.408577	-0.614822
C	-5.053394	1.674065	-0.536840
C	-2.681496	-1.498018	0.836251
C	-3.247265	-2.802508	0.857620
C	-4.527973	-3.029884	0.270422
C	-5.265744	-2.002802	-0.212905
C	-1.499674	-1.286018	1.570499
C	-0.865397	-2.320838	2.220076
C	-1.385784	-3.620258	2.170323
C	-2.568486	-3.849945	1.507510
C	-3.745584	3.905123	1.348030
C	-3.230001	4.300433	-1.286792
C	2.221168	0.808367	-0.178990
C	5.056434	-3.138830	-0.776232
C	3.869592	-2.471054	-0.990468

C	3.595596	-1.220914	-0.395460
C	4.680674	-0.566857	0.246818
C	5.854778	-1.293062	0.517426
C	6.034035	-2.574918	0.046693
C	3.458732	1.559516	-0.008287
C	4.627200	0.876348	0.404585
C	3.563960	2.939692	-0.277271
C	4.709053	3.648639	0.013337
C	5.800620	2.995247	0.592776
C	5.763842	1.628120	0.757942
H	-0.446068	4.440734	0.906533
H	1.653888	3.218930	0.992338
H	1.718519	-3.314301	-0.501224
H	-0.190778	-4.342028	-1.583570
H	-1.974899	-2.909737	-2.589823
H	-1.983029	-0.506335	-2.082094
H	-6.590137	0.242793	-0.889403
H	-5.654683	2.559764	-0.679195
H	-4.925342	-4.037217	0.275105
H	-6.270069	-2.163889	-0.582053
H	-1.087742	-0.293273	1.643694
H	0.044066	-2.123995	2.772139
H	-0.871777	-4.430881	2.669658
H	-3.007652	-4.839628	1.493280
H	-3.226278	4.819998	1.650522
H	-4.799138	4.168666	1.206101
H	-3.689290	3.206591	2.188869
H	-4.278846	4.556618	-1.473705
H	-2.700731	5.241833	-1.110562
H	-2.825619	3.868891	-2.207721
H	5.226672	-4.095638	-1.252254
H	3.154607	-2.908253	-1.664538
H	2.739384	3.450181	-0.748861
H	4.761482	4.705288	-0.213732
H	6.692467	3.548226	0.856896
H	6.650966	1.126862	1.114598
H	6.657177	-0.825829	1.068403
H	6.949773	-3.110084	0.260707

PPM (**C3**)
E = -1659.724981 Hartree

Symbol	X	Y	Z
C	0.782026	3.512901	0.548947
C	-0.492870	2.987872	0.536468
C	-0.720576	1.593524	0.461983
C	0.405974	0.743437	0.578376
C	-2.208232	-0.354089	0.650664
C	-1.124868	-1.041584	1.334794
C	0.194833	-0.544280	1.204831

C	-1.341733	-2.133978	2.198132
C	-0.300541	-2.767244	2.840897
C	1.007922	-2.307917	2.665117
C	1.239804	-1.200835	1.881650
C	1.674928	1.299997	0.267111
C	2.930793	0.629546	-0.090583
N	3.952077	1.504450	0.048526
B	3.445281	3.025769	0.244526
C	1.886503	2.683306	0.330938
C	3.203094	-0.687725	-0.558462
C	4.550217	-1.127051	-0.462123
C	5.568456	-0.190902	-0.190425
C	5.238473	1.122680	-0.020964
C	2.220917	-1.599004	-1.110727
C	2.557544	-2.974834	-1.224899
C	3.889063	-3.403921	-0.937746
C	4.860674	-2.509932	-0.636606
C	0.976704	-1.186037	-1.622924
C	0.062913	-2.095120	-2.105072
C	0.357259	-3.465803	-2.108583
C	1.596953	-3.892618	-1.693827
C	3.891970	3.867021	-1.072110
C	4.029670	3.639903	1.628688
C	-2.034255	0.980055	0.295877
C	-4.802821	-3.043202	0.145853
C	-3.584479	-2.425146	0.316137
C	-3.468514	-1.019949	0.373593
C	-4.621768	-0.244339	0.101564
C	-5.863971	-0.897667	-0.017085
C	-5.962614	-2.270487	0.026417
C	-3.152275	1.710224	-0.290085
C	-4.461371	1.165004	-0.210774
C	-2.998495	2.931022	-0.982676
C	-4.080732	3.667602	-1.410140
C	-5.376796	3.205621	-1.164605
C	-5.554390	1.962505	-0.600318
H	0.919494	4.577754	0.694369
H	-1.335535	3.651042	0.652087
H	-2.350149	-2.459758	2.396756
H	-0.503299	-3.595903	3.506705
H	1.829558	-2.783815	3.184083
H	2.238127	-0.796909	1.834199
H	6.601211	-0.506310	-0.153930
H	5.974034	1.903456	0.103120
H	4.121058	-4.457678	-1.028608
H	5.886643	-2.826792	-0.503048
H	0.733010	-0.137238	-1.649814

H	-0.888718	-1.745196	-2.482585
H	-0.374406	-4.177388	-2.467590
H	1.863584	-4.940761	-1.745987
H	3.477152	4.878580	-1.023306
H	4.979935	3.970648	-1.146293
H	3.538236	3.415272	-2.004264
H	3.610924	4.635225	1.806102
H	3.785803	3.026048	2.501376
H	5.118767	3.755072	1.598359
H	-4.858372	-4.122866	0.097698
H	-2.694310	-3.030574	0.379602
H	-2.009928	3.289295	-1.214351
H	-3.922558	4.598132	-1.939377
H	-6.234915	3.793914	-1.462006
H	-6.559380	1.584069	-0.490626
H	-6.760737	-0.321974	-0.189130
H	-6.928510	-2.746686	-0.079683

PPP (**C4**)
E = -1659.723584 Hartree

Symbol	X	Y	Z
C	-0.484238	3.419642	-0.591690
C	0.722808	2.814866	-0.330706
C	0.842936	1.405178	-0.273373
C	-0.294760	0.611857	-0.552875
C	2.363383	-0.491317	-0.592161
C	1.220553	-1.322269	-0.918357
C	-0.085109	-0.760503	-0.959125
C	1.338417	-2.718392	-1.102057
C	0.268409	-3.493919	-1.484795
C	-0.965243	-2.888540	-1.746765
C	-1.130071	-1.551402	-1.476539
C	-1.562590	1.266218	-0.461142
C	-2.916507	0.729412	-0.238590
N	-3.825551	1.657071	-0.619701
B	-3.170672	3.114732	-0.820771
C	-1.656533	2.657566	-0.602746
C	-3.384916	-0.484169	0.345526
C	-4.741632	-0.826020	0.097422
C	-5.612815	0.134150	-0.454160
C	-5.137024	1.385547	-0.718159
C	-2.593507	-1.375061	1.169117
C	-3.090486	-2.679294	1.438215
C	-4.401006	-3.042036	1.003885
C	-5.215232	-2.135594	0.413740
C	-1.388942	-0.994114	1.788888
C	-0.655617	-1.886043	2.537791
C	-1.100900	-3.203487	2.713434

C	-2.312562	-3.583721	2.185993
C	-3.443343	3.664218	-2.323775
C	-3.770680	4.084712	0.337153
C	2.135913	0.778111	-0.073935
C	5.382059	-2.130377	-2.164867
C	4.073869	-1.829440	-1.853443
C	3.743648	-0.884209	-0.858225
C	4.807823	-0.180947	-0.238772
C	6.134339	-0.513744	-0.565285
C	6.425988	-1.484888	-1.497849
C	3.179821	1.406125	0.724274
C	4.498847	0.890513	0.690039
C	2.881470	2.402461	1.678780
C	3.846796	2.911081	2.519746
C	5.156817	2.429270	2.450670
C	5.466096	1.425435	1.558830
H	-0.528290	4.491154	-0.745801
H	1.616108	3.416301	-0.253451
H	2.284214	-3.197205	-0.907683
H	0.391257	-4.562571	-1.602291
H	-1.793207	-3.469471	-2.130767
H	-2.088434	-1.098531	-1.665652
H	-6.653889	-0.104525	-0.616870
H	-5.767854	2.200550	-1.040564
H	-4.753320	-4.044674	1.211499
H	-6.236340	-2.386989	0.158834
H	-1.034308	0.017557	1.687866
H	0.269878	-1.562686	2.995591
H	-0.509442	-3.904928	3.286756
H	-2.698508	-4.580752	2.357477
H	-2.922360	4.613617	-2.480536
H	-4.507789	3.853438	-2.501514
H	-3.096162	2.972485	-3.097578
H	-3.263342	5.053981	0.305909
H	-3.633385	3.678301	1.344182
H	-4.839998	4.279402	0.201610
H	5.594938	-2.848639	-2.945902
H	3.289343	-2.291928	-2.429042
H	1.864644	2.743080	1.791079
H	3.580113	3.663887	3.249986
H	5.918047	2.817902	3.114349
H	6.468998	1.024654	1.552945
H	7.455595	-1.714886	-1.738508
H	6.948028	0.027045	-0.104837

Cartesian coordinates (B3LYP-D3(BJ)/def2-SVP, CH_2Cl_2, PCM model)
Inversion of EH1
EH1
PMM (**C1**)
E = -1350.872062 Hartree

Symbol	X	Y	Z
C	1.607612	2.694736	-0.209684
C	0.272891	2.337636	-0.100767
C	-0.124862	1.012364	0.219269
C	0.887966	0.014811	0.361622
C	-1.841100	-0.745981	0.105078
C	-0.878026	-1.703082	0.638831
C	0.468183	-1.288634	0.856048
C	-1.285207	-2.958335	1.157879
C	-0.413559	-3.762558	1.876288
C	0.896914	-3.325448	2.133101
C	1.317653	-2.100371	1.640051
C	2.232373	0.376899	0.057057
C	3.393418	-0.486071	-0.231053
N	4.513459	0.272891	-0.402250
B	4.198776	1.860525	-0.348978
C	2.612640	1.718864	-0.144208
C	3.473917	-1.868649	-0.466675
C	4.700304	-2.440253	-0.789412
C	5.845704	-1.638542	-0.879949
C	5.701657	-0.271278	-0.694376
C	4.640336	2.523812	-1.767776
C	4.953711	2.516842	0.936618
C	-1.532586	0.614197	0.232750
C	-4.471730	-2.734726	-1.744889
C	-3.299212	-2.406821	-1.082833
C	-3.111937	-1.130254	-0.494349
C	-4.147785	-0.161638	-0.636359
C	-5.334474	-0.522408	-1.313266
C	-5.505500	-1.788857	-1.850453
C	-2.642964	1.557103	0.349155
C	-3.949961	1.167019	-0.069188
C	-2.499258	2.804803	1.008453
C	-3.571846	3.665462	1.180909
C	-4.842980	3.306154	0.703381
C	-5.023699	2.069483	0.104478
H	1.868651	3.735478	-0.421912
H	-0.487421	3.089015	-0.301597
H	-2.322920	-3.270308	1.054099
H	-0.765678	-4.713148	2.283291
H	1.570907	-3.929653	2.744321
H	2.313267	-1.736080	1.891876
H	6.822709	-2.059142	-1.118513
H	6.535988	0.424379	-0.799084
H	4.321191	3.580571	-1.799835

H	5.735951	2.518851	-1.912347
H	4.185949	2.020094	-2.639069
H	4.681127	3.582114	1.035518
H	4.690702	2.022494	1.888212
H	6.053187	2.475217	0.830314
H	-4.579236	-3.721010	-2.201971
H	-2.486977	-3.131383	-1.058644
H	-1.537004	3.075387	1.438105
H	-3.428034	4.611326	1.708011
H	-5.692831	3.979748	0.833981
H	-6.026553	1.778231	-0.207640
H	-6.429983	-2.038885	-2.375685
H	-6.127401	0.214501	-1.440871
H	2.580423	-2.483963	-0.408579
H	4.764331	-3.514364	-0.975726

PMP (**C2**)
E = -1350.855543 Hartree

Symbol	X	Y	Z
C	-1.641348	-2.649210	-0.130665
C	-0.310033	-2.276523	-0.178690
C	0.111208	-0.949262	0.107846
C	-0.888726	0.052006	0.290661
C	1.909001	0.768947	0.063520
C	0.874535	1.796403	0.198229
C	-0.446667	1.403820	0.577793
C	1.083541	3.193290	0.034329
C	0.180750	4.130538	0.512284
C	-0.974779	3.706937	1.192509
C	-1.291656	2.359367	1.187964
C	-2.258842	-0.326614	0.112059
C	-3.451961	0.498418	-0.175899
N	-4.575845	-0.275450	-0.159792
B	-4.238039	-1.848278	0.001174
C	-2.642270	-1.679368	0.025161
C	-3.571196	1.834981	-0.594628
C	-4.827284	2.360731	-0.877387
C	-5.969434	1.559855	-0.748826
C	-5.792264	0.227760	-0.406324
C	-4.818128	-2.637153	-1.298295
C	-4.837803	-2.395389	1.413727
C	1.525846	-0.580305	0.147494
C	5.210904	2.672806	-0.182767
C	3.904299	2.362953	0.163309
C	3.335503	1.088835	-0.095125
C	4.235557	0.061059	-0.514425
C	5.541255	0.412435	-0.927707
C	6.019743	1.706011	-0.800019
C	2.560881	-1.612797	0.215063

C	3.855004	-1.331302	-0.304080
C	2.348482	-2.881177	0.811810
C	3.302346	-3.884501	0.749527
C	4.515934	-3.651319	0.080852
C	4.791677	-2.386358	-0.412389
H	-1.914609	-3.694836	-0.298553
H	0.429238	-3.022656	-0.457049
H	1.967328	3.548937	-0.484888
H	0.388095	5.195094	0.382345
H	-1.640327	4.430042	1.668725
H	-2.221402	2.020239	1.643896
H	-6.968690	1.947553	-0.947480
H	-6.623204	-0.477547	-0.351102
H	-4.481176	-3.688702	-1.276110
H	-5.922726	-2.659309	-1.321027
H	-4.472819	-2.205172	-2.254110
H	-5.942777	-2.365908	1.424762
H	-4.543263	-3.447271	1.573662
H	-4.479216	-1.819337	2.284704
H	5.605491	3.668060	0.033970
H	3.322947	3.116820	0.683009
H	1.425519	-3.065748	1.358024
H	3.110601	-4.848121	1.226653
H	5.262648	-4.444192	-0.002132
H	5.774300	-2.196900	-0.842890
H	6.207297	-0.356926	-1.317303
H	7.034837	1.950709	-1.120383
H	-4.916479	3.398619	-1.205180
H	-2.684966	2.450852	-0.710339

PPM (**C3**)
E = -1350.866652 Hartree

Symbol	X	Y	Z
C	1.771122	2.400698	1.073982
C	0.415554	2.124607	0.984580
C	-0.067698	0.881514	0.491404
C	0.884706	-0.158480	0.284788
C	-1.919518	-0.721917	0.155443
C	-0.985319	-1.784415	0.523931
C	0.417153	-1.524183	0.484683
C	-1.416409	-3.036422	1.029655
C	-0.519856	-4.022884	1.408503
C	0.860603	-3.778638	1.328804
C	1.310822	-2.541438	0.900057
C	2.250268	0.231550	0.135104
C	3.351661	-0.490627	-0.514102
N	4.513920	0.211234	-0.394605
B	4.299576	1.630903	0.359478
C	2.709916	1.493573	0.553951

C	3.336746	-1.660225	-1.291716
C	4.518395	-2.111071	-1.869611
C	5.709157	-1.396459	-1.676704
C	5.658486	-0.222795	-0.938217
C	4.750555	2.826186	-0.648668
C	5.122963	1.619659	1.764425
C	-1.480016	0.606185	0.214658
C	-5.050910	-2.585365	-0.901355
C	-3.717981	-2.294225	-0.662976
C	-3.301835	-1.015568	-0.210798
C	-4.278060	0.020472	-0.151920
C	-5.639184	-0.313207	-0.350778
C	-6.028418	-1.596435	-0.696918
C	-2.432505	1.686662	-0.050690
C	-3.832153	1.405493	-0.050549
C	-2.033807	3.014944	-0.352573
C	-2.949832	4.047579	-0.468643
C	-4.319481	3.788906	-0.294241
C	-4.746296	2.483760	-0.115782
H	2.100534	3.349223	1.507187
H	-0.298657	2.863008	1.343484
H	-2.479320	-3.216756	1.172841
H	-0.891046	-4.970229	1.805842
H	1.576918	-4.535134	1.656945
H	2.375808	-2.326355	0.955742
H	6.652390	-1.729495	-2.109998
H	6.535693	0.406903	-0.779845
H	4.504964	3.804684	-0.199646
H	5.839230	2.827258	-0.838983
H	4.240493	2.779825	-1.626948
H	4.916227	2.542878	2.333633
H	4.853277	0.768438	2.413784
H	6.215219	1.582350	1.599198
H	-5.336129	-3.579005	-1.254195
H	-2.970918	-3.061904	-0.853441
H	-0.983551	3.232347	-0.527231
H	-2.603110	5.056545	-0.702907
H	-5.048549	4.600422	-0.348784
H	-5.816531	2.288092	-0.062272
H	-6.403568	0.458756	-0.270949
H	-7.085374	-1.822677	-0.854217
H	4.514261	-3.018632	-2.476885
H	2.403448	-2.198307	-1.443877

PPP (**C4**)
E = -1350.865163 Hartree

Symbol	X	Y	Z
C	-1.629242	-2.311902	1.244522
C	-0.308441	-2.060814	0.919536

C	0.095354	-0.825666	0.338680
C	-0.872431	0.207264	0.187027
C	1.969652	0.748736	0.038452
C	1.005797	1.835399	-0.078869
C	-0.394037	1.575681	0.062118
C	1.397535	3.161514	-0.406388
C	0.499188	4.214527	-0.400649
C	-0.835418	3.984797	-0.022073
C	-1.264431	2.687071	0.191853
C	-2.249308	-0.194977	0.248693
C	-3.443013	0.423088	-0.353705
N	-4.568112	-0.248399	0.025137
B	-4.230013	-1.560796	0.909018
C	-2.631631	-1.414247	0.837831
C	-3.557279	1.434435	-1.323455
C	-4.813365	1.786639	-1.804532
C	-5.956467	1.131953	-1.325755
C	-5.783775	0.097275	-0.418137
C	-4.820533	-1.368942	2.414848
C	-4.822824	-2.867951	0.142148
C	1.496650	-0.568293	0.030104
C	5.258375	2.323867	1.108048
C	3.910437	2.157884	0.830886
C	3.403563	0.962907	0.256776
C	4.319006	-0.111699	0.053383
C	5.690636	0.086587	0.330865
C	6.164780	1.288015	0.832118
C	2.409419	-1.642136	-0.362295
C	3.812546	-1.398798	-0.406126
C	1.931630	-2.877312	-0.872236
C	2.794123	-3.852725	-1.346295
C	4.180068	-3.624937	-1.342632
C	4.671769	-2.410117	-0.892295
H	-1.894460	-3.236998	1.763703
H	0.454807	-2.795826	1.172557
H	2.428551	3.355315	-0.692548
H	0.836718	5.219564	-0.663293
H	-1.534848	4.816777	0.085148
H	-2.296161	2.518735	0.490308
H	-6.955605	1.394384	-1.673837
H	-6.618582	-0.497590	-0.043661
H	-4.521346	-2.218135	3.053788
H	-5.925444	-1.337965	2.418277
H	-4.458115	-0.448094	2.904294
H	-4.492175	-3.784994	0.661034
H	-4.480838	-2.945357	-0.904981
H	-5.927747	-2.887757	0.135393
H	5.605439	3.253832	1.564149
H	3.223740	2.951057	1.115041

H	0.859768	-3.051365	-0.940041
H	2.389745	-4.786167	-1.744212
H	4.866254	-4.386298	-1.720208
H	5.744965	-2.227197	-0.941915
H	7.228762	1.410759	1.046281
H	6.392636	-0.733470	0.181910
H	-2.665382	1.926830	-1.702916
H	-4.903840	2.569991	-2.559983

TS1
E = -1350.860029 Hartree

Symbol	X	Y	Z
C	-4.336787	-2.891851	-1.466847
C	-3.171194	-2.448275	-0.866901
C	-3.057810	-1.130761	-0.349066
C	-4.145452	-0.240693	-0.523952
C	-5.333125	-0.720028	-1.127842
C	-5.438316	-2.024993	-1.578597
C	-2.660986	1.660381	0.166934
C	-3.970555	1.149911	-0.134888
C	-2.587403	3.067619	0.351435
C	-3.695781	3.899616	0.334055
C	-4.974034	3.369207	0.122608
C	-5.090877	2.012964	-0.117187
C	2.238433	0.381386	0.110389
C	0.890891	0.044290	0.422575
C	-0.092312	1.089813	0.481054
C	0.439486	2.403684	0.622041
C	1.794169	2.692933	0.533544
C	0.489028	-1.279011	0.864955
C	-0.883383	-1.617463	0.807341
C	-1.824199	-0.651376	0.253558
C	-1.524140	0.723164	0.323004
C	-0.408803	-3.683427	2.022654
C	0.954903	-3.344812	2.076751
B	4.290343	1.776795	-0.130653
C	1.388472	-2.152166	1.521841
C	-1.309927	-2.827425	1.409308
C	5.581210	-0.343047	-1.023529
C	5.599209	-1.659503	-1.461084
C	4.405188	-2.391924	-1.407222
C	3.304190	-0.487248	-0.414187
C	3.255179	-1.808975	-0.886181
C	2.718481	1.699549	0.189090
N	4.466135	0.214975	-0.534216
H	-4.390106	-3.906001	-1.869084
H	-2.311668	-3.114964	-0.826485
H	-6.175973	-0.047580	-1.280956

H	-6.362438	-2.365556	-2.050989
H	-1.645696	3.559226	0.522995
H	-3.558759	4.970819	0.498083
H	-5.858954	4.008988	0.135746
H	-6.081999	1.597660	-0.293119
H	-0.199218	3.241810	0.850977
H	2.122150	3.724680	0.688779
H	-0.767014	-4.599730	2.497170
H	1.664235	-3.995805	2.592612
H	2.429994	-1.856248	1.640951
H	-2.372033	-3.067368	1.435369
H	6.460498	0.301599	-1.071703
H	6.518681	-2.092179	-1.855708
H	4.372959	-3.418762	-1.777390
H	2.321094	-2.364408	-0.848814
C	5.200216	2.095057	1.181851
H	6.280268	2.009088	0.962982
H	5.024147	3.129850	1.524388
H	4.976892	1.427169	2.032220
C	4.670220	2.691028	-1.422058
H	4.447387	3.750994	-1.206978
H	5.745011	2.636306	-1.673742
H	4.100743	2.415870	-2.327192

TS2

E = -1350.854290 Hartree

Symbol	X	Y	Z
C	1.318342	3.327837	0.126378
C	0.373202	4.329138	0.284621
C	-0.970836	4.001331	0.494152
C	-1.327556	2.668067	0.458570
C	-3.403982	0.439267	-0.426419
N	-4.532342	-0.268908	-0.136462
B	-4.220394	-1.579063	0.762697
C	-3.481900	1.487554	-1.359366
C	-4.713335	1.824427	-1.909759
C	-5.865311	1.118727	-1.534728
C	-5.725143	0.059669	-0.649795
C	5.578677	2.289947	0.207915
C	4.204076	2.208287	0.042279
C	5.703108	-0.091128	0.251413
C	6.344954	1.128734	0.353333
C	1.493782	-0.471561	0.099341
C	2.003708	0.840387	0.040214
C	3.484527	0.981883	0.010199
C	4.309645	-0.195574	0.037861
C	3.730657	-1.499475	-0.244485
C	2.321241	-1.617024	-0.271717

C	0.106337	-0.740092	0.455514
C	-0.868049	0.260179	0.243246
C	-0.394181	1.632116	0.209249
C	1.011248	1.939227	0.122259
C	-0.294950	-1.963113	1.063931
C	-1.627081	-2.242884	1.311488
C	-2.625578	-1.387575	0.810340
C	-2.237282	-0.164625	0.234253
C	4.521793	-2.623031	-0.586068
C	3.945582	-3.814270	-0.993821
C	2.548172	-3.905238	-1.112425
C	1.757074	-2.824220	-0.763120
H	2.326896	3.679152	0.007163
H	0.698800	5.371749	0.278196
H	-1.718176	4.771530	0.695786
H	-2.357660	2.398144	0.676002
H	-6.846189	1.366603	-1.940631
H	-6.567295	-0.564947	-0.346980
H	6.050531	3.274760	0.234776
H	3.709830	3.157611	-0.062257
H	0.471843	-2.670625	1.378481
H	-1.900181	-3.160155	1.840290
H	5.608155	-2.547471	-0.575824
H	4.579185	-4.662067	-1.263987
H	2.084633	-4.815708	-1.498663
H	0.680580	-2.895114	-0.905072
C	-4.713409	-2.898014	-0.053055
H	-4.400093	-3.808609	0.487409
H	-5.813334	-2.947011	-0.148085
H	-4.287080	-2.959989	-1.069797
C	-4.929333	-1.413488	2.219612
H	-6.031780	-1.416854	2.139883
H	-4.652567	-2.256599	2.876460
H	-4.633850	-0.484571	2.737960
H	7.422394	1.179248	0.523649
H	6.293530	-1.001997	0.337950
H	-4.778303	2.636278	-2.637225
H	-2.580692	2.021527	-1.652194

TS3

E = -1350.848385 Hartree

Symbol	X	Y	Z
C	4.625025	2.282920	2.172598
C	3.415141	2.120639	1.515892
C	3.179212	1.018901	0.655084
C	4.201810	0.033062	0.526746
C	5.430255	0.228719	1.197257
C	5.650909	1.339291	1.996884

C	2.601615	-1.446974	-0.664034
C	3.942034	-1.156066	-0.276909
C	2.366782	-2.576818	-1.488131
C	3.398900	-3.414682	-1.878870
C	4.714289	-3.147003	-1.462255
C	4.975446	-2.029453	-0.684646
C	-2.269945	-0.318822	-0.185541
C	-0.911192	0.122050	-0.455778
C	0.120823	-0.865534	-0.325239
C	-0.181770	-2.185547	0.093346
C	-1.456081	-2.531021	0.481735
C	-0.421983	1.494295	-0.685486
C	0.920236	1.836482	-0.299100
C	1.880543	0.792158	0.030012
C	1.537778	-0.503904	-0.342783
C	0.551983	4.158711	-1.007165
C	-0.736928	3.820588	-1.432463
B	-3.955945	-1.781273	0.983867
C	-1.183759	2.515832	-1.282804
C	1.363921	3.170898	-0.473333
C	-5.777745	0.031744	0.711398
C	-6.303141	1.148363	0.079666
C	-5.478316	1.802096	-0.836327
C	-3.616299	0.340262	-0.250968
C	-4.157467	1.393948	-1.011552
C	-2.492157	-1.598755	0.380002
N	-4.512547	-0.354703	0.520507
H	4.768647	3.134300	2.841783
H	2.613379	2.831583	1.707391
H	6.215056	-0.523047	1.112552
H	6.606551	1.460794	2.511706
H	1.359692	-2.768410	-1.856875
H	3.189545	-4.269212	-2.526268
H	5.533358	-3.800732	-1.770315
H	6.006484	-1.811077	-0.406318
H	0.630102	-2.898065	0.227235
H	-1.649634	-3.517099	0.912744
H	0.938671	5.171549	-1.139987
H	-1.380467	4.561125	-1.912532
H	-2.139329	2.267122	-1.702718
H	2.398843	3.416742	-0.248474
H	-6.375376	-0.605524	1.365326
H	-7.335373	1.451450	0.254306
H	-5.863777	2.627209	-1.438852
H	-3.600217	1.874512	-1.800757
C	-3.951011	-1.893282	2.608543
H	-4.974862	-1.886744	3.025376

H	-3.485608	-2.843350	2.923977
H	-3.387481	-1.076865	3.093250
C	-4.854045	-2.933979	0.260534
H	-4.406062	-3.925830	0.447140
H	-5.886092	-2.970529	0.653904
H	-4.917682	-2.805340	-0.834346

En-TS4
E = -1350.846435 Hartree

Symbol	X	Y	Z
C	2.237015	-0.348223	0.084187
C	3.423104	0.479440	-0.197842
N	4.533864	-0.305790	-0.304148
B	4.182413	-1.885803	-0.223008
C	2.594966	-1.699556	-0.091395
C	3.543403	1.849297	-0.498147
C	4.790624	2.380250	-0.803554
C	5.923661	1.554935	-0.815014
C	5.741849	0.201058	-0.576551
C	-1.534792	-0.524036	0.216121
C	-1.902257	0.832325	0.100618
C	-3.306989	1.129441	-0.253476
C	-4.184696	0.049960	-0.603362
C	-3.858298	-1.301129	-0.171692
C	-2.566257	-1.546695	0.353106
C	-0.135577	-0.931520	0.153124
C	0.883038	0.039257	0.332474
C	0.463385	1.367331	0.729584
C	-0.850724	1.827298	0.384012
C	0.245425	-2.260805	-0.181203
C	1.571576	-2.650670	-0.222287
C	-4.824248	-2.333523	-0.121160
C	-4.554343	-3.543311	0.496246
C	-3.313042	-3.743074	1.123603
C	-2.340749	-2.759785	1.050858
C	-5.129847	2.667588	-0.884702
C	-3.884542	2.424933	-0.325367
C	-5.423295	0.319672	-1.226064
C	-5.889532	1.610251	-1.400342
C	-1.018518	3.239074	0.449104
C	-0.099379	4.077587	1.061705
C	1.057203	3.548914	1.653539
C	1.337807	2.210009	1.451370
H	6.917023	1.945374	-1.037813
H	6.559970	-0.519872	-0.625246
H	-0.524104	-2.987459	-0.432248
H	1.822015	-3.692595	-0.439641
H	-5.821696	-2.163023	-0.524788

H	-5.323378	-4.318137	0.536681
H	-3.117842	-4.662219	1.680591
H	-1.394455	-2.907343	1.569158
H	-5.509895	3.691297	-0.917985
H	-3.378461	3.277561	0.099583
H	-1.877435	3.715518	0.001807
H	-0.302369	5.150318	1.102640
H	1.739035	4.182561	2.224826
H	2.258271	1.780383	1.846791
C	4.672388	-2.591466	-1.603446
H	5.773182	-2.623243	-1.695534
H	4.327616	-3.639880	-1.625001
H	4.273149	-2.099874	-2.507542
C	4.861900	-2.521474	1.112778
H	4.561390	-3.577140	1.225311
H	5.965724	-2.509230	1.055748
H	4.567244	-1.997777	2.038482
H	-6.849587	1.792801	-1.887968
H	-6.038944	-0.514169	-1.562282
H	2.661374	2.482635	-0.502207
H	4.881502	3.442919	-1.040239

En-TS5

E = -1350.839256 Hartree

Symbol	X	Y	Z
C	-5.335823	2.558461	0.308619
C	-3.983672	2.330229	0.109902
C	-3.418665	1.030137	0.183992
C	-4.327660	-0.068450	0.284211
C	-5.694207	0.191888	0.542631
C	-6.194400	1.482184	0.584499
C	-2.490263	-1.603068	-0.374852
C	-3.860747	-1.411144	-0.041840
C	-2.116727	-2.821086	-0.999749
C	-3.018727	-3.857560	-1.169123
C	-4.341674	-3.710887	-0.716101
C	-4.753234	-2.501324	-0.181894
C	2.283300	-0.221590	-0.144281
C	0.885229	0.183603	-0.262554
C	-0.101285	-0.827932	-0.003689
C	0.271470	-2.099468	0.490238
C	1.591292	-2.427400	0.689539
C	0.342785	1.551155	-0.338929
C	-0.963021	1.807950	0.202822
C	-1.978406	0.762318	0.124128
C	-1.532119	-0.537294	-0.105105
C	-0.387330	4.181890	0.424202
C	0.690637	3.982950	-0.451161

B	4.138767	-1.745257	0.639728
C	1.039734	2.687880	-0.801413
C	-1.215866	3.107445	0.709346
C	5.908757	0.047880	0.143474
C	6.330870	1.251201	-0.397715
C	5.352684	2.054503	-0.982300
C	3.605678	0.469517	-0.344350
C	4.015030	1.665243	-0.964227
C	2.591470	-1.509127	0.364192
N	4.621399	-0.311966	0.148413
H	-5.728008	3.575878	0.242946
H	-3.352036	3.177029	-0.139113
H	-6.383997	-0.639848	0.680531
H	-7.255148	1.653181	0.780914
H	-1.100237	-2.938753	-1.372217
H	-2.702218	-4.780880	-1.659355
H	-5.055620	-4.530690	-0.822760
H	-5.799834	-2.385098	0.096586
H	-0.506907	-2.800109	0.785783
H	1.859797	-3.390434	1.131810
H	-0.610290	5.171392	0.828924
H	1.271412	4.826216	-0.831176
H	1.840292	2.541221	-1.506188
H	-2.090270	3.267009	1.335439
H	6.602951	-0.673072	0.578027
H	7.385090	1.527426	-0.386706
H	5.623660	2.993033	-1.470280
H	3.322128	2.312541	-1.470068
C	4.816409	-2.883369	-0.312747
H	4.394265	-3.874458	-0.071071
H	5.909045	-2.955882	-0.163217
H	4.637238	-2.705242	-1.387719
C	4.485774	-1.941132	2.220073
H	5.574790	-1.991610	2.402409
H	4.058312	-2.890063	2.588242
H	4.078187	-1.132346	2.851702

TS6
E = -1350.847794 Hartree

Symbol	X	Y	Z
N	4.547938	0.190031	-0.247711
B	4.357216	1.636623	0.454280
C	3.368054	-0.479635	-0.385359
C	3.338498	-1.630770	-1.191557
C	4.517565	-2.107090	-1.753141
C	5.727280	-1.439297	-1.517248
C	5.692505	-0.273002	-0.767877
C	-2.426172	3.113514	0.264070

C	-3.301966	4.038939	-0.286398
C	-4.372672	3.611117	-1.081038
C	-4.613708	2.251703	-1.179871
C	1.817443	2.530552	0.927292
C	0.462586	2.276577	0.799297
C	2.273581	0.278696	0.251034
C	2.759575	1.550617	0.589884
C	-1.905470	-0.665327	0.259321
C	-1.509728	0.685216	0.338401
C	-0.071042	0.986489	0.511279
C	0.891045	-0.070146	0.400951
C	0.446741	-1.444443	0.468062
C	-0.924602	-1.742640	0.249222
C	-3.331192	-1.000889	0.205179
C	-4.226903	-0.073220	-0.380331
C	-3.772572	1.299141	-0.563452
C	-2.546484	1.713670	0.055728
C	-3.871920	-2.187769	0.758627
C	-5.207694	-2.522179	0.595117
C	-6.056196	-1.669804	-0.130095
C	-5.574454	-0.452304	-0.584447
C	1.332128	-2.502827	0.793764
C	0.949184	-3.825932	0.679852
C	-0.335404	-4.124115	0.187379
C	-1.249992	-3.104486	-0.009384
H	6.669840	-1.796230	-1.932602
H	6.581594	0.333726	-0.588012
H	-1.664012	3.519388	0.911071
H	-3.144490	5.102178	-0.091972
H	-5.036659	4.329769	-1.566046
H	-5.494513	1.909860	-1.722613
H	2.142370	3.533229	1.219143
H	-0.186070	3.118408	0.966959
H	-3.237319	-2.838283	1.356682
H	-5.597323	-3.438920	1.043279
H	-7.104888	-1.933634	-0.284270
H	-6.270544	0.243687	-1.051251
H	2.335172	-2.263180	1.142837
H	1.647031	-4.625529	0.937592
H	-0.624589	-5.157783	-0.015025
H	-2.238315	-3.360153	-0.380773
C	5.120335	1.646687	1.893280
H	6.216165	1.564596	1.773817
H	4.925654	2.595588	2.423014
H	4.794062	0.828185	2.558512
C	4.888007	2.783996	-0.569864
H	4.648605	3.783128	-0.164783

H	5.983936	2.752117	-0.708379
H	4.422707	2.718323	-1.569121
H	4.495479	-2.999893	-2.381617
H	2.396329	-2.138142	-1.379919

Inversion of EH2
PMM (**C1**)
E = -1504.420960 Hartree

Symbol	X	Y	Z
C	0.773410	3.207104	-0.447130
C	-0.493945	2.649515	-0.365097
C	-0.697141	1.320669	0.093972
C	0.452903	0.512403	0.350787
C	-2.121314	-0.680165	0.251821
C	-1.029977	-1.389675	0.909799
C	0.236334	-0.751024	1.036262
C	-1.236578	-2.608429	1.604642
C	-0.243760	-3.167511	2.395038
C	0.991003	-2.513192	2.544006
C	1.215893	-1.318333	1.880759
C	1.733713	1.040210	0.013762
C	3.037548	0.369657	-0.137370
N	4.028080	1.283595	-0.122915
B	3.471738	2.797465	-0.255260
C	1.913205	2.415771	-0.244808
C	3.360986	-0.999242	-0.427814
C	4.743959	-1.378475	-0.412214
C	5.731203	-0.371167	-0.221527
C	5.339692	0.938391	-0.141653
C	2.395616	-1.969168	-0.807840
C	2.770988	-3.269215	-1.075553
C	4.126204	-3.662945	-0.966519
C	5.095524	-2.733330	-0.652222
C	4.023037	3.406118	-1.661898
C	3.907074	3.674289	1.045989
C	-2.026806	0.715778	0.171305
C	-4.376833	-3.304274	-1.272828
C	-3.283356	-2.704801	-0.668241
C	-3.305976	-1.341275	-0.278772
C	-4.474117	-0.576181	-0.564049
C	-5.576175	-1.212056	-1.178576
C	-5.539928	-2.555350	-1.519226
C	-3.272614	1.480816	0.155191
C	-4.494697	0.835652	-0.200255
C	-3.339635	2.815825	0.630040
C	-4.536068	3.514028	0.677026
C	-5.724986	2.897355	0.254441
C	-5.698488	1.575030	-0.159480
H	0.879998	4.259162	-0.726750

H	-1.350112	3.248078	-0.667062
H	-2.215173	-3.084342	1.573904
H	-0.442255	-4.095655	2.935789
H	1.760481	-2.928948	3.198010
H	2.156384	-0.789574	2.032549
H	6.789651	-0.633583	-0.205083
H	6.048679	1.765484	-0.092281
H	4.405784	-4.700821	-1.160797
H	6.149269	-3.017546	-0.611751
H	3.560438	4.392348	-1.843780
H	5.116698	3.562870	-1.653102
H	3.783425	2.767841	-2.530308
H	3.461762	4.683117	0.993511
H	3.579251	3.221592	1.998079
H	5.002664	3.810395	1.099792
H	-4.322322	-4.352279	-1.576056
H	-2.371128	-3.283943	-0.535902
H	-2.442331	3.291150	1.020058
H	-4.552098	4.535194	1.064502
H	-6.671412	3.441679	0.286004
H	-6.636909	1.089004	-0.425950
H	-6.403327	-3.020084	-2.000478
H	-6.469313	-0.633662	-1.414746
H	2.016854	-3.998856	-1.377175
H	1.354398	-1.677931	-0.914875

PMP (**C2**)
E -1504.404660 Hartree

Symbol	X	Y	Z
C	-0.810424	3.176272	0.266932
C	0.444443	2.594549	0.347920
C	0.679268	1.252912	-0.064311
C	-0.453342	0.443619	-0.370649
C	2.184191	-0.720904	-0.246784
C	1.010193	-1.545827	-0.542435
C	-0.225651	-0.901917	-0.854730
C	0.992023	-2.967232	-0.580795
C	-0.045888	-3.670940	-1.173147
C	-1.118398	-2.980476	-1.763539
C	-1.218162	-1.615343	-1.565441
C	-1.753945	1.001720	-0.164105
C	-3.071622	0.358909	-0.006258
N	-4.051051	1.264887	-0.197835
B	-3.487278	2.783299	-0.176856
C	-1.937457	2.391798	-0.020941
C	-3.425422	-0.947211	0.477214
C	-4.808646	-1.322350	0.441690
C	-5.773190	-0.357037	0.034213
C	-5.365098	0.926312	-0.211155

C	-2.494307	-1.838389	1.073332
C	-2.899719	-3.071626	1.539252
C	-4.249115	-3.478584	1.413896
C	-5.187585	-2.617633	0.883114
C	-4.143186	3.531330	1.112365
C	-3.799930	3.530704	-1.588853
C	2.019729	0.671245	-0.133452
C	5.137059	-3.141335	-0.246284
C	3.910886	-2.576741	-0.563755
C	3.535902	-1.283804	-0.115559
C	4.564215	-0.490117	0.479421
C	5.779931	-1.105342	0.856862
C	6.057250	-2.423466	0.533478
C	3.207262	1.520792	-0.024043
C	4.413812	0.960929	0.481302
C	3.229649	2.878179	-0.432114
C	4.324033	3.693885	-0.191209
C	5.447388	3.174224	0.473454
C	5.495705	1.823303	0.777325
H	-0.926836	4.239071	0.497820
H	1.269600	3.190389	0.727643
H	1.801818	-3.530895	-0.129697
H	-0.011915	-4.762478	-1.195901
H	-1.894915	-3.518140	-2.311339
H	-2.089479	-1.075953	-1.935854
H	-6.831069	-0.618920	-0.007484
H	-6.061982	1.739233	-0.418248
H	-4.551285	-4.467736	1.765295
H	-6.239606	-2.905414	0.825689
H	-3.677104	4.524791	1.237790
H	-5.228634	3.700393	0.994794
H	-3.991003	2.977751	2.055429
H	-4.886102	3.659150	-1.748748
H	-3.354231	4.540684	-1.595730
H	-3.395460	2.988700	-2.461451
H	5.382538	-4.140953	-0.611925
H	3.242935	-3.137247	-1.209033
H	2.380377	3.285651	-0.976554
H	4.311796	4.733478	-0.525984
H	6.302490	3.815437	0.698724
H	6.412055	1.416629	1.203674
H	6.539883	-0.521871	1.375800
H	7.007121	-2.873801	0.829912
H	-2.172086	-3.739269	2.004648
H	-1.456274	-1.535844	1.186599

PPM (**C3**)
E = -1504.417576 Hartree

Symbol	X	Y	Z

C	0.979238	3.090711	0.829529
C	-0.319792	2.625627	0.689307
C	-0.599150	1.258897	0.419538
C	0.492326	0.342552	0.449084
C	-2.202625	-0.621604	0.398151
C	-1.154926	-1.437352	1.010697
C	0.200126	-1.000453	0.933712
C	-1.435910	-2.601991	1.768233
C	-0.430451	-3.356072	2.352113
C	0.909011	-2.953433	2.223477
C	1.207907	-1.783637	1.546823
C	1.804528	0.885258	0.281284
C	3.055700	0.236930	-0.134402
N	4.102642	1.050595	0.108897
B	3.643658	2.530228	0.568158
C	2.064499	2.244990	0.550809
C	3.275045	-0.994953	-0.839148
C	4.629934	-1.410984	-1.057368
C	5.687557	-0.549662	-0.651281
C	5.385522	0.677065	-0.123131
C	2.225425	-1.784358	-1.378989
C	2.500271	-2.963891	-2.039733
C	3.835269	-3.410684	-2.187711
C	4.880240	-2.644214	-1.715406
C	4.160282	3.589680	-0.556258
C	4.205488	2.847530	2.063693
C	-1.945061	0.735428	0.171671
C	-5.000658	-3.088422	-0.294971
C	-3.732973	-2.569807	-0.089648
C	-3.512098	-1.177616	0.071333
C	-4.615554	-0.299693	-0.135002
C	-5.907876	-0.854930	-0.293940
C	-6.107539	-2.224145	-0.350834
C	-3.012147	1.591434	-0.348147
C	-4.360363	1.122813	-0.331819
C	-2.770166	2.870712	-0.913220
C	-3.803322	3.716632	-1.281369
C	-5.136094	3.308570	-1.105516
C	-5.400604	2.023475	-0.662758
H	1.151929	4.128945	1.126075
H	-1.148707	3.310291	0.858732
H	-2.470495	-2.891616	1.937836
H	-0.686827	-4.240658	2.939384
H	1.707777	-3.527074	2.698687
H	2.236704	-1.429837	1.541280
H	6.726483	-0.843550	-0.805292
H	6.148086	1.411782	0.137294
H	4.036491	-4.354235	-2.699835
H	5.915097	-2.962313	-1.859087

H	3.761184	4.593949	-0.329308
H	5.261410	3.679367	-0.574324
H	3.829323	3.329461	-1.576933
H	3.821567	3.820888	2.415884
H	3.902533	2.088496	2.805890
H	5.308642	2.912823	2.081904
H	-5.134160	-4.165302	-0.420188
H	-2.883674	-3.249565	-0.078101
H	-1.747494	3.192951	-1.090951
H	-3.576862	4.693718	-1.713937
H	-5.959417	3.979295	-1.361108
H	-6.437741	1.695470	-0.605014
H	-6.768574	-0.198178	-0.413930
H	-7.115232	-2.623998	-0.484079
H	1.681889	-3.553970	-2.457093
H	1.196926	-1.444844	-1.289667

PPP (**C4**)
E = -1504.415707 Hartree

Symbol	X	Y	Z
C	-0.817567	2.973059	-1.020370
C	0.426708	2.503989	-0.634729
C	0.633277	1.137049	-0.296658
C	-0.458745	0.232297	-0.410140
C	2.279663	-0.698089	-0.290972
C	1.186522	-1.646950	-0.469333
C	-0.159767	-1.179874	-0.590240
C	1.391650	-3.052683	-0.445940
C	0.370310	-3.947581	-0.716276
C	-0.905213	-3.464749	-1.056701
C	-1.158459	-2.107565	-0.977464
C	-1.768817	0.817187	-0.463600
C	-3.086932	0.267540	-0.111553
N	-4.065984	1.049635	-0.609460
B	-3.498828	2.454305	-1.172450
C	-1.948294	2.150276	-0.885135
C	-3.439243	-0.804254	0.777727
C	-4.823956	-1.158462	0.887079
C	-5.790065	-0.377740	0.191863
C	-5.380286	0.736382	-0.490717
C	-2.497072	-1.473718	1.602502
C	-2.896294	-2.494391	2.440410
C	-4.252141	-2.898585	2.484835
C	-5.199252	-2.237550	1.730513
C	-3.820842	2.599793	-2.762041
C	-4.142404	3.647337	-0.269065
C	1.968459	0.634036	0.003674
C	5.428090	-2.413166	-1.550704
C	4.092628	-2.135491	-1.304450

C	3.690132	-1.044864	-0.490164
C	4.709495	-0.175445	-0.000434
C	6.064672	-0.488031	-0.252770
C	6.428394	-1.597655	-0.998291
C	2.971526	1.465770	0.666458
C	4.328033	1.033742	0.718303
C	2.608049	2.615252	1.415065
C	3.543299	3.340629	2.135474
C	4.887771	2.933934	2.147728
C	5.263003	1.790826	1.459979
H	-0.921764	3.997657	-1.387756
H	1.287800	3.170144	-0.677157
H	2.372147	-3.443102	-0.184611
H	0.566158	-5.021665	-0.684029
H	-1.699312	-4.152553	-1.354868
H	-2.152321	-1.744232	-1.226826
H	-6.849433	-0.629180	0.254331
H	-6.077377	1.427920	-0.965198
H	-4.550471	-3.720511	3.139438
H	-6.253772	-2.515418	1.790214
H	-3.362109	3.520341	-3.163188
H	-4.907055	2.674055	-2.952526
H	-3.433356	1.754791	-3.357447
H	-3.676303	4.610150	-0.543468
H	-3.980895	3.503007	0.813592
H	-5.229192	3.759999	-0.433090
H	5.694990	-3.253449	-2.195676
H	3.339492	-2.744227	-1.797992
H	1.562727	2.913022	1.467712
H	3.225878	4.212677	2.711600
H	5.629728	3.496269	2.719008
H	6.299694	1.459742	1.517742
H	7.483219	-1.810109	-1.185920
H	6.846308	0.176062	0.115384
H	-2.161186	-2.991735	3.076267
H	-1.455542	-1.162150	1.589748

En-TS1

E = -1504.410282 Hartree

Symbol	X	Y	Z
C	3.384376	3.003952	-0.134324
C	4.596331	3.646505	-0.331721
C	5.772031	2.903334	-0.491895
C	5.683393	1.523980	-0.489030
C	-1.008763	3.312784	0.230304
C	0.299673	2.850123	0.288430
C	-1.783207	1.048276	0.123063
C	-3.001334	0.291557	-0.207562
N	-4.071951	1.109668	-0.178411

B	-3.660706	2.668285	-0.030934
C	-2.079433	2.424202	0.085911
C	-3.167619	-1.062685	-0.655354
C	-4.504812	-1.553688	-0.820750
C	-5.594618	-0.657286	-0.635169
C	-5.337719	0.661140	-0.369839
C	-2.084295	-1.912637	-1.003283
C	-2.310852	-3.208487	-1.418259
C	-3.629335	-3.717297	-1.499378
C	-4.705807	-2.902342	-1.217765
C	2.096407	-0.517134	0.452200
C	1.996451	0.874649	0.259943
C	0.636791	1.468575	0.351647
C	-0.484143	0.577649	0.470516
C	-0.265381	-0.680667	1.159615
C	1.043567	-1.214185	1.180964
C	3.223284	-1.276562	-0.065514
C	4.412466	-0.602566	-0.438023
C	4.454001	0.846327	-0.316282
C	3.247525	1.590590	-0.078412
C	3.129082	-2.671388	-0.317148
C	4.189978	-3.384264	-0.847655
C	5.391674	-2.722441	-1.156190
C	5.489421	-1.354283	-0.967307
C	-1.278680	-1.296754	1.930600
C	-1.011964	-2.420718	2.693972
C	0.292705	-2.944171	2.732743
C	1.301873	-2.343757	1.996012
H	2.534426	3.652090	-0.009358
H	4.622600	4.738391	-0.348071
H	6.737372	3.397175	-0.621642
H	6.595092	0.942508	-0.616780
H	-1.186835	4.391320	0.263298
H	1.058566	3.614042	0.348050
H	-6.620829	-1.006210	-0.756337
H	-6.123811	1.413387	-0.296699
H	-3.792489	-4.750925	-1.812540
H	-5.728016	-3.273393	-1.318695
H	2.190123	-3.187629	-0.126046
H	4.083173	-4.453501	-1.043880
H	-2.273020	-0.853025	1.961283
H	-1.804648	-2.872761	3.294166
H	0.521655	-3.802430	3.368414
H	2.320097	-2.721339	2.081316
C	-4.301009	3.286696	1.332608
H	-3.952016	4.323655	1.480430
H	-5.404711	3.323887	1.287638

H	-4.021858	2.716114	2.235568
C	-4.140189	3.441698	-1.381838
H	-5.240545	3.494957	-1.467186
H	-3.769920	4.481892	-1.363757
H	-3.755469	2.973825	-2.304915
H	6.234275	-3.276237	-1.576276
H	6.407085	-0.852108	-1.270360
H	-1.067858	-1.530896	-0.966821
H	-1.466473	-3.844251	-1.692210

TS2

E = -1504.405188 Hartree

Symbol	X	Y	Z
C	1.323418	-3.073607	-1.007963
C	0.269707	-3.888084	-1.392570
C	-1.018391	-3.358696	-1.525577
C	-1.212053	-2.030973	-1.202638
C	-3.068743	0.248431	-0.018796
N	-4.070329	1.054001	-0.425772
B	-3.529949	2.448213	-1.036317
C	-3.370667	-0.857006	0.847585
C	-4.749272	-1.194463	1.049764
C	-5.752001	-0.371186	0.464300
C	-5.376789	0.757055	-0.214642
C	-2.380070	-1.584855	1.558171
C	-2.732169	-2.641007	2.373099
C	-4.088541	-3.021924	2.509951
C	-5.078622	-2.306509	1.869135
C	5.677416	-2.613466	-0.656654
C	4.313047	-2.385546	-0.559128
C	6.085918	-0.329310	-0.106323
C	6.585837	-1.567823	-0.461355
C	1.951329	0.570000	-0.113786
C	2.296582	-0.777898	-0.330982
C	3.743361	-1.116145	-0.264211
C	4.700615	-0.086337	0.038554
C	4.257809	1.191297	0.573835
C	2.875811	1.492285	0.539253
C	0.631984	1.085694	-0.458578
C	-0.467163	0.199520	-0.480950
C	-0.165787	-1.197902	-0.737148
C	1.186169	-1.693587	-0.693689
C	0.426870	2.439148	-0.845522
C	-0.837970	2.916355	-1.145109
C	-1.966290	2.119976	-0.882553
C	-1.773770	0.790967	-0.453715
C	5.143857	2.099526	1.202547
C	4.681297	3.248516	1.821293

C	3.300450	3.506710	1.864699
C	2.419655	2.640247	1.240788
H	2.277449	-3.566546	-0.966580
H	0.466819	-4.938451	-1.618399
H	-1.848573	-3.969563	-1.885852
H	-2.199167	-1.598594	-1.343452
H	-6.807775	-0.607724	0.601693
H	-6.096253	1.471362	-0.616290
H	-4.350801	-3.870664	3.145492
H	-6.130618	-2.569081	1.999839
H	6.029762	-3.618258	-0.900591
H	3.702268	-3.253570	-0.730704
H	1.293627	3.087741	-0.971777
H	-0.957854	3.928772	-1.540230
H	6.209596	1.879978	1.244629
H	5.385486	3.926165	2.309232
H	2.917465	4.374470	2.406274
H	1.351344	2.829084	1.324922
C	-4.071185	3.655683	-0.086110
H	-3.617641	4.610068	-0.406832
H	-5.166612	3.783843	-0.152085
H	-3.814714	3.513423	0.978325
C	-3.987187	2.593578	-2.592783
H	-5.084329	2.686232	-2.689021
H	-3.548812	3.504683	-3.036045
H	-3.667237	1.740120	-3.215620
H	7.661005	-1.724383	-0.570654
H	6.785972	0.486618	0.066045
H	-1.958850	-3.183821	2.920300
H	-1.336278	-1.292960	1.476219

En-TS3
E = -1504.378724 Hartree

Symbol	X	Y	Z
C	-1.642638	1.109996	-0.477452
C	-0.420562	0.414348	-0.804009
C	0.805625	1.152696	-0.738618
C	0.767985	2.561800	-0.609401
C	-0.421786	3.225967	-0.380433
C	-0.233997	-1.020386	-1.047676
C	0.896694	-1.666340	-0.457100
C	2.073551	0.451431	-0.504175
C	0.165896	-3.790299	-1.429505
C	-0.837074	-3.122297	-2.142419
B	-2.856911	2.988507	0.669837
C	-3.984784	3.832502	-0.156506
C	-1.007922	-1.755919	-1.957769
C	1.039808	-3.063640	-0.629973

C	-2.433672	3.689696	2.073599
C	-4.420322	1.236272	1.821817
C	-4.953966	-0.019685	1.924331
C	-4.671106	-0.954053	0.892743
C	-2.956580	0.607043	0.047473
C	-3.748315	-0.582807	-0.147536
C	-5.384436	-2.178000	0.788643
C	-3.819517	-1.310280	-1.361095
C	-5.319075	-2.931947	-0.364576
C	-4.582823	-2.454472	-1.473044
C	-1.599340	2.499279	-0.188193
N	-3.460809	1.519144	0.907369
C	3.903863	-2.805296	2.625778
C	2.902231	-2.407871	1.754592
C	3.090850	-1.329178	0.854118
C	4.329525	-0.623562	0.886576
C	5.335458	-1.053114	1.781367
C	5.136975	-2.130501	2.630966
C	3.385283	1.059743	-0.683609
C	4.517072	0.511680	-0.009410
C	3.601032	2.098861	-1.624487
C	4.863679	2.623162	-1.849473
C	5.967729	2.120222	-1.140045
C	5.791734	1.074126	-0.248035
C	2.011637	-0.846502	0.008874
H	1.699355	3.121294	-0.554533
H	-0.408081	4.302463	-0.189480
H	0.318248	-4.863946	-1.561456
H	-1.460620	-3.657622	-2.861674
H	-4.888316	4.015956	0.452471
H	-3.579015	4.819709	-0.439082
H	-4.303197	3.328440	-1.085610
H	-1.693919	-1.215387	-2.596319
H	1.906161	-3.569808	-0.208163
H	-3.306575	3.918600	2.711730
H	-1.930848	4.652853	1.877702
H	-1.735876	3.074928	2.668981
H	-4.702728	2.059013	2.478575
H	-5.662294	-0.263700	2.716684
H	-6.034872	-2.475431	1.614135
H	-3.341296	-0.899397	-2.240340
H	-5.889608	-3.859618	-0.447619
H	-4.633437	-2.979711	-2.428896
H	3.724643	-3.629107	3.320373
H	1.933775	-2.904389	1.797071
H	6.282313	-0.514959	1.826882
H	5.928864	-2.436035	3.318442

H	2.764554	2.461880	-2.219800
H	4.999496	3.412150	-2.592584
H	6.965955	2.528939	-1.311923
H	6.665286	0.660304	0.255670

TS3a

E = -1504.365118 Hartree

Symbol	X	Y	Z
C	-1.734297	0.650008	-0.854737
C	-0.413003	0.030719	-0.760646
C	0.705669	0.920452	-0.779850
C	0.567545	2.189902	-1.397985
C	-0.677478	2.691740	-1.705908
C	-0.075012	-1.375325	-0.604038
C	1.230215	-1.796654	-0.190419
C	2.001312	0.516822	-0.245248
C	0.395584	-4.101238	-0.033817
C	-0.771947	-3.723527	-0.717335
B	-3.175005	2.710618	-0.800398
C	-4.291906	2.896735	-1.977786
C	-0.982715	-2.381920	-0.978912
C	1.383346	-3.155789	0.192956
C	-2.933727	4.060652	0.071293
C	-4.600631	1.620604	1.089740
C	-4.986959	0.545862	1.842248
C	-4.616759	-0.747834	1.388866
C	-3.051103	0.325154	-0.191192
C	-3.759083	-0.868902	0.236612
C	-5.214443	-1.909799	1.948253
C	-3.848928	-2.092181	-0.480502
C	-5.135940	-3.122432	1.298076
C	-4.513891	-3.188391	0.031017
C	-1.813710	2.007689	-1.249435
N	-3.666003	1.491809	0.117834
C	5.549089	-2.805026	-0.532493
C	4.200296	-2.486140	-0.508777
C	3.736051	-1.216865	-0.075179
C	4.715377	-0.234981	0.257611
C	6.083813	-0.587806	0.237249
C	6.502447	-1.855789	-0.132138
C	2.942802	1.504710	0.288818
C	4.281579	1.117027	0.586381
C	2.522990	2.799205	0.693303
C	3.391980	3.694335	1.296158
C	4.725218	3.324265	1.535984
C	5.149934	2.049340	1.198288
C	2.320633	-0.839131	-0.131819
H	1.461857	2.774945	-1.610937

H	-0.774394	3.684504	-2.152940
H	0.549934	-5.136256	0.279142
H	-1.510417	-4.467121	-1.024644
H	-5.257664	3.246984	-1.571313
H	-3.949589	3.657268	-2.701171
H	-4.481261	1.966168	-2.540345
H	-1.837522	-2.066095	-1.552589
H	2.298481	-3.464480	0.692677
H	-2.486371	4.843486	-0.565680
H	-3.873689	4.484129	0.469386
H	-2.248823	3.906322	0.923827
H	-4.973537	2.631111	1.254638
H	-5.657848	0.666730	2.693191
H	-5.800014	-1.803483	2.864131
H	-3.483567	-2.142540	-1.499219
H	-5.619932	-4.006877	1.718000
H	-4.579751	-4.102298	-0.562692
H	5.865105	-3.788497	-0.887814
H	3.491496	-3.217700	-0.886979
H	6.835412	0.161423	0.484592
H	7.567225	-2.098741	-0.147291
H	1.481050	3.087974	0.573941
H	3.027759	4.676636	1.605399
H	5.415632	4.022069	2.015030
H	6.171369	1.755644	1.438988

En-TS4

E = -1504.395437 Hartree

Symbol	X	Y	Z
C	-1.728338	1.016874	0.081857
C	-3.047883	0.383506	-0.072657
N	-4.017777	1.316811	0.003719
B	-3.428124	2.823590	-0.093350
C	-1.882136	2.400337	-0.144138
C	-3.411054	-0.959670	-0.438630
C	-4.801094	-1.308099	-0.417328
C	-5.760666	-0.291809	-0.143602
C	-5.336033	1.001613	-0.003006
C	-2.477750	-1.922086	-0.905026
C	-2.888290	-3.191851	-1.253507
C	-4.248158	-3.562382	-1.139611
C	-5.187851	-2.635594	-0.738521
C	2.024926	0.617343	0.191369
C	2.171653	-0.783980	0.267887
C	3.500965	-1.347049	-0.051712
C	4.523347	-0.481612	-0.565243
C	4.427823	0.950819	-0.325627
C	3.209834	1.469306	0.177541

C	0.706470	1.231057	0.078551
C	-0.445132	0.454849	0.364158
C	-0.237055	-0.850605	0.950263
C	0.984812	-1.553507	0.689159
C	0.519894	2.564719	-0.383263
C	-0.729820	3.159981	-0.400020
C	5.545390	1.811545	-0.432369
C	5.492462	3.120318	0.017166
C	4.322822	3.597927	0.631811
C	3.205379	2.783414	0.708554
C	5.039602	-3.229092	-0.472596
C	3.867626	-2.714500	0.060576
C	5.681756	-1.030439	-1.157935
C	5.935074	-2.390339	-1.147706
C	0.925983	-2.951658	0.948876
C	-0.119669	-3.543792	1.642251
C	-1.184049	-2.768332	2.122882
C	-1.246524	-1.441971	1.741736
H	-6.824531	-0.531608	-0.118989
H	-6.021013	1.843673	0.103585
H	-4.557183	-4.578119	-1.397476
H	-6.247029	-2.899417	-0.692969
H	1.379489	3.135691	-0.726721
H	-0.823028	4.213381	-0.678294
H	6.488100	1.429990	-0.823076
H	6.374784	3.759695	-0.060885
H	4.296216	4.601203	1.062972
H	2.315619	3.147817	1.219579
H	5.255295	-4.294086	-0.359444
H	3.251251	-3.405801	0.613714
H	1.701226	-3.614978	0.597528
H	-0.090053	-4.619650	1.830086
H	-1.969828	-3.209754	2.739110
H	-2.097528	-0.829115	2.038243
C	-4.008533	3.487781	-1.461300
H	-5.095449	3.678762	-1.410253
H	-3.524262	4.465880	-1.627162
H	-3.816879	2.872714	-2.357389
C	-3.798590	3.664803	1.249146
H	-3.332177	4.664117	1.213138
H	-4.887934	3.826886	1.342499
H	-3.451282	3.175266	2.175193
H	6.837108	-2.790728	-1.615501
H	6.407476	-0.361331	-1.619525
H	-1.431393	-1.648419	-1.010552
H	-2.155086	-3.914657	-1.616614

TS5

E = -1504.366743 Hartree

Symbol	X	Y	Z
C	4.778420	-3.107331	1.495019
C	3.561447	-2.629788	1.037139
C	3.447669	-1.366634	0.402285
C	4.594800	-0.523388	0.382055
C	5.836692	-1.052419	0.807450
C	5.937972	-2.329495	1.334170
C	3.102096	1.403144	-0.129644
C	4.419458	0.894800	0.088051
C	2.916707	2.802254	0.022388
C	3.982432	3.673511	0.180308
C	5.294982	3.174906	0.202905
C	5.498124	1.805283	0.186392
C	-1.721063	0.808462	-0.672628
C	-0.448628	0.115324	-0.793306
C	0.716252	0.939972	-0.933287
C	0.551235	2.230033	-1.499623
C	-0.680539	2.852153	-1.494520
C	-0.165560	-1.317228	-0.850861
C	1.157518	-1.812225	-0.622271
C	2.204441	-0.893842	-0.197061
C	2.014911	0.468264	-0.446047
C	0.449687	-4.035907	-1.387512
C	-0.850193	-3.555173	-1.593168
B	-2.953803	2.910592	-0.069046
C	-1.119683	-2.218569	-1.352199
C	1.430207	-3.167250	-0.933202
C	-4.328822	1.532937	1.695806
C	-4.812678	0.345545	2.176269
C	-4.617648	-0.820244	1.387070
C	-2.993723	0.454559	0.040899
C	-3.805722	-0.730911	0.202281
C	-5.337467	-2.017197	1.651063
C	-4.037349	-1.705449	-0.805546
C	-5.409153	-3.016030	0.703384
C	-4.815498	-2.819545	-0.565293
C	-1.760582	2.210968	-0.871353
N	-3.460652	1.564965	0.656318
H	4.831224	-4.082258	1.984793
H	2.668007	-3.231394	1.191760
H	6.733297	-0.435441	0.762332
H	6.907785	-2.708180	1.664602
H	1.422296	2.755318	-1.886601
H	-0.774137	3.884091	-1.843859
H	0.706503	-5.070441	-1.626151
H	-1.632552	-4.203791	-1.993947

H	-2.073415	-1.820524	-1.643255
H	2.452423	-3.530975	-0.862851
H	-4.581016	2.500513	2.130294
H	-5.437636	0.314548	3.069233
H	-5.891324	-2.097011	2.589082
H	-3.687837	-1.517353	-1.817128
H	-5.986954	-3.921543	0.900967
H	-4.994950	-3.536883	-1.368577
H	6.146129	3.854144	0.288102
H	6.514284	1.427449	0.292662
H	1.910373	3.210292	0.046616
H	3.794796	4.743510	0.294389
C	-2.406148	3.998400	1.007660
H	-1.979132	4.869023	0.479910
H	-3.210341	4.388279	1.658048
H	-1.613450	3.594396	1.661691
C	-4.203543	3.456270	-0.964514
H	-5.040733	3.805355	-0.333813
H	-3.876044	4.317671	-1.572830
H	-4.600309	2.694533	-1.657829

TS6
E = -1504.398158 Hartree

Symbol	X	Y	Z
N	-4.130034	1.016146	-0.256849
B	-3.714332	2.531433	-0.646124
C	-3.058233	0.246697	0.019190
C	-3.248091	-0.966193	0.766244
C	-4.589194	-1.439277	0.945630
C	-5.669543	-0.642685	0.470252
C	-5.403577	0.588409	-0.065771
C	-2.188252	-1.672074	1.394829
C	-2.435506	-2.830342	2.101723
C	-3.750744	-3.343149	2.202985
C	-4.808488	-2.655914	1.644530
C	3.197842	3.053267	0.288416
C	4.159793	3.724556	1.030104
C	5.126075	3.006732	1.745987
C	5.182325	1.633887	1.578174
C	-1.050353	3.193914	-0.644664
C	0.250188	2.746105	-0.472453
C	-1.822864	0.943468	-0.379204
C	-2.127499	2.302959	-0.562731
C	2.189287	-0.542207	-0.483167
C	1.978406	0.838109	-0.288821
C	0.601989	1.365274	-0.425338
C	-0.489985	0.447143	-0.550274
C	-0.229779	-0.928936	-0.905263

C	1.076647	-1.453713	-0.723764
C	3.553887	-1.076011	-0.460917
C	4.531276	-0.416293	0.324164
C	4.254085	0.943896	0.767303
C	3.124952	1.636752	0.217256
C	3.963045	-2.194660	-1.228248
C	5.231709	-2.739124	-1.099115
C	6.144094	-2.174133	-0.193025
C	5.803309	-1.014240	0.484598
C	-1.242566	-1.765570	-1.436860
C	-1.048311	-3.125452	-1.583523
C	0.163076	-3.691850	-1.146096
C	1.200897	-2.871681	-0.737376
H	-6.698881	-0.982355	0.590774
H	-6.187499	1.286985	-0.360306
H	-3.929060	-4.272537	2.748533
H	-5.831795	-3.021428	1.754770
H	2.526776	3.675917	-0.283139
H	4.153143	4.816683	1.047475
H	5.854889	3.519187	2.377522
H	5.984486	1.075883	2.060311
H	-1.227419	4.264593	-0.780493
H	1.004644	3.512409	-0.427846
H	3.282093	-2.618741	-1.963079
H	5.520895	-3.595266	-1.712709
H	7.138412	-2.608207	-0.066242
H	6.558391	-0.528832	1.101907
H	-2.194470	-1.324879	-1.728562
H	-1.841425	-3.754197	-1.993565
H	0.299283	-4.775605	-1.148099
H	2.127201	-3.334641	-0.409310
C	-4.212435	2.886711	-2.155060
H	-5.315260	2.903256	-2.227316
H	-3.857572	3.890030	-2.448872
H	-3.838204	2.173473	-2.910080
C	-4.329292	3.521539	0.491035
H	-3.955874	4.547918	0.327361
H	-5.432330	3.573020	0.453287
H	-4.043118	3.229558	1.516639
H	-1.609737	-3.354412	2.586954
H	-1.174689	-1.283500	1.338140

Inversion of EH3
PMM (**C1**)
E = -1657.975594 Hartree

Symbol	X	Y	Z
C	0.482115	3.526388	-0.600489
C	-0.754340	2.922013	-0.423687

C	-0.877808	1.625053	0.144576
C	0.318678	0.903571	0.435863
C	-2.166217	-0.447899	0.449368
C	-1.030163	-1.042832	1.140300
C	0.196079	-0.324224	1.200628
C	-1.149529	-2.231403	1.901859
C	-0.103148	-2.694123	2.684867
C	1.095355	-1.963959	2.763446
C	1.229293	-0.789403	2.041895
C	1.556867	1.452235	-0.002493
C	2.882765	0.817927	-0.125462
N	3.833778	1.784337	-0.163018
B	3.206386	3.249469	-0.468189
C	1.666054	2.805300	-0.381727
C	3.279854	-0.550038	-0.260326
C	4.647535	-0.844686	0.026338
C	5.578377	0.217043	0.145966
C	5.147126	1.508195	-0.043893
C	2.413233	-1.634779	-0.692970
C	2.860056	-2.980991	-0.518383
C	4.186720	-3.231396	-0.033150
C	5.065110	-2.209272	0.172347
C	1.184349	-1.429412	-1.362874
C	0.389328	-2.494483	-1.750847
C	0.788736	-3.818759	-1.485076
C	2.015900	-4.054442	-0.890659
C	3.716575	3.694879	-1.949706
C	3.619896	4.299580	0.703841
C	-2.165375	0.943361	0.276946
C	-4.218803	-3.308603	-0.924162
C	-3.174919	-2.599384	-0.351575
C	-3.298350	-1.220521	-0.042150
C	-4.514850	-0.557523	-0.375600
C	-5.565186	-1.305024	-0.954152
C	-5.430844	-2.659837	-1.216036
C	-3.459163	1.619127	0.208371
C	-4.633519	0.869939	-0.102035
C	-3.616121	2.977075	0.586362
C	-4.857027	3.594686	0.586318
C	-6.000800	2.870610	0.211796
C	-5.884447	1.527446	-0.109687
H	0.527941	4.555966	-0.967013
H	-1.647647	3.455143	-0.740650
H	-2.098765	-2.764186	1.920990
H	-0.230282	-3.605200	3.274001
H	1.906875	-2.303757	3.410474
H	2.138351	-0.198496	2.144531
H	6.632093	0.005719	0.330510
H	5.824209	2.362627	-0.076946

H	4.501665	-4.268229	0.107097
H	6.100503	-2.407430	0.457064
H	0.860580	-0.417562	-1.594638
H	-0.556180	-2.301293	-2.261652
H	0.145826	-4.653339	-1.773297
H	2.364410	-5.076460	-0.723937
H	3.223005	4.638042	-2.244096
H	4.805579	3.880046	-1.978960
H	3.483568	2.946901	-2.727664
H	3.123160	5.271351	0.536628
H	3.330404	3.954793	1.711968
H	4.707555	4.496261	0.717085
H	-4.087912	-4.365636	-1.166744
H	-2.223746	-3.101298	-0.183717
H	-2.752792	3.537593	0.938599
H	-4.942968	4.637546	0.899777
H	-6.981842	3.350780	0.207897
H	-6.787253	0.962020	-0.340579
H	-6.255705	-3.211986	-1.671884
H	-6.495375	-0.806234	-1.226454

PMP (**C2**)
E = -1657.959043 Hartree

Symbol	X	Y	Z
C	-0.405758	3.445574	0.541803
C	0.802335	2.769337	0.539286
C	0.934924	1.475415	-0.036244
C	-0.253077	0.789154	-0.424600
C	2.297455	-0.567001	-0.422635
C	1.063991	-1.281253	-0.753060
C	-0.124180	-0.530719	-1.005729
C	0.943745	-2.692013	-0.865818
C	-0.157404	-3.287345	-1.459381
C	-1.183120	-2.489626	-1.995835
C	-1.172156	-1.131173	-1.739089
C	-1.511088	1.414828	-0.148102
C	-2.888285	0.871534	-0.110043
N	-3.766895	1.894283	-0.274740
B	-3.084443	3.334078	0.009001
C	-1.584272	2.789337	0.157004
C	-3.411537	-0.436749	0.153911
C	-4.767295	-0.669216	-0.234034
C	-5.589885	0.424733	-0.599210
C	-5.077098	1.695991	-0.514690
C	-2.692001	-1.508808	0.823707
C	-3.258754	-2.821656	0.829734
C	-4.550596	-3.042039	0.248676
C	-5.296242	-2.002295	-0.220075
C	-1.495106	-1.306278	1.549917

C	-0.845984	-2.357420	2.175250
C	-1.368083	-3.663248	2.109933
C	-2.566721	-3.884522	1.456192
C	-3.748545	3.920514	1.376203
C	-3.260548	4.320130	-1.273491
C	2.232231	0.816075	-0.182293
C	5.066819	-3.173467	-0.737098
C	3.873360	-2.502038	-0.954577
C	3.608133	-1.229987	-0.384588
C	4.707842	-0.563378	0.237424
C	5.890603	-1.289536	0.508235
C	6.063208	-2.588975	0.060570
C	3.476049	1.571381	-0.024377
C	4.656046	0.887287	0.382081
C	3.577496	2.959865	-0.293040
C	4.729744	3.675484	-0.008794
C	5.834563	3.019890	0.560143
C	5.800836	1.644750	0.725394
H	-0.446096	4.477977	0.900882
H	1.671337	3.250852	0.979322
H	1.718163	-3.333430	-0.457687
H	-0.212741	-4.376024	-1.525148
H	-2.009792	-2.940225	-2.548697
H	-2.001544	-0.511844	-2.076345
H	-6.632672	0.262073	-0.873201
H	-5.681955	2.593137	-0.652315
H	-4.952332	-4.058157	0.243417
H	-6.312184	-2.160333	-0.587624
H	-1.082790	-0.304611	1.638701
H	0.079263	-2.166162	2.722900
H	-0.841284	-4.489422	2.592349
H	-3.008857	-4.883403	1.430813
H	-3.223450	4.844769	1.676167
H	-4.813330	4.185050	1.245135
H	-3.680830	3.215951	2.223573
H	-4.320621	4.579511	-1.449391
H	-2.724921	5.270249	-1.102203
H	-2.863585	3.885583	-2.207592
H	5.227866	-4.152093	-1.194902
H	3.141803	-2.958497	-1.613227
H	2.741369	3.474710	-0.761781
H	4.776968	4.742959	-0.235246
H	6.736393	3.579049	0.819302
H	6.698922	1.139514	1.079054
H	6.707886	-0.807047	1.043437
H	6.989636	-3.125715	0.276495

PPM (**C3**)
E = -1657.969829 Hartree

Symbol	X	Y	Z
C	0.801802	3.539931	0.553144
C	-0.481832	3.014797	0.556064
C	-0.717146	1.613862	0.478776
C	0.415586	0.757454	0.586330
C	-2.205144	-0.351275	0.646606
C	-1.117135	-1.046268	1.328689
C	0.207691	-0.539467	1.207903
C	-1.330358	-2.161460	2.175947
C	-0.280486	-2.805456	2.811382
C	1.032391	-2.333018	2.646633
C	1.260869	-1.204486	1.878600
C	1.689773	1.313265	0.264037
C	2.943887	0.636215	-0.105845
N	3.974228	1.509819	0.017663
B	3.473342	3.037306	0.216907
C	1.907846	2.703158	0.324125
C	3.205982	-0.693359	-0.567451
C	4.559214	-1.141093	-0.479006
C	5.589121	-0.201300	-0.225567
C	5.262576	1.123019	-0.061334
C	2.207668	-1.608812	-1.099500
C	2.534815	-2.996340	-1.197345
C	3.874014	-3.431232	-0.921996
C	4.861144	-2.533827	-0.642026
C	0.950832	-1.192939	-1.598634
C	0.015825	-2.108475	-2.050311
C	0.302226	-3.488096	-2.035812
C	1.554100	-3.919491	-1.635238
C	3.906813	3.874184	-1.112486
C	4.081559	3.650683	1.596346
C	-2.036372	0.998007	0.308654
C	-4.793694	-3.064812	0.095696
C	-3.572794	-2.437323	0.279103
C	-3.464932	-1.024643	0.353228
C	-4.628622	-0.249197	0.084469
C	-5.872051	-0.912252	-0.050192
C	-5.963292	-2.294039	-0.023486
C	-3.165154	1.734631	-0.264991
C	-4.477196	1.172998	-0.201551
C	-3.023444	2.982252	-0.926849
C	-4.118430	3.724363	-1.338395
C	-5.416779	3.238982	-1.111174
C	-5.582839	1.972381	-0.576852
H	0.944869	4.614371	0.697293
H	-1.327632	3.687116	0.684400
H	-2.346177	-2.499955	2.368911

H	-0.481998	-3.656385	3.465974
H	1.863835	-2.818694	3.162120
H	2.265657	-0.789710	1.839832
H	6.630770	-0.522622	-0.196690
H	6.009546	1.909440	0.052691
H	4.101128	-4.496973	-1.002160
H	5.895891	-2.858738	-0.515878
H	0.713691	-0.133416	-1.643240
H	-0.949439	-1.754340	-2.418217
H	-0.448004	-4.207239	-2.371698
H	1.814461	-4.979886	-1.674831
H	3.494693	4.897291	-1.059779
H	5.003688	3.973721	-1.202747
H	3.534660	3.419017	-2.047033
H	3.664366	4.656224	1.780776
H	3.846969	3.033159	2.481002
H	5.179866	3.765394	1.549732
H	-4.842351	-4.154422	0.035905
H	-2.671158	-3.043728	0.339059
H	-2.030037	3.361964	-1.147982
H	-3.966120	4.680368	-1.844349
H	-6.288725	3.830825	-1.398360
H	-6.592694	1.575695	-0.479178
H	-6.779342	-0.333931	-0.220477
H	-6.934941	-2.778940	-0.141400

PPP (**C4**)
E = -1657.968463 Hartree

Symbol	X	Y	Z
C	-0.500607	3.446033	-0.613671
C	0.718643	2.841908	-0.366200
C	0.843119	1.425662	-0.295648
C	-0.302859	0.624601	-0.560335
C	2.365602	-0.492672	-0.584304
C	1.213860	-1.329155	-0.894560
C	-0.095019	-0.758042	-0.953045
C	1.322983	-2.736812	-1.049834
C	0.242278	-3.517955	-1.421187
C	-0.992441	-2.907188	-1.702767
C	-1.148961	-1.555313	-1.461579
C	-1.578181	1.278700	-0.459907
C	-2.934214	0.739250	-0.220643
N	-3.853013	1.670771	-0.585881
B	-3.200622	3.131266	-0.799571
C	-1.676848	2.677186	-0.605397
C	-3.397384	-0.487064	0.360659
C	-4.764431	-0.829734	0.123709
C	-5.647358	0.141228	-0.408565
C	-5.169715	1.401671	-0.670946

C	-2.589463	-1.393794	1.161168
C	-3.079527	-2.712861	1.412224
C	-4.403932	-3.070757	0.995139
C	-5.234341	-2.149893	0.430036
C	-1.365724	-1.021506	1.764825
C	-0.606065	-1.934703	2.475786
C	-1.044851	-3.264681	2.628875
C	-2.276444	-3.637665	2.121320
C	-3.502284	3.676697	-2.303476
C	-3.782382	4.104333	0.371492
C	2.141638	0.796877	-0.088875
C	5.391650	-2.211945	-2.105989
C	4.077612	-1.886603	-1.808275
C	3.749427	-0.900840	-0.841434
C	4.823865	-0.182865	-0.238048
C	6.155281	-0.541349	-0.548794
C	6.444009	-1.550848	-1.452812
C	3.195197	1.442240	0.694436
C	4.521234	0.921803	0.663318
C	2.905815	2.471385	1.627851
C	3.885214	3.008561	2.447804
C	5.201752	2.524351	2.377186
C	5.502847	1.488002	1.507824
H	-0.550713	4.526463	-0.773871
H	1.618990	3.452325	-0.307277
H	2.273057	-3.223056	-0.841288
H	0.359950	-4.599705	-1.515863
H	-1.831516	-3.495578	-2.079783
H	-2.110493	-1.093778	-1.668364
H	-6.700300	-0.097174	-0.561626
H	-5.809624	2.227185	-0.984816
H	-4.755091	-4.086327	1.192849
H	-6.268475	-2.401156	0.185930
H	-1.016006	0.003750	1.682582
H	0.338795	-1.616183	2.921262
H	-0.430848	-3.985581	3.172982
H	-2.658304	-4.649216	2.278669
H	-2.978527	4.633479	-2.474341
H	-4.578814	3.869856	-2.463140
H	-3.167861	2.976674	-3.088983
H	-3.272416	5.082928	0.328607
H	-3.625395	3.697488	1.385835
H	-4.863431	4.300366	0.253466
H	5.602308	-2.966194	-2.867604
H	3.284407	-2.368204	-2.374500
H	1.881408	2.819714	1.742254
H	3.622895	3.790464	3.164050
H	5.978306	2.938230	3.024302
H	6.516000	1.086633	1.500025

H	7.482339	-1.801242	-1.681306
H	6.979593	0.008642	-0.095211

TS1

E = -1657.964903 Hartree

Symbol	X	Y	Z
C	0.851302	3.659423	0.060877
C	-0.438891	3.167776	0.212718
C	-0.729889	1.783509	0.390835
C	0.421999	0.943963	0.544521
C	-2.106217	-0.248117	0.648031
C	-1.014098	-0.857630	1.395051
C	0.272630	-0.279727	1.306214
C	-1.207743	-1.949821	2.275088
C	-0.154937	-2.478208	3.005940
C	1.128093	-1.912553	2.899266
C	1.329402	-0.817539	2.075877
C	-2.064589	1.131381	0.362801
C	-4.101293	-3.272666	-0.429699
C	-3.063794	-2.486324	0.039046
C	-3.209702	-1.082449	0.199729
C	-4.428947	-0.480853	-0.198557
C	-5.481976	-1.306980	-0.661657
C	-5.332066	-2.679609	-0.763562
C	-3.344395	1.773162	-0.010756
C	-4.524860	0.970475	-0.176470
C	-3.530466	3.172161	-0.175141
C	-4.767149	3.755375	-0.401176
C	-5.917794	2.961513	-0.483142
C	-5.780134	1.589961	-0.379262
C	1.686418	1.417535	0.095694
C	1.943164	2.790281	-0.063504
C	2.910443	0.653744	-0.199824
C	5.242525	1.059155	-0.246128
C	3.118623	-0.732523	-0.483692
C	5.519898	-0.286582	-0.222913
C	4.455031	-1.214531	-0.343614
C	4.703084	-2.627154	-0.330878
C	3.682947	-3.513989	-0.509455
C	2.362590	-3.065530	-0.847553
C	2.085044	-1.665324	-0.905009
C	1.353882	-3.995706	-1.199377
C	0.846314	-1.260840	-1.456689
C	0.125577	-3.566100	-1.668415
C	-0.111945	-2.186602	-1.830109
B	3.515358	3.058963	-0.246073
N	3.969064	1.500063	-0.213685
H	-2.208675	-2.356850	2.412728

H	-0.332825	-3.311033	3.689982
H	1.954615	-2.305877	3.495131
H	2.304966	-0.334652	2.059300
H	-3.953621	-4.347365	-0.558136
H	-2.102071	-2.950427	0.245758
H	-6.422334	-0.861388	-0.983062
H	-6.156764	-3.291811	-1.135380
H	-2.699255	3.853826	-0.120241
H	-4.832778	4.840950	-0.503204
H	-6.901904	3.410445	-0.633497
H	-6.672020	0.969227	-0.450212
H	6.018072	1.825961	-0.251440
H	6.549797	-0.638707	-0.155441
H	5.724539	-2.973445	-0.161030
H	3.870375	-4.589408	-0.462598
H	1.575419	-5.062690	-1.120436
H	0.644255	-0.203575	-1.608611
H	-0.645872	-4.289542	-1.940919
H	-1.060051	-1.841647	-2.246679
H	0.998091	4.741836	0.007434
H	-1.218670	3.912467	0.257063
C	3.950846	3.664719	-1.694205
H	3.570393	4.696464	-1.795534
H	5.048457	3.714153	-1.812633
H	3.549194	3.083013	-2.542318
C	4.165074	3.857149	1.014180
H	5.265588	3.925823	0.937924
H	3.783264	4.892753	1.044941
H	3.925577	3.392708	1.986779

TS2

E = -1657.957163 Hartree

Symbol	X	Y	Z
C	1.228890	-2.688957	-1.568055
C	0.122990	-3.377542	-2.040864
C	-1.124112	-2.746724	-2.109728
C	-1.226065	-1.456044	-1.633295
C	-2.934779	0.700003	-0.118995
N	-3.885016	1.622437	-0.416812
B	-3.260709	3.084486	-0.703803
C	-3.338450	-0.542676	0.469664
C	-4.713098	-0.901233	0.321183
C	-5.643412	0.067396	-0.129971
C	-5.201646	1.337107	-0.410242
C	-2.462415	-1.451055	1.193168
C	-2.913929	-2.782976	1.448595
C	-4.258483	-3.153990	1.114422
C	-5.140844	-2.234802	0.630789

C	-1.211807	-1.066551	1.731224
C	-0.397731	-1.976183	2.384110
C	-0.803228	-3.316699	2.537982
C	-2.053853	-3.705031	2.092496
C	5.601648	-2.558031	-1.224443
C	4.255947	-2.262962	-1.064685
C	6.161722	-0.384522	-0.418279
C	6.576643	-1.597640	-0.934769
C	2.094695	0.757840	-0.211476
C	2.350264	-0.567934	-0.614418
C	3.772320	-1.004427	-0.612185
C	4.797434	-0.081120	-0.206250
C	4.443351	1.134231	0.509896
C	3.081620	1.514907	0.554488
C	0.812829	1.402233	-0.464333
C	-0.338399	0.601202	-0.622867
C	-0.129509	-0.761821	-1.069150
C	1.184332	-1.350871	-1.089632
C	0.686283	2.813403	-0.605979
C	-0.550931	3.407580	-0.778883
C	-1.722068	2.642029	-0.631274
C	-1.606417	1.249651	-0.452063
C	5.393033	1.889175	1.239983
C	5.009866	2.956639	2.034480
C	3.647241	3.278479	2.155841
C	2.705904	2.565428	1.433383
H	2.146534	-3.247788	-1.589406
H	0.247354	-4.405901	-2.387549
H	-1.992823	-3.251505	-2.537106
H	-2.177821	-0.940768	-1.723995
H	-6.701437	-0.183063	-0.212359
H	-5.872513	2.156092	-0.672203
H	-4.580095	-4.178998	1.313620
H	-6.185883	-2.497831	0.455011
H	-0.885950	-0.033329	1.649269
H	0.564087	-1.646638	2.782813
H	-0.147110	-4.034587	3.034898
H	-2.407704	-4.726183	2.253494
H	5.886448	-3.546996	-1.590647
H	3.588727	-3.069197	-1.311873
H	1.589164	3.422589	-0.641237
H	-0.616497	4.479235	-0.985363
H	6.444585	1.606563	1.220540
H	5.761434	3.514589	2.597539
H	3.325418	4.073046	2.832670
H	1.651917	2.796392	1.574501
C	-3.747015	4.072162	0.497984

H	-3.252670	5.054307	0.395827
H	-4.836238	4.256422	0.471524
H	-3.498364	3.683041	1.500885
C	-3.693492	3.603703	-2.184714
H	-4.780359	3.792044	-2.254593
H	-3.189648	4.558584	-2.415628
H	-3.425191	2.891451	-2.984495
H	7.637532	-1.802641	-1.092727
H	6.913799	0.361311	-0.165515

En-TS3

E = -1657.919542 Hartree

Symbol	X	Y	Z
C	-3.624874	-3.387194	-1.836026
C	-2.768305	-2.712854	-0.981071
C	-3.069902	-1.408558	-0.513976
C	-4.270368	-0.782627	-0.957677
C	-5.124109	-1.491518	-1.832653
C	-4.816798	-2.773961	-2.261003
C	-3.579233	1.303503	0.220130
C	-4.584612	0.552784	-0.463596
C	-3.966281	2.522317	0.834724
C	-5.248841	3.031215	0.704713
C	-6.208096	2.332244	-0.046233
C	-5.878304	1.106009	-0.600733
C	0.546476	-2.016213	3.271012
C	-0.467973	-2.873324	2.820766
C	0.805189	-0.837996	2.585715
C	-1.269388	-2.500708	1.748630
C	0.051235	-0.472489	1.456410
C	-1.067106	-1.268313	1.083982
C	0.209909	0.839049	0.844869
C	-2.118535	-0.654628	0.279775
C	-2.243055	0.736105	0.389015
C	-1.025406	1.529491	0.609851
C	1.435376	1.518263	0.545153
C	-0.984333	2.932132	0.427647
C	1.426099	2.932306	0.463214
C	0.213787	3.627871	0.490815
C	3.828803	3.829432	1.313673
C	2.817839	4.719254	-1.044604
C	2.675048	1.037611	-0.161213
C	4.147896	2.095622	-1.712707
C	3.059475	-0.239298	-0.704956
C	4.207032	0.974136	-2.502754
C	3.627210	-0.219289	-2.017523
C	3.627532	-1.420116	-2.808087
C	3.237641	-2.610354	-2.271659

C	3.020777	-2.722616	-0.856798
C	3.001230	-1.537569	-0.055858
C	3.012947	-3.988862	-0.228279
C	3.190749	-1.677800	1.333022
C	3.130951	-4.093448	1.148572
C	3.272486	-2.928981	1.923910
B	2.858592	3.512593	0.037214
N	3.345951	2.126714	-0.629956
H	-3.361814	-4.384959	-2.194554
H	-1.822430	-3.174161	-0.698998
H	-3.260875	3.046273	1.475353
H	-5.514353	3.964291	1.206822
H	-7.219188	2.729684	-0.159012
H	-6.650557	0.541262	-1.122782
H	1.127395	-2.270169	4.159687
H	-0.673312	-3.806958	3.349499
H	1.565186	-0.147050	2.949737
H	-2.128078	-3.118641	1.487844
H	-1.891049	3.477451	0.181036
H	0.210375	4.716056	0.389167
H	4.859253	4.057359	0.986145
H	3.461542	4.711681	1.866272
H	3.881233	2.990499	2.029329
H	2.327337	5.600116	-0.593627
H	3.823323	5.055770	-1.355891
H	2.251097	4.467381	-1.958538
H	4.642981	3.030588	-1.971514
H	4.718541	0.993773	-3.465548
H	3.949004	-1.349754	-3.849372
H	3.209874	-3.515808	-2.882424
H	2.986590	-4.885437	-0.852289
H	3.334673	-0.782303	1.933956
H	3.163416	-5.075825	1.624739
H	3.442644	-3.007819	2.999027
H	-6.036870	-1.020114	-2.197176
H	-5.491555	-3.296931	-2.942430

TS3a

E = -1657.908865 Hartree

Symbol	X	Y	Z
C	-5.788463	2.408131	-0.836708
C	-4.439165	2.102034	-0.921924
C	-3.878720	0.970624	-0.272607
C	-4.781553	0.083351	0.387365
C	-6.150028	0.427141	0.477456
C	-6.651538	1.580758	-0.102113
C	-2.991049	-1.628763	0.518023
C	-4.285795	-1.183065	0.910544

C	-2.514942	-2.852542	1.057882
C	-3.291946	-3.626988	1.903727
C	-4.580328	-3.196967	2.262090
C	-5.055996	-1.987929	1.780513
C	0.474997	3.222163	-2.059033
C	-0.661945	3.775299	-1.449939
C	0.756270	1.881961	-1.862307
C	-1.584883	2.942829	-0.835310
C	-0.108208	1.038499	-1.134073
C	-1.409225	1.535158	-0.789722
C	0.231254	-0.359481	-0.959911
C	-2.462445	0.604407	-0.402112
C	-2.128062	-0.748382	-0.267113
C	-0.871620	-1.244335	-0.819876
C	1.537985	-0.980677	-1.056609
C	-0.764435	-2.559776	-1.343054
C	1.609730	-2.291505	-1.576347
C	0.437506	-3.013979	-1.855172
C	3.984632	-2.552771	-2.841367
C	3.206340	-4.427665	-1.038057
C	2.798101	-0.738649	-0.275960
C	4.412938	-2.237767	0.641452
C	3.146488	0.216440	0.746446
C	4.435374	-1.562595	1.837541
C	3.762846	-0.322219	1.918424
C	3.712301	0.422735	3.147715
C	3.227250	1.695930	3.174482
C	2.970139	2.389581	1.943161
C	3.008117	1.662579	0.711780
C	2.879378	3.800160	1.917227
C	3.192599	2.396227	-0.478190
C	2.982479	4.491885	0.720609
C	3.198079	3.782578	-0.474356
B	3.102495	-2.878745	-1.505087
N	3.561849	-1.867207	-0.335836
H	-0.843219	4.851803	-1.487581
H	3.617538	-3.143443	-3.698585
H	5.046437	-2.820411	-2.693738
H	3.942389	-1.488567	-3.131478
H	2.805795	4.339646	2.864594
H	3.401351	1.856334	-1.399767
H	2.953138	5.583640	0.712364
H	3.372345	4.324566	-1.405686
H	-3.814443	2.722121	-1.557264
H	-6.177491	3.281300	-1.365499
H	-1.497828	-3.174349	0.844295
H	-2.888109	-4.557041	2.310070

H	-5.195017	-3.796071	2.937621
H	-6.039326	-1.645909	2.102139
H	2.732997	-5.073043	-1.799053
H	2.695260	-4.630523	-0.080125
H	-1.653113	-3.190071	-1.384452
H	0.496523	-3.997902	-2.326653
H	1.639930	1.434773	-2.310526
H	1.151137	3.846645	-2.644710
H	3.156754	2.247530	4.114923
H	4.069868	-0.063523	4.057839
H	4.988046	-1.953820	2.692238
H	4.979205	-3.151348	0.465335
H	4.248987	-4.781086	-0.942710
H	-2.465403	3.388521	-0.381404
H	-6.840689	-0.248521	0.981153
H	-7.715219	1.816414	-0.024524

En-TS4

E = -1657.957448 Hartree

Symbol	X	Y	Z
C	-1.498056	1.431597	0.045052
C	-2.869318	0.882285	-0.017788
N	-3.755440	1.909615	0.030557
B	-3.058550	3.333625	-0.301981
C	-1.550060	2.790334	-0.325611
C	-3.372645	-0.448208	-0.188535
C	-4.740751	-0.658955	0.165081
C	-5.584069	0.456070	0.395616
C	-5.074709	1.720511	0.226485
C	-2.620172	-1.566661	-0.735104
C	-3.174611	-2.881403	-0.641976
C	-4.484257	-3.063372	-0.087341
C	-5.257186	-1.994121	0.254115
C	-1.405888	-1.413686	-1.444180
C	-0.731753	-2.506919	-1.961958
C	-1.242670	-3.808040	-1.796029
C	-2.455136	-3.986458	-1.154222
C	2.216271	0.790117	0.260521
C	2.260422	-0.607713	0.449761
C	3.551224	-1.285988	0.197653
C	4.644938	-0.534064	-0.349075
C	4.647113	0.916111	-0.221186
C	3.457179	1.556179	0.204033
C	0.945593	1.482025	0.071836
C	-0.260725	0.819613	0.413786
C	-0.155065	-0.450957	1.095072
C	1.015623	-1.256433	0.907266
C	0.861467	2.768796	-0.529916

C	-0.345460	3.438958	-0.640960
C	5.825429	1.686576	-0.366576
C	5.853733	3.029933	-0.028937
C	4.705142	3.637387	0.507403
C	3.531738	2.909729	0.619973
C	4.954450	-3.300049	-0.061269
C	3.812126	-2.666978	0.406160
C	5.774176	-1.203939	-0.871037
C	5.926239	-2.575256	-0.763315
C	0.851329	-2.625525	1.255089
C	-0.254019	-3.098241	1.945769
C	-1.270069	-2.220016	2.349472
C	-1.220792	-0.916401	1.895642
H	-6.636939	0.308579	0.637824
H	-5.689488	2.620623	0.264806
H	-4.876589	-4.079654	-0.002331
H	-6.285072	-2.130451	0.596368
H	-1.000662	-0.418314	-1.607941
H	0.205470	-2.352817	-2.500894
H	-0.696247	-4.666620	-2.192432
H	-2.888325	-4.984511	-1.053894
H	1.764207	3.244461	-0.906475
H	-0.360055	4.462087	-1.027464
H	6.747755	1.211773	-0.699345
H	6.780086	3.599026	-0.134671
H	4.737717	4.674337	0.848979
H	2.657729	3.379115	1.068730
H	5.085512	-4.368515	0.124401
H	3.133112	-3.277599	0.979855
H	1.578775	-3.367174	0.964826
H	-0.312389	-4.161283	2.189639
H	-2.103742	-2.565956	2.963681
H	-2.027846	-0.227402	2.140145
C	-3.639372	3.831338	-1.740358
H	-4.712048	4.091964	-1.690987
H	-3.102734	4.741422	-2.062308
H	-3.514677	3.078337	-2.538107
C	-3.316314	4.394177	0.904958
H	-2.770763	5.334095	0.710232
H	-4.385266	4.659894	0.997966
H	-2.978802	4.018417	1.886772
H	6.807037	-3.070176	-1.177913
H	6.558495	-0.623218	-1.355670

TS5

E = -1657.918035 Hartree

Symbol	X	Y	Z
C	-0.740340	-2.726632	-2.699415
C	0.477787	-3.373301	-2.450318
C	-0.979688	-1.482616	-2.137914
C	1.441429	-2.751808	-1.667332
C	-0.048225	-0.859500	-1.285819
C	1.213898	-1.489491	-1.071472
C	-0.234862	0.542055	-0.940814
C	2.270809	-0.731641	-0.406730
C	2.201254	0.664497	-0.465854
C	0.973176	1.302089	-0.958649
C	-1.466157	1.278744	-0.796041
C	0.901292	2.626613	-1.459962
C	-1.481418	2.652071	-1.130615
C	-0.320491	3.255741	-1.638300
C	-4.005081	3.480341	-1.712202
C	-2.534922	4.783000	0.167238
C	-2.628829	1.011179	0.107218
C	-3.855391	2.410128	1.591454
C	-3.037050	-0.129410	0.879026
C	-3.878989	1.437530	2.563643
C	-3.451082	0.133946	2.220304
C	-3.459597	-0.943081	3.173551
C	-3.228723	-2.227975	2.779024
C	-3.179969	-2.556315	1.380836
C	-3.162488	-1.499471	0.419607
C	-3.330492	-3.891728	0.939985
C	-3.531832	-1.804281	-0.907720
C	-3.603723	-4.169523	-0.390153
C	-3.759622	-3.111811	-1.305684
B	-2.809716	3.382268	-0.603762
N	-3.198657	2.204058	0.432497
C	4.502558	-3.385712	1.112266
C	3.380215	-2.747958	0.611523
C	3.414043	-1.387691	0.210359
C	4.603254	-0.643028	0.454090
C	5.748829	-1.327980	0.926182
C	5.711542	-2.678035	1.232738
C	3.321185	1.455836	0.047788
C	4.561519	0.810556	0.344437
C	3.231755	2.848009	0.311557
C	4.336300	3.599296	0.677299
C	5.590636	2.980518	0.807457
C	5.686355	1.606490	0.667369
H	-1.480194	-3.175591	-3.364711
H	0.696432	-4.337164	-2.915547

H	-1.861624	-0.920643	-2.426861
H	2.418583	-3.220807	-1.572004
H	1.820093	3.153573	-1.711877
H	-0.353564	4.276361	-2.028175
H	-3.733448	4.201401	-2.502854
H	-4.212297	2.513971	-2.204071
H	-4.947673	3.835984	-1.258475
H	-3.454663	5.231145	0.585152
H	-2.126587	5.522776	-0.543709
H	-1.804490	4.689264	0.990401
H	-4.269378	3.407274	1.738139
H	-4.266808	1.652006	3.559934
H	-3.656588	-0.703194	4.220634
H	-3.205046	-3.039265	3.510502
H	-3.298180	-4.696470	1.678409
H	-3.693022	-0.985614	-1.608350
H	-3.752464	-5.201344	-0.715882
H	-4.062972	-3.319991	-2.333784
H	4.443255	-4.432429	1.418955
H	2.443350	-3.298342	0.547768
H	2.266620	3.343273	0.254998
H	4.224411	4.668460	0.871088
H	6.475319	3.567737	1.063541
H	6.650304	1.130844	0.843750
H	6.677723	-0.783173	1.090076
H	6.608898	-3.177686	1.604547

TS6

E = -1657.951589 Hartree

Symbol	X	Y	Z
N	-3.990852	1.488724	-0.292765
B	-3.510204	3.031611	-0.405773
C	-2.952993	0.640567	-0.080761
C	-3.226867	-0.666893	0.438604
C	-4.562260	-1.146154	0.275521
C	-5.585311	-0.248510	-0.120065
C	-5.273440	1.075703	-0.307248
C	-2.267747	-1.514527	1.130709
C	-2.602389	-2.885223	1.361621
C	-3.902013	-3.371090	0.999519
C	-4.861761	-2.525598	0.529128
C	-1.051332	-1.035802	1.672159
C	-0.168870	-1.881781	2.322498
C	-0.464912	-3.250857	2.467892
C	-1.674128	-3.737952	2.005241
C	3.423067	3.122942	0.468316
C	4.421295	3.648770	1.276524
C	5.361327	2.802367	1.877463

C	5.352288	1.461888	1.532663
C	-0.821715	3.575107	-0.365546
C	0.460425	3.055032	-0.294551
C	-1.689587	1.343687	-0.384419
C	-1.936212	2.725967	-0.373095
C	2.238097	-0.297550	-0.733552
C	2.098231	1.059789	-0.376958
C	0.749260	1.665087	-0.436924
C	-0.385171	0.822565	-0.664431
C	-0.198113	-0.517364	-1.166177
C	1.080932	-1.121168	-1.058352
C	3.573938	-0.896179	-0.795787
C	4.592978	-0.381140	0.042156
C	4.385535	0.924769	0.654012
C	3.285185	1.732719	0.212820
C	3.915398	-1.936632	-1.694976
C	5.157476	-2.551540	-1.655919
C	6.110655	-2.138591	-0.710508
C	5.837115	-1.051726	0.104681
C	-1.256706	-1.246170	-1.762244
C	-1.136569	-2.594605	-2.034437
C	0.050684	-3.259797	-1.674564
C	1.135185	-2.534573	-1.214262
H	-6.614172	-0.596414	-0.217251
H	-6.023914	1.842958	-0.501372
H	-4.128214	-4.426714	1.167987
H	-5.875728	-2.880203	0.333328
H	-0.808661	0.020763	1.599309
H	0.762478	-1.478082	2.725379
H	0.243822	-3.915789	2.966263
H	-1.942118	-4.787100	2.151188
H	2.773884	3.844273	-0.003143
H	4.463065	4.728363	1.437224
H	6.118697	3.195497	2.558956
H	6.131235	0.810148	1.927328
H	-0.954039	4.660669	-0.354863
H	1.250030	3.777036	-0.178047
H	3.202270	-2.240465	-2.458374
H	5.394706	-3.343591	-2.369694
H	7.084718	-2.629942	-0.656423
H	6.624261	-0.677603	0.758360
H	-2.183985	-0.731501	-2.004944
H	-1.966728	-3.141437	-2.486377
H	0.129062	-4.344412	-1.776525
H	2.040604	-3.071690	-0.946405
C	-3.990249	3.677086	-1.820358
H	-5.090753	3.760282	-1.882173

H	-3.587442	4.699276	-1.929483
H	-3.649193	3.096920	-2.695598
C	-4.090278	3.817858	0.897713
H	-3.689550	4.846784	0.918933
H	-5.191863	3.902898	0.879251
H	-3.808189	3.337068	1.850749

(*P*)-Azabora[5]helicene H1
E = -890.224773 Hartree

Symbol	X	Y	Z
C	-0.463572	-0.452039	0.013530
C	0.938334	-0.743991	-0.045332
C	1.289899	-2.113525	-0.259176
C	0.300112	-3.127164	-0.166394
C	-1.015429	-2.817632	0.123685
C	2.024056	0.206291	0.152231
C	3.359835	-0.171559	-0.197156
C	3.632307	-1.519445	-0.605162
C	2.648293	-2.461971	-0.568431
C	4.188068	2.011774	0.506573
C	2.896017	2.344982	0.962793
B	-2.901515	-0.866253	0.345939
C	-3.969500	-1.369643	-0.774373
C	1.845694	1.458830	0.792436
C	4.413482	0.764609	-0.047108
H	0.868949	1.716891	1.197746
H	5.011978	2.718827	0.627410
H	5.421482	0.467620	-0.347781
H	4.656545	-1.788861	-0.873779
H	2.872844	-3.508403	-0.789723
H	0.609579	-4.165941	-0.311127
H	-1.752490	-3.616002	0.248005
H	-4.157740	-2.450736	-0.650340
H	-3.612841	-1.218082	-1.808421
H	-4.947888	-0.864316	-0.680846
C	-3.462643	-0.974847	1.871421
H	-3.654805	-2.030017	2.133331
H	-4.418860	-0.434038	1.994362
H	-2.754289	-0.576568	2.618795
C	-3.373717	1.677479	-0.131527
C	-2.962395	2.922795	-0.583824
C	-1.611045	3.090212	-0.914776
C	-1.171071	0.821380	-0.207476
C	-0.714208	2.043385	-0.730082
H	-4.417768	1.452212	0.092711
H	0.331466	2.162071	-1.001509
H	2.723337	3.298354	1.467441
H	-3.686656	3.728438	-0.705100
H	-1.258217	4.039312	-1.323807

C	-1.422815	-1.470579	0.176123
N	-2.506466	0.669210	0.025013

TS-Azabora[5]helicene H1

E = -890.202094 Hartree

Symbol	X	Y	Z
C	-0.443810	-0.338990	-0.182874
C	0.997681	-0.625808	-0.173217
C	1.348472	-2.026419	-0.169022
C	0.380527	-3.056128	-0.263538
C	-0.956046	-2.759265	-0.246186
C	2.151856	0.289598	-0.088854
C	3.475984	-0.216099	0.170077
C	3.724481	-1.620350	0.212171
C	2.699725	-2.480877	-0.002468
C	4.427991	2.036027	0.209315
C	3.162170	2.544394	-0.127529
B	-2.869769	-0.995053	0.094192
C	-3.863618	-1.303442	-1.163045
C	2.083591	1.688168	-0.271337
C	4.570956	0.668607	0.337006
H	1.154158	2.116380	-0.580317
H	5.283472	2.702800	0.337856
H	5.549728	0.233354	0.553406
H	4.741859	-1.976432	0.388294
H	2.872034	-3.559399	-0.009841
H	0.729759	-4.091133	-0.297835
H	-1.708670	-3.551901	-0.263712
H	-3.955562	-2.393776	-1.310048
H	-3.498725	-0.874825	-2.112985
H	-4.884561	-0.916844	-0.991484
C	-3.455384	-1.526892	1.519450
H	-3.548728	-2.626702	1.505904
H	-4.463658	-1.125510	1.729510
H	-2.806665	-1.264604	2.373605
C	-3.587653	1.448663	0.342637
C	-3.383726	2.813412	0.230481
C	-2.107176	3.221648	-0.151430
C	-1.297546	0.910277	-0.119078
C	-1.088296	2.288695	-0.333635
H	-4.565470	1.019979	0.567472
H	-0.157191	2.693823	-0.689951
H	3.020703	3.615233	-0.291234
H	-4.200255	3.516303	0.394315
H	-1.890617	4.277749	-0.325065
C	-1.360487	-1.420597	-0.150410
N	-2.600132	0.567576	0.154295

(*M*)-Azabora[6]helicene H2
E = -1043.774204 Hartree

Symbol	X	Y	Z
C	0.338141	-1.035095	-0.060501
C	1.211174	0.148223	0.013913
N	2.456085	-0.215973	0.382414
B	2.664395	-1.817073	0.332293
C	1.158277	-2.182905	-0.085093
C	0.595102	-3.424799	-0.434745
C	-0.741999	-3.501732	-0.780448
C	-1.606100	-2.387751	-0.612235
C	-1.090101	-1.147759	-0.121782
C	0.977503	1.508410	-0.384982
C	2.002992	2.473356	-0.114100
C	3.237638	2.028357	0.436873
C	3.442283	0.686047	0.613191
C	-3.007712	-2.510543	-0.900650
C	-3.877889	-1.494943	-0.636036
C	-3.432765	-0.320401	0.057290
C	-2.046045	-0.169372	0.374267
C	-0.175736	1.924447	-1.101317
C	-0.341796	3.245773	-1.461617
C	0.630916	4.213106	-1.113487
C	1.785481	3.833046	-0.461153
C	-4.360403	0.655021	0.501517
C	-3.958033	1.712892	1.296608
C	-2.609035	1.808982	1.696082
C	-1.680109	0.887685	1.245047
C	3.054576	-2.384893	1.808180
C	3.789405	-2.137199	-0.801711
H	1.225456	-4.317562	-0.476861
H	-1.171516	-4.439538	-1.143444
H	4.033020	2.742178	0.654380
H	4.396859	0.271901	0.939522
H	-3.361950	-3.444821	-1.343383
H	-4.938438	-1.588076	-0.881723
H	-0.922644	1.189185	-1.388989
H	-1.229322	3.546764	-2.021807
H	0.476543	5.259096	-1.387708
H	2.561242	4.565403	-0.227489
H	-5.412075	0.537698	0.227847
H	-4.685770	2.451140	1.641278
H	-2.294403	2.608645	2.370355
H	-0.650551	0.965500	1.589534
H	2.316448	-2.111595	2.582290
H	3.116317	-3.486976	1.789390
H	4.041552	-2.014711	2.140767
H	3.862073	-3.229018	-0.950981

H	3.552727	-1.688437	-1.782328
H	4.795297	-1.787318	-0.507690

TS-Azabora[6]helicene H2
E = -1043.727547 Hartree

Symbol	X	Y	Z
C	-0.297442	-0.984148	0.462831
C	1.131460	-1.175250	0.255898
C	1.553925	-2.519125	-0.022365
C	0.712143	-3.621568	0.260054
C	-0.602267	-3.423245	0.619132
C	2.201466	-0.180535	0.195078
C	3.376303	-0.435734	-0.587761
C	3.628169	-1.761115	-1.068898
C	2.804044	-2.773974	-0.686008
C	4.255302	1.775202	-0.045460
C	3.211638	1.944558	0.882795
B	-2.668804	-1.797474	0.264545
C	-3.627488	-1.673437	1.582256
C	2.225106	0.977679	0.995968
C	4.348652	0.582001	-0.742549
H	5.021432	2.545109	-0.162698
H	5.210998	0.383260	-1.384120
H	4.532480	-1.953053	-1.650988
H	3.041047	-3.810891	-0.936410
H	1.108152	-4.630086	0.112957
H	-1.265434	-4.277223	0.780358
H	-3.762425	-2.668000	2.042202
H	-3.209705	-1.005274	2.355282
H	-4.633156	-1.297847	1.320725
C	-3.307734	-2.731355	-0.901697
H	-3.380461	-3.775346	-0.550206
H	-4.333563	-2.424990	-1.175820
H	-2.703579	-2.742442	-1.826024
C	-3.290282	0.404398	-0.989444
C	-3.021122	1.687562	-1.379810
C	-1.924371	2.352738	-0.770400
C	-1.203732	0.177661	0.152027
C	-1.080593	1.619826	0.138201
H	-4.195261	-0.129727	-1.278105
H	3.186375	2.822765	1.531977
H	-3.692486	2.222070	-2.052585
C	-1.744211	3.754212	-0.918901
H	-2.348102	4.281295	-1.660807
C	-0.338964	2.387658	1.071664
C	-0.890603	4.444880	-0.084832
H	-0.775357	5.526137	-0.187457
C	-0.241422	3.760569	0.967614

H	0.320133	4.320028	1.718430
H	0.069793	1.896734	1.944791
H	1.496125	1.052463	1.788513
C	-1.134189	-2.125047	0.568092
N	-2.400818	-0.304869	-0.253380

(*P*)-Carbo[4]helicene
E = -692.758602 Hartree

Symbol	X	Y	Z
C	0.000000	0.398019	0.000000
C	0.000000	1.818310	0.000000
C	-1.219651	2.544739	-0.184515
C	-2.411212	1.897299	-0.332509
C	-2.485656	0.477799	-0.178475
C	-1.288239	-0.278983	0.056025
C	1.288239	-0.278983	-0.056025
C	2.485656	0.477799	0.178475
C	2.411212	1.897299	0.332509
C	1.219651	2.544739	0.184515
C	1.455825	-1.642561	-0.425342
C	2.700686	-2.247364	-0.445857
C	3.858218	-1.518763	-0.102625
C	3.746105	-0.172575	0.184751
C	-3.746105	-0.172575	-0.184751
C	-3.858218	-1.518763	0.102625
C	-2.700686	-2.247364	0.445857
C	-1.455825	-1.642561	0.425342
H	-1.169657	3.635440	-0.225984
H	-3.333142	2.454840	-0.514145
H	3.333142	2.454840	0.514145
H	1.169657	3.635440	0.225984
H	4.835971	-2.005775	-0.103398
H	4.637035	0.424919	0.394176
H	-4.637035	0.424919	-0.394176
H	-4.835971	-2.005775	0.103398
H	-0.596530	-2.222154	0.751712
H	-2.784640	-3.293805	0.748287
H	2.784640	-3.293805	-0.748287
H	0.596530	-2.222154	-0.751712

TS-Carbo[4]helicene
E = -692.752548 Hartree

Symbol	X	Y	Z
C	0.000000	0.339555	0.000066
C	0.000004	1.767793	0.000024
C	-1.210682	2.528911	0.000170
C	-2.425514	1.922128	0.000212
C	-2.511677	0.499005	0.000078

C	-1.320316	-0.316056	0.000100
C	1.320317	-0.316057	0.000018
C	2.511679	0.499002	-0.000079
C	2.425523	1.922123	-0.000207
C	1.210692	2.528907	-0.000165
C	1.567138	-1.719930	0.000116
C	2.838182	-2.269302	0.000104
C	3.981928	-1.451675	0.000019
C	3.804674	-0.084997	-0.000063
C	-3.804673	-0.084988	-0.000094
C	-3.981937	-1.451664	-0.000235
C	-2.838197	-2.269300	-0.000116
C	-1.567137	-1.719935	0.000074
H	-1.130802	3.618123	0.000258
H	-3.348832	2.505774	0.000341
H	3.348842	2.505768	-0.000302
H	1.130817	3.618120	-0.000277
H	4.982639	-1.889009	0.000049
H	4.667409	0.585659	-0.000111
H	-4.667405	0.585671	-0.000110
H	-4.982652	-1.888990	-0.000425
H	-0.760805	-2.434276	0.000223
H	-2.942143	-3.356845	-0.000187
H	2.942117	-3.356847	0.000160
H	0.760807	-2.434270	0.000255

Cartesian coordinates (M06-2X/def2-SVP) of the S_0 geometries
EH2
PMM (**C1**)
E = -1503.594686 Hartree

Symbol	X	Y	Z
C	0.763160	3.185576	-0.480594
C	-0.497388	2.625450	-0.401183
C	-0.694944	1.303841	0.075856
C	0.447004	0.501296	0.334419
C	-2.128427	-0.682535	0.234993
C	-1.033915	-1.401179	0.881339
C	0.226125	-0.767980	1.012935
C	-1.248135	-2.616465	1.575053
C	-0.260444	-3.178317	2.362629
C	0.973863	-2.528812	2.515160
C	1.201390	-1.335156	1.858695
C	1.727045	1.033009	0.007611
C	3.042535	0.371752	-0.126985
N	4.017931	1.284520	-0.108259
B	3.460391	2.800156	-0.256673
C	1.903357	2.399743	-0.259775
C	3.382491	-0.996932	-0.410140

C	4.759692	-1.359456	-0.382836
C	5.738442	-0.344030	-0.187369
C	5.332480	0.956360	-0.117466
C	2.427801	-1.971313	-0.804144
C	2.816550	-3.263001	-1.067865
C	4.173502	-3.643633	-0.944839
C	5.127429	-2.709570	-0.621399
C	4.027595	3.388508	-1.661222
C	3.874602	3.679316	1.044532
C	-2.027191	0.701493	0.156336
C	-4.404282	-3.284132	-1.282004
C	-3.307489	-2.695273	-0.682075
C	-3.324891	-1.337786	-0.283089
C	-4.483139	-0.569413	-0.557453
C	-5.589748	-1.191976	-1.170391
C	-5.562453	-2.529397	-1.517525
C	-3.269252	1.476262	0.149623
C	-4.492065	0.843031	-0.188999
C	-3.319242	2.803453	0.639233
C	-4.507973	3.504746	0.709048
C	-5.703777	2.898255	0.300148
C	-5.690654	1.583037	-0.122764
H	0.872805	4.232103	-0.774934
H	-1.357037	3.212044	-0.718172
H	-2.231755	-3.082733	1.545899
H	-0.463609	-4.103154	2.904383
H	1.738059	-2.945174	3.172520
H	2.138858	-0.801618	2.017726
H	6.797877	-0.597769	-0.164427
H	6.024621	1.797909	-0.066688
H	4.464852	-4.677043	-1.138402
H	6.182902	-2.982312	-0.571218
H	3.562384	4.368160	-1.858881
H	5.117643	3.557478	-1.636921
H	3.803691	2.739809	-2.523449
H	3.422466	4.682036	0.978463
H	3.537641	3.229594	1.992262
H	4.966232	3.828761	1.108414
H	-4.356296	-4.328380	-1.593738
H	-2.393925	-3.275288	-0.560645
H	-2.411978	3.265438	1.023349
H	-4.514187	4.520175	1.107400
H	-6.645868	3.445315	0.353599
H	-6.635030	1.100759	-0.371779
H	-6.428253	-2.985645	-1.999094
H	-6.476798	-0.604775	-1.404744
H	2.073396	-3.997403	-1.379869

H	1.385932	-1.683339	-0.924843

EH3
PMM (**C1**)
E = -1657.065853 Hartree

Symbol	X	Y	Z
C	0.467032	3.508988	-0.628922
C	-0.762718	2.901331	-0.456345
C	-0.880926	1.613500	0.129898
C	0.307937	0.900500	0.427244
C	-2.172260	-0.451037	0.434824
C	-1.032357	-1.050375	1.120394
C	0.184546	-0.331739	1.188945
C	-1.155250	-2.236081	1.881573
C	-0.114022	-2.694821	2.666855
C	1.079427	-1.961834	2.754228
C	1.212480	-0.788220	2.037901
C	1.544752	1.452520	-0.004208
C	2.879470	0.824023	-0.115584
N	3.818074	1.787354	-0.151399
B	3.191834	3.255208	-0.471164
C	1.652256	2.795293	-0.392605
C	3.288492	-0.541682	-0.243281
C	4.648361	-0.821084	0.048337
C	5.572180	0.244191	0.170153
C	5.130652	1.526893	-0.028044
C	2.432470	-1.634715	-0.682238
C	2.886628	-2.968403	-0.505102
C	4.213696	-3.208811	-0.008289
C	5.078636	-2.184663	0.199634
C	1.212861	-1.435515	-1.368228
C	0.435600	-2.505189	-1.766649
C	0.843962	-3.824355	-1.497267
C	2.061375	-4.048652	-0.889230
C	3.716202	3.681292	-1.948966
C	3.591152	4.303224	0.701867
C	-2.169017	0.928990	0.261935
C	-4.237023	-3.302478	-0.923743
C	-3.191640	-2.598351	-0.356737
C	-3.313552	-1.222911	-0.044840
C	-4.522016	-0.560362	-0.372914
C	-5.574643	-1.300560	-0.949318
C	-5.445650	-2.651515	-1.211078
C	-3.462117	1.610216	0.196451
C	-4.634701	0.869305	-0.099669
C	-3.607475	2.962946	0.586142
C	-4.843195	3.581466	0.603953
C	-5.991008	2.863245	0.240569

C	-5.882867	1.525391	-0.087332
H	0.514981	4.531794	-1.010322
H	-1.658646	3.420809	-0.789863
H	-2.106932	-2.765001	1.900299
H	-0.243225	-3.602793	3.257622
H	1.884118	-2.295798	3.410569
H	2.113563	-0.185422	2.151504
H	6.625614	0.039614	0.358434
H	5.792490	2.393561	-0.063203
H	4.534402	-4.242942	0.133173
H	6.112817	-2.371530	0.492850
H	0.886210	-0.424406	-1.605892
H	-0.501715	-2.319739	-2.294267
H	0.216323	-4.664164	-1.798914
H	2.419230	-5.066230	-0.720679
H	3.223792	4.619065	-2.254402
H	4.802487	3.874106	-1.967693
H	3.492408	2.926981	-2.720539
H	3.089691	5.268786	0.526536
H	3.295082	3.958903	1.705823
H	4.674981	4.511343	0.719538
H	-4.110911	-4.358322	-1.167490
H	-2.238042	-3.099195	-0.192220
H	-2.736192	3.514050	0.934767
H	-4.923605	4.620475	0.926171
H	-6.969700	3.344539	0.254306
H	-6.789277	0.960822	-0.302536
H	-6.271416	-3.200021	-1.665979
H	-6.500605	-0.796574	-1.223853

Cartesian coordinates (M06-2X/def2-SVP) of the S_1 geometries
EH2
PMM (**C1**)
E = -1503.481514 Hartree

Symbol	X	Y	Z
C	0.723279	3.184753	-0.538508
C	-0.543401	2.623562	-0.446200
C	-0.715519	1.301963	0.045410
C	0.446076	0.482856	0.308303
C	-2.114620	-0.698910	0.272313
C	-1.042215	-1.395751	0.944485
C	0.232688	-0.764788	1.024933
C	-1.268904	-2.597559	1.654293
C	-0.265405	-3.175059	2.414661
C	0.986463	-2.551772	2.494550
C	1.228236	-1.365572	1.814608
C	1.714383	0.998606	-0.053085
C	3.016009	0.369300	-0.183902

N	3.994214	1.337039	-0.176111
B	3.408984	2.835345	-0.216040
C	1.857807	2.412718	-0.287784
C	3.377132	-0.993780	-0.460247
C	4.770262	-1.335150	-0.428348
C	5.725344	-0.288382	-0.247046
C	5.292320	1.016257	-0.186908
C	2.449176	-2.002189	-0.803594
C	2.860773	-3.312529	-1.012825
C	4.213755	-3.658795	-0.897116
C	5.156132	-2.677968	-0.623585
C	3.944273	3.617740	-1.537563
C	3.755146	3.605441	1.177871
C	-2.015446	0.715090	0.159556
C	-4.315562	-3.307250	-1.310715
C	-3.239566	-2.711694	-0.678695
C	-3.282803	-1.352391	-0.283641
C	-4.442062	-0.588615	-0.585859
C	-5.520219	-1.215855	-1.228975
C	-5.467800	-2.557200	-1.578767
C	-3.269102	1.468174	0.168039
C	-4.478230	0.822339	-0.193601
C	-3.331441	2.785172	0.674884
C	-4.531632	3.473731	0.738915
C	-5.712151	2.856436	0.312886
C	-5.682571	1.542290	-0.128845
H	0.837424	4.227415	-0.843690
H	-1.411502	3.191181	-0.774535
H	-2.265149	-3.038978	1.653976
H	-0.461745	-4.092095	2.970676
H	1.777147	-2.989570	3.105337
H	2.196916	-0.876550	1.908734
H	6.790409	-0.515853	-0.227991
H	5.987197	1.857869	-0.144227
H	4.528909	-4.692280	-1.047759
H	6.217650	-2.927582	-0.575108
H	3.449802	4.599059	-1.631312
H	5.027945	3.817943	-1.474787
H	3.758921	3.061042	-2.470132
H	3.279762	4.599591	1.202907
H	3.415144	3.050945	2.067696
H	4.842633	3.766083	1.278572
H	-4.253287	-4.350110	-1.622863
H	-2.322301	-3.279524	-0.529963
H	-2.427858	3.246130	1.069916
H	-4.554692	4.484731	1.146787
H	-6.661680	3.390469	0.364663

H	-6.619639	1.054362	-0.394323
H	-6.315595	-3.017684	-2.087456
H	-6.407825	-0.638609	-1.484933
H	2.122975	-4.072342	-1.274470
H	1.400393	-1.742952	-0.932129

EH3
PMM (**C1**)
E = -1656.953192 Hartree

Symbol	X	Y	Z
C	0.547336	3.548673	-0.556056
C	-0.699844	2.951866	-0.371700
C	-0.821801	1.651217	0.189006
C	0.369259	0.899933	0.469174
C	-2.093267	-0.424402	0.496898
C	-0.989054	-1.023673	1.209546
C	0.249664	-0.320696	1.242199
C	-1.128741	-2.216391	1.954954
C	-0.072166	-2.718319	2.697421
C	1.144271	-2.023870	2.730453
C	1.297493	-0.838724	2.023631
C	1.593295	1.422394	-0.015003
C	2.865217	0.774605	-0.209318
N	3.866792	1.720467	-0.259044
B	3.274775	3.214308	-0.492646
C	1.715620	2.814741	-0.354256
C	3.202177	-0.615084	-0.327002
C	4.531364	-0.977403	0.020793
C	5.501831	0.048129	0.150341
C	5.135022	1.367991	-0.086237
C	2.287228	-1.629449	-0.765460
C	2.601587	-2.998468	-0.513239
C	3.886907	-3.328199	0.020987
C	4.835776	-2.364926	0.217841
C	1.101502	-1.331438	-1.494117
C	0.219247	-2.326732	-1.843723
C	0.474042	-3.669996	-1.477205
C	1.655680	-3.996086	-0.843949
C	3.674821	3.714257	-1.988164
C	3.777913	4.209936	0.689273
C	-2.086609	0.987801	0.318474
C	-4.093510	-3.243378	-0.988839
C	-3.072161	-2.550917	-0.363428
C	-3.209477	-1.178478	-0.042088
C	-4.407405	-0.507356	-0.404966
C	-5.428785	-1.232925	-1.038094
C	-5.283760	-2.583449	-1.319794
C	-3.384542	1.655709	0.267916

C	-4.541872	0.916132	-0.085216
C	-3.539914	2.990899	0.702660
C	-4.781116	3.605167	0.705136
C	-5.910712	2.892285	0.289346
C	-5.789591	1.561677	-0.081680
H	0.605669	4.578384	-0.915662
H	-1.594527	3.480973	-0.694445
H	-2.096514	-2.715848	1.989344
H	-0.199724	-3.633522	3.276125
H	1.974275	-2.403058	3.328656
H	2.236564	-0.289952	2.084820
H	6.540353	-0.200702	0.364516
H	5.866611	2.178741	-0.111904
H	4.116832	-4.377728	0.216507
H	5.839793	-2.632622	0.551928
H	0.909917	-0.302750	-1.798873
H	-0.679233	-2.080069	-2.413115
H	-0.239894	-4.451905	-1.742554
H	1.891382	-5.037487	-0.613970
H	3.193931	4.680898	-2.212135
H	4.763782	3.869282	-2.077312
H	3.371472	3.002133	-2.772332
H	3.296486	5.196386	0.590098
H	3.548483	3.829718	1.697853
H	4.866759	4.381735	0.632995
H	-3.959241	-4.295271	-1.243676
H	-2.124328	-3.049731	-0.164125
H	-2.676028	3.525935	1.093473
H	-4.877134	4.632449	1.058068
H	-6.892710	3.366874	0.294302
H	-6.688250	1.002502	-0.339536
H	-6.088734	-3.121901	-1.821502
H	-6.344945	-0.726590	-1.340067

10. References

[S1] J. R. Lakowicz, *Principle of Fluorescence spectroscopy*, 2nd ed., Kluwer Academic/Plenum, New York, **1999**.
[S2] I. B. Berlman, in *Handbook of Fluorescence Spectra of Aromatic Molecules*, 2nd ed., Academic Press, New York, **1971**, p. 399.
[S3] A. J. Fry, 2nd ed., Marcel Dekker Ltd., New York, **1996**, p. 481.
[S4] J. Full, S. P. Panchal, J. Götz, A.-M. Krause, A. Nowak-Król, *Angew. Chem. Int. Ed.* **2021**, *60*, 4350-4357.
[S5] T. Tsuda, Y. Kawakami, S.-M. Choi, R. Shintani, *Angew. Chem. Int. Ed.* **2020**, *59*, 8057-8061.
[S6] K. Pati, C. Michas, D. Allenger, I. Piskun, P. S. Coutros, G. d. P. Gomes, I. V. Alabugin, *J. Org. Chem.* **2015**, *80*, 11706-11717.
[S7] G. Sheldrick, *Acta Crystallogr. A* **2015**, *71*, 3-8.
[S8] G. Sheldrick, *Acta Crystallogr. A* **2008**, *64*, 112-122.
[S9] Gaussian 16, Revision A.03, M. J. Frisch, G. W. Trucks, H. B. Schlegel, G. E. Scuseria, M. A. Robb, J. R. Cheeseman, G. Scalmani, V. Barone, G. A. Petersson, H. Nakatsuji, M. C. X. Li, A. V. Marenich, J. Bloino, B. G. Janesko, R. Gomperts, B. Mennucci, H. P. Hratchian, J. V. Ortiz, A. F. Izmaylov, J. L. Sonnenberg, D. Williams-Young, F. Ding, F. Lipparini, F. Egidi, J. Goings, B. Peng, A. Petrone, T. Henderson, D. Ranasinghe, V. G. Zakrzewski, N. R. J. Gao, G. Zheng, W. Liang, M. Hada, M. Ehara, K. Toyota, R. Fukuda, J. Hasegawa, M. Ishida, T. Nakajima, Y. Honda, O. Kitao, H. Nakai, T. Vreven, K. Throssell, J. A. Montgomery, J. E. P. Jr., F. Ogliaro, M. J. Bearpark, J. J. Heyd, E. N. Brothers, K. N. Kudin, V. N. Staroverov, T. A. Keith, R. Kobayashi, J. Normand, K. Raghavachari, A. P. Rendell, J. C. Burant, S. S. Iyengar, J. Tomasi, M. Cossi, J. M. Millam, M. Klene, C. Adamo, R. Cammi, J. W. Ochterski, R. L. Martin, K. Morokuma, O. Farkas, J. B. Foresman and D. J. Fox, Gaussian, Inc., Wallingford CT, **2016**.
[S10] a) A. D. Becke, *Phys. Rev. A* **1988**, *38*, 3098-3100; b) C. Lee, W. Yang, R. G. Parr, *Phys. Rev. B* **1988**, *37*, 785-789; c) A. D. Becke, *J. Chem. Phys.* **1993**, *98*, 5648-5652.
[S11] a) A. Schäfer, C. Huber, R. Ahlrichs, *J. Chem. Phys.* **1994**, *100*, 5829-5835; b) F. Weigend, R. Ahlrichs, *Phys. Chem. Chem. Phys.* **2005**, *7*, 3297-3305; c) F. Weigend, *Phys. Chem. Chem. Phys.* **2006**, *8*, 1057-1065.
[S12] S. Grimme, S. Ehrlich, L. Goerigk, *J. Comput. Chem.* **2011**, *32*, 1456-1465.
[S13] T. Yanai, D. P. Tew, N. C. Handy, *Chem. Phys. Lett.* **2004**, *393*, 51-57.

[S14] GaussView; Version 5, R. Dennington, T. Keith, J. Millam, Semichem Inc., Shawnee Mission KS, **2009**.
[S15] H. Kubo, T. Hirose, T. Nakashima, T. Kawai, J.-y. Hasegawa, K. Matsuda, *J. Phys. Chem. Lett.* **2021**, *12*, 686-695.
[S16] Y. Zhao, D. G. Truhlar, *Theor. Chem. Acc.* **2008**, *120*, 215-241.
[S17] W. Humphrey, A. Dalke, K. Schulten, *J. Molec. Graphics* **1996**, *14*, 33-38.
